# Sensory perception of rivals has trait-dependent effects on plasticity in *Drosophila melanogaster*

**DOI:** 10.1093/beheco/arae031

**Published:** 2024-04-24

**Authors:** Claire H Smithson, Elizabeth J Duncan, Steven M Sait, Amanda Bretman

**Affiliations:** School of Biology, Faculty of Biological Sciences, University of Leeds, Clarendon Road, Leeds, West Yorkshire, LS2 9JT, United Kingdom; School of Biology, Faculty of Biological Sciences, University of Leeds, Clarendon Road, Leeds, West Yorkshire, LS2 9JT, United Kingdom; School of Biology, Faculty of Biological Sciences, University of Leeds, Clarendon Road, Leeds, West Yorkshire, LS2 9JT, United Kingdom; School of Biology, Faculty of Biological Sciences, University of Leeds, Clarendon Road, Leeds, West Yorkshire, LS2 9JT, United Kingdom

**Keywords:** lifespan, mating duration, plasticity, sensory cues, social environment, sperm competition

## Abstract

The social environment has myriad effects on individuals, altering reproduction, immune function, cognition, and aging. Phenotypic plasticity enables animals to respond to heterogeneous environments such as the social environment but requires that they assess those environments accurately. It has been suggested that combinations of sensory cues allow animals to respond rapidly and accurately to changeable environments, but it is unclear whether the same sensory inputs are required in all traits that respond to a particular environmental cue. *Drosophila melanogaster* males, in the presence of rival males, exhibit a consistent behavioral response by extending mating duration. However, exposure to a rival also results in a reduction in their lifespan, a phenomenon interpreted as a trade-off associated with sperm competition strategies. *D. melanogaster* perceive their rivals by using multiple sensory cues; interfering with at least two olfactory, auditory, or tactile cues eliminates the extension of mating duration. Here, we assessed whether these same cues were implicated in the lifespan reduction. Removal of combinations of auditory and olfactory cues removed the extended mating duration response to a rival, as previously found. However, we found that these manipulations did not alter the reduction in lifespan of males exposed to rivals or induce any changes in activity patterns, grooming, or male–male aggression. Therefore, our analysis suggests that lifespan reduction is not a cost associated with the behavioral responses to sperm competition. Moreover, this highlights the trait-specific nature of the mechanisms underlying plasticity in response to the same environmental conditions.

## Teaser

Male fruit flies change their mating behavior when sensing rival males and have a shortened lifespan. Our study uncovers how flies respond to rivals through both smell and sound. When blocking these senses, paired flies’ mating behavior was the same as if socially isolated, but their lifespan still decreased. This highlights that responses to the same environmental cue can act through different senses.

## Introduction

Animals that are highly plastic are generally considered to be more able to respond rapidly to heterogenous environments (Gotthard and Nylin 1995; Duncan et al. 2022; Yoon et al. 2023). Being sensitive to changes in environmental conditions ensures individuals are able to alter their phenotype appropriately (Grime et al. 1986; Reznick and Yang 1993; Ellers and Van Alphen 1997; Fox et al. 2019; Yoon et al. 2023). Behavioral plasticity, one of the most rapidly flexible forms of plasticity, enables organisms to swiftly respond to environmental changes, by altering foraging tactics, seeking shelter, or avoiding predators, often within seconds of detecting stress (Altwegg 2002; Gilmour et al. 2018; Rossi et al. 2023). Plasticity can occur in different forms and timescales; fixed alternative phenotypes describe alternative developmental trajectories, sequential plasticity is a scenario where one early phenotype is replaced by another during aging, whereas labile plasticity enables individuals to switch rapidly between behavioral states (Cardoso et al. 2015). Whether these forms have similar underlying mechanisms is unclear but may include neuronal, hormonal, epigenetic, or gene regulation, with behavior perhaps accompanied by other physiological changes (Cardoso et al. 2015).

For plasticity to be adaptive, cues should accurately and reliably convey information about environmental conditions on a relevant timescale (Dewitt et al. 1998; Snell-Rood 2013). Sensory inputs must accurately reflect the environment, which can entail significant energy expenditure for environmental surveillance, processing complexity, and resource allocation (Callahan et al. 2008). Multimodal sensory perception, which allows animals to integrate information from multiple senses, leads to a comprehensive and accurate environmental representation and has been highlighted as a robust mechanism by which animals can reliably predict their environment (Fetsch et al. 2013). These sensory cues can elicit effects on multiple traits, which raises intriguing questions about the underlying mechanisms. For example, in the brown-headed cowbird (*Molothrus ater*), visual courtship displays and male song work in tandem to induce differing effects on both female courtship (Ronald et al. 2017) and male–male communication (Rothstein et al. 1988). It remains unclear whether these multi-trait effects are mediated through shared sensory pathways or interact with distinct and specialized sensory processing networks, warranting further investigation into the complex interactions between sensory cues and trait responses across multiple contexts (Ronald et al. 2017; Dore et al. 2018).

Accurate information may require multiple sensory inputs when the environmental variable is multidimensional and rapidly variable, such as the social environment (Dore et al. 2018), an influential stimulus for plasticity across a range of taxa (Maleszka et al. 2009; Bretman, Gage, et al. 2011; Oliveira 2012; Han and Brooks 2014; Cardoso et al. 2015). The social environment has myriad effects on individuals, altering reproduction, immune function, condition, and even aging (Joop and Rolff 2004; Maleszka et al. 2009; Rueppell et al. 2016; Leech et al. 2017; Fox et al. 2019). In humans, adverse social environments can have effects akin to the health impacts of well-known risk factors for chronic diseases such as smoking, obesity, or high blood pressure, leading to increased risks of depression, impaired immune function, more rapid cognitive decline, and higher rates of mortality (Cacioppo and Cacioppo 2014; Hämmig 2019).

Social environments are important in a reproductive context, signaling both mate competition and mate availability, and these can vary both spatially and temporally in wild populations (Chen and Sokolowski 2022). Differences in the number of mates or rivals will translate into different levels of opportunity or competition for reproductive resources and are therefore considered to be a complex cue (Dore et al. 2018). Consequently, males are particularly sensitive to the presence of rivals within the environment (Wedell et al. 2002; Bretman, Gage, et al. 2011; Bretman et al. 2023). Reproductive plasticity encompasses an animal’s capacity to modify its reproductive strategies in response to cues from the social environment, such as the risk of sperm competition. This plasticity is observed across a broad range of animal taxa manifested through changes to mating behavior (Crowder et al. 2010; Bretman, Gage, et al. 2011; McDowall et al. 2019; Churchill et al. 2021; Fowler et al. 2022), and ejaculate components (Wedell et al., 2002; Bretman, Gage, et al., 2011; Moatt et al., 2014) or number of offspring (Smith and Ryan 2011; Wehrtmann et al. 2012; Bretman, Westmancoat, Gage, et al. 2013).

Males use the presence of other males to predict the levels of sperm competition they face (Wedell et al. 2002; Bretman, Gage, et al. 2011; Bretman et al. 2023). In order to detect the level of sperm competition in the social environment, males can use auditory (Gray and Simmons 2013; Krobath et al. 2017; Rebar and Greenfield 2017) and olfactory perception (delBarco-Trillo and Ferkin 2004; Carazo et al. 2007; Thomas and Simmons 2009; Lane et al. 2015; Ferkin and Ferkin 2017). The role of visual cues is less clear. Some studies have suggested that vision is important in the escalation of aggressive behaviors in a range of aquatic and mammalian species (Rowland 1999; Earley and Dugatkin 2002; Luescher and Reisner 2008). However, visual cues have little to no effect on male competition in a number of insect species (Bretman, Gage, et al. 2011; Sakura et al. 2012; Ramin et al. 2014; Maguire et al. 2015; Bretman et al. 2017).

Male *D. melanogaster* demonstrate remarkable plasticity in their response to rival males, employing a combination of sensory cues to detect male presence prior to mating and accordingly increasing or decreasing mating duration depending on this prior exposure to rivals (Bretman et al. 2009, 2017, 2023; Bretman, Westmancoat, et al. 2011; Rouse and Bretman 2016). Manipulating at least 2 touch, auditory, and olfactory sensory inputs impairs rival perception such that males do not significantly extend mating duration, while inhibiting any single sense increases the time taken to show a behavioral response to a conspecific rival and increased off-target responses to other species (Bretman, Westmancoat, et al. 2011; Maguire et al. 2015; Rouse and Bretman 2016; Bretman et al. 2017; Dore et al. 2020). Overall, increasing mating duration has been shown to align with a fitness benefit potentially through increased transfer of sperm and seminal fluid proteins, but these adjustments in the ejaculate are complex and possibly sensitive to the number of rival males (Bretman, Westmancoat, Gage, et al. 2013; Hopkins et al. 2019; Bretman et al. 2023). Exposure to conspecific rivals leads to enduring consequences, including compromised immune functioning, accelerated senescence in climbing ability, and shortened lifespans compared to flies in isolation (Bretman, Westmancoat, Gage, et al. 2013; Moatt et al. 2013; Lizé et al. 2014; Leech et al. 2017). Despite experiencing such physical health effects, males exposed to rivals display enhanced cognitive performance (Rouse et al. 2020).

Although it is established that males experience longer lifespans and slower senescence in social isolation compared to those exposed to rival males (Bretman et al. 2013; Leech et al. 2017), the underlying mechanisms are unknown. It may be that the direct aggressive interactions between males decrease lifespan, though there has been little evidence found to support this idea (Bretman, Westmancoat, Gage, et al. 2013). Alternatively, the fly’s sensory perception of an increased sperm competition environment leads to increased reproductive investment at a cost to somatic maintenance. Interestingly, males physically separated from rivals through an opaque, permeable divider, where they could still utilize olfactory and auditory cues, exhibited enhanced, rather than shortened, starvation resistance (Moatt et al. 2013). This indicates that direct physical contact is not necessary for rivals to have a physiological impact.

We hypothesized that if there is a trade-off between reproductive investment and somatic maintenance, then common sensory pathways would underpin both behavioral and lifespan changes in response to male-male competition. Therefore, removing cues that males utilize to extend mating duration in response to a rival will eliminate response in any trait. Auditory and olfactory cues are already known to be important in the perception of rival presence in *D. melanogaster* (Bretman, Westmancoat, et al. 2011; Dore et al. 2020); however, it is unknown whether these sensory inputs are essential for other responses to social contact. We investigated the influence of auditory and olfactory deprivation on lifelong responses to rivals including how this affects changes to lifespan, male activity, and male–male interactive behavior. As flies lacking olfactory and auditory cues in combination are unable to increase mating duration in a response to a rival, we predicted that flies that lacked this combination of cues would likewise not show a decrease in lifespan if the 2 responses are linked. As an alternative explanation, we explored whether sensory inputs altered direct interactions, such as aggression, which could also explain decreases in the lifespan of males exposed to rivals.

## Methods

### Fly rearing

Fly rearing and all experiments were performed at 25 °C and 50% humidity with a 12:12 h light:dark cycle. Fly stocks were maintained on standard sugar-yeast-agar media (100 g brewer’s yeast, 50 g sugar, 15 g agar, 30 mL Nipagin solution (10% w/v) and 3 mL propionic acid per liter of medium) (Bass et al. 2007).

Wildtype Dahomey (Benin) *Drosophila melanogaster*, raised in mass stock cages, were provided with grape juice agar plate to lay eggs for 8 h. Larvae were transferred 24 h later to plastic vials (75 × 25 mm), raised at a density 100 larvae per 7 mL of media until eclosion. *Orco2* lines (BDSC: 23130, formally *odorant receptor 83b*) were also utilized as focal males as they lack a co-receptor responsible for perceiving 80% of *D. melanogaster*’s odor range (Larsson et al. 2004). The *Orco2* line had been backcrossed into a wildtype background by mating with Dahomey flies for 3 successive generations. The maintenance of the *Orco2* mutation was confirmed with PCR. The backcrossed *Orco2* line was raised for experimentation by placing 5 male and 5 female flies per vial and allowing them to mate and lay eggs for 48 h. After discarding the adult flies, the larvae were left to develop until eclosion. All virgin adults were sexed within 8 h of eclosion on ice anesthesia before being assigned to their social treatments after 24 h.

### Removal of sensory cues

**Fig. 1.**
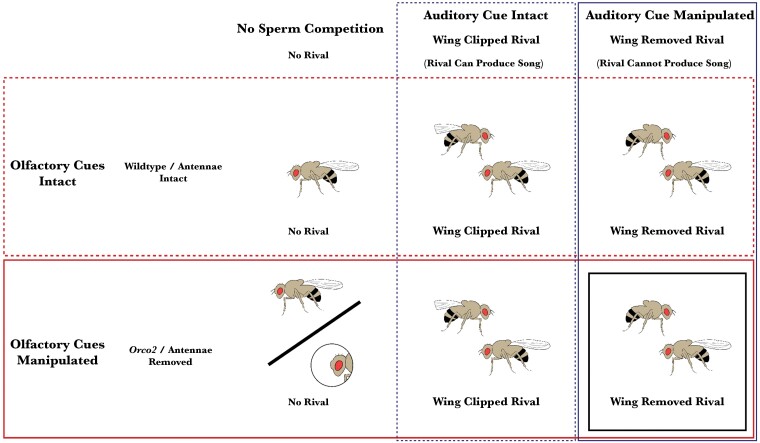
Methods schematic outlining the 6 treatment groups per experiment that interfered with male sensory perception of rival males. Exposure to a rival male increases subsequent mating duration of focal males and reduces their lifespan. Hence, here, male flies were kept in isolation or with a rival. Rival males were identified with a wing clip (which does not alter the mating duration or lifespan response by the focal male) (blue dash), or had their wings fully removed to manipulate auditory cues (solid blue). We performed two distinct manipulations of olfactory cues, using olfactory deficient flies carrying the *Orco2* mutation or removal of antennae of wildtype flies (solid red). Previous work has shown that any one of these manipulations on their own does not affect the mating duration response to a rival. However, in combination (identified by the black box), these remove the ability of focal males to increase their mating duration. We hypothesized that if these social responses are linked, this combination of cues where males do not seem to perceive the rival would also not show a decrease in lifespan. Color version of the figure is available online.

Olfaction and auditory cues were removed as in Bretman, Westmancoat, et al. (2011). When only one sensory modality was manipulated (i.e., either olfaction or auditory cues), males still increase their mating duration in response to a rival. However, when manipulated in combination, males are no longer able to increase mating duration in comparison to their single counterparts (Summarized in Fig. 1). Olfaction was manipulated in 2 ways in 2 separate experiments to account for manipulations to the focal fly. In the first experiment, the focal male carried the *Orco2* mutant, lacking a co-receptor necessary for odorant perception by all odorant receptors (Larsson et al. 2004). In the second experiment, all males were wildtype, and olfaction was manipulated through surgical removal of the entire antennae under CO_2_ (Bretman, Westmancoat, et al. 2011). Although the antennae are involved in sensory perception beyond olfaction, prior research established that removing the antennae effectively equates to a single sensory interruption in this context (Bretman, Westmancoat, et al. 2011). However, these manipulations are not fully reciprocal, as they have slightly different impacts on off-target responses to heterospecific males (Bretman et al. 2017). To account for any differences resulting from injury caused by the removal of the antennae, comparable control focal males were exposed to CO_2_ anesthesia and surgical injury to the right mesothoracic leg (Krstic et al. 2009). Note manipulations using *Orco2* or removal of antennae were entirely separate experiments on different cohorts of flies and thus are treated separately in subsequent analysis.

Auditory cues are also used to detect sperm competition risk as being provided with rivals lacking wings (hence song) in combination with manipulation of either olfaction or touch removed males’ ability to increase mating duration in response to the rival (Bretman, Westmancoat, et al. 2011). In both experiments, the focal flies were subjected to 3 social conditions cues: (1) kept alone with no exposure to sperm competition cues, (2) presence of a rival male capable of producing wing song (with clipped wing for identification), or (3) presence of a rival male with modified wings to alter the auditory cues (wing completely removed). Hence, there were 6 treatments in each experiment (Fig. 1). The modification of rival males’ wings was performed under CO_2_ anesthesia, where they either received a minor wing clip that does not influence auditory cues or had their wings completely removed to disrupt the production of auditory cues (Bretman, Westmancoat, et al. 2011).

### Mating duration

To confirm that removal of both auditory and olfactory perception influence responses to rivals, we repeated the mating duration experiments as in Bretman et al. (2011). Virgin males were randomly assigned to their social treatments for 72 h. After 72 h, males were removed from their social treatments by aspiration and exposed to a single age-matched virgin female. Females were kept in single-sex groups of 10 prior to the assay. Observation of mating was carried out blind to the treatment identity of the focal male. The flies were allowed to mate, and the duration of mating was recorded. If no mating occurred within 3 h, the vial was discarded. Mating duration assays were performed at 9 am on 3 occasions with 30 flies per treatment per experimental block.

### Lifespan measures

As in previous studies (Bretman, Westmancoat, Gage, et al. 2013; Moatt et al. 2013; Leech et al. 2017), virgin male focal flies, either unmanipulated wildtype, antennae-removed or *Orco2* mutants, were kept in their social treatments (either isolation, with a same-sex rival with a wing clip or a same-sex rival with wings removed). The starting sample size for *Orco2* was 50 flies per treatment. For the antennal-removal experiments, the initial sample size was increased to 100 flies per treatment in anticipation of early deaths resulting from surgical injuries. Yet, deaths were minimal, hence the larger overall sample size. Flies that did not die naturally, such as those lost on transfer and those that died from injuries, were excluded from the final analyses and sample size (see Fig. 3). Focal flies were monitored daily until death, and their lifespan was recorded. Rival deaths were recorded, and they were replaced with age-matched individuals. Flies were maintained on standard media, which was changed weekly.

### Behavior scores

To evaluate differences in behavior among the different treatment groups, flies were monitored weekly with behavioral scans. The experiments started 7 d after eclosion with a starting population of 50 flies in each treatment group utilizing flies within the lifespan assay experiment. Scans were then performed at 9 am weekly for a duration of 5 wk, during which fewer than 20% of flies remained in some treatment groups. Fly behavior was recorded every minute for 10 min as per Leech et al. (2017). The behavior of the focal was scored as inactive, walking, on the food, or exhibiting grooming behavior. Flies kept with a rival were also scored if they were within a body length of the rival fly or engaging in any form of aggressive behaviors (wing threats, chasing, lunging, or boxing) (Hoopfer 2016). Note it was not possible to carry out this experiment blind given that presence or absence of rivals identifies treatments.

### Ethical consideration

As invertebrates, *Drosophila melanogaster* are not subject to any specific ethical considerations for experimentation in the United Kingdom. All physical manipulations were performed under CO_2_ or ice anesthesia, and flies were given at least 24 h to recover before further experimentation.

### Statistical analysis

All statistical analyses were performed using R version 4.1.1 (R Core Team 2023) and the package “lme4” (Bates et al. 2014). Mating duration assays were analyzed using a GLMM with a Poisson distribution with olfaction and auditory manipulations as fixed factors and the date of the experiment as a random factor. Lifespan data did not have equal hazards, so it violated the assumption of a Cox regression; therefore was instead analyzed using a GLM with a Quasipoisson distribution with olfaction and auditory manipulations as fixed factors as in Leech et al. (2017). To correct for zero inflation, the “glmmTMB” package was used to model behavioral scans (Brooks et al. 2017). The number of observations of each behavior within the 10-min observation time was modeled with a Poisson distribution. Week, auditory, and olfactory modifications were added as fixed factors, and individual fly ID was included as a random effect. There were insufficient observations of grooming behaviors to compare across groups, so this behavior was excluded from the analysis. Analysis of Deviances (AOD) were used (using *F* or χ^2^ tests as appropriate) to simplify the full model, resulting in the final model when no additional terms could be eliminated without significantly diminishing the model’s descriptive power. After model selection, the chosen model was compared to the null model using AOD. Tukey tests, using the package “emmeans” (Lenth 2022), were conducted for post-hoc pairwise comparisons between groups following model selection. These comparisons focused on treatments involving the same focal fly treatment to internally control and mitigate the impact of the focal male modification on experimental outcomes. To enable easier comparison of effect sizes, we also provide tables of means and SD (Supplementary Tables S1 and S2), though for figures, we present medians and ranges to better align with our statistical analyses. Standardized effect sizes were calculated using Cohen’s *d* and can be seen in Supplementary Tables S3 (*Orco2* experiment) and Table S4 (antennae removal experiment).

## Results

### Mating duration

#### Olfactory disruption with Orco2

**Fig. 2.**
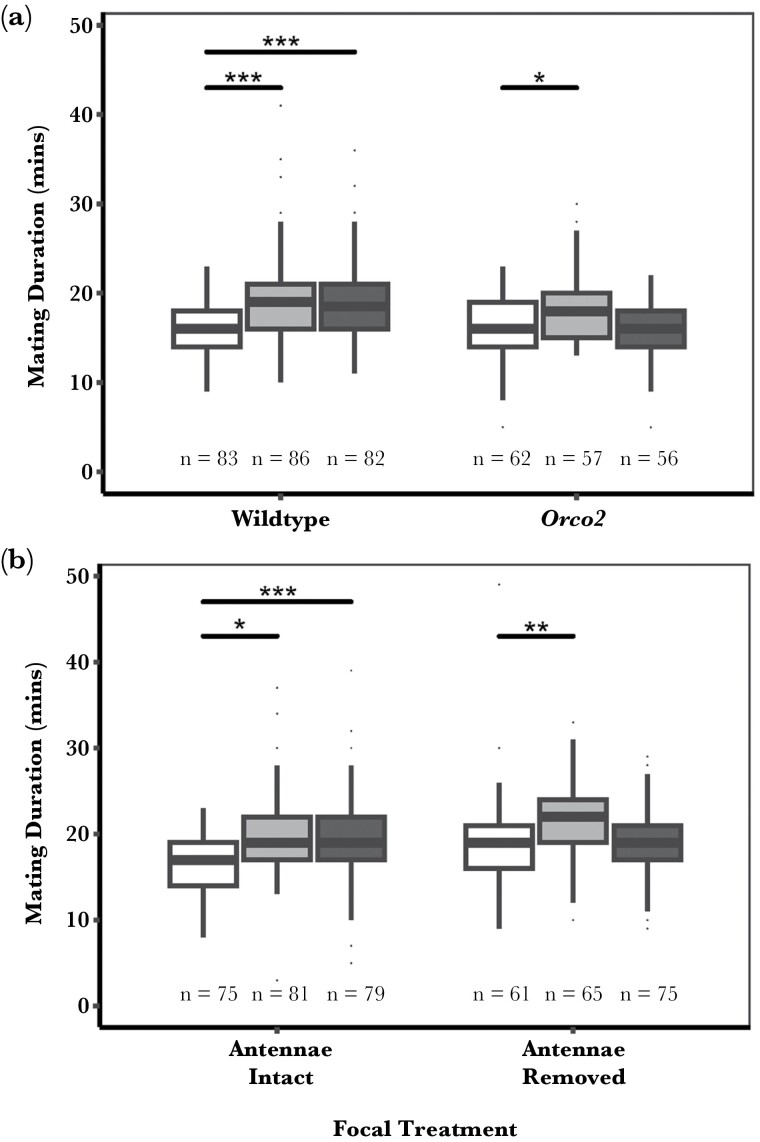
Median mating duration, in minutes, of males held singly (white bars), with a wing-clipped rival that can produce song (light gray) or with a wing-removed rival that cannot produce song (dark gray) in wildtype, unmanipulated flies compared to interrupted olfaction by (A) *Orco2* or (B) flies with antennae removed. Removing rival wings interferes with auditory cues, and the *Orco2* mutation or removal of antennae interferes with olfactory cues. Final sample sizes, excluding flies that did not die of natural causes, are given below the boxes. Significant differences between the treatments are represented by the overarching bar. * indicates a significant difference between paired treatments (*P < 0.05, **P < 0.01, ***P < 0.001).

The mating duration was affected by a significant interaction between social environment and sensory removal when using *Orco2* to remove olfactory perception (AOD; χ^2^_2 =_ 14.328, *P* < 0.001, Fig. 2A). Post-hoc tests showed that virgin wildtype male flies extended mating duration when paired with a wing-clipped conspecific or a wing-removed rival, compared to being kept in isolation (*P* < 0.001). However, while *Orco2* males did respond to the presence of a wing-clipped rival, showing a significant extension in mating duration compared to their isolated counterparts (*P* = 0.012), the scenario changed when both olfaction and rival song perception were eliminated. When multiple senses are interrupted, the focal male fly did not extend its mating duration in comparison to an *Orco2* male kept in isolation (*P* = 0.996).

#### Olfactory disruption by antennae removal

When olfaction was interrupted by removing antennae from the focal male, there was a significant interacting effect of social environment and sensory removal on mating duration (AOD; χ^2^_2 =_ 7.662, *P* = 0.022, Fig. 2B). Male flies with their antennae intact had longer mating durations when paired with a wing-clipped conspecific compared to being kept in isolation (*P* = 0.001). Both intact and antennae-removed flies had longer mating durations compared to flies kept in isolation when a single sensory cue was removed, either through removing the wing of the rival male (song removal) in the intact treatment (*P* < 0.001), or through surgical removal of the antennae (olfaction) (*P* = 0.034). However, when both olfactory and auditory cues were manipulated, the focal male fly no longer extended its mating duration compared to its internal control: males whose antennae were removed but kept in isolation (*P* = 0.999).

### Lifespan

#### Olfactory disruption with Orco2

**Fig. 3.**
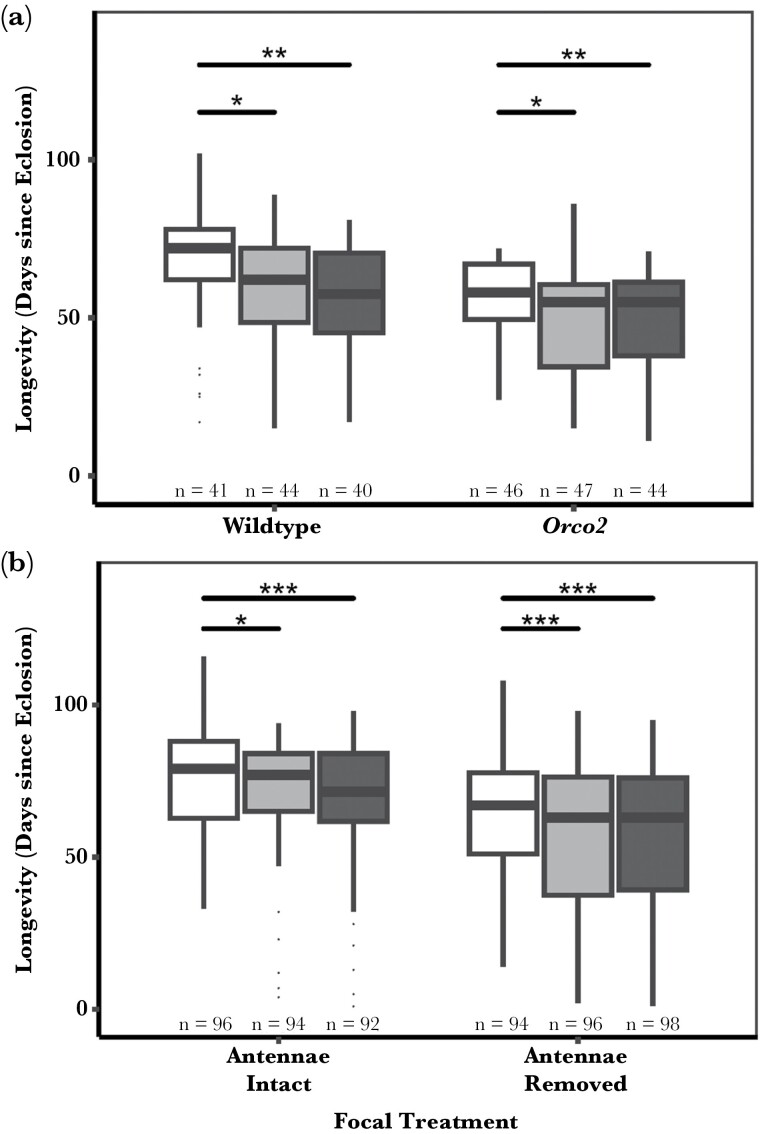
Median lifespan in days of males held singly (white bars), with a wing-clipped rival that can produce song (light gray) or with a wing removed rival that cannot produce song (dark gray) in wildtype, unmanipulated flies compared to interrupted olfaction by (A) *Orco2* or (B) flies with antennae removed. Removing rival wings interferes with auditory cues, and the *Orco2* mutation or removal of antennae interferes with olfactory cues. Final sample sizes, excluding flies that did not die of natural causes, are given below the boxes. Significant differences between the treatments are represented by the overarching bar. (*P < 0.05, **P < 0.01, ***P < 0.001).

No significant interaction was observed between the olfactory manipulation and social treatment (AOD; *F*_2,256_ = 0.097, *P* = 0.908). *Orco2* mutant flies have a significantly shorter lifespan than their wildtype counterparts (AOD; *F*_1,258_ = 19.478, *P* < 0.001). Lifespan was also significantly affected by the social treatment (AOD; *F*_2,258_ = 6.736, *P* = 0.001). When compared with flies kept in isolation, both wildtype and *Orco2* mutant virgin males kept with a rival had a significantly shorter lifespan regardless of whether the rival could (wing clipped) (*P* = 0.044) or could not (wing removed) produce a song (*P* = 0.010) (Fig. 3A).

#### Olfactory disruption by antennae removal

There was a significant interacting effect of social environment and sensory removal when removing antennae on lifespan (AOD; χ^2^_5 =_ 206.56, *P* < 0.001, Fig. 3B). Antennae-removed flies, overall, had a shorter lifespan than their antennae intact counterparts (*P* < 0.001). Virgin antennae-intact males kept with a rival had a significantly shorter lifespan compared to those kept in isolation, regardless of whether the rival could produce song (*P* = 0.017) or not (*P* < 0.001). While removing the antennae reduces the lifespan overall, the presence of a rival further impacts this lifespan, whether the rival could produce song (*P* < 0.001) or not (*P* < 0.001).

### Behavioral observations

#### Olfactory disruption with Orco2

While there was no significant interaction between sensory treatment and sensory manipulation treatments (AOD; χ^2^_2_ = 4.056, *P* = 0.132), *Orco2* flies generally spent more time on the food than their wildtype counterparts (AOD; χ^2^_1_ = 14.164, *P* < 0.001) and flies kept in isolation spending less time on the food (AOD; χ^2^_2_ = 74.301, *P* < 0.001, Supplementary Fig. S1A). Both wildtype and *Orco2* flies spent more time on the food if they were kept with a rival, regardless of whether it could produce song or not (*P* < 0.001 for all social treatments when compared to flies kept alone). All flies spent more time on the food as they aged (AOD; χ^2^_4 =_ 128.48, *P* < 0.001). While wildtype flies spent more time walking than *Orco2* (AOD; χ^2^_1_ = 119.2, *P* < 0.001), there was no significant effect of social treatment (AOD; χ^2^_2_ = 4.710, *P* = 0.095, Supplementary Fig. S1B). Unsurprisingly, *Orco2* flies, therefore, spent more time inactive than their wildtype counterparts (AOD; χ^2^_3_ = 115.73, *P* < 0.001, Supplementary Fig. S1C)

Observation of aggressive encounters between males was rare, regardless of treatment (Supplementary Fig. S2A). While there is no difference in aggression between wildtype and the *Orco2* flies (AOD; χ^2^_1_ = 0.473, *P* = 0.492), there appears to be a role of social treatment (AOD; χ^2^_1_ = 5.570, *P* = 0.018). Yet, when post-hoc analyses are performed, there are no significant differences in aggression between wildtype (*P* = 0.871) flies and marginally no difference between *Orco2* (*P* = 0.057) flies kept with a wing clipped or wing removed rival. Observations of flies within a body length of their rival did not differ between wildtype and *Orco2* (AOD; χ^2^_1_ = 1.151, *P* = 0.283) or as a result of social treatment (AOD; χ^2^_1_ = 0.023, *P* = 0.880) but significantly declined with age (AOD; χ^2^_4_ = 23.601, *P* < 0.001) (Supplementary Fig. S2B).

#### Olfactory disruption by antennae removal

Again, antennae-intact flies spent more time walking than flies lacking olfactory perception (AOD; χ^2^_2_ 26.158, *P* < 0.001), and social treatment had a significant effect on walking (AOD; χ^2^_1_ = 4.598, *P* = 0.032), but there was no interaction between the two (AOD; χ^2^_2_ = 5.601, *P* = 0.061) (Supplementary Fig. S3B). For both antennae-intact and antennae-removed flies, males kept in isolation spent more time walking than both flies kept with a rival that could (*P* = 0.007) or could not (*P* < 0.001) produce a song. The proportion of time spent walking decreased as flies aged (AOD; χ^2^_4_ = 171.34, *P* < 0.001). Antennae intact flies also spent more time inactive on the side of the vial than their antennae-removed counterparts (AOD; χ^2^_1_ = 95.738, *P* < 0.001), and the proportion of time flies spent inactive, not on the food, reduced as flies aged (AOD; χ^2^_4_ = 157.15, *P* < 0.001) (Supplementary Fig. S3C). Overall, there was no significant difference in the proportion of time spent inactive between flies kept alone and flies kept with a wing clipped (*P* = 0.191) or wing removed (*P* = 0.364) rival across the 5-wk treatment. There was a significant interaction between the time spent on the food between social treatments and olfactory modification (AOD; χ^2^_2_ = 24.15, p < 0.001) (Supplementary Fig. S3A). When comparing the treatment groups, antennae-intact flies with rivals spent more time on the food compared to flies kept alone, regardless of whether the rival could (*P* < 0.001) or could not (*P* < 0.001) produce a song. However, there was no difference between the antennae-removed group, with both wing clipped (*P* = 0.561) and wing-removed (*P* = 1.000) flies not differing in time spent on the food compared to the single treatment. Flies spent more time on the food, regardless of treatment, as they aged (AOD; χ^2^_4_ = 464.92, *P* < 0.001). Again, aggressive encounters were rare (Supplementary Fig. S4A). Flies with their antennae removed tended to show aggressive behaviors more frequently than their antennae-intact counterparts (AOD; χ^2^_1_ = 11.673, *P* = 0.006); however, there were no significant differences between rival song treatments (AOD; χ^2^_1_ = 1.213, *P* = 0.271). Observations of flies within a body length of their rival did not differ if olfaction was removed (AOD; χ^2^_1_ = 0.001, *P* = 0.978), or as a result of social treatments (AOD; χ^2^_1_ = 0.006, *P* = 0.939) (Supplementary Fig. S4B).

## Discussion

We aimed to determine if the sensory cues that enable male *D. melanogaster* to perceive a potential sperm competition rival also mediate the decrease in lifespan of males exposed to rivals exhibit. We predicted that olfactory and auditory sensory removal in combination would influence both mating duration and lifespan. As in previous studies, we found that the removal of these cues meant that males no longer responded to sperm competition in terms of mating duration. However, the same manipulations did not change the response in terms of shortened lifespan. Additionally, the detrimental effects of exposure to a rival, irrespective of which sensory cues were removed, cannot be wholly explained by the changes in behaviors that we observed.

In terms of mating duration, our results are completely in line with previous studies. Males kept in social treatments, with a wing-clipped rival that can produce song, extended their mating duration compared to flies kept alone (Bretman et al. 2009, 2010; Bretman, Gage, et al. 2011; Bretman, Westmancoat, Gage, et al. 2013; Rouse and Bretman 2016). We confirmed that they were no longer able to extend mating duration when both olfactory and auditory cues were eliminated (Bretman, Westmancoat, et al. 2011; Dore et al. 2020). In *Drosophila*, males control mating duration (Bretman, Westmancoat, and Chapman 2013); hence, the patterns we observed are unlikely to be due to a difference in female behavior. Indeed, because our design compares focal males manipulated in the same way under different social exposure, if females did respond differently to them, it would not change the interpretation. These results support the view that perception of male competition is under the control of multimodal sensory perception, to ensure a rapid yet reliable measure of the social environment (Bretman, Westmancoat, et al. 2011; Arbuthnott et al. 2017).

We hypothesized the same sensory cues that influence plasticity in mating duration in response to rivals would also control lifespan responses to rival exposure. We therefore expected that the same sensory cues that regulate mating duration changes would also have a role in the reduction in lifespan, and thus removing a combination of cues would result in a lifespan similar to flies kept in isolation. However, we found males were still negatively affected by lifelong exposure to rivals, even when both auditory and olfactory cues were removed. This is especially surprising as *Orco* olfactory mutants have previously been linked to eliminating lifespan responses to stressful social environments in *D. melanogaster* females (Chakraborty et al. 2019; Cho et al. 2021). A reduction in lifespan when exposed to rivals was previously interpreted as a cost of sperm competition responses, which are thought to be energetically costly (Bretman, Westmancoat, Gage, et al. 2013; Leech et al. 2017). Our results do not support this idea. We found that lifespan reduction was seen even when perception of sperm competition was eliminated, through manipulation of a combination of senses. Hence, we also considered various other possible factors that may have contributed to this pattern.

Understanding variations in behavior between social treatments, especially direct interaction, or competition, may offer valuable insights into the persistent lifelong effects observed in flies kept with conspecifics, despite the constraints on their sensory awareness. The decline in lifespan recorded in this investigation does not appear to be a consequence of direct competition between males. Our results show that both wildtype and *Orco2* flies kept with a rival were more likely to spend time on the food than flies kept alone, and there was no difference between any groups when flies had their antennae removed. These results thus support previous work that suggests the reduced lifespan of flies kept with a conspecific is not as a result of being actively excluded from resources (Leech et al. 2017). Furthermore, as flies aged, they spent increasingly more time on the food, though we do not know how this specifically affects the feeding rate. Interestingly, flies were found by Leech et al. (2017) to spend more time on the food if they were paired, but also if they were injured. One plausible explanation for this preference for food with aging is the potential impairment of locomotion as flies age. Given that injuries might become more prevalent or severe with age, it could lead to a diminished ability for these flies to rest on the sides of the vial as they once could, instead opting for the bottom of the vial and regular access to food sources.

Interactive behaviors (aggressive encounters and spending time within a body length of the conspecific) were rare and did not differ across manipulated treatments despite male aggression being modulated through sound (Versteven et al. 2017) and chemosensory cues in *Drosophila* (Svetec and Ferveur 2005; Wang and Anderson 2010). These results indicate that interrupting the sensory perception of a rival is likely to have a minimal influence on interactive behaviors between male conspecific flies, despite significant effects on sexual behaviors. Work in both insects and rodents indicates that aggressive encounters are trigged by novel smells and thus rapid habituation to scents and conspecifics is consistent with the low level of aggression recorded (Burn 2008; Kaidanovich-Beilin et al. 2011; Liu et al. 2011; Twick et al. 2014; Tachibana et al. 2015; Chen and Sokolowski 2022). Furthermore, Flintham et al. (2018) found that reduced lifespan was associated with receipt of, rather than initiating, aggressive encounters and courtship in males. In our study, the rival males did not have their senses moderated to the same extent as the focal flies, allowing them to still fully engage in and execute these interactive behaviors. However, while aggressive encounters have detrimental survival costs to *D. melanogaster* males (Flintham et al. 2018; Guo and Dukas 2020), and contact with rivals is costly in early life (Moatt et al. 2013), observations of aggressive encounters were rare across all treatments, in line with previous work (Bretman, Westmancoat, Gage, et al. 2013; Leech et al. 2017; Guo and Dukas 2020). It is, therefore, unlikely that aggressive interactions between males can wholly explain the reduction in survival across the entire lifespan.

When flies had their antennae removed, there was no difference in lifespan or behaviors between flies kept in isolation or kept with a rival regardless of whether the rival could or could not produce a song. Additionally, flies with their antennae removed spent more time on the food, less time walking, and more time aggressive overall than their antennae intact counterparts. The antennae are a highly sensitive sensory organ for all insects, encompassing more sensory perception than olfaction alone (Montell 2021). Nevertheless, previous research has shown that the removal of antennae yields results akin to the removal of a single sense in *D. melanogaster* (Bretman, Westmancoat, et al. 2011). However, the 2 olfactory manipulations do not have exactly the same effect, as they have slightly different outcomes in altering off-target responses to heterospecific males (Bretman et al. 2017). Interruption of olfaction in a range of arthropod species significantly reduces their ability to locate food sources (Feir and Beck 1963; Haskell and Luntz 1969; Devaney et al. 1970; Gaire et al. 2020). Flies without antennae may consequently respond more to the perception of no food resources than the perception of rival males and sperm competition risk. However, this study reveals that flies do employ their antennae to quickly adjust to a high sperm competition context and prolong their mating duration in the short term when dependence on food is likely to be less critical.

Environmental processing might not rely solely on auditory and olfactory mechanisms. Evidence suggests that visual cues (Chakraborty et al. 2019) or touch (Moatt et al. 2013) could be integral in influencing lifelong responses. Furthermore, different sensory inputs may feed into a range of biochemical, genetic, and epigenetic modifications (Rouse and Bretman 2016; Bretman et al. 2017; Dore et al. 2020). Tissue-specific responses to the environment play a significant role in trait-dependent responses to perception of the social environment. For example, both the brain and testes are important modulators of the extended mating duration phenotype (Mohorianu et al. 2017). The role of the gut, and as an extension, the gut microbiota, has been highlighted by a number of studies as increasingly important in mediating lifespan responses to the social environment (Lewis and Lizé 2015; Flintham et al. 2018; Leech et al. 2021; Proshkina et al. 2021; Tain et al. 2021). It could, therefore, be hypothesized that even if *D. melanogaster* are unable to detect high sperm competition environments through sensory perception, direct interactions between males may be sufficient to induce changes to mating behaviors and lifespan through pathways such as the microbiome (Lewis and Lizé 2015).

The social environment, and how isolation is perceived by the individual, is important in evolutionary fitness, influencing the life history outcomes across animal species, including humans (Hawkley and Capitanio 2015; Bhatti and ul Haq 2017). We have shown that the perception of isolation versus truly being alone has significant and highly variable effects on both the lifespan and behavior of the individual, even in a species not typically considered social, such as *D. melanogaster*. In our study, sensory perception plays a pivotal role in shaping responses to perceived sperm competition risk, with flies lacking this perception not extending their mating duration when a rival is present. This could place them at a reproductive disadvantage, given the potential implications for reduced sperm transfer or mate guarding. Yet, intriguingly, while sensory manipulation alters mating behaviors, it does not influence the observed lifespan reductions when flies were kept with a rival. This suggests that other factors, independent of sensory cues, might be causing this lifespan decline in competitive contexts, highlighting the nuanced nature of biological responses to competition. Underpinning why, when, and how individuals perceive being alone may have important implications across both animal welfare and human health outcomes (Cacioppo et al. 2015; Hawkley and Capitanio 2015).

Overall, this study demonstrates that although eliminating auditory and olfactory cues modifies behavioral reactions to perceived sperm competition, it does not mitigate the lifespan reduction in males or affect their interactive behaviors when confronted with competitors. This indicates that the flexibility of various traits responding to identical social cues does not necessarily originate from shared sensory pathways. Therefore, the underlying processes driving flexibility, even when prompted by the same environmental stimuli, are intricately multifaceted and vary based on the specific traits.

## Supplementary Material

arae031_suppl_Supplementary_Material

## Data Availability

Analyses reported in this article can be reproduced using the data provided by Smithson et al (2024).
