# Supplementary material for: Sensory perception of rivals has trait-dependent effects on plasticity in Drosophila melanogaster
Source: Behav Ecol. 2024 Apr 24;35(3):arae031. doi: 10.1093/beheco/arae031 (PMC11053361; doi:10.1093/beheco/arae031)
Supplement: arae031_suppl_Supplementary_Material [file arae031_suppl_supplementary_material.docx]

**Supplementary Material**

**Sensory perception of rivals has trait-dependent effects on plasticity in *Drosophila melanogaster***

Claire H. Smithson, Elizabeth J. Duncan, Steven M. Sait, Amanda Bretman

School of Biology, Faculty of Biological Science, University of Leeds, Leeds, UK, LS2 9JT

Author for correspondence: [a.j.bretman@leeds.ac.uk](mailto:a.j.bretman@leeds.ac.uk)

OrCID:

Claire H Smithson: 0000-0002-0032-8124

Elizabeth J Duncan: 0000-0002-1841-504X

Steven M Sait: 0000-0002-7208-8617

Amanda Bretman: 0000-0002-4421-3337

This document contains supplementary figures and tables (see contents table below) and R code used to analyse the associated data.

| **Figure** | **Description** | **Page** |
| --- | --- | --- |
| Figure S 1 | Activity observations for *Orco2* olfaction mutant experiment | 3 |
| Figure S 2 | Interactive behavior observations for *Orco2* olfaction mutant experiment | 5 |
| Figure S 3 | Activity observations for antennae removed experiment | 6 |
| Figure S 4 | Interactive behavior observations for antennae removed experiment | 8 |
| Table S 1 | Means and standard deviations for mating duration, lifespan, and behavioral observations for *Orco2* olfaction mutant experiment | 9 |
| Table S 2 | Means and standard deviations for mating duration, lifespan, and behavioral observations for antennae removed experiment | 10 |
| Table S 3 | Pairwise effect sizes (Cohen's*d*) for mating duration, lifespan, and behavioral observations for *Orco2* olfaction mutant experiment | 11 |
| Table S 4 | Pairwise effect sizes (Cohen's*d*) for mating duration, lifespan, and behavioral observations for antennae removed experiment | 12 |


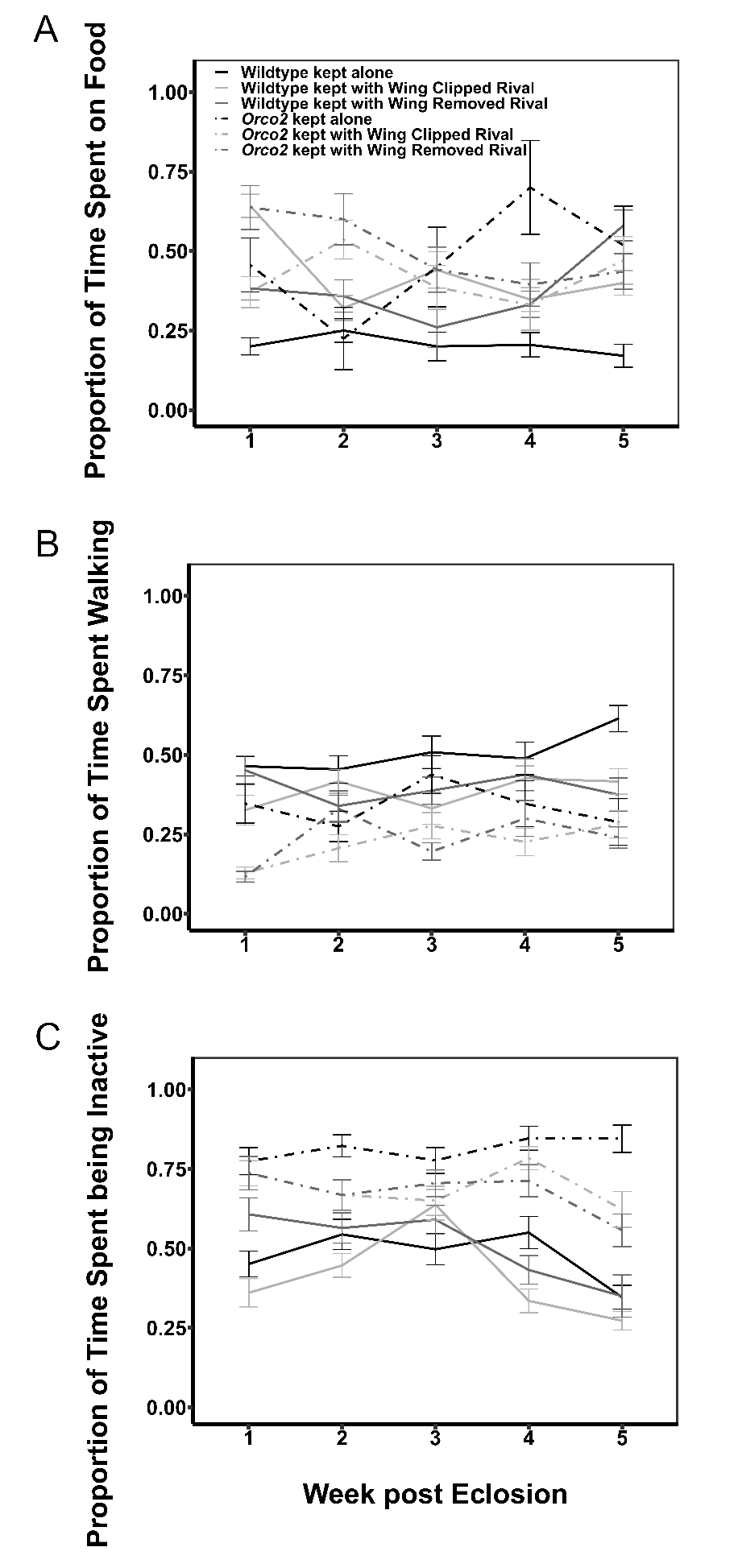


**Figure S 1****:** Activity observations of the focal wildtype (solid) or *Orco2* olfaction mutant (dash) males kept alone (black), a wing clipped rival that can produce song (light grey) or with a wing removed rival that cannot produce song (dark grey) rival once a week, for five weeks. Activity was measured by the proportion of time A) spent stationary on the food, B) spent walking, C) spent inactive within a ten minute scan period.

**
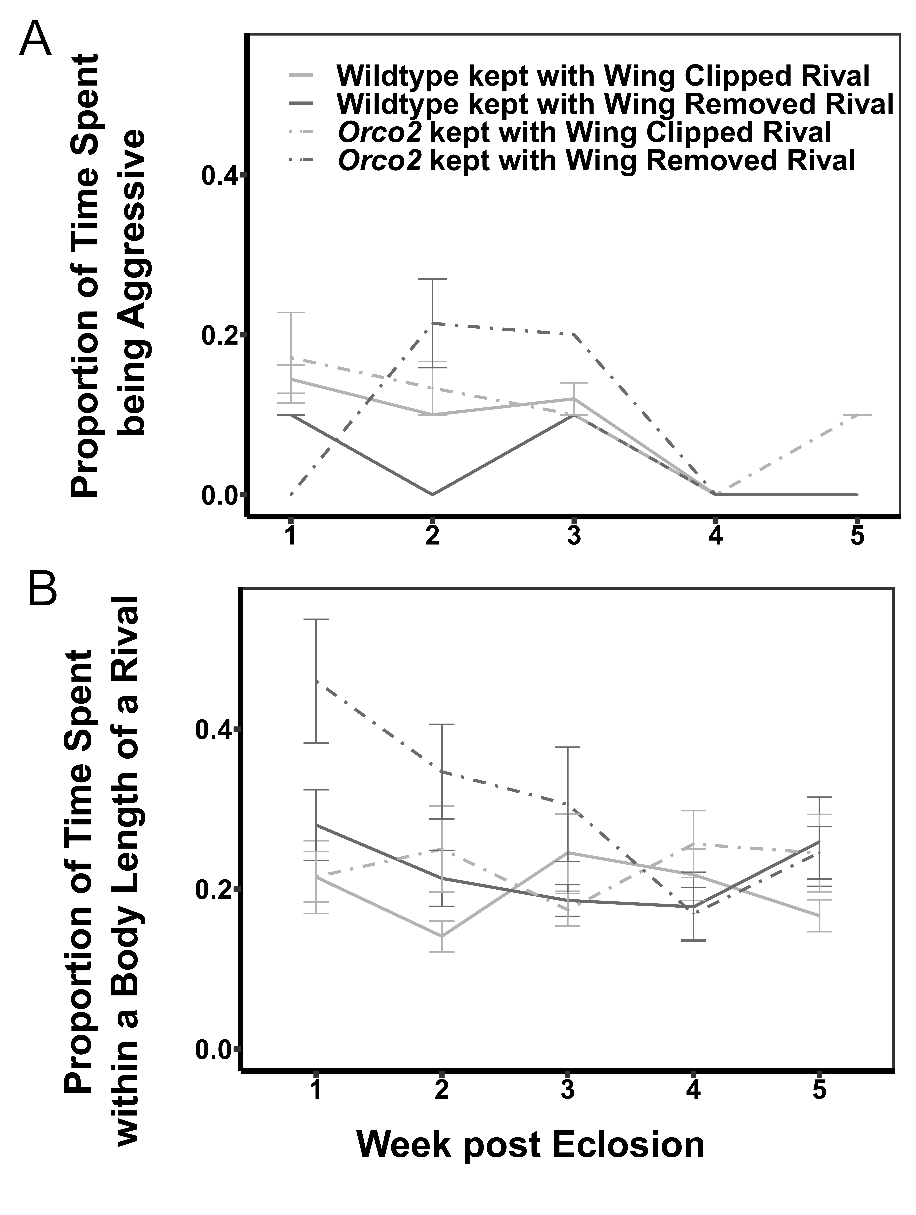
**

**Figure S 2:** Interactive behavior observations of the focal wildtype (solid) or *Orco2* olfactory mutant (dashed), with a wing clipped rival that can produce song (light grey) or a wing removed that cannot produce song (dark grey). Activity was measured by the proportion of time A) spent displaying aggressive encounters or B) spent within a body length of the conspecific male

**
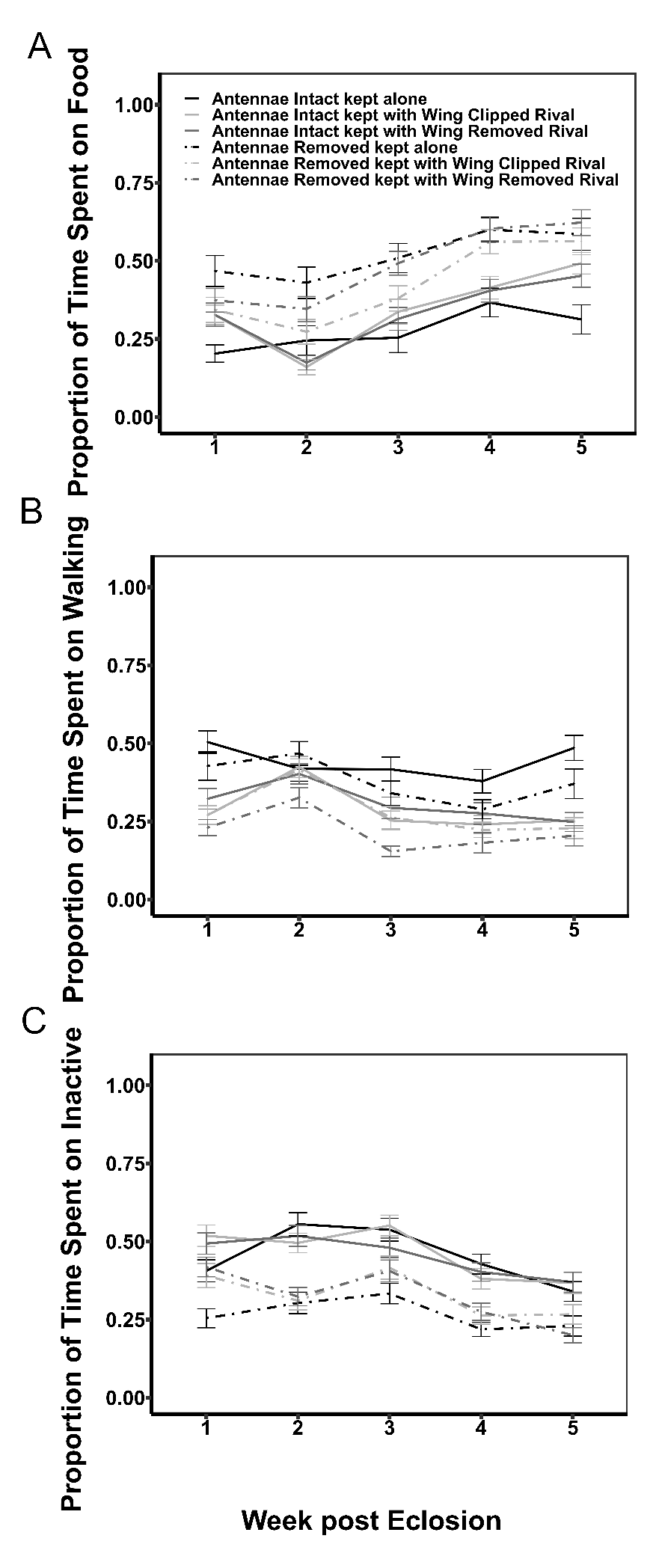
**

**Figure S 3:** Activity observations of the focal antennae intact (solid) or antennae removed lacking olfaction (dashed) males kept alone (black), with a wing clipped rival that can produce song (light grey) or a wing removed that cannot produce song (dark grey). Activity was measured by the proportion of time A) spent stationary on the food, B) spent walking, C) spent inactive within a ten minute scan period

**
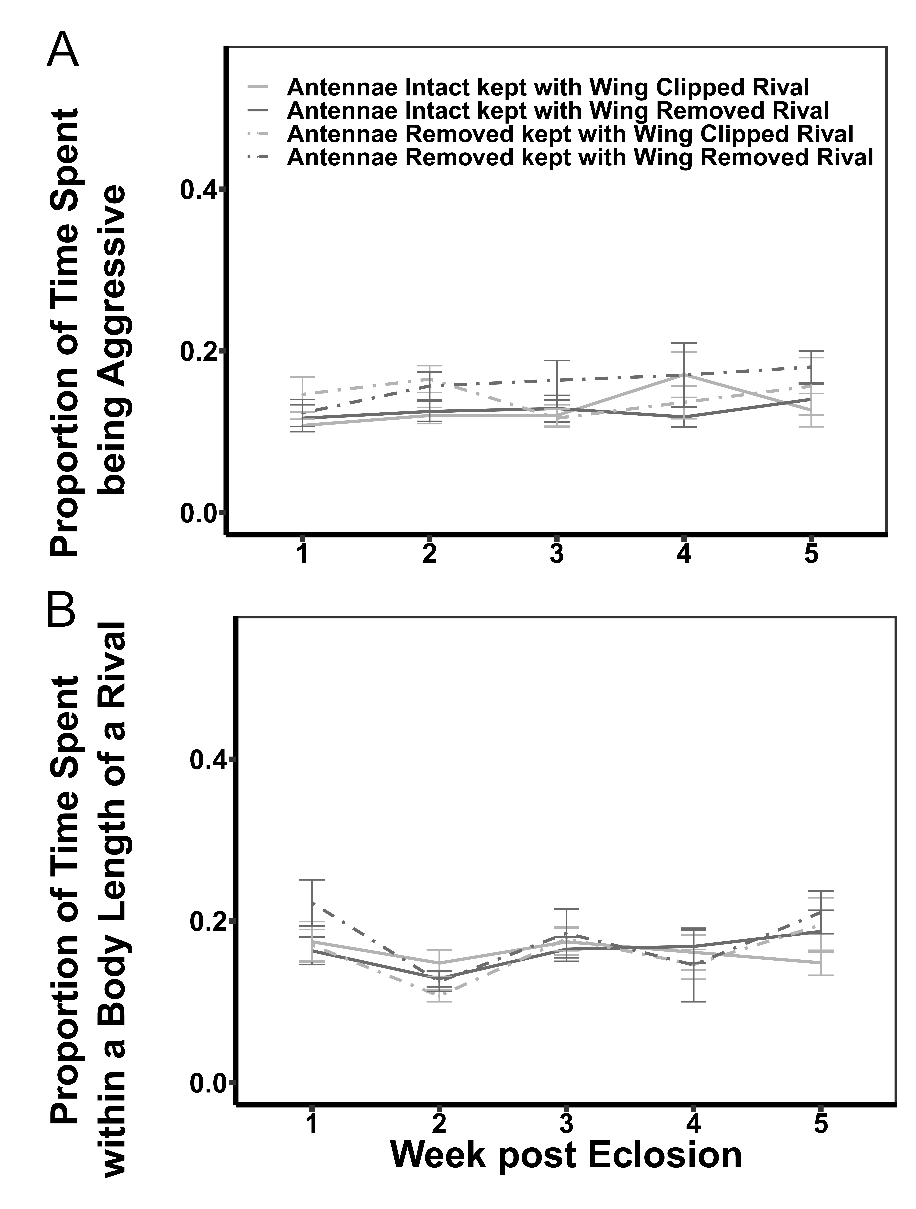
**

**Figure S 4:** Interactive behavior observations of the focal antennae intact (solid) or antennae removed lacking olfaction (dashed) with a wing clipped rival that can produce song (light grey) or a wing removed that cannot produce song (dark grey) once a week, for five weeks. Activity was measured by the proportion of time A) spent displaying aggressive encounters or B) spent within a body length of the conspecific male.

**Table S 1:** Means and standard deviations for mating duration, lifespan, and behavioral observations of focal Wildtype or *Orco2* males kept with a wing clipped (can produce song) or wing removed (cannot produce song) rival. Positive values indicate that the mean of the first group is greater than the mean of the second group. Significant Tukey post hoc pairwise comparisons identified in bold.

| Focal Treatment  (Olfactory) | Rival Treatment  (Auditory) | Mating Duration  (Minutes)  (Mean (±SD)) | Lifespan  (Days Since Eclosion)  (Mean (±SD)) | Time Spent on Food  (Proportion of Time)  (Mean (±SD)) | Time Spent Walking  (Proportion of Time)  (Mean (±SD)) | Time Spent Inactive  (Proportion of Time)  (Mean (±SD)) | Time Spent being Aggressive  (Proportion of Time)  (Mean (±SD)) | Time Spent within Body Length of Rival  (Proportion of Time)  (Mean (±SD)) |
| --- | --- | --- | --- | --- | --- | --- | --- | --- |
| Wildtype  (Olfactory Cues Intact) | Single  (No Sperm Competition) | 15.9 (±2.95) | 55.7 (±12.8) | 0.94 (±2.46) | 1.22 (±2.23) | 7.75 (±3.12) | - | - |
|  | Wing Clipped  (Auditory Cues Intact) | 19.0 (±5.03) | 48.8 (±17.0) | 2.20 (±3.07) | 1.00 (±1.71) | 6.63 (±3.22) | 0.10 (±0.37) | 0.96 (±1.58) |
|  | Wing Removed  (Manipulated Auditory Cues) | 19.1 (±4.42) | 48.4 (±17.2) | 2.62 (±3.48) | 1.06 (±1.73) | 6.20 (±3.54) | 0.02 (±0.14) | 1.25 (±2.25) |
| *Orco2*  (Manipulated Olfactory Cues) | Single  (No Sperm Competition) | 16.2 (±3.80) | 66.7 (±21.1) | 1.10 (±1.61) | 4.37 (±3.01) | 4.32 (±3.10) | - | - |
|  | Wing Clipped  (Auditory Cues Intact) | 18.6 (±3.90) | 58.4 (±16.5) | 3.64 (±3.03) | 2.76 (±2.70) | 3.40 (±2.91) | 0.09 (±0.44) | 1.04 (±1.56) |
|  | Wing Removed  (Manipulated Auditory Cues) | 15.7 (±3.55) | 55.8 (±18.4) | 2.51 (±2.82) | 2.72 (±2.81) | 4.64 (±3.43) | 0.08 (±0.48) | 0.89 (±1.57) |

**Table S 2:** Means and standard deviations for mating duration, lifespan, and behavioral observations of focal antennae intact or antennae removed males kept with a wing clipped (can produce song) or wing removed (cannot produce song) rival. Positive values indicate that the mean of the first group is greater than the mean of the second group. Significant Tukey post hoc pairwise comparisons identified in bold.

| Focal Treatment  (Olfactory) | Rival Treatment  (Auditory) | Mating Duration  (Minutes)  (Mean (±SD)) | Lifespan  (Days Since Eclosion)  (Mean (±SD)) | Time Spent on Food  (Proportion of Time)  (Mean (±SD)) | Time Spent Walking  (Proportion of Time)  (Mean (±SD)) | Time Spent Inactive  (Proportion of Time)  (Mean (±SD)) | Time Spent being Aggressive  (Proportion of Time)  (Mean (±SD)) | Time Spent within Body Length of Rival  (Proportion of Time)  (Mean (±SD)) |
| --- | --- | --- | --- | --- | --- | --- | --- | --- |
| Antennae Intact  (Olfactory Cues Intact) | Single  (No Sperm Competition) | 16.8 (±3.49) | 75.8 (±18.1) | 1.75 (±2.34) | 3.93 (±2.52) | 4.31 (±2.60) | - | - |
|  | Wing Clipped  (Auditory Cues Intact) | 19.6 (±4.65) | 71.7 (±17.8) | 2.82 (±2.56) | 2.33 (±1.97) | 4.47 (±2.45) | 0.45 (±0.76) | 0.89 (±1.05) |
|  | Wing Removed  (Manipulated Auditory Cues) | 19.6 (±4.97) | 70.0 (±19.6) | 2.98 (±2.56) | 1.74 (±1.77) | 4.39 (±2.31) | 0.37 (±0.65) | 0.79 (±1.02) |
| Antennae Removed  (Manipulated Olfactory Cues) | Single  (No Sperm Competition) | 18.8 (±5.65) | 65.1 (±21.0) | 4.79 (±3.05) | 2.84 (±2.52) | 2.35 (±1.90) | - | - |
|  | Wing Clipped  (Auditory Cues Intact) | 21.2 (±4.63) | 55.4 (±28.6) | 3.96 (±2.65) | 2.33 (±1.97) | 2.98 (±2.08) | 0.65 (±0.94) | 0.76 (±1.05) |
|  | Wing Removed  (Manipulated Auditory Cues) | 19.1 (±3.91) | 57.2 (±25.8) | 4.65 (±2.67) | 1.74 (±1.77) | 2.92 (±2.13) | 0.62 (±0.94) | 0.91 (±1.27) |

**Table S 3:** Pairwise effect sizes (Cohen's*d*) for mating duration, lifespan, and behavioral observations of focal wildtype or *Orco2* males kept with a wing clipped (can produce song) or wing removed (cannot produce song) rival. Positive values indicate that the mean of the first group is greater than the mean of the second group. Significant Tukey post hoc pairwise comparisons identified in bold.

| Focal Treatment  (Olfactory) | Rival Treatment  (Auditory) | Mating Duration  Effect Size  (Cohen’s*d*) | Lifespan  Effect Size  (Cohen’s*d*) | Time Spent on Food  Effect Size  (Cohen’s*d*) | Time Spent Walking  Effect Size  (Cohen’s*d*) | Time Spent Inactive  Effect Size  (Cohen’s*d*) | Time Spent being Aggressive  Effect Size  (Cohen’s*d*) | Time Spent within Body Length of Rival  Effect Size  (Cohen’s*d*) |
| --- | --- | --- | --- | --- | --- | --- | --- | --- |
| Wildtype  (Olfactory Cues Intact) | Single – Wing Clipped | **-0.759** | **0.443** | **-1.033** | 0.567 | **0.304** | - | - |
|  | Single – Wing Removed | **-0.854** | **0.548** | **-0.610** | 0.570 | -0.097 | - | - |
|  | Wing Clipped – Wing Removed | -0.013 | 0.144 | 0.385 | 0.016 | -0.388 | 0.028 | 0.096 |
| *Orco2*  (Manipulated Olfactory Cues) | Single – Wing Clipped | **-0.625** | **0.454** | **-0.455** | 0.108 | 0.354 | - | - |
|  | Single – Wing Removed | 0.126 | **0.480** | **-0.562** | 0.080 | **0.465** | - | - |
|  | Wing Clipped – Wing Removed | **0.769** | 0.024 | -0.129 | 0.032 | 0.126 | 0.297 | 0.146 |

**Table S 4:** Pairwise effect sizes (Cohen's*d*) for mating duration, lifespan, and behavioral observations of focal antennae intact or antennae removed males kept with a wing clipped (can produce song) or wing removed (cannot produce song) rival. Positive values indicate that the mean of the first group is greater than the mean of the second group. Significant Tukey post hoc pairwise comparisons identified in bold.

| Focal Treatment  (Olfactory) | Rival Treatment  (Auditory) | Mating Duration  Effect Size  (Cohen’s*d*) | Lifespan  Effect Size  (Cohen’s*d*) | Time Spent on Food  Effect Size  (Cohen’s*d*) | Time Spent Walking  Effect Size  (Cohen’s*d*) | Time Spent Inactive  Effect Size  (Cohen’s*d*) | Time Spent being Aggressive  Effect Size  (Cohen’s*d*) | Time Spent within Body Length of Rival  Effect Size  (Cohen’s*d*) |
| --- | --- | --- | --- | --- | --- | --- | --- | --- |
| Antennae Intact  (Olfactory Cues Intact) | Single – Wing Clipped | **-0.677** | **0.228** | **-0.438** | **0.688** | -0.061 | - | - |
|  | Single – Wing Removed | **-0.669** | **0.307** | **-0.504** | **0.691** | -0.032 | - | - |
|  | Wing Clipped – Wing Removed | -0.331 | 0.090 | -0.063 | 0.010 | 0.032 | -0.121 | 0.100 |
| Antennae Removed  (Manipulated Olfactory Cues) | Single – Wing Clipped | **-0.462** | **0.385** | 0.288 | **0.221** | -0.318 | - | - |
|  | Single – Wing Removed | -0.065 | **0.335** | 0.049 | **0.506** | -0.285 | - | - |
|  | Wing Clipped – Wing Removed | **0.146** | -0.065 | -0.258 | 0.320 | 0.027 | 0.041 | 0.131 |

**Data analysis R code associated with “Sensory perception of sperm competition risk has trait-dependent effects on plasticity in *Drosophila melanogaster”***

# Orco

Orco2 (Olfactory Receptor Coreceptor) is necessary for olfactory reception in Drosophila. Mutants in these gene lack olfactory reception.

## Extended Mating Duration Analysis

**Figure 1A**

**Description of Data:** Experiment on mating duration conducted on 30th June 2022 compared the mating times of WT and Orco2 flies when kept solo versus when they were with a rival having a clipped wing or a completely removed wing.

**Factors:**

- FlyID-Unique ID for each fly within experiment
- DateEclosed-Date fly eclosed as an adult
- DateExperiment-Date mating experiment performed
- FocalTreatment-Fly Genotype. (WT=not manipulated, Orco=Orco2 olfactory mutant)
- RivalTreatment-Social Treatment. Single=focal fly kept in isolation post eclosion, WingClip=fly kept with single rival with a wing clip from eclosion to mating, WingRemoved=fly kept with a rival with wings surgical removed
- Treatment-Interaction between Focal and Rival Treatments
- Mated?-Flies mated within 3h (Y= succesfully mated, N= didn’t mate within 3h, NA = excluded from experiment)
- TimeUp-Time when male aspirated into vial with female (to nearest minute)
- TimeMatingStart-Time when mating started (to nearest minute)
- TimeMatingEnd-Time when mating ended (to nearest minute)
- MatingLatency-Time taken to start mating (TimeMatingStart - TimeUp)
- MatingDuration-Mating Duration (TimeMatingEnd - TimeMatingStart)

##Library
library(emmeans)
library(lme4)

Loading required package: Matrix

library(ggplot2)
library(tidyverse)

── Attaching core tidyverse packages ──────────────────────── tidyverse 2.0.0 ──
✔ dplyr 1.1.3 ✔ readr 2.1.4
✔ forcats 1.0.0 ✔ stringr 1.5.0
✔ lubridate 1.9.2 ✔ tibble 3.2.1
✔ purrr 1.0.2 ✔ tidyr 1.3.0

── Conflicts ────────────────────────────────────────── tidyverse_conflicts() ──
✖ tidyr::expand() masks Matrix::expand()
✖ dplyr::filter() masks stats::filter()
✖ dplyr::lag() masks stats::lag()
✖ tidyr::pack() masks Matrix::pack()
✖ tidyr::unpack() masks Matrix::unpack()
ℹ Use the conflicted package (<http://conflicted.r-lib.org/>) to force all conflicts to become errors

library(ggsignif)
library(AER)

Loading required package: car
Loading required package: carData

Attaching package: 'car'

The following object is masked from 'package:dplyr':

 recode

The following object is masked from 'package:purrr':

 some

Loading required package: lmtest
Loading required package: zoo

Attaching package: 'zoo'

The following objects are masked from 'package:base':

 as.Date, as.Date.numeric

Loading required package: sandwich
Loading required package: survival

library(lsr)
library(effsize)

##Importing Data
OrcoEMD1 <- read.csv("Orco_EMD_30.6.2022.csv")
OrcoEMD2 <- read.csv("Orco_EMD_27.7.2022.csv")
OrcoEMD3 <- read.csv("Orco_EMD_3.8.2022.csv")

##Combining Data
OrcoEMD_Combined <- bind_rows(OrcoEMD1, OrcoEMD2, OrcoEMD3)

#Removing Outliers
OrcoEMD_5 <- subset(OrcoEMD_Combined, MatingDuration > 4)
OrcoEMD_60 <- subset(OrcoEMD_5, MatingDuration < 45)
OrcoEMD_All <- subset(OrcoEMD_60, Mated. == "Y")

##Plotting the Data
#Ordering Factors
OrcoEMD_All$Rival_Treatment <- factor(OrcoEMD_All$Rival_Treatment, levels = c("Single", "WingClip", "WingRemoved"))
OrcoEMD_All$Focal_Treatment <- factor(OrcoEMD_All$Focal_Treatment, levels = c("WT", "Orco"))


#Box Plot
OrcoEMD_All$Treatment <- gsub(" ", "", OrcoEMD_All$Treatment)
OrcoEMD_All$Treatment <- factor(OrcoEMD_All$Treatment, levels = c("WTSingle", "WTWingClip", "WTWingRemoved", "OrcoSingle", "OrcoWingClip", "OrcoWingRemoved"))

orco_matingduration_sample_sizes <- OrcoEMD_All %>%
 group_by(Focal_Treatment, Rival_Treatment, Treatment) %>%
 dplyr::summarise(
 Count = n(),
 Mean_MatingDuration = mean(MatingDuration))

`summarise()` has grouped output by 'Focal_Treatment', 'Rival_Treatment'. You
can override using the `.groups` argument.

orco_matingduration_sample_sizes$Count <- as.character(orco_matingduration_sample_sizes$Count)

boxplot_orco_matingduration_bw <- ggplot(OrcoEMD_All) +
 aes(
 x = Focal_Treatment,
 y = MatingDuration,
 fill = Rival_Treatment
 ) +
 geom_boxplot(size = 5) +
 scale_fill_manual(
 values = c(Single = "white",
 WingClip = "grey70",
 WingRemoved = "grey25"),
 labels=c("Single" = "Single",
 "WingClip" = "Wing Clipped",
 "WingRemoved" = "Wing Removed")
 ) +
 labs(
 x = "Focal Treatment",
 y = "Mating Duration (mins)",
 fill = "Social Treatment"
 ) +
 ylim(0,49)+
 geom_signif(y_position = c(43, 47, 43),
 xmin = c(0.75, 0.75, 1.75),
 xmax = c(1, 1.25, 2),
 annotation = c("***","***", "*"),
 tip_length = 0, textsize= 25,
 size = 5) +
 theme_bw()


boxplot_orco_matingduration_bw +
 theme_bw() + theme(panel.grid.major = element_blank(), panel.grid.minor = element_blank()) +
 theme(axis.text=element_text(size=55, face="bold", colour="black"),
 axis.title=element_text(size=75, face="bold", colour="black"),
 legend.text=element_text(size=55, face="bold", colour="black"),
 legend.title=element_text(size=75, face="bold", colour="black")) +
 theme(axis.line = element_line(size = 5),
 panel.border = element_rect(size = 5)) +
 theme(
 axis.ticks.length = unit(0.5, "cm"),
 axis.ticks = element_line(size = 5) ) +
 scale_x_discrete(labels=c("WT" = "Wildtype", "Orco" = "Orco2")) +
 geom_text(
 data = orco_matingduration_sample_sizes,
 aes(
 x = Focal_Treatment,
 y = 0.1,
 label = paste0("n=", Count)
 ),
 position = position_dodge(width = 0.75),
 vjust = -0.5,
 size = 12
 )

Warning: The `size` argument of `element_line()` is deprecated as of ggplot2 3.4.0.
ℹ Please use the `linewidth` argument instead.

Warning: The `size` argument of `element_rect()` is deprecated as of ggplot2 3.4.0.
ℹ Please use the `linewidth` argument instead.


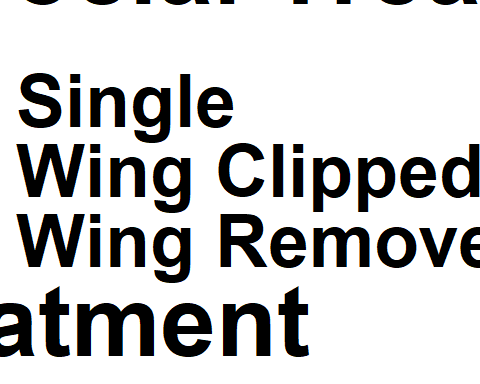


##Data Analysis
#testing data normality
qqnorm(OrcoEMD_All$MatingDuration)


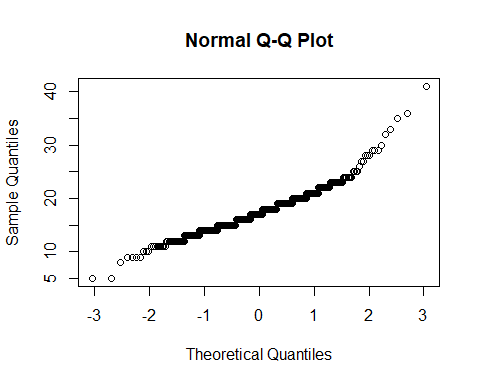


shapiro.test(OrcoEMD_All$MatingDuration)

Shapiro-Wilk normality test

data: OrcoEMD_All$MatingDuration
W = 0.9435, p-value = 1.165e-11

#data is not normal

matingduration_glmer1 <- glmer(MatingDuration ~ Focal_Treatment * Rival_Treatment + (1|Date_Experiment), data = OrcoEMD_All,
 family = poisson(link=log))
summary(matingduration_glmer1)

Generalized linear mixed model fit by maximum likelihood (Laplace
 Approximation) [glmerMod]
 Family: poisson ( log )
Formula:
MatingDuration ~ Focal_Treatment * Rival_Treatment + (1 | Date_Experiment)
 Data: OrcoEMD_All

 AIC BIC logLik deviance df.resid
 2379.2 2407.6 -1182.6 2365.2 419

Scaled residuals:
 Min 1Q Median 3Q Max
-2.6718 -0.6441 -0.0799 0.4874 5.0997

Random effects:
 Groups Name Variance Std.Dev.
 Date_Experiment (Intercept) 0.00146 0.0382
Number of obs: 426, groups: Date_Experiment, 3

Fixed effects:
 Estimate Std. Error z value
(Intercept) 2.766028 0.035275 78.413
Focal_TreatmentOrco 0.008844 0.042046 0.210
Rival_TreatmentWingClip 0.179380 0.036987 4.850
Rival_TreatmentWingRemoved 0.184221 0.037367 4.930
Focal_TreatmentOrco:Rival_TreatmentWingClip -0.033412 0.057616 -0.580
Focal_TreatmentOrco:Rival_TreatmentWingRemoved -0.207810 0.059438 -3.496
 Pr(>|z|)
(Intercept) < 2e-16 ***
Focal_TreatmentOrco 0.833405
Rival_TreatmentWingClip 1.24e-06 ***
Rival_TreatmentWingRemoved 8.22e-07 ***
Focal_TreatmentOrco:Rival_TreatmentWingClip 0.561981
Focal_TreatmentOrco:Rival_TreatmentWingRemoved 0.000472 ***
---
Signif. codes: 0 '***' 0.001 '**' 0.01 '*' 0.05 '.' 0.1 ' ' 1

Correlation of Fixed Effects:
 (Intr) Fcl_TO Rv_TWC Rv_TWR F_TO:R_TWC
Fcl_TrtmntO -0.510
Rvl_TrtmnWC -0.580 0.488
Rvl_TrtmnWR -0.575 0.483 0.548
Fc_TO:R_TWC 0.372 -0.728 -0.642 -0.351
Fc_TO:R_TWR 0.361 -0.704 -0.345 -0.628 0.514

matingduration_glmer2 <- glmer(MatingDuration ~ Focal_Treatment + Rival_Treatment + (1|Date_Experiment), data = OrcoEMD_All,
 family = poisson(link=log))
anova(matingduration_glmer1, matingduration_glmer2, test = "Chi")

Data: OrcoEMD_All
Models:
matingduration_glmer2: MatingDuration ~ Focal_Treatment + Rival_Treatment + (1 | Date_Experiment)
matingduration_glmer1: MatingDuration ~ Focal_Treatment * Rival_Treatment + (1 | Date_Experiment)
 npar AIC BIC logLik deviance Chisq Df Pr(>Chisq)
matingduration_glmer2 5 2389.5 2409.8 -1189.8 2379.5
matingduration_glmer1 7 2379.2 2407.6 -1182.6 2365.2 14.328 2 0.0007739

matingduration_glmer2
matingduration_glmer1 ***
---
Signif. codes: 0 '***' 0.001 '**' 0.01 '*' 0.05 '.' 0.1 ' ' 1

matingduration_glmer3 <- glmer(MatingDuration ~ Focal_Treatment + (1|Date_Experiment), data = OrcoEMD_All,
 family = poisson(link=log))
anova(matingduration_glmer1, matingduration_glmer3, test = "Chi")

Data: OrcoEMD_All
Models:
matingduration_glmer3: MatingDuration ~ Focal_Treatment + (1 | Date_Experiment)
matingduration_glmer1: MatingDuration ~ Focal_Treatment * Rival_Treatment + (1 | Date_Experiment)
 npar AIC BIC logLik deviance Chisq Df Pr(>Chisq)
matingduration_glmer3 3 2419.6 2431.7 -1206.8 2413.6
matingduration_glmer1 7 2379.2 2407.6 -1182.6 2365.2 48.354 4 7.963e-10

matingduration_glmer3
matingduration_glmer1 ***
---
Signif. codes: 0 '***' 0.001 '**' 0.01 '*' 0.05 '.' 0.1 ' ' 1

matingduration_glmer4 <- glmer(MatingDuration ~ Rival_Treatment + (1|Date_Experiment), data = OrcoEMD_All,
 family = poisson(link=log))
anova(matingduration_glmer1, matingduration_glmer4, test = "Chi")

Data: OrcoEMD_All
Models:
matingduration_glmer4: MatingDuration ~ Rival_Treatment + (1 | Date_Experiment)
matingduration_glmer1: MatingDuration ~ Focal_Treatment * Rival_Treatment + (1 | Date_Experiment)
 npar AIC BIC logLik deviance Chisq Df Pr(>Chisq)
matingduration_glmer4 4 2396.2 2412.4 -1194.1 2388.2
matingduration_glmer1 7 2379.2 2407.6 -1182.6 2365.2 23.007 3 4.025e-05

matingduration_glmer4
matingduration_glmer1 ***
---
Signif. codes: 0 '***' 0.001 '**' 0.01 '*' 0.05 '.' 0.1 ' ' 1

matingduration_glmer_null <- glmer(MatingDuration ~ (1|Date_Experiment), data = OrcoEMD_All,
 family = poisson(link=log))
anova(matingduration_glmer1, matingduration_glmer_null, test = "Chi")

Data: OrcoEMD_All
Models:
matingduration_glmer_null: MatingDuration ~ (1 | Date_Experiment)
matingduration_glmer1: MatingDuration ~ Focal_Treatment * Rival_Treatment + (1 | Date_Experiment)
 npar AIC BIC logLik deviance Chisq Df
matingduration_glmer_null 2 2427.1 2435.2 -1211.6 2423.1
matingduration_glmer1 7 2379.2 2407.6 -1182.6 2365.2 57.919 5
 Pr(>Chisq)
matingduration_glmer_null
matingduration_glmer1 3.268e-11 ***
---
Signif. codes: 0 '***' 0.001 '**' 0.01 '*' 0.05 '.' 0.1 ' ' 1

#post hoc tests
lsmeans(matingduration_glmer1, pairwise ~ Focal_Treatment * Rival_Treatment, adjust="tukey")

$lsmeans
 Focal_Treatment Rival_Treatment lsmean SE df asymp.LCL asymp.UCL
 WT Single 2.77 0.0353 Inf 2.70 2.84
 Orco Single 2.77 0.0387 Inf 2.70 2.85
 WT WingClip 2.95 0.0331 Inf 2.88 3.01
 Orco WingClip 2.92 0.0379 Inf 2.85 3.00
 WT WingRemoved 2.95 0.0335 Inf 2.88 3.02
 Orco WingRemoved 2.75 0.0404 Inf 2.67 2.83

Results are given on the log (not the response) scale.
Confidence level used: 0.95

$contrasts
 contrast estimate SE df z.ratio p.value
 WT Single - Orco Single -0.00884 0.0420 Inf -0.210 0.9999
 WT Single - WT WingClip -0.17938 0.0370 Inf -4.850 <.0001
 WT Single - Orco WingClip -0.15481 0.0413 Inf -3.748 0.0025
 WT Single - WT WingRemoved -0.18422 0.0374 Inf -4.930 <.0001
 WT Single - Orco WingRemoved 0.01475 0.0437 Inf 0.338 0.9994
 Orco Single - WT WingClip -0.17054 0.0402 Inf -4.239 0.0003
 Orco Single - Orco WingClip -0.14597 0.0442 Inf -3.306 0.0122
 Orco Single - WT WingRemoved -0.17538 0.0406 Inf -4.321 0.0002
 Orco Single - Orco WingRemoved 0.02359 0.0463 Inf 0.510 0.9958
 WT WingClip - Orco WingClip 0.02457 0.0395 Inf 0.622 0.9894
 WT WingClip - WT WingRemoved -0.00484 0.0353 Inf -0.137 1.0000
 WT WingClip - Orco WingRemoved 0.19412 0.0419 Inf 4.629 0.0001
 Orco WingClip - WT WingRemoved -0.02941 0.0398 Inf -0.739 0.9771
 Orco WingClip - Orco WingRemoved 0.16956 0.0456 Inf 3.715 0.0028
 WT WingRemoved - Orco WingRemoved 0.19897 0.0422 Inf 4.712 <.0001

Results are given on the log (not the response) scale.
P value adjustment: tukey method for comparing a family of 6 estimates

#effect sizes
Orco_group_stats_EMD <- OrcoEMD_All %>%
 group_by(Focal_Treatment, Rival_Treatment) %>%
 dplyr::summarise(mean = mean(MatingDuration),
 sd = sd(MatingDuration),
 N = n())

`summarise()` has grouped output by 'Focal_Treatment'. You can override using
the `.groups` argument.

OrcoExpEMD_WT_Single <- OrcoEMD_All %>%
 filter(Rival_Treatment == "Single", Focal_Treatment == "WT") %>%
 select(MatingDuration)
OrcoExpEMD_WT_WC <- OrcoEMD_All %>%
 filter(Rival_Treatment == "WingClip", Focal_Treatment == "WT") %>%
 select(MatingDuration)
OrcoExpEMD_WT_WR <- OrcoEMD_All %>%
 filter(Rival_Treatment == "WingRemoved", Focal_Treatment == "WT") %>%
 select(MatingDuration)
cohen.d(OrcoExpEMD_WT_Single$MatingDuration, OrcoExpEMD_WT_WC$MatingDuration)

Cohen's d

d estimate: -0.7590572 (medium)
95 percent confidence interval:
 lower upper
-1.0735848 -0.4445295

cohen.d(OrcoExpEMD_WT_Single$MatingDuration, OrcoExpEMD_WT_WR$MatingDuration)

Cohen's d

d estimate: -0.8540302 (large)
95 percent confidence interval:
 lower upper
-1.1751937 -0.5328666

cohen.d(OrcoExpEMD_WT_WR$MatingDuration, OrcoExpEMD_WT_WC$MatingDuration)

Cohen's d

d estimate: 0.01334235 (negligible)
95 percent confidence interval:
 lower upper
-0.2913974 0.3180821

OrcoExpEMD_Orco_Single <- OrcoEMD_All %>%
 filter(Rival_Treatment == "Single", Focal_Treatment == "Orco") %>%
 select(MatingDuration)
OrcoExpEMD_Orco_WC <- OrcoEMD_All %>%
 filter(Rival_Treatment == "WingClip", Focal_Treatment == "Orco") %>%
 select(MatingDuration)
OrcoExpEMD_Orco_WR <- OrcoEMD_All %>%
 filter(Rival_Treatment == "WingRemoved", Focal_Treatment == "Orco") %>%
 select(MatingDuration)
cohen.d(OrcoExpEMD_Orco_Single$MatingDuration, OrcoExpEMD_Orco_WC$MatingDuration)

Cohen's d

d estimate: -0.6247269 (medium)
95 percent confidence interval:
 lower upper
-0.9968864 -0.2525675

cohen.d(OrcoExpEMD_Orco_Single$MatingDuration, OrcoExpEMD_Orco_WR$MatingDuration)

Cohen's d

d estimate: 0.1258692 (negligible)
95 percent confidence interval:
 lower upper
-0.2396264 0.4913648

cohen.d(OrcoExpEMD_Orco_WR$MatingDuration, OrcoExpEMD_Orco_WC$MatingDuration)

Cohen's d

d estimate: -0.768755 (medium)
95 percent confidence interval:
 lower upper
-1.1551147 -0.3823953

## Lifespan

**Figure 2A**

**Description of Data:** Lifespan assay comparing effects of social treatment and senses of WT and Orco2 flies when kept solo versus when they were with a rival having a clipped wing or a completely removed wing.

**Factors:**

- ID-Unique Fly ID
- Number-Unique Fly ID (this experiment)
- Focal_Treatment-Focal fly genotye. (WT=wildtype, Orco= Orco2 olfactory mutant)
- Rival_Treatment-Social Treatment. Single=focal fly kept in isolation post eclosion, WingClip=fly kept with single rival with a wing clip from eclosion to mating, WingRemoved=fly kept with a rival with wings surgical removed
- Treatment-Interaction between Focal and Rival Treatments
- Date_Eclosed-Day experiment started
- Date_Death-Day fly finished experiment (died or lost)
- Censoring_Status-Censoring Status (1=fly died naturally, 0=fly didn't die naturally (e.g. lost on transfer)
- Lifespan-Lifespan in days
- Number_Rival_Changes-Number of times rival fly replaced
- Rival_Changed?-Date of rival changes
- Notes-Notes

###loading packages
library(ggplot2)
library(ggpubr)
library(survival)
library(survminer)

Attaching package: 'survminer'

The following object is masked from 'package:survival':

 myeloma

library(dplyr)
library(emmeans)
library(lme4)
library(AER)
library(effsize)

##Importing Data
OrcoLifespan <- read.csv("Orco_Perception_Data.csv", header = TRUE)
OrcoLifespan_Dead <- subset(OrcoLifespan, Censoring_Status == "1" )

#Creating Survival Plot
surv_object_orco <- Surv(time=OrcoLifespan$Lifespan, event=OrcoLifespan$Censoring_Status)
surv_fit_orco <- survfit(surv_object_orco ~ Treatment, data = OrcoLifespan)
summary(surv_fit_orco)

Call: survfit(formula = surv_object_orco ~ Treatment, data = OrcoLifespan)

20 observations deleted due to missingness
 Treatment=OrcoSingle
 time n.risk n.event survival std.err lower 95% CI upper 95% CI
 10 48 2 0.9583 0.0288 0.9034 1.000
 24 46 1 0.9375 0.0349 0.8715 1.000
 25 45 1 0.9167 0.0399 0.8417 0.998
 31 44 1 0.8958 0.0441 0.8135 0.987
 33 43 1 0.8750 0.0477 0.7863 0.974
 37 42 1 0.8542 0.0509 0.7599 0.960
 38 41 2 0.8125 0.0563 0.7093 0.931
 39 39 2 0.7708 0.0607 0.6606 0.899
 46 37 1 0.7500 0.0625 0.6370 0.883
 47 36 1 0.7292 0.0641 0.6137 0.866
 49 35 1 0.7083 0.0656 0.5907 0.849
 51 34 1 0.6875 0.0669 0.5681 0.832
 55 33 6 0.5625 0.0716 0.4383 0.722
 56 27 1 0.5417 0.0719 0.4176 0.703
 57 26 3 0.4792 0.0721 0.3568 0.644
 59 23 2 0.4375 0.0716 0.3174 0.603
 60 21 1 0.4167 0.0712 0.2981 0.582
 61 20 2 0.3750 0.0699 0.2603 0.540
 62 18 4 0.2917 0.0656 0.1877 0.453
 64 14 1 0.2708 0.0641 0.1703 0.431
 67 13 3 0.2083 0.0586 0.1200 0.362
 68 10 5 0.1042 0.0441 0.0454 0.239
 69 5 1 0.0833 0.0399 0.0326 0.213
 70 4 1 0.0625 0.0349 0.0209 0.187
 71 3 2 0.0208 0.0206 0.0030 0.145
 72 1 1 0.0000 NaN NA NA

 Treatment=OrcoWingClip
 time n.risk n.event survival std.err lower 95% CI upper 95% CI
 10 48 1 0.9792 0.0206 0.9396 1.000
 15 47 1 0.9583 0.0288 0.9034 1.000
 16 46 1 0.9375 0.0349 0.8715 1.000
 17 45 1 0.9167 0.0399 0.8417 0.998
 23 44 1 0.8958 0.0441 0.8135 0.987
 25 43 1 0.8750 0.0477 0.7863 0.974
 26 42 2 0.8333 0.0538 0.7343 0.946
 27 40 1 0.8125 0.0563 0.7093 0.931
 28 39 1 0.7917 0.0586 0.6847 0.915
 33 38 1 0.7708 0.0607 0.6606 0.899
 34 37 2 0.7292 0.0641 0.6137 0.866
 35 35 1 0.7083 0.0656 0.5907 0.849
 39 34 2 0.6667 0.0680 0.5458 0.814
 42 32 1 0.6458 0.0690 0.5238 0.796
 44 31 2 0.6042 0.0706 0.4805 0.760
 46 29 2 0.5625 0.0716 0.4383 0.722
 48 27 1 0.5417 0.0719 0.4176 0.703
 52 26 1 0.5208 0.0721 0.3971 0.683
 55 25 2 0.4792 0.0721 0.3568 0.644
 56 23 1 0.4583 0.0719 0.3370 0.623
 57 22 5 0.3542 0.0690 0.2417 0.519
 58 17 1 0.3333 0.0680 0.2234 0.497
 59 16 1 0.3125 0.0669 0.2054 0.475
 60 15 3 0.2500 0.0625 0.1532 0.408
 61 12 2 0.2083 0.0586 0.1200 0.362
 62 10 2 0.1667 0.0538 0.0885 0.314
 63 8 1 0.1458 0.0509 0.0735 0.289
 67 7 1 0.1250 0.0477 0.0591 0.264
 68 6 1 0.1042 0.0441 0.0454 0.239
 69 5 3 0.0417 0.0288 0.0107 0.162
 70 2 1 0.0208 0.0206 0.0030 0.145
 86 1 1 0.0000 NaN NA NA

 Treatment=OrcoWingRemoval
 time n.risk n.event survival std.err lower 95% CI upper 95% CI
 10 47 3 0.9362 0.0357 0.86883 1.000
 11 44 1 0.9149 0.0407 0.83850 0.998
 12 43 1 0.8936 0.0450 0.80968 0.986
 13 42 1 0.8723 0.0487 0.78197 0.973
 16 41 1 0.8511 0.0519 0.75513 0.959
 23 40 1 0.8298 0.0548 0.72901 0.944
 26 39 1 0.8085 0.0574 0.70350 0.929
 28 38 1 0.7872 0.0597 0.67851 0.913
 30 37 1 0.7660 0.0618 0.65399 0.897
 31 36 1 0.7447 0.0636 0.62990 0.880
 37 35 1 0.7234 0.0652 0.60619 0.863
 38 34 2 0.6809 0.0680 0.55982 0.828
 39 32 2 0.6383 0.0701 0.51471 0.792
 40 30 1 0.6170 0.0709 0.49259 0.773
 43 29 1 0.5957 0.0716 0.47074 0.754
 44 28 1 0.5745 0.0721 0.44916 0.735
 47 27 1 0.5532 0.0725 0.42785 0.715
 51 26 2 0.5106 0.0729 0.38598 0.676
 52 24 1 0.4894 0.0729 0.36543 0.655
 55 23 4 0.4043 0.0716 0.28571 0.572
 56 19 3 0.3404 0.0691 0.22866 0.507
 58 16 2 0.2979 0.0667 0.19205 0.462
 60 14 1 0.2766 0.0652 0.17420 0.439
 61 13 2 0.2340 0.0618 0.13953 0.393
 62 11 1 0.2128 0.0597 0.12277 0.369
 63 10 1 0.1915 0.0574 0.10642 0.345
 64 9 3 0.1277 0.0487 0.06046 0.270
 67 6 1 0.1064 0.0450 0.04645 0.244
 69 5 1 0.0851 0.0407 0.03333 0.217
 70 4 3 0.0213 0.0210 0.00306 0.148
 71 1 1 0.0000 NaN NA NA

 Treatment=WTSingle
 time n.risk n.event survival std.err lower 95% CI upper 95% CI
 10 44 3 0.9318 0.0380 0.86024 1.000
 17 41 2 0.8864 0.0478 0.79738 0.985
 25 39 1 0.8636 0.0517 0.76796 0.971
 26 38 1 0.8409 0.0551 0.73949 0.956
 32 37 1 0.8182 0.0581 0.71180 0.940
 34 36 1 0.7955 0.0608 0.68477 0.924
 47 35 1 0.7727 0.0632 0.65831 0.907
 48 34 1 0.7500 0.0653 0.63237 0.890
 50 33 1 0.7273 0.0671 0.60690 0.872
 61 32 1 0.7045 0.0688 0.58185 0.853
 62 31 2 0.6591 0.0715 0.53291 0.815
 63 29 1 0.6364 0.0725 0.50898 0.796
 64 28 1 0.6136 0.0734 0.48539 0.776
 65 27 1 0.5909 0.0741 0.46211 0.756
 69 26 2 0.5455 0.0751 0.41650 0.714
 71 24 1 0.5227 0.0753 0.39415 0.693
 72 23 5 0.4091 0.0741 0.28681 0.584
 73 18 1 0.3864 0.0734 0.26624 0.561
 74 17 2 0.3409 0.0715 0.22605 0.514
 76 15 1 0.3182 0.0702 0.20646 0.490
 77 14 1 0.2955 0.0688 0.18721 0.466
 78 13 3 0.2273 0.0632 0.13180 0.392
 82 10 2 0.1818 0.0581 0.09715 0.340
 83 8 3 0.1136 0.0478 0.04979 0.259
 91 5 2 0.0682 0.0380 0.02287 0.203
 92 3 1 0.0455 0.0314 0.01174 0.176
 97 2 1 0.0227 0.0225 0.00327 0.158
 102 1 1 0.0000 NaN NA NA

 Treatment=WTWingClip
 time n.risk n.event survival std.err lower 95% CI upper 95% CI
 10 46 2 0.9565 0.0301 0.89937 1.000
 15 44 1 0.9348 0.0364 0.86609 1.000
 19 43 1 0.9130 0.0415 0.83514 0.998
 26 42 2 0.8696 0.0497 0.77749 0.973
 34 40 1 0.8478 0.0530 0.75013 0.958
 38 39 1 0.8261 0.0559 0.72350 0.943
 39 38 1 0.8043 0.0585 0.69750 0.928
 46 37 2 0.7609 0.0629 0.64707 0.895
 47 35 2 0.7174 0.0664 0.59839 0.860
 49 33 1 0.6957 0.0678 0.57462 0.842
 52 32 1 0.6739 0.0691 0.55119 0.824
 57 31 2 0.6304 0.0712 0.50530 0.787
 59 29 4 0.5435 0.0734 0.41702 0.708
 60 25 2 0.5000 0.0737 0.37451 0.668
 62 23 2 0.4565 0.0734 0.33306 0.626
 63 21 4 0.3696 0.0712 0.25338 0.539
 65 17 1 0.3478 0.0702 0.23416 0.517
 66 16 1 0.3261 0.0691 0.21523 0.494
 68 15 1 0.3043 0.0678 0.19662 0.471
 69 14 1 0.2826 0.0664 0.17833 0.448
 70 13 1 0.2609 0.0647 0.16039 0.424
 72 12 4 0.1739 0.0559 0.09264 0.326
 73 8 2 0.1304 0.0497 0.06185 0.275
 74 6 2 0.0870 0.0415 0.03409 0.222
 75 4 1 0.0652 0.0364 0.02184 0.195
 77 3 2 0.0217 0.0215 0.00313 0.151
 89 1 1 0.0000 NaN NA NA

 Treatment=WTWingRemoval
 time n.risk n.event survival std.err lower 95% CI upper 95% CI
 2 47 1 0.9787 0.0210 0.9383 1.000
 10 46 6 0.8511 0.0519 0.7551 0.959
 17 40 1 0.8298 0.0548 0.7290 0.944
 18 39 1 0.8085 0.0574 0.7035 0.929
 21 38 1 0.7872 0.0597 0.6785 0.913
 27 37 1 0.7660 0.0618 0.6540 0.897
 30 36 1 0.7447 0.0636 0.6299 0.880
 34 35 1 0.7234 0.0652 0.6062 0.863
 37 34 1 0.7021 0.0667 0.5828 0.846
 38 33 1 0.6809 0.0680 0.5598 0.828
 40 32 1 0.6596 0.0691 0.5371 0.810
 43 31 1 0.6383 0.0701 0.5147 0.792
 46 30 2 0.5957 0.0716 0.4707 0.754
 47 28 3 0.5319 0.0728 0.4068 0.696
 51 25 1 0.5106 0.0729 0.3860 0.676
 55 24 1 0.4894 0.0729 0.3654 0.655
 56 23 2 0.4468 0.0725 0.3251 0.614
 57 21 1 0.4255 0.0721 0.3053 0.593
 58 20 1 0.4043 0.0716 0.2857 0.572
 59 19 1 0.3830 0.0709 0.2664 0.551
 61 18 1 0.3617 0.0701 0.2474 0.529
 63 17 1 0.3404 0.0691 0.2287 0.507
 66 16 1 0.3191 0.0680 0.2102 0.485
 68 15 2 0.2766 0.0652 0.1742 0.439
 70 13 3 0.2128 0.0597 0.1228 0.369
 72 10 2 0.1702 0.0548 0.0905 0.320
 75 8 3 0.1064 0.0450 0.0465 0.244
 77 5 1 0.0851 0.0407 0.0333 0.217
 80 4 2 0.0426 0.0294 0.0110 0.165
 81 2 2 0.0000 NaN NA NA

surv_plot_orco_1 <- ggsurvplot(surv_fit_orco, data = OrcoLifespan,
 linetype = c("solid", "twodash", "dotted", "solid", "twodash", "dotted"),
 legend.labs = c("Orco kept Alone", "Orco kept with Wing Clipped Rival", "Orco kept with Wing Removed Rival", "Wildtype kept Singly", "Wildtype kept with Wing Clipped Rival", "Wildtype kept with Wing Removed Rival"),
 palette = c("gray73", "gray73", "gray73", "black", "black", "black"),
 pval = FALSE)
surv_plot_orco_1_Figure <- ggpar(surv_plot_orco_1, font.main=c(16, "bold", "black"),
 xlab = "Days Since Eclosion", ylab = "Cumulative Survival",
 legend = "right", legend.title = "",
 xlim = c (0 , 120))

Coordinate system already present. Adding new coordinate system, which will
replace the existing one.

surv_plot_orco_1_Figure


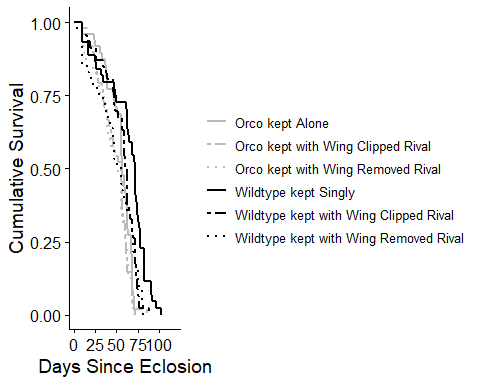


#Orco Lifespan Cox Model
fit_cox_orco <- coxph(surv_object_orco ~ Focal_Treatment * Rival_Treatment,
 data = OrcoLifespan)
#testing proportional hazards assumptions
test.ph <- cox.zph(fit_cox_orco)
test.ph #test is sig (p<0.01) - data does not have proportional hazards, will do a GLM instead

chisq df p
Focal_Treatment 9.56 1 0.0020
Rival_Treatment 2.73 2 0.2554
Focal_Treatment:Rival_Treatment 7.67 2 0.0216
GLOBAL 20.27 5 0.0011

#Orco Lifespan GLMS
#setting treatments as factors
OrcoLifespan$Focal_Treatment <- as.factor(OrcoLifespan$Focal_Treatment)
OrcoLifespan$Rival_Treatment <- as.factor(OrcoLifespan$Rival_Treatment)

OrcoLifespan_10 <- subset(OrcoLifespan, Lifespan > 10)
OrcoLifespan_Dead <- subset(OrcoLifespan_10, Censoring_Status == "1")

Orco_GLM_poisson <- glm(Lifespan ~ Focal_Treatment * Rival_Treatment, data = OrcoLifespan_Dead,
 family=poisson (link="log"))
dispersiontest(Orco_GLM_poisson) #dispersion factor is 5.24 - data is overdispersed, will use quasipoisson

Overdispersion test

data: Orco_GLM_poisson
z = 9.4442, p-value < 2.2e-16
alternative hypothesis: true dispersion is greater than 1
sample estimates:
dispersion
 5.242363

#Orco Lifespan Model Selection
Orco_GLM_1 <- glm(Lifespan ~ Focal_Treatment * Rival_Treatment, data = OrcoLifespan_Dead,
 family=quasipoisson (link="log"))
summary(Orco_GLM_1)

Call:
glm(formula = Lifespan ~ Focal_Treatment * Rival_Treatment, family = quasipoisson(link = "log"),
 data = OrcoLifespan_Dead)

Deviance Residuals:
 Min 1Q Median 3Q Max
-7.2780 -1.4408 0.5463 1.6063 4.8005

Coefficients:
 Estimate Std. Error t value
(Intercept) 4.019121 0.045780 87.792
Focal_TreatmentWT 0.181559 0.063693 2.851
Rival_TreatmentWing Clip -0.131216 0.066593 -1.970
Rival_TreatmentWing Removal -0.139903 0.067940 -2.059
Focal_TreatmentWT:Rival_TreatmentWing Clip -0.002771 0.092113 -0.030
Focal_TreatmentWT:Rival_TreatmentWing Removal -0.038108 0.094755 -0.402
 Pr(>|t|)
(Intercept) < 2e-16 ***
Focal_TreatmentWT 0.00472 **
Rival_TreatmentWing Clip 0.04987 *
Rival_TreatmentWing Removal 0.04049 *
Focal_TreatmentWT:Rival_TreatmentWing Clip 0.97603
Focal_TreatmentWT:Rival_TreatmentWing Removal 0.68789
---
Signif. codes: 0 '***' 0.001 '**' 0.01 '*' 0.05 '.' 0.1 ' ' 1

(Dispersion parameter for quasipoisson family taken to be 5.365239)

 Null deviance: 1738.3 on 261 degrees of freedom
Residual deviance: 1562.9 on 256 degrees of freedom
AIC: NA

Number of Fisher Scoring iterations: 4

Orco_GLM_2 <- glm(Lifespan ~ Focal_Treatment + Rival_Treatment, data = OrcoLifespan_Dead,
 family=quasipoisson (link="log"))
anova(Orco_GLM_1, Orco_GLM_2, test = "F")

Analysis of Deviance Table

Model 1: Lifespan ~ Focal_Treatment * Rival_Treatment
Model 2: Lifespan ~ Focal_Treatment + Rival_Treatment
 Resid. Df Resid. Dev Df Deviance F Pr(>F)
1 256 1562.9
2 258 1563.9 -2 -1.036 0.0965 0.908

Orco_GLM_3 <- glm(Lifespan ~ Rival_Treatment, data = OrcoLifespan_Dead,
 family=quasipoisson (link="log"))
anova(Orco_GLM_2, Orco_GLM_3, test = "F")

Analysis of Deviance Table

Model 1: Lifespan ~ Focal_Treatment + Rival_Treatment
Model 2: Lifespan ~ Rival_Treatment
 Resid. Df Resid. Dev Df Deviance F Pr(>F)
1 258 1563.9
2 259 1667.8 -1 -103.84 19.478 1.497e-05 ***
---
Signif. codes: 0 '***' 0.001 '**' 0.01 '*' 0.05 '.' 0.1 ' ' 1

Orco_GLM_4 <- glm(Lifespan ~ Focal_Treatment, data = OrcoLifespan_Dead,
 family=quasipoisson (link="log"))
anova(Orco_GLM_2, Orco_GLM_4, test = "F")

Analysis of Deviance Table

Model 1: Lifespan ~ Focal_Treatment + Rival_Treatment
Model 2: Lifespan ~ Focal_Treatment
 Resid. Df Resid. Dev Df Deviance F Pr(>F)
1 258 1563.9
2 260 1635.7 -2 -71.816 6.7357 0.001408 **
---
Signif. codes: 0 '***' 0.001 '**' 0.01 '*' 0.05 '.' 0.1 ' ' 1

Orco_GLM_null <- glm(Lifespan ~ 1, data=OrcoLifespan_Dead, family=quasipoisson(link ="log"))

anova(Orco_GLM_2, Orco_GLM_null, test = "F")

Analysis of Deviance Table

Model 1: Lifespan ~ Focal_Treatment + Rival_Treatment
Model 2: Lifespan ~ 1
 Resid. Df Resid. Dev Df Deviance F Pr(>F)
1 258 1563.9
2 261 1738.3 -3 -174.4 10.905 9.098e-07 ***
---
Signif. codes: 0 '***' 0.001 '**' 0.01 '*' 0.05 '.' 0.1 ' ' 1

summary(Orco_GLM_2)

Call:
glm(formula = Lifespan ~ Focal_Treatment + Rival_Treatment, family = quasipoisson(link = "log"),
 data = OrcoLifespan_Dead)

Deviance Residuals:
 Min 1Q Median 3Q Max
-7.2370 -1.4602 0.5754 1.6191 4.7613

Coefficients:
 Estimate Std. Error t value Pr(>|t|)
(Intercept) 4.02550 0.03734 107.799 < 2e-16 ***
Focal_TreatmentWT 0.16918 0.03835 4.411 1.51e-05 ***
Rival_TreatmentWing Clip -0.13253 0.04586 -2.890 0.004183 **
Rival_TreatmentWing Removal -0.15954 0.04721 -3.379 0.000839 ***
---
Signif. codes: 0 '***' 0.001 '**' 0.01 '*' 0.05 '.' 0.1 ' ' 1

(Dispersion parameter for quasipoisson family taken to be 5.33102)

 Null deviance: 1738.3 on 261 degrees of freedom
Residual deviance: 1563.9 on 258 degrees of freedom
AIC: NA

Number of Fisher Scoring iterations: 4

#model is significant p<2e-16***

#post-hoc tests
lsmeans(Orco_GLM_2, pairwise ~ Focal_Treatment + Rival_Treatment, adjust="tukey")

$lsmeans
 Focal_Treatment Rival_Treatment lsmean SE df asymp.LCL asymp.UCL
 Orco Single 4.03 0.0373 Inf 3.95 4.10
 WT Single 4.19 0.0368 Inf 4.12 4.27
 Orco Wing Clip 3.89 0.0388 Inf 3.82 3.97
 WT Wing Clip 4.06 0.0378 Inf 3.99 4.14
 Orco Wing Removal 3.87 0.0402 Inf 3.79 3.94
 WT Wing Removal 4.04 0.0395 Inf 3.96 4.11

Results are given on the log (not the response) scale.
Confidence level used: 0.95

$contrasts
 contrast estimate SE df z.ratio p.value
 Orco Single - WT Single -0.16918 0.0384 Inf -4.411 0.0001
 Orco Single - Orco Wing Clip 0.13253 0.0459 Inf 2.890 0.0446
 Orco Single - WT Wing Clip -0.03665 0.0595 Inf -0.616 0.9899
 Orco Single - Orco Wing Removal 0.15954 0.0472 Inf 3.379 0.0095
 Orco Single - WT Wing Removal -0.00964 0.0607 Inf -0.159 1.0000
 WT Single - Orco Wing Clip 0.30170 0.0601 Inf 5.021 <.0001
 WT Single - WT Wing Clip 0.13253 0.0459 Inf 2.890 0.0446
 WT Single - Orco Wing Removal 0.32871 0.0609 Inf 5.394 <.0001
 WT Single - WT Wing Removal 0.15954 0.0472 Inf 3.379 0.0095
 Orco Wing Clip - WT Wing Clip -0.16918 0.0384 Inf -4.411 0.0001
 Orco Wing Clip - Orco Wing Removal 0.02701 0.0481 Inf 0.561 0.9935
 Orco Wing Clip - WT Wing Removal -0.14217 0.0617 Inf -2.303 0.1927
 WT Wing Clip - Orco Wing Removal 0.19618 0.0614 Inf 3.196 0.0175
 WT Wing Clip - WT Wing Removal 0.02701 0.0481 Inf 0.561 0.9935
 Orco Wing Removal - WT Wing Removal -0.16918 0.0384 Inf -4.411 0.0001

Results are given on the log (not the response) scale.
P value adjustment: tukey method for comparing a family of 6 estimates

#boxplot
OrcoLifespan_10$Treatment <- gsub(" ", "", OrcoLifespan_10$Treatment)
OrcoLifespan_10$Rival_Treatment <- gsub(" ", "", OrcoLifespan_10$Rival_Treatment)

OrcoLifespan_10$Focal_Treatment <- factor(OrcoLifespan_10$Focal_Treatment, levels = c("WT", "Orco"))
OrcoLifespan_10$Rival_Treatment <- factor(OrcoLifespan_10$Rival_Treatment, levels = c("Single", "WingClip", "WingRemoval"))
OrcoLifespan_10$Treatment <- factor(OrcoLifespan_10$Treatment, levels = c("WTSingle", "WTWingClip", "WTWingRemoval", "OrcoSingle", "OrcoWingClip", "OrcoWingRemoval"))

orco_lifespan_sample_sizes <- OrcoLifespan_10 %>%
 group_by(Focal_Treatment, Rival_Treatment, Treatment) %>%
 dplyr::summarise(
 Count = n(),
 Mean_lifespan = mean(Lifespan)
 )

`summarise()` has grouped output by 'Focal_Treatment', 'Rival_Treatment'. You
can override using the `.groups` argument.

orco_lifespan_sample_sizes$Count <- as.character(orco_lifespan_sample_sizes$Count)

boxplot_orco_lifespan_bw <- ggplot(OrcoLifespan_10) +
 aes(
 x = Focal_Treatment,
 y = Lifespan,
 fill = Rival_Treatment
 ) +
 geom_boxplot(size = 5) +
 scale_fill_manual(
 values = c(Single = "white",
 WingClip = "grey70",
 WingRemoval = "grey25"),
 labels=c("Single" = "Single",
 "WingClip" = "Wing Clipped",
 "WingRemoval" = "Wing Removed")
 ) +
 labs(
 x = "Focal Treatment",
 y = "Days since Eclosion ",
 fill = "Social Treatment"
 ) +
 ylim(-2, 140) +
 geom_signif(y_position = c(115, 130, 115, 130),
 xmin = c(0.75, 0.75, 1.75, 1.75),
 xmax = c(1, 1.25, 2, 2.25), annotation = c("*", "**", "*", "**"),
 tip_length = 0, textsize = 25, size = 5)

boxplot_orco_lifespan_bw +
 theme_bw() + theme(panel.grid.major = element_blank(), panel.grid.minor = element_blank()) +
 theme(axis.text=element_text(size=55, face="bold", colour="black"),
 axis.title=element_text(size=75, face="bold", colour="black"),
 legend.text=element_text(size=55, face="bold", colour="black"),
 legend.title=element_text(size=75, face="bold", colour="black")) +
 theme(axis.line = element_line(size = 5),
 panel.border = element_rect(size = 5)) +
 theme(
 axis.ticks.length = unit(0.5, "cm"),
 axis.ticks = element_line(size = 5) ) +
 scale_x_discrete(labels=c("WT" = "Wildtype", "Orco" = "Orco2")) +
 geom_text(
 data = orco_lifespan_sample_sizes,
 aes(
 x = Focal_Treatment,
 y = -1,
 label = paste0("n=", Count)
 ),
 position = position_dodge(width = 0.75),
 vjust = -0.5,
 size = 12)


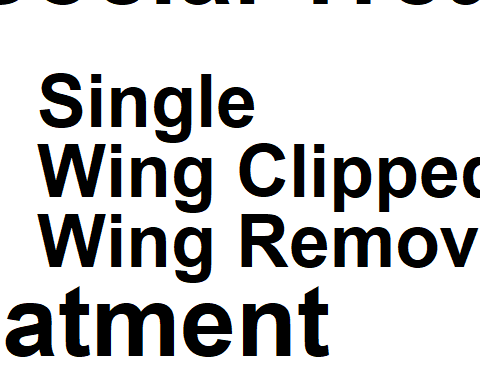


Orco_group_stats_lifespan <- OrcoLifespan_Dead %>%
 group_by(Focal_Treatment, Rival_Treatment) %>%
 dplyr::summarise(mean = mean(Lifespan),
 sd = sd(Lifespan),
 N = n())

`summarise()` has grouped output by 'Focal_Treatment'. You can override using
the `.groups` argument.

OrcoExp_WT_Single <- OrcoLifespan_Dead %>%
 filter(Rival_Treatment == "Single", Focal_Treatment == "WT") %>%
 select(Lifespan)
OrcoExp_WT_WC <- OrcoLifespan_Dead %>%
 filter(Rival_Treatment == "Wing Clip", Focal_Treatment == "WT") %>%
 select(Lifespan)
OrcoExp_WT_WR <- OrcoLifespan_Dead %>%
 filter(Rival_Treatment == "Wing Removal", Focal_Treatment == "WT") %>%
 select(Lifespan)
cohen.d(OrcoExp_WT_Single$Lifespan, OrcoExp_WT_WC$Lifespan)

Cohen's d

d estimate: 0.4430705 (small)
95 percent confidence interval:
 lower upper
0.006077031 0.880063981

cohen.d(OrcoExp_WT_Single$Lifespan, OrcoExp_WT_WR$Lifespan)

Cohen's d

d estimate: 0.5480967 (medium)
95 percent confidence interval:
 lower upper
0.09751303 0.99868045

cohen.d(OrcoExp_WT_WR$Lifespan, OrcoExp_WT_WC$Lifespan)

Cohen's d

d estimate: -0.1439325 (negligible)
95 percent confidence interval:
 lower upper
-0.5790915 0.2912265

OrcoExp_Orco_Single <- OrcoLifespan_Dead %>%
 filter(Rival_Treatment == "Single", Focal_Treatment == "Orco") %>%
 select(Lifespan)
OrcoExp_Orco_WC <- OrcoLifespan_Dead %>%
 filter(Rival_Treatment == "Wing Clip", Focal_Treatment == "Orco") %>%
 select(Lifespan)
OrcoExp_Orco_WR <- OrcoLifespan_Dead %>%
 filter(Rival_Treatment == "Wing Removal", Focal_Treatment == "Orco") %>%
 select(Lifespan)
cohen.d(OrcoExp_Orco_Single$Lifespan, OrcoExp_Orco_WC$Lifespan)

Cohen's d

d estimate: 0.4541561 (small)
95 percent confidence interval:
 lower upper
0.0369004 0.8714118

cohen.d(OrcoExp_Orco_Single$Lifespan, OrcoExp_Orco_WR$Lifespan)

Cohen's d

d estimate: 0.4804618 (small)
95 percent confidence interval:
 lower upper
0.05540069 0.90552295

cohen.d(OrcoExp_Orco_WR$Lifespan, OrcoExp_Orco_WC$Lifespan)

Cohen's d

d estimate: -0.02473299 (negligible)
95 percent confidence interval:
 lower upper
-0.4415594 0.3920934

## Lifelong Behaviour

**Supplementary Figure 3 & 4**

**Description of Data:** Behavioural scans of social treatment and senses of WT and Orco2 flies when kept solo versus when they were with a rival having a clipped wing or a completely removed wing.

**Factors:**

- FlyID-Unique fly ID
- Focal_Treatment-Focal fly genotype. (WT=not manipulated, Orco=Orco2 olfactory mutant)
- Rival_Treatment-Social Treatment. Single=focal fly kept in isolation post eclosion, WingClip=fly kept with single rival with a wing clip from eclosion to mating, WingRemoved=fly kept with a rival with wings surgical removed
- Week-Week of observation (weeks since eclosion)
- Observation-Behaviour observed of focal fly (W=walking, F=inactive on food, I=inactive, A=aggressive behaviour, G=grooming [not analysed], NA = no recorded behaviour)
- Paired-Interactive behaviour of focal fly observed. (WBL = within one body length, 1=wing flicks, 2=chasing, 3=boxing)
- n-Number of observations within a ten minute period (one scan per minute)

###loading packages
library(tidyverse)
library(dplyr)
library(ggplot2)
library(lme4)
library(emmeans)
library(glmmTMB)
library(mgcv)

Loading required package: nlme

Attaching package: 'nlme'

The following object is masked from 'package:dplyr':

 collapse

The following object is masked from 'package:lme4':

 lmList

This is mgcv 1.8-42. For overview type 'help("mgcv-package")'.

library(ggstance)

Attaching package: 'ggstance'

The following objects are masked from 'package:ggplot2':

 geom_errorbarh, GeomErrorbarh

library(ggpubr)
library(effsize)
library(Rmisc)

Loading required package: lattice

Loading required package: plyr

------------------------------------------------------------------------------

You have loaded plyr after dplyr - this is likely to cause problems.
If you need functions from both plyr and dplyr, please load plyr first, then dplyr:
library(plyr); library(dplyr)

------------------------------------------------------------------------------

Attaching package: 'plyr'

The following object is masked from 'package:ggpubr':

 mutate

The following objects are masked from 'package:dplyr':

 arrange, count, desc, failwith, id, mutate, rename, summarise,
 summarize

The following object is masked from 'package:purrr':

 compact

###required data
OrcoBehaviour_all <- read.csv("OrcoBehaviour_all.csv")
OrcoBehaviour_all <- OrcoBehaviour_all[!is.na(OrcoBehaviour_all$Week), ]
OrcoGroup_all <- read.csv("OrcoBehaviour_paired.csv")
OrcoGroup_all <- OrcoGroup_all[!is.na(OrcoGroup_all$Week), ]


###Success & Failures
OrcoBehaviour_all$Success <- OrcoBehaviour_all$n
OrcoBehaviour_all$Failure <- 10 - OrcoBehaviour_all$n
OrcoGroup_all$Success <- OrcoGroup_all$n
OrcoGroup_all$Failure <- 10 - OrcoGroup_all$n

###Subsetting Data
Orco_Food <- subset(OrcoBehaviour_all, Observation == "F")
Orco_Walking <- subset(OrcoBehaviour_all, Observation == "W")
Orco_Grooming <- subset(OrcoBehaviour_all, Observation == "G")
Orco_Inactive <- subset(OrcoBehaviour_all, Observation == "I")
Orco_Aggression <- subset(OrcoBehaviour_all, Observation == "A")
Orco_WBL <- subset(OrcoGroup_all, Paired == "WBL")


###Orco Food
##Orco Food Model
Orco_Food$Week <- as.factor(Orco_Food$Week)
OrcoFeeding_glmmTMB1 <- glmmTMB(Success ~ Focal_Treatment * Rival_Treatment + Week + (1|FlyID), family=poisson(link = "log"), data = Orco_Food)
OrcoFeeding_glmmTMB2 <- glmmTMB(Success ~ Focal_Treatment + Rival_Treatment + Week + (1|FlyID), family=poisson(link = "log"), data = Orco_Food)
anova(OrcoFeeding_glmmTMB1, OrcoFeeding_glmmTMB2, test="Chi")

Data: Orco_Food
Models:
OrcoFeeding_glmmTMB2: Success ~ Focal_Treatment + Rival_Treatment + Week + (1 | FlyID), zi=~0, disp=~1
OrcoFeeding_glmmTMB1: Success ~ Focal_Treatment * Rival_Treatment + Week + (1 | FlyID), zi=~0, disp=~1
 Df AIC BIC logLik deviance Chisq Chi Df Pr(>Chisq)
OrcoFeeding_glmmTMB2 9 5653.3 5700.0 -2817.7 5635.3
OrcoFeeding_glmmTMB1 11 5653.3 5710.3 -2815.6 5631.3 4.0558 2 0.1316

OrcoFeeding_glmmTMB3 <- glmmTMB(Success ~ Rival_Treatment + Week + (1|FlyID), family=poisson(link = "log"), data = Orco_Food)
anova(OrcoFeeding_glmmTMB2, OrcoFeeding_glmmTMB3, test="Chi")

Data: Orco_Food
Models:
OrcoFeeding_glmmTMB3: Success ~ Rival_Treatment + Week + (1 | FlyID), zi=~0, disp=~1
OrcoFeeding_glmmTMB2: Success ~ Focal_Treatment + Rival_Treatment + Week + (1 | FlyID), zi=~0, disp=~1
 Df AIC BIC logLik deviance Chisq Chi Df Pr(>Chisq)
OrcoFeeding_glmmTMB3 8 5665.5 5707 -2824.8 5649.5
OrcoFeeding_glmmTMB2 9 5653.3 5700 -2817.7 5635.3 14.164 1 0.0001676

OrcoFeeding_glmmTMB3
OrcoFeeding_glmmTMB2 ***
---
Signif. codes: 0 '***' 0.001 '**' 0.01 '*' 0.05 '.' 0.1 ' ' 1

OrcoFeeding_glmmTMB4 <- glmmTMB(Success ~ Focal_Treatment + Week + (1|FlyID), family=poisson(link = "log"), data = Orco_Food)
anova(OrcoFeeding_glmmTMB2, OrcoFeeding_glmmTMB4, test="Chi")

Data: Orco_Food
Models:
OrcoFeeding_glmmTMB4: Success ~ Focal_Treatment + Week + (1 | FlyID), zi=~0, disp=~1
OrcoFeeding_glmmTMB2: Success ~ Focal_Treatment + Rival_Treatment + Week + (1 | FlyID), zi=~0, disp=~1
 Df AIC BIC logLik deviance Chisq Chi Df Pr(>Chisq)
OrcoFeeding_glmmTMB4 7 5723.6 5759.9 -2854.8 5709.6
OrcoFeeding_glmmTMB2 9 5653.3 5700.0 -2817.7 5635.3 74.301 2 < 2.2e-16

OrcoFeeding_glmmTMB4
OrcoFeeding_glmmTMB2 ***
---
Signif. codes: 0 '***' 0.001 '**' 0.01 '*' 0.05 '.' 0.1 ' ' 1

OrcoFeeding_glmmTMB5 <- glmmTMB(Success ~ Focal_Treatment + Rival_Treatment + (1|FlyID), family=poisson(link = "log"), data = Orco_Food)
anova(OrcoFeeding_glmmTMB2, OrcoFeeding_glmmTMB5, test="Chi")

Data: Orco_Food
Models:
OrcoFeeding_glmmTMB5: Success ~ Focal_Treatment + Rival_Treatment + (1 | FlyID), zi=~0, disp=~1
OrcoFeeding_glmmTMB2: Success ~ Focal_Treatment + Rival_Treatment + Week + (1 | FlyID), zi=~0, disp=~1
 Df AIC BIC logLik deviance Chisq Chi Df Pr(>Chisq)
OrcoFeeding_glmmTMB5 5 5773.8 5799.7 -2881.9 5763.8
OrcoFeeding_glmmTMB2 9 5653.3 5700.0 -2817.7 5635.3 128.48 4 < 2.2e-16

OrcoFeeding_glmmTMB5
OrcoFeeding_glmmTMB2 ***
---
Signif. codes: 0 '***' 0.001 '**' 0.01 '*' 0.05 '.' 0.1 ' ' 1

OrcoFeeding_glmmTMB_null <- glmmTMB(Success ~ (1|FlyID), family=poisson(link = "log"), data = Orco_Food)
anova(OrcoFeeding_glmmTMB1, OrcoFeeding_glmmTMB_null, test="Chi")

Data: Orco_Food
Models:
OrcoFeeding_glmmTMB_null: Success ~ (1 | FlyID), zi=~0, disp=~1
OrcoFeeding_glmmTMB1: Success ~ Focal_Treatment * Rival_Treatment + Week + (1 | FlyID), zi=~0, disp=~1
 Df AIC BIC logLik deviance Chisq Chi Df
OrcoFeeding_glmmTMB_null 2 5854.4 5864.8 -2925.2 5850.4
OrcoFeeding_glmmTMB1 11 5653.3 5710.3 -2815.6 5631.3 219.17 9
 Pr(>Chisq)
OrcoFeeding_glmmTMB_null
OrcoFeeding_glmmTMB1 < 2.2e-16 ***
---
Signif. codes: 0 '***' 0.001 '**' 0.01 '*' 0.05 '.' 0.1 ' ' 1

summary(OrcoFeeding_glmmTMB1)

Family: poisson ( log )
Formula:
Success ~ Focal_Treatment * Rival_Treatment + Week + (1 | FlyID)
Data: Orco_Food

 AIC BIC logLik deviance df.resid
 5653.3 5710.3 -2815.6 5631.3 1308

Random effects:

Conditional model:
 Groups Name Variance Std.Dev.
 FlyID (Intercept) 0.7367 0.8583
Number of obs: 1319, groups: FlyID, 300

Conditional model:
 Estimate Std. Error z value
(Intercept) -0.35949 0.15922 -2.258
Focal_TreatmentWT 0.40070 0.21110 1.898
Rival_TreatmentWingClip 1.06211 0.20446 5.195
Rival_TreatmentWingRemoved 1.09597 0.20437 5.363
Week2 -0.39421 0.05725 -6.886
Week3 -0.45362 0.05977 -7.589
Week4 -0.37610 0.06019 -6.249
Week5 0.04684 0.05512 0.850
Focal_TreatmentWT:Rival_TreatmentWingClip 0.29762 0.28076 1.060
Focal_TreatmentWT:Rival_TreatmentWingRemoved -0.24078 0.28385 -0.848
 Pr(>|z|)
(Intercept) 0.0240 *
Focal_TreatmentWT 0.0577 .
Rival_TreatmentWingClip 2.05e-07 ***
Rival_TreatmentWingRemoved 8.19e-08 ***
Week2 5.75e-12 ***
Week3 3.22e-14 ***
Week4 4.14e-10 ***
Week5 0.3955
Focal_TreatmentWT:Rival_TreatmentWingClip 0.2891
Focal_TreatmentWT:Rival_TreatmentWingRemoved 0.3963
---
Signif. codes: 0 '***' 0.001 '**' 0.01 '*' 0.05 '.' 0.1 ' ' 1

lsmeans(OrcoFeeding_glmmTMB2, pairwise ~ Focal_Treatment + Rival_Treatment * Week, adjust="tukey")

$lsmeans
 Focal_Treatment Rival_Treatment Week lsmean SE df asymp.LCL asymp.UCL
 Orco Single 1 -0.3806 0.130 Inf -0.6346 -0.1265
 WT Single 1 0.0485 0.124 Inf -0.1953 0.2923
 Orco WingClip 1 0.8422 0.116 Inf 0.6143 1.0700
 WT WingClip 1 1.2712 0.112 Inf 1.0514 1.4910
 Orco WingRemoved 1 0.5955 0.119 Inf 0.3632 0.8278
 WT WingRemoved 1 1.0246 0.115 Inf 0.7985 1.2507
 Orco Single 2 -0.7746 0.132 Inf -1.0338 -0.5154
 WT Single 2 -0.3455 0.127 Inf -0.5953 -0.0957
 Orco WingClip 2 0.4481 0.119 Inf 0.2145 0.6818
 WT WingClip 2 0.8772 0.116 Inf 0.6506 1.1039
 Orco WingRemoved 2 0.2015 0.122 Inf -0.0368 0.4398
 WT WingRemoved 2 0.6306 0.119 Inf 0.3975 0.8636
 Orco Single 3 -0.8341 0.133 Inf -1.0951 -0.5731
 WT Single 3 -0.4050 0.129 Inf -0.6570 -0.1531
 Orco WingClip 3 0.3886 0.120 Inf 0.1528 0.6244
 WT WingClip 3 0.8177 0.117 Inf 0.5885 1.0468
 Orco WingRemoved 3 0.1420 0.123 Inf -0.0984 0.3823
 WT WingRemoved 3 0.5710 0.120 Inf 0.3357 0.8064
 Orco Single 4 -0.7563 0.133 Inf -1.0177 -0.4949
 WT Single 4 -0.3272 0.129 Inf -0.5792 -0.0752
 Orco WingClip 4 0.4664 0.121 Inf 0.2301 0.7028
 WT WingClip 4 0.8955 0.117 Inf 0.6661 1.1248
 Orco WingRemoved 4 0.2198 0.123 Inf -0.0210 0.4606
 WT WingRemoved 4 0.6488 0.120 Inf 0.4134 0.8843
 Orco Single 5 -0.3329 0.131 Inf -0.5897 -0.0761
 WT Single 5 0.0962 0.126 Inf -0.1510 0.3434
 Orco WingClip 5 0.8898 0.118 Inf 0.6582 1.1215
 WT WingClip 5 1.3189 0.114 Inf 1.0945 1.5433
 Orco WingRemoved 5 0.6432 0.120 Inf 0.4071 0.8793
 WT WingRemoved 5 1.0723 0.118 Inf 0.8418 1.3028

Results are given on the log (not the response) scale.
Confidence level used: 0.95

$contrasts
 contrast estimate SE df z.ratio
 Orco Single Week1 - WT Single Week1 -0.42907 0.1145 Inf -3.747
 Orco Single Week1 - Orco WingClip Week1 -1.22272 0.1428 Inf -8.564
 Orco Single Week1 - WT WingClip Week1 -1.65179 0.1841 Inf -8.972
 Orco Single Week1 - Orco WingRemoved Week1 -0.97607 0.1439 Inf -6.784
 Orco Single Week1 - WT WingRemoved Week1 -1.40514 0.1855 Inf -7.576
 Orco Single Week1 - Orco Single Week2 0.39401 0.0573 Inf 6.881
 Orco Single Week1 - WT Single Week2 -0.03506 0.1284 Inf -0.273
 Orco Single Week1 - Orco WingClip Week2 -0.82871 0.1539 Inf -5.386
 Orco Single Week1 - WT WingClip Week2 -1.25778 0.1931 Inf -6.514
 Orco Single Week1 - Orco WingRemoved Week2 -0.58206 0.1550 Inf -3.755
 Orco Single Week1 - WT WingRemoved Week2 -1.01113 0.1945 Inf -5.199
 Orco Single Week1 - Orco Single Week3 0.45353 0.0598 Inf 7.586
 Orco Single Week1 - WT Single Week3 0.02446 0.1297 Inf 0.189
 Orco Single Week1 - Orco WingClip Week3 -0.76918 0.1549 Inf -4.966
 Orco Single Week1 - WT WingClip Week3 -1.19825 0.1940 Inf -6.177
 Orco Single Week1 - Orco WingRemoved Week3 -0.52254 0.1560 Inf -3.350
 Orco Single Week1 - WT WingRemoved Week3 -0.95161 0.1954 Inf -4.871
 Orco Single Week1 - Orco Single Week4 0.37572 0.0602 Inf 6.241
 Orco Single Week1 - WT Single Week4 -0.05334 0.1297 Inf -0.411
 Orco Single Week1 - Orco WingClip Week4 -0.84699 0.1551 Inf -5.461
 Orco Single Week1 - WT WingClip Week4 -1.27606 0.1941 Inf -6.576
 Orco Single Week1 - Orco WingRemoved Week4 -0.60035 0.1562 Inf -3.844
 Orco Single Week1 - WT WingRemoved Week4 -1.02941 0.1954 Inf -5.269
 Orco Single Week1 - Orco Single Week5 -0.04769 0.0551 Inf -0.865
 Orco Single Week1 - WT Single Week5 -0.47676 0.1274 Inf -3.743
 Orco Single Week1 - Orco WingClip Week5 -1.27041 0.1533 Inf -8.285
 Orco Single Week1 - WT WingClip Week5 -1.69948 0.1926 Inf -8.823
 Orco Single Week1 - Orco WingRemoved Week5 -1.02376 0.1544 Inf -6.632
 Orco Single Week1 - WT WingRemoved Week5 -1.45283 0.1939 Inf -7.492
 WT Single Week1 - Orco WingClip Week1 -0.79365 0.1819 Inf -4.363
 WT Single Week1 - WT WingClip Week1 -1.22272 0.1428 Inf -8.564
 WT Single Week1 - Orco WingRemoved Week1 -0.54700 0.1823 Inf -3.001
 WT Single Week1 - WT WingRemoved Week1 -0.97607 0.1439 Inf -6.784
 WT Single Week1 - Orco Single Week2 0.82308 0.1277 Inf 6.448
 WT Single Week1 - WT Single Week2 0.39401 0.0573 Inf 6.881
 WT Single Week1 - Orco WingClip Week2 -0.39964 0.1905 Inf -2.098
 WT Single Week1 - WT WingClip Week2 -0.82871 0.1539 Inf -5.386
 WT Single Week1 - Orco WingRemoved Week2 -0.15299 0.1909 Inf -0.801
 WT Single Week1 - WT WingRemoved Week2 -0.58206 0.1550 Inf -3.755
 WT Single Week1 - Orco Single Week3 0.88260 0.1287 Inf 6.858
 WT Single Week1 - WT Single Week3 0.45353 0.0598 Inf 7.586
 WT Single Week1 - Orco WingClip Week3 -0.34012 0.1913 Inf -1.778
 WT Single Week1 - WT WingClip Week3 -0.76918 0.1549 Inf -4.966
 WT Single Week1 - Orco WingRemoved Week3 -0.09347 0.1917 Inf -0.488
 WT Single Week1 - WT WingRemoved Week3 -0.52254 0.1560 Inf -3.350
 WT Single Week1 - Orco Single Week4 0.80479 0.1290 Inf 6.237
 WT Single Week1 - WT Single Week4 0.37572 0.0602 Inf 6.241
 WT Single Week1 - Orco WingClip Week4 -0.41792 0.1915 Inf -2.182
 WT Single Week1 - WT WingClip Week4 -0.84699 0.1551 Inf -5.461
 WT Single Week1 - Orco WingRemoved Week4 -0.17128 0.1919 Inf -0.893
 WT Single Week1 - WT WingRemoved Week4 -0.60035 0.1562 Inf -3.844
 WT Single Week1 - Orco Single Week5 0.38137 0.1268 Inf 3.007
 WT Single Week1 - WT Single Week5 -0.04769 0.0551 Inf -0.865
 WT Single Week1 - Orco WingClip Week5 -0.84134 0.1901 Inf -4.425
 WT Single Week1 - WT WingClip Week5 -1.27041 0.1533 Inf -8.285
 WT Single Week1 - Orco WingRemoved Week5 -0.59469 0.1905 Inf -3.122
 WT Single Week1 - WT WingRemoved Week5 -1.02376 0.1544 Inf -6.632
 Orco WingClip Week1 - WT WingClip Week1 -0.42907 0.1145 Inf -3.747
 Orco WingClip Week1 - Orco WingRemoved Week1 0.24665 0.1348 Inf 1.830
 Orco WingClip Week1 - WT WingRemoved Week1 -0.18242 0.1774 Inf -1.028
 Orco WingClip Week1 - Orco Single Week2 1.61673 0.1538 Inf 10.514
 Orco WingClip Week1 - WT Single Week2 1.18766 0.1909 Inf 6.221
 Orco WingClip Week1 - Orco WingClip Week2 0.39401 0.0573 Inf 6.881
 Orco WingClip Week1 - WT WingClip Week2 -0.03506 0.1284 Inf -0.273
 Orco WingClip Week1 - Orco WingRemoved Week2 0.64066 0.1466 Inf 4.371
 Orco WingClip Week1 - WT WingRemoved Week2 0.21159 0.1867 Inf 1.133
 Orco WingClip Week1 - Orco Single Week3 1.67625 0.1547 Inf 10.839
 Orco WingClip Week1 - WT Single Week3 1.24718 0.1917 Inf 6.506
 Orco WingClip Week1 - Orco WingClip Week3 0.45353 0.0598 Inf 7.586
 Orco WingClip Week1 - WT WingClip Week3 0.02446 0.1297 Inf 0.189
 Orco WingClip Week1 - Orco WingRemoved Week3 0.70018 0.1475 Inf 4.746
 Orco WingClip Week1 - WT WingRemoved Week3 0.27111 0.1876 Inf 1.445
 Orco WingClip Week1 - Orco Single Week4 1.59844 0.1548 Inf 10.328
 Orco WingClip Week1 - WT Single Week4 1.16937 0.1917 Inf 6.100
 Orco WingClip Week1 - Orco WingClip Week4 0.37572 0.0602 Inf 6.241
 Orco WingClip Week1 - WT WingClip Week4 -0.05334 0.1297 Inf -0.411
 Orco WingClip Week1 - Orco WingRemoved Week4 0.62237 0.1477 Inf 4.215
 Orco WingClip Week1 - WT WingRemoved Week4 0.19330 0.1876 Inf 1.031
 Orco WingClip Week1 - Orco Single Week5 1.17502 0.1527 Inf 7.693
 Orco WingClip Week1 - WT Single Week5 0.74595 0.1900 Inf 3.926
 Orco WingClip Week1 - Orco WingClip Week5 -0.04769 0.0551 Inf -0.865
 Orco WingClip Week1 - WT WingClip Week5 -0.47676 0.1274 Inf -3.743
 Orco WingClip Week1 - Orco WingRemoved Week5 0.19895 0.1456 Inf 1.366
 Orco WingClip Week1 - WT WingRemoved Week5 -0.23011 0.1859 Inf -1.238
 WT WingClip Week1 - Orco WingRemoved Week1 0.67572 0.1763 Inf 3.832
 WT WingClip Week1 - WT WingRemoved Week1 0.24665 0.1348 Inf 1.830
 WT WingClip Week1 - Orco Single Week2 2.04580 0.1925 Inf 10.626
 WT WingClip Week1 - WT Single Week2 1.61673 0.1538 Inf 10.514
 WT WingClip Week1 - Orco WingClip Week2 0.82308 0.1277 Inf 6.448
 WT WingClip Week1 - WT WingClip Week2 0.39401 0.0573 Inf 6.881
 WT WingClip Week1 - Orco WingRemoved Week2 1.06973 0.1852 Inf 5.775
 WT WingClip Week1 - WT WingRemoved Week2 0.64066 0.1466 Inf 4.371
 WT WingClip Week1 - Orco Single Week3 2.10532 0.1932 Inf 10.900
 WT WingClip Week1 - WT Single Week3 1.67625 0.1547 Inf 10.839
 WT WingClip Week1 - Orco WingClip Week3 0.88260 0.1287 Inf 6.858
 WT WingClip Week1 - WT WingClip Week3 0.45353 0.0598 Inf 7.586
 WT WingClip Week1 - Orco WingRemoved Week3 1.12925 0.1859 Inf 6.074
 WT WingClip Week1 - WT WingRemoved Week3 0.70018 0.1475 Inf 4.746
 WT WingClip Week1 - Orco Single Week4 2.02751 0.1934 Inf 10.486
 WT WingClip Week1 - WT Single Week4 1.59844 0.1548 Inf 10.328
 WT WingClip Week1 - Orco WingClip Week4 0.80479 0.1290 Inf 6.237
 WT WingClip Week1 - WT WingClip Week4 0.37572 0.0602 Inf 6.241
 WT WingClip Week1 - Orco WingRemoved Week4 1.05144 0.1861 Inf 5.649
 WT WingClip Week1 - WT WingRemoved Week4 0.62237 0.1477 Inf 4.215
 WT WingClip Week1 - Orco Single Week5 1.60409 0.1918 Inf 8.365
 WT WingClip Week1 - WT Single Week5 1.17502 0.1527 Inf 7.693
 WT WingClip Week1 - Orco WingClip Week5 0.38137 0.1268 Inf 3.007
 WT WingClip Week1 - WT WingClip Week5 -0.04769 0.0551 Inf -0.865
 WT WingClip Week1 - Orco WingRemoved Week5 0.62802 0.1845 Inf 3.403
 WT WingClip Week1 - WT WingRemoved Week5 0.19895 0.1456 Inf 1.366
 Orco WingRemoved Week1 - WT WingRemoved Week1 -0.42907 0.1145 Inf -3.747
 Orco WingRemoved Week1 - Orco Single Week2 1.37008 0.1547 Inf 8.857
 Orco WingRemoved Week1 - WT Single Week2 0.94101 0.1912 Inf 4.922
 Orco WingRemoved Week1 - Orco WingClip Week2 0.14736 0.1463 Inf 1.007
 Orco WingRemoved Week1 - WT WingClip Week2 -0.28171 0.1855 Inf -1.518
 Orco WingRemoved Week1 - Orco WingRemoved Week2 0.39401 0.0573 Inf 6.881
 Orco WingRemoved Week1 - WT WingRemoved Week2 -0.03506 0.1284 Inf -0.273
 Orco WingRemoved Week1 - Orco Single Week3 1.42960 0.1556 Inf 9.187
 Orco WingRemoved Week1 - WT Single Week3 1.00053 0.1920 Inf 5.211
 Orco WingRemoved Week1 - Orco WingClip Week3 0.20688 0.1474 Inf 1.404
 Orco WingRemoved Week1 - WT WingClip Week3 -0.22218 0.1865 Inf -1.192
 Orco WingRemoved Week1 - Orco WingRemoved Week3 0.45353 0.0598 Inf 7.586
 Orco WingRemoved Week1 - WT WingRemoved Week3 0.02446 0.1297 Inf 0.189
 Orco WingRemoved Week1 - Orco Single Week4 1.35179 0.1558 Inf 8.679
 Orco WingRemoved Week1 - WT Single Week4 0.92272 0.1920 Inf 4.806
 Orco WingRemoved Week1 - Orco WingClip Week4 0.12908 0.1476 Inf 0.875
 Orco WingRemoved Week1 - WT WingClip Week4 -0.29999 0.1865 Inf -1.608
 Orco WingRemoved Week1 - Orco WingRemoved Week4 0.37572 0.0602 Inf 6.241
 Orco WingRemoved Week1 - WT WingRemoved Week4 -0.05334 0.1297 Inf -0.411
 Orco WingRemoved Week1 - Orco Single Week5 0.92837 0.1538 Inf 6.037
 Orco WingRemoved Week1 - WT Single Week5 0.49931 0.1904 Inf 2.623
 Orco WingRemoved Week1 - Orco WingClip Week5 -0.29434 0.1456 Inf -2.021
 Orco WingRemoved Week1 - WT WingClip Week5 -0.72341 0.1849 Inf -3.912
 Orco WingRemoved Week1 - Orco WingRemoved Week5 -0.04769 0.0551 Inf -0.865
 Orco WingRemoved Week1 - WT WingRemoved Week5 -0.47676 0.1274 Inf -3.743
 WT WingRemoved Week1 - Orco Single Week2 1.79915 0.1937 Inf 9.287
 WT WingRemoved Week1 - WT Single Week2 1.37008 0.1547 Inf 8.857
 WT WingRemoved Week1 - Orco WingClip Week2 0.57643 0.1860 Inf 3.098
 WT WingRemoved Week1 - WT WingClip Week2 0.14736 0.1463 Inf 1.007
 WT WingRemoved Week1 - Orco WingRemoved Week2 0.82308 0.1277 Inf 6.448
 WT WingRemoved Week1 - WT WingRemoved Week2 0.39401 0.0573 Inf 6.881
 WT WingRemoved Week1 - Orco Single Week3 1.85867 0.1944 Inf 9.561
 WT WingRemoved Week1 - WT Single Week3 1.42960 0.1556 Inf 9.187
 WT WingRemoved Week1 - Orco WingClip Week3 0.63595 0.1868 Inf 3.405
 WT WingRemoved Week1 - WT WingClip Week3 0.20688 0.1474 Inf 1.404
 WT WingRemoved Week1 - Orco WingRemoved Week3 0.88260 0.1287 Inf 6.858
 WT WingRemoved Week1 - WT WingRemoved Week3 0.45353 0.0598 Inf 7.586
 WT WingRemoved Week1 - Orco Single Week4 1.78086 0.1946 Inf 9.150
 WT WingRemoved Week1 - WT Single Week4 1.35179 0.1558 Inf 8.679
 WT WingRemoved Week1 - Orco WingClip Week4 0.55814 0.1871 Inf 2.984
 WT WingRemoved Week1 - WT WingClip Week4 0.12908 0.1476 Inf 0.875
 WT WingRemoved Week1 - Orco WingRemoved Week4 0.80479 0.1290 Inf 6.237
 WT WingRemoved Week1 - WT WingRemoved Week4 0.37572 0.0602 Inf 6.241
 WT WingRemoved Week1 - Orco Single Week5 1.35744 0.1931 Inf 7.030
 WT WingRemoved Week1 - WT Single Week5 0.92837 0.1538 Inf 6.037
 WT WingRemoved Week1 - Orco WingClip Week5 0.13473 0.1856 Inf 0.726
 WT WingRemoved Week1 - WT WingClip Week5 -0.29434 0.1456 Inf -2.021
 WT WingRemoved Week1 - Orco WingRemoved Week5 0.38137 0.1268 Inf 3.007
 WT WingRemoved Week1 - WT WingRemoved Week5 -0.04769 0.0551 Inf -0.865
 Orco Single Week2 - WT Single Week2 -0.42907 0.1145 Inf -3.747
 Orco Single Week2 - Orco WingClip Week2 -1.22272 0.1428 Inf -8.564
 Orco Single Week2 - WT WingClip Week2 -1.65179 0.1841 Inf -8.972
 Orco Single Week2 - Orco WingRemoved Week2 -0.97607 0.1439 Inf -6.784
 Orco Single Week2 - WT WingRemoved Week2 -1.40514 0.1855 Inf -7.576
 Orco Single Week2 - Orco Single Week3 0.05952 0.0651 Inf 0.914
 Orco Single Week2 - WT Single Week3 -0.36955 0.1318 Inf -2.803
 Orco Single Week2 - Orco WingClip Week3 -1.16319 0.1570 Inf -7.410
 Orco Single Week2 - WT WingClip Week3 -1.59226 0.1954 Inf -8.148
 Orco Single Week2 - Orco WingRemoved Week3 -0.91655 0.1579 Inf -5.803
 Orco Single Week2 - WT WingRemoved Week3 -1.34561 0.1967 Inf -6.842
 Orco Single Week2 - Orco Single Week4 -0.01829 0.0655 Inf -0.279
 Orco Single Week2 - WT Single Week4 -0.44735 0.1319 Inf -3.393
 Orco Single Week2 - Orco WingClip Week4 -1.24100 0.1572 Inf -7.895
 Orco Single Week2 - WT WingClip Week4 -1.67007 0.1955 Inf -8.544
 Orco Single Week2 - Orco WingRemoved Week4 -0.99435 0.1581 Inf -6.289
 Orco Single Week2 - WT WingRemoved Week4 -1.42342 0.1967 Inf -7.237
 Orco Single Week2 - Orco Single Week5 -0.44170 0.0608 Inf -7.259
 Orco Single Week2 - WT Single Week5 -0.87077 0.1296 Inf -6.720
 Orco Single Week2 - Orco WingClip Week5 -1.66442 0.1554 Inf -10.708
 Orco Single Week2 - WT WingClip Week5 -2.09349 0.1940 Inf -10.789
 Orco Single Week2 - Orco WingRemoved Week5 -1.41777 0.1563 Inf -9.069
 Orco Single Week2 - WT WingRemoved Week5 -1.84684 0.1952 Inf -9.459
 WT Single Week2 - Orco WingClip Week2 -0.79365 0.1819 Inf -4.363
 WT Single Week2 - WT WingClip Week2 -1.22272 0.1428 Inf -8.564
 WT Single Week2 - Orco WingRemoved Week2 -0.54700 0.1823 Inf -3.001
 WT Single Week2 - WT WingRemoved Week2 -0.97607 0.1439 Inf -6.784
 WT Single Week2 - Orco Single Week3 0.48859 0.1316 Inf 3.713
 WT Single Week2 - WT Single Week3 0.05952 0.0651 Inf 0.914
 WT Single Week2 - Orco WingClip Week3 -0.73413 0.1932 Inf -3.800
 WT Single Week2 - WT WingClip Week3 -1.16319 0.1570 Inf -7.410
 WT Single Week2 - Orco WingRemoved Week3 -0.48748 0.1935 Inf -2.519
 WT Single Week2 - WT WingRemoved Week3 -0.91655 0.1579 Inf -5.803
 WT Single Week2 - Orco Single Week4 0.41078 0.1319 Inf 3.113
 WT Single Week2 - WT Single Week4 -0.01829 0.0655 Inf -0.279
 WT Single Week2 - Orco WingClip Week4 -0.81193 0.1935 Inf -4.197
 WT Single Week2 - WT WingClip Week4 -1.24100 0.1572 Inf -7.895
 WT Single Week2 - Orco WingRemoved Week4 -0.56529 0.1937 Inf -2.918
 WT Single Week2 - WT WingRemoved Week4 -0.99435 0.1581 Inf -6.289
 WT Single Week2 - Orco Single Week5 -0.01264 0.1298 Inf -0.097
 WT Single Week2 - WT Single Week5 -0.44170 0.0608 Inf -7.259
 WT Single Week2 - Orco WingClip Week5 -1.23535 0.1921 Inf -6.431
 WT Single Week2 - WT WingClip Week5 -1.66442 0.1554 Inf -10.708
 WT Single Week2 - Orco WingRemoved Week5 -0.98870 0.1923 Inf -5.141
 WT Single Week2 - WT WingRemoved Week5 -1.41777 0.1563 Inf -9.069
 Orco WingClip Week2 - WT WingClip Week2 -0.42907 0.1145 Inf -3.747
 Orco WingClip Week2 - Orco WingRemoved Week2 0.24665 0.1348 Inf 1.830
 Orco WingClip Week2 - WT WingRemoved Week2 -0.18242 0.1774 Inf -1.028
 Orco WingClip Week2 - Orco Single Week3 1.28224 0.1568 Inf 8.176
 Orco WingClip Week2 - WT Single Week3 0.85317 0.1932 Inf 4.416
 Orco WingClip Week2 - Orco WingClip Week3 0.05952 0.0651 Inf 0.914
 Orco WingClip Week2 - WT WingClip Week3 -0.36955 0.1318 Inf -2.803
 Orco WingClip Week2 - Orco WingRemoved Week3 0.30617 0.1496 Inf 2.046
 Orco WingClip Week2 - WT WingRemoved Week3 -0.12290 0.1890 Inf -0.650
 Orco WingClip Week2 - Orco Single Week4 1.20443 0.1570 Inf 7.674
 Orco WingClip Week2 - WT Single Week4 0.77536 0.1932 Inf 4.013
 Orco WingClip Week2 - Orco WingClip Week4 -0.01829 0.0655 Inf -0.279
 Orco WingClip Week2 - WT WingClip Week4 -0.44735 0.1319 Inf -3.393
 Orco WingClip Week2 - Orco WingRemoved Week4 0.22836 0.1498 Inf 1.525
 Orco WingClip Week2 - WT WingRemoved Week4 -0.20071 0.1890 Inf -1.062
 Orco WingClip Week2 - Orco Single Week5 0.78101 0.1549 Inf 5.041
 Orco WingClip Week2 - WT Single Week5 0.35194 0.1915 Inf 1.837
 Orco WingClip Week2 - Orco WingClip Week5 -0.44170 0.0608 Inf -7.259
 Orco WingClip Week2 - WT WingClip Week5 -0.87077 0.1296 Inf -6.720
 Orco WingClip Week2 - Orco WingRemoved Week5 -0.19506 0.1477 Inf -1.320
 Orco WingClip Week2 - WT WingRemoved Week5 -0.62412 0.1873 Inf -3.331
 WT WingClip Week2 - Orco WingRemoved Week2 0.67572 0.1763 Inf 3.832
 WT WingClip Week2 - WT WingRemoved Week2 0.24665 0.1348 Inf 1.830
 WT WingClip Week2 - Orco Single Week3 1.71131 0.1951 Inf 8.770
 WT WingClip Week2 - WT Single Week3 1.28224 0.1568 Inf 8.176
 WT WingClip Week2 - Orco WingClip Week3 0.48859 0.1316 Inf 3.713
 WT WingClip Week2 - WT WingClip Week3 0.05952 0.0651 Inf 0.914
 WT WingClip Week2 - Orco WingRemoved Week3 0.73524 0.1878 Inf 3.914
 WT WingClip Week2 - WT WingRemoved Week3 0.30617 0.1496 Inf 2.046
 WT WingClip Week2 - Orco Single Week4 1.63350 0.1953 Inf 8.362
 WT WingClip Week2 - WT Single Week4 1.20443 0.1570 Inf 7.674
 WT WingClip Week2 - Orco WingClip Week4 0.41078 0.1319 Inf 3.113
 WT WingClip Week2 - WT WingClip Week4 -0.01829 0.0655 Inf -0.279
 WT WingClip Week2 - Orco WingRemoved Week4 0.65743 0.1881 Inf 3.496
 WT WingClip Week2 - WT WingRemoved Week4 0.22836 0.1498 Inf 1.525
 WT WingClip Week2 - Orco Single Week5 1.21008 0.1938 Inf 6.245
 WT WingClip Week2 - WT Single Week5 0.78101 0.1549 Inf 5.041
 WT WingClip Week2 - Orco WingClip Week5 -0.01264 0.1298 Inf -0.097
 WT WingClip Week2 - WT WingClip Week5 -0.44170 0.0608 Inf -7.259
 WT WingClip Week2 - Orco WingRemoved Week5 0.23401 0.1865 Inf 1.255
 WT WingClip Week2 - WT WingRemoved Week5 -0.19506 0.1477 Inf -1.320
 Orco WingRemoved Week2 - WT WingRemoved Week2 -0.42907 0.1145 Inf -3.747
 Orco WingRemoved Week2 - Orco Single Week3 1.03559 0.1579 Inf 6.559
 Orco WingRemoved Week2 - WT Single Week3 0.60652 0.1936 Inf 3.133
 Orco WingRemoved Week2 - Orco WingClip Week3 -0.18713 0.1497 Inf -1.250
 Orco WingRemoved Week2 - WT WingClip Week3 -0.61619 0.1881 Inf -3.276
 Orco WingRemoved Week2 - Orco WingRemoved Week3 0.05952 0.0651 Inf 0.914
 Orco WingRemoved Week2 - WT WingRemoved Week3 -0.36955 0.1318 Inf -2.803
 Orco WingRemoved Week2 - Orco Single Week4 0.95778 0.1580 Inf 6.060
 Orco WingRemoved Week2 - WT Single Week4 0.52871 0.1936 Inf 2.731
 Orco WingRemoved Week2 - Orco WingClip Week4 -0.26493 0.1499 Inf -1.767
 Orco WingRemoved Week2 - WT WingClip Week4 -0.69400 0.1881 Inf -3.689
 Orco WingRemoved Week2 - Orco WingRemoved Week4 -0.01829 0.0655 Inf -0.279
 Orco WingRemoved Week2 - WT WingRemoved Week4 -0.44735 0.1319 Inf -3.393
 Orco WingRemoved Week2 - Orco Single Week5 0.53436 0.1561 Inf 3.423
 Orco WingRemoved Week2 - WT Single Week5 0.10530 0.1920 Inf 0.548
 Orco WingRemoved Week2 - Orco WingClip Week5 -0.68835 0.1480 Inf -4.651
 Orco WingRemoved Week2 - WT WingClip Week5 -1.11742 0.1866 Inf -5.989
 Orco WingRemoved Week2 - Orco WingRemoved Week5 -0.44170 0.0608 Inf -7.259
 Orco WingRemoved Week2 - WT WingRemoved Week5 -0.87077 0.1296 Inf -6.720
 WT WingRemoved Week2 - Orco Single Week3 1.46466 0.1965 Inf 7.455
 WT WingRemoved Week2 - WT Single Week3 1.03559 0.1579 Inf 6.559
 WT WingRemoved Week2 - Orco WingClip Week3 0.24194 0.1889 Inf 1.281
 WT WingRemoved Week2 - WT WingClip Week3 -0.18713 0.1497 Inf -1.250
 WT WingRemoved Week2 - Orco WingRemoved Week3 0.48859 0.1316 Inf 3.713
 WT WingRemoved Week2 - WT WingRemoved Week3 0.05952 0.0651 Inf 0.914
 WT WingRemoved Week2 - Orco Single Week4 1.38685 0.1967 Inf 7.051
 WT WingRemoved Week2 - WT Single Week4 0.95778 0.1580 Inf 6.060
 WT WingRemoved Week2 - Orco WingClip Week4 0.16413 0.1892 Inf 0.868
 WT WingRemoved Week2 - WT WingClip Week4 -0.26493 0.1499 Inf -1.767
 WT WingRemoved Week2 - Orco WingRemoved Week4 0.41078 0.1319 Inf 3.113
 WT WingRemoved Week2 - WT WingRemoved Week4 -0.01829 0.0655 Inf -0.279
 WT WingRemoved Week2 - Orco Single Week5 0.96343 0.1952 Inf 4.936
 WT WingRemoved Week2 - WT Single Week5 0.53436 0.1561 Inf 3.423
 WT WingRemoved Week2 - Orco WingClip Week5 -0.25928 0.1877 Inf -1.381
 WT WingRemoved Week2 - WT WingClip Week5 -0.68835 0.1480 Inf -4.651
 WT WingRemoved Week2 - Orco WingRemoved Week5 -0.01264 0.1298 Inf -0.097
 WT WingRemoved Week2 - WT WingRemoved Week5 -0.44170 0.0608 Inf -7.259
 Orco Single Week3 - WT Single Week3 -0.42907 0.1145 Inf -3.747
 Orco Single Week3 - Orco WingClip Week3 -1.22272 0.1428 Inf -8.564
 Orco Single Week3 - WT WingClip Week3 -1.65179 0.1841 Inf -8.972
 Orco Single Week3 - Orco WingRemoved Week3 -0.97607 0.1439 Inf -6.784
 Orco Single Week3 - WT WingRemoved Week3 -1.40514 0.1855 Inf -7.576
 Orco Single Week3 - Orco Single Week4 -0.07781 0.0671 Inf -1.160
 Orco Single Week3 - WT Single Week4 -0.50688 0.1326 Inf -3.824
 Orco Single Week3 - Orco WingClip Week4 -1.30053 0.1578 Inf -8.243
 Orco Single Week3 - WT WingClip Week4 -1.72959 0.1959 Inf -8.830
 Orco Single Week3 - Orco WingRemoved Week4 -1.05388 0.1588 Inf -6.638
 Orco Single Week3 - WT WingRemoved Week4 -1.48295 0.1971 Inf -7.522
 Orco Single Week3 - Orco Single Week5 -0.50123 0.0626 Inf -8.005
 Orco Single Week3 - WT Single Week5 -0.93030 0.1303 Inf -7.140
 Orco Single Week3 - Orco WingClip Week5 -1.72394 0.1561 Inf -11.046
 Orco Single Week3 - WT WingClip Week5 -2.15301 0.1945 Inf -11.071
 Orco Single Week3 - Orco WingRemoved Week5 -1.47730 0.1570 Inf -9.409
 Orco Single Week3 - WT WingRemoved Week5 -1.90636 0.1957 Inf -9.741
 WT Single Week3 - Orco WingClip Week3 -0.79365 0.1819 Inf -4.363
 WT Single Week3 - WT WingClip Week3 -1.22272 0.1428 Inf -8.564
 WT Single Week3 - Orco WingRemoved Week3 -0.54700 0.1823 Inf -3.001
 WT Single Week3 - WT WingRemoved Week3 -0.97607 0.1439 Inf -6.784
 WT Single Week3 - Orco Single Week4 0.35126 0.1329 Inf 2.644
 WT Single Week3 - WT Single Week4 -0.07781 0.0671 Inf -1.160
 WT Single Week3 - Orco WingClip Week4 -0.87146 0.1940 Inf -4.492
 WT Single Week3 - WT WingClip Week4 -1.30053 0.1578 Inf -8.243
 WT Single Week3 - Orco WingRemoved Week4 -0.62481 0.1943 Inf -3.215
 WT Single Week3 - WT WingRemoved Week4 -1.05388 0.1588 Inf -6.638
 WT Single Week3 - Orco Single Week5 -0.07216 0.1307 Inf -0.552
 WT Single Week3 - WT Single Week5 -0.50123 0.0626 Inf -8.005
 WT Single Week3 - Orco WingClip Week5 -1.29488 0.1927 Inf -6.721
 WT Single Week3 - WT WingClip Week5 -1.72394 0.1561 Inf -11.046
 WT Single Week3 - Orco WingRemoved Week5 -1.04823 0.1929 Inf -5.433
 WT Single Week3 - WT WingRemoved Week5 -1.47730 0.1570 Inf -9.409
 Orco WingClip Week3 - WT WingClip Week3 -0.42907 0.1145 Inf -3.747
 Orco WingClip Week3 - Orco WingRemoved Week3 0.24665 0.1348 Inf 1.830
 Orco WingClip Week3 - WT WingRemoved Week3 -0.18242 0.1774 Inf -1.028
 Orco WingClip Week3 - Orco Single Week4 1.14491 0.1577 Inf 7.260
 Orco WingClip Week3 - WT Single Week4 0.71584 0.1937 Inf 3.695
 Orco WingClip Week3 - Orco WingClip Week4 -0.07781 0.0671 Inf -1.160
 Orco WingClip Week3 - WT WingClip Week4 -0.50688 0.1326 Inf -3.824
 Orco WingClip Week3 - Orco WingRemoved Week4 0.16884 0.1505 Inf 1.122
 Orco WingClip Week3 - WT WingRemoved Week4 -0.26023 0.1895 Inf -1.373
 Orco WingClip Week3 - Orco Single Week5 0.72149 0.1557 Inf 4.633
 Orco WingClip Week3 - WT Single Week5 0.29242 0.1921 Inf 1.522
 Orco WingClip Week3 - Orco WingClip Week5 -0.50123 0.0626 Inf -8.005
 Orco WingClip Week3 - WT WingClip Week5 -0.93030 0.1303 Inf -7.140
 Orco WingClip Week3 - Orco WingRemoved Week5 -0.25458 0.1485 Inf -1.714
 Orco WingClip Week3 - WT WingRemoved Week5 -0.68365 0.1879 Inf -3.639
 WT WingClip Week3 - Orco WingRemoved Week3 0.67572 0.1763 Inf 3.832
 WT WingClip Week3 - WT WingRemoved Week3 0.24665 0.1348 Inf 1.830
 WT WingClip Week3 - Orco Single Week4 1.57398 0.1960 Inf 8.030
 WT WingClip Week3 - WT Single Week4 1.14491 0.1577 Inf 7.260
 WT WingClip Week3 - Orco WingClip Week4 0.35126 0.1329 Inf 2.644
 WT WingClip Week3 - WT WingClip Week4 -0.07781 0.0671 Inf -1.160
 WT WingClip Week3 - Orco WingRemoved Week4 0.59791 0.1887 Inf 3.168
 WT WingClip Week3 - WT WingRemoved Week4 0.16884 0.1505 Inf 1.122
 WT WingClip Week3 - Orco Single Week5 1.15056 0.1945 Inf 5.916
 WT WingClip Week3 - WT Single Week5 0.72149 0.1557 Inf 4.633
 WT WingClip Week3 - Orco WingClip Week5 -0.07216 0.1307 Inf -0.552
 WT WingClip Week3 - WT WingClip Week5 -0.50123 0.0626 Inf -8.005
 WT WingClip Week3 - Orco WingRemoved Week5 0.17449 0.1872 Inf 0.932
 WT WingClip Week3 - WT WingRemoved Week5 -0.25458 0.1485 Inf -1.714
 Orco WingRemoved Week3 - WT WingRemoved Week3 -0.42907 0.1145 Inf -3.747
 Orco WingRemoved Week3 - Orco Single Week4 0.89826 0.1587 Inf 5.659
 Orco WingRemoved Week3 - WT Single Week4 0.46919 0.1941 Inf 2.417
 Orco WingRemoved Week3 - Orco WingClip Week4 -0.32446 0.1506 Inf -2.155
 Orco WingRemoved Week3 - WT WingClip Week4 -0.75353 0.1886 Inf -3.996
 Orco WingRemoved Week3 - Orco WingRemoved Week4 -0.07781 0.0671 Inf -1.160
 Orco WingRemoved Week3 - WT WingRemoved Week4 -0.50688 0.1326 Inf -3.824
 Orco WingRemoved Week3 - Orco Single Week5 0.47484 0.1568 Inf 3.028
 Orco WingRemoved Week3 - WT Single Week5 0.04577 0.1925 Inf 0.238
 Orco WingRemoved Week3 - Orco WingClip Week5 -0.74787 0.1487 Inf -5.029
 Orco WingRemoved Week3 - WT WingClip Week5 -1.17694 0.1870 Inf -6.293
 Orco WingRemoved Week3 - Orco WingRemoved Week5 -0.50123 0.0626 Inf -8.005
 Orco WingRemoved Week3 - WT WingRemoved Week5 -0.93030 0.1303 Inf -7.140
 WT WingRemoved Week3 - Orco Single Week4 1.32733 0.1973 Inf 6.726
 WT WingRemoved Week3 - WT Single Week4 0.89826 0.1587 Inf 5.659
 WT WingRemoved Week3 - Orco WingClip Week4 0.10461 0.1898 Inf 0.551
 WT WingRemoved Week3 - WT WingClip Week4 -0.32446 0.1506 Inf -2.155
 WT WingRemoved Week3 - Orco WingRemoved Week4 0.35126 0.1329 Inf 2.644
 WT WingRemoved Week3 - WT WingRemoved Week4 -0.07781 0.0671 Inf -1.160
 WT WingRemoved Week3 - Orco Single Week5 0.90391 0.1958 Inf 4.616
 WT WingRemoved Week3 - WT Single Week5 0.47484 0.1568 Inf 3.028
 WT WingRemoved Week3 - Orco WingClip Week5 -0.31881 0.1883 Inf -1.693
 WT WingRemoved Week3 - WT WingClip Week5 -0.74787 0.1487 Inf -5.029
 WT WingRemoved Week3 - Orco WingRemoved Week5 -0.07216 0.1307 Inf -0.552
 WT WingRemoved Week3 - WT WingRemoved Week5 -0.50123 0.0626 Inf -8.005
 Orco Single Week4 - WT Single Week4 -0.42907 0.1145 Inf -3.747
 Orco Single Week4 - Orco WingClip Week4 -1.22272 0.1428 Inf -8.564
 Orco Single Week4 - WT WingClip Week4 -1.65179 0.1841 Inf -8.972
 Orco Single Week4 - Orco WingRemoved Week4 -0.97607 0.1439 Inf -6.784
 Orco Single Week4 - WT WingRemoved Week4 -1.40514 0.1855 Inf -7.576
 Orco Single Week4 - Orco Single Week5 -0.42342 0.0623 Inf -6.801
 Orco Single Week4 - WT Single Week5 -0.85249 0.1303 Inf -6.543
 Orco Single Week4 - Orco WingClip Week5 -1.64614 0.1559 Inf -10.560
 Orco Single Week4 - WT WingClip Week5 -2.07520 0.1944 Inf -10.673
 Orco Single Week4 - Orco WingRemoved Week5 -1.39949 0.1569 Inf -8.922
 Orco Single Week4 - WT WingRemoved Week5 -1.82856 0.1957 Inf -9.345
 WT Single Week4 - Orco WingClip Week4 -0.79365 0.1819 Inf -4.363
 WT Single Week4 - WT WingClip Week4 -1.22272 0.1428 Inf -8.564
 WT Single Week4 - Orco WingRemoved Week4 -0.54700 0.1823 Inf -3.001
 WT Single Week4 - WT WingRemoved Week4 -0.97607 0.1439 Inf -6.784
 WT Single Week4 - Orco Single Week5 0.00565 0.1304 Inf 0.043
 WT Single Week4 - WT Single Week5 -0.42342 0.0623 Inf -6.801
 WT Single Week4 - Orco WingClip Week5 -1.21707 0.1924 Inf -6.325
 WT Single Week4 - WT WingClip Week5 -1.64614 0.1559 Inf -10.560
 WT Single Week4 - Orco WingRemoved Week5 -0.97042 0.1927 Inf -5.035
 WT Single Week4 - WT WingRemoved Week5 -1.39949 0.1569 Inf -8.922
 Orco WingClip Week4 - WT WingClip Week4 -0.42907 0.1145 Inf -3.747
 Orco WingClip Week4 - Orco WingRemoved Week4 0.24665 0.1348 Inf 1.830
 Orco WingClip Week4 - WT WingRemoved Week4 -0.18242 0.1774 Inf -1.028
 Orco WingClip Week4 - Orco Single Week5 0.79930 0.1556 Inf 5.136
 Orco WingClip Week4 - WT Single Week5 0.37023 0.1921 Inf 1.927
 Orco WingClip Week4 - Orco WingClip Week5 -0.42342 0.0623 Inf -6.801
 Orco WingClip Week4 - WT WingClip Week5 -0.85249 0.1303 Inf -6.543
 Orco WingClip Week4 - Orco WingRemoved Week5 -0.17677 0.1484 Inf -1.191
 Orco WingClip Week4 - WT WingRemoved Week5 -0.60584 0.1879 Inf -3.224
 WT WingClip Week4 - Orco WingRemoved Week4 0.67572 0.1763 Inf 3.832
 WT WingClip Week4 - WT WingRemoved Week4 0.24665 0.1348 Inf 1.830
 WT WingClip Week4 - Orco Single Week5 1.22837 0.1943 Inf 6.323
 WT WingClip Week4 - WT Single Week5 0.79930 0.1556 Inf 5.136
 WT WingClip Week4 - Orco WingClip Week5 0.00565 0.1304 Inf 0.043
 WT WingClip Week4 - WT WingClip Week5 -0.42342 0.0623 Inf -6.801
 WT WingClip Week4 - Orco WingRemoved Week5 0.25230 0.1870 Inf 1.349
 WT WingClip Week4 - WT WingRemoved Week5 -0.17677 0.1484 Inf -1.191
 Orco WingRemoved Week4 - WT WingRemoved Week4 -0.42907 0.1145 Inf -3.747
 Orco WingRemoved Week4 - Orco Single Week5 0.55265 0.1567 Inf 3.527
 Orco WingRemoved Week4 - WT Single Week5 0.12358 0.1925 Inf 0.642
 Orco WingRemoved Week4 - Orco WingClip Week5 -0.67007 0.1485 Inf -4.512
 Orco WingRemoved Week4 - WT WingClip Week5 -1.09913 0.1870 Inf -5.878
 Orco WingRemoved Week4 - Orco WingRemoved Week5 -0.42342 0.0623 Inf -6.801
 Orco WingRemoved Week4 - WT WingRemoved Week5 -0.85249 0.1303 Inf -6.543
 WT WingRemoved Week4 - Orco Single Week5 0.98172 0.1956 Inf 5.019
 WT WingRemoved Week4 - WT Single Week5 0.55265 0.1567 Inf 3.527
 WT WingRemoved Week4 - Orco WingClip Week5 -0.24100 0.1881 Inf -1.281
 WT WingRemoved Week4 - WT WingClip Week5 -0.67007 0.1485 Inf -4.512
 WT WingRemoved Week4 - Orco WingRemoved Week5 0.00565 0.1304 Inf 0.043
 WT WingRemoved Week4 - WT WingRemoved Week5 -0.42342 0.0623 Inf -6.801
 Orco Single Week5 - WT Single Week5 -0.42907 0.1145 Inf -3.747
 Orco Single Week5 - Orco WingClip Week5 -1.22272 0.1428 Inf -8.564
 Orco Single Week5 - WT WingClip Week5 -1.65179 0.1841 Inf -8.972
 Orco Single Week5 - Orco WingRemoved Week5 -0.97607 0.1439 Inf -6.784
 Orco Single Week5 - WT WingRemoved Week5 -1.40514 0.1855 Inf -7.576
 WT Single Week5 - Orco WingClip Week5 -0.79365 0.1819 Inf -4.363
 WT Single Week5 - WT WingClip Week5 -1.22272 0.1428 Inf -8.564
 WT Single Week5 - Orco WingRemoved Week5 -0.54700 0.1823 Inf -3.001
 WT Single Week5 - WT WingRemoved Week5 -0.97607 0.1439 Inf -6.784
 Orco WingClip Week5 - WT WingClip Week5 -0.42907 0.1145 Inf -3.747
 Orco WingClip Week5 - Orco WingRemoved Week5 0.24665 0.1348 Inf 1.830
 Orco WingClip Week5 - WT WingRemoved Week5 -0.18242 0.1774 Inf -1.028
 WT WingClip Week5 - Orco WingRemoved Week5 0.67572 0.1763 Inf 3.832
 WT WingClip Week5 - WT WingRemoved Week5 0.24665 0.1348 Inf 1.830
 Orco WingRemoved Week5 - WT WingRemoved Week5 -0.42907 0.1145 Inf -3.747
 p.value
 0.0502
 <.0001
 <.0001
 <.0001
 <.0001
 <.0001
 1.0000
 <.0001
 <.0001
 0.0490
 0.0001
 <.0001
 1.0000
 0.0003
 <.0001
 0.1695
 0.0004
 <.0001
 1.0000
 <.0001
 <.0001
 0.0358
 0.0001
 1.0000
 0.0509
 <.0001
 <.0001
 <.0001
 <.0001
 0.0046
 <.0001
 0.3835
 <.0001
 <.0001
 <.0001
 0.9617
 <.0001
 1.0000
 0.0490
 <.0001
 <.0001
 0.9962
 0.0003
 1.0000
 0.1695
 <.0001
 <.0001
 0.9395
 <.0001
 1.0000
 0.0358
 0.3787
 1.0000
 0.0036
 <.0001
 0.2972
 <.0001
 0.0502
 0.9940
 1.0000
 <.0001
 <.0001
 <.0001
 1.0000
 0.0045
 1.0000
 <.0001
 <.0001
 <.0001
 1.0000
 0.0008
 0.9999
 <.0001
 <.0001
 <.0001
 1.0000
 0.0086
 1.0000
 <.0001
 0.0267
 1.0000
 0.0509
 1.0000
 1.0000
 0.0374
 0.9940
 <.0001
 <.0001
 <.0001
 <.0001
 <.0001
 0.0045
 <.0001
 <.0001
 <.0001
 <.0001
 <.0001
 0.0008
 <.0001
 <.0001
 <.0001
 <.0001
 <.0001
 0.0086
 <.0001
 <.0001
 0.3787
 1.0000
 0.1463
 1.0000
 0.0502
 <.0001
 0.0003
 1.0000
 0.9998
 <.0001
 1.0000
 <.0001
 0.0001
 0.9999
 1.0000
 <.0001
 1.0000
 <.0001
 0.0006
 1.0000
 0.9993
 <.0001
 1.0000
 <.0001
 0.6907
 0.9759
 0.0281
 1.0000
 0.0509
 <.0001
 <.0001
 0.3131
 1.0000
 <.0001
 <.0001
 <.0001
 <.0001
 0.1456
 0.9999
 <.0001
 <.0001
 <.0001
 <.0001
 0.3966
 1.0000
 <.0001
 <.0001
 <.0001
 <.0001
 1.0000
 0.9759
 0.3787
 1.0000
 0.0502
 <.0001
 <.0001
 <.0001
 <.0001
 1.0000
 0.5429
 <.0001
 <.0001
 <.0001
 <.0001
 1.0000
 0.1507
 <.0001
 <.0001
 <.0001
 <.0001
 <.0001
 <.0001
 <.0001
 <.0001
 <.0001
 <.0001
 0.0046
 <.0001
 0.3835
 <.0001
 0.0564
 1.0000
 0.0419
 <.0001
 0.7677
 <.0001
 0.3030
 1.0000
 0.0093
 <.0001
 0.4486
 <.0001
 1.0000
 <.0001
 <.0001
 <.0001
 0.0001
 <.0001
 0.0502
 0.9940
 1.0000
 <.0001
 0.0037
 1.0000
 0.5429
 0.9718
 1.0000
 <.0001
 0.0192
 1.0000
 0.1507
 0.9997
 1.0000
 0.0002
 0.9937
 <.0001
 <.0001
 1.0000
 0.1779
 0.0374
 0.9940
 <.0001
 <.0001
 0.0564
 1.0000
 0.0278
 0.9718
 <.0001
 <.0001
 0.3030
 1.0000
 0.1119
 0.9997
 <.0001
 0.0002
 1.0000
 <.0001
 1.0000
 1.0000
 0.0502
 <.0001
 0.2901
 1.0000
 0.2054
 1.0000
 0.5429
 <.0001
 0.6033
 0.9965
 0.0611
 1.0000
 0.1507
 0.1382
 1.0000
 0.0013
 <.0001
 <.0001
 <.0001
 <.0001
 <.0001
 1.0000
 1.0000
 0.0564
 1.0000
 <.0001
 <.0001
 1.0000
 0.9965
 0.3030
 1.0000
 0.0003
 0.1382
 1.0000
 0.0013
 1.0000
 <.0001
 0.0502
 <.0001
 <.0001
 <.0001
 <.0001
 1.0000
 0.0385
 <.0001
 <.0001
 <.0001
 <.0001
 <.0001
 <.0001
 <.0001
 <.0001
 <.0001
 <.0001
 0.0046
 <.0001
 0.3835
 <.0001
 0.6740
 1.0000
 0.0026
 <.0001
 0.2392
 <.0001
 1.0000
 <.0001
 <.0001
 <.0001
 <.0001
 <.0001
 0.0502
 0.9940
 1.0000
 <.0001
 0.0599
 1.0000
 0.0385
 1.0000
 1.0000
 0.0014
 0.9997
 <.0001
 <.0001
 0.9979
 0.0720
 0.0374
 0.9940
 <.0001
 <.0001
 0.6740
 1.0000
 0.2676
 1.0000
 <.0001
 0.0014
 1.0000
 <.0001
 1.0000
 0.9979
 0.0502
 <.0001
 0.8340
 0.9475
 0.0205
 1.0000
 0.0385
 0.3634
 1.0000
 0.0002
 <.0001
 <.0001
 <.0001
 <.0001
 <.0001
 1.0000
 0.9475
 0.6740
 1.0000
 0.0015
 0.3634
 0.9983
 0.0002
 1.0000
 <.0001
 0.0502
 <.0001
 <.0001
 <.0001
 <.0001
 <.0001
 <.0001
 <.0001
 <.0001
 <.0001
 <.0001
 0.0046
 <.0001
 0.3835
 <.0001
 1.0000
 <.0001
 <.0001
 <.0001
 0.0002
 <.0001
 0.0502
 0.9940
 1.0000
 0.0001
 0.9873
 <.0001
 <.0001
 1.0000
 0.2340
 0.0374
 0.9940
 <.0001
 0.0001
 1.0000
 <.0001
 1.0000
 1.0000
 0.0502
 0.1020
 1.0000
 0.0024
 <.0001
 <.0001
 <.0001
 0.0002
 0.1020
 1.0000
 0.0024
 1.0000
 <.0001
 0.0502
 <.0001
 <.0001
 <.0001
 <.0001
 0.0046
 <.0001
 0.3835
 <.0001
 0.0502
 0.9940
 1.0000
 0.0374
 0.9940
 0.0502

Results are given on the log (not the response) scale.
P value adjustment: tukey method for comparing a family of 30 estimates

lsmeans(OrcoFeeding_glmmTMB2, pairwise ~ Focal_Treatment + Rival_Treatment, adjust="tukey")

$lsmeans
 Focal_Treatment Rival_Treatment lsmean SE df asymp.LCL asymp.UCL
 Orco Single -0.616 0.126 Inf -0.863 -0.3687
 WT Single -0.187 0.121 Inf -0.424 0.0503
 Orco WingClip 0.607 0.112 Inf 0.387 0.8272
 WT WingClip 1.036 0.108 Inf 0.824 1.2487
 Orco WingRemoved 0.360 0.115 Inf 0.135 0.5854
 WT WingRemoved 0.789 0.112 Inf 0.570 1.0086

Results are averaged over the levels of: Week
Results are given on the log (not the response) scale.
Confidence level used: 0.95

$contrasts
 contrast estimate SE df z.ratio p.value
 Orco Single - WT Single -0.429 0.115 Inf -3.747 0.0025
 Orco Single - Orco WingClip -1.223 0.143 Inf -8.564 <.0001
 Orco Single - WT WingClip -1.652 0.184 Inf -8.972 <.0001
 Orco Single - Orco WingRemoved -0.976 0.144 Inf -6.784 <.0001
 Orco Single - WT WingRemoved -1.405 0.185 Inf -7.576 <.0001
 WT Single - Orco WingClip -0.794 0.182 Inf -4.363 0.0002
 WT Single - WT WingClip -1.223 0.143 Inf -8.564 <.0001
 WT Single - Orco WingRemoved -0.547 0.182 Inf -3.001 0.0322
 WT Single - WT WingRemoved -0.976 0.144 Inf -6.784 <.0001
 Orco WingClip - WT WingClip -0.429 0.115 Inf -3.747 0.0025
 Orco WingClip - Orco WingRemoved 0.247 0.135 Inf 1.830 0.4462
 Orco WingClip - WT WingRemoved -0.182 0.177 Inf -1.028 0.9085
 WT WingClip - Orco WingRemoved 0.676 0.176 Inf 3.832 0.0018
 WT WingClip - WT WingRemoved 0.247 0.135 Inf 1.830 0.4462
 Orco WingRemoved - WT WingRemoved -0.429 0.115 Inf -3.747 0.0025

Results are averaged over the levels of: Week
Results are given on the log (not the response) scale.
P value adjustment: tukey method for comparing a family of 6 estimates

lsmeans(OrcoFeeding_glmmTMB2, pairwise ~ Focal_Treatment, adjust="tukey")

$lsmeans
 Focal_Treatment lsmean SE df asymp.LCL asymp.UCL
 Orco 0.117 0.0855 Inf -0.0503 0.285
 WT 0.546 0.0799 Inf 0.3898 0.703

Results are averaged over the levels of: Rival_Treatment, Week
Results are given on the log (not the response) scale.
Confidence level used: 0.95

$contrasts
 contrast estimate SE df z.ratio p.value
 Orco - WT -0.429 0.115 Inf -3.747 0.0002

Results are averaged over the levels of: Rival_Treatment, Week
Results are given on the log (not the response) scale.

lsmeans(OrcoFeeding_glmmTMB2, pairwise ~ Rival_Treatment, adjust="tukey")

$lsmeans
 Rival_Treatment lsmean SE df asymp.LCL asymp.UCL
 Single -0.401 0.1094 Inf -0.616 -0.187
 WingClip 0.822 0.0944 Inf 0.637 1.007
 WingRemoved 0.575 0.0978 Inf 0.383 0.767

Results are averaged over the levels of: Focal_Treatment, Week
Results are given on the log (not the response) scale.
Confidence level used: 0.95

$contrasts
 contrast estimate SE df z.ratio p.value
 Single - WingClip -1.223 0.143 Inf -8.564 <.0001
 Single - WingRemoved -0.976 0.144 Inf -6.784 <.0001
 WingClip - WingRemoved 0.247 0.135 Inf 1.830 0.1598

Results are averaged over the levels of: Focal_Treatment, Week
Results are given on the log (not the response) scale.
P value adjustment: tukey method for comparing a family of 3 estimates

lsmeans(OrcoFeeding_glmmTMB2, pairwise ~ Week, adjust="tukey")

$lsmeans
 Week lsmean SE df asymp.LCL asymp.UCL
 1 0.567 0.0665 Inf 0.4365 0.697
 2 0.173 0.0720 Inf 0.0318 0.314
 3 0.113 0.0738 Inf -0.0313 0.258
 4 0.191 0.0741 Inf 0.0460 0.336
 5 0.615 0.0699 Inf 0.4776 0.752

Results are averaged over the levels of: Focal_Treatment, Rival_Treatment
Results are given on the log (not the response) scale.
Confidence level used: 0.95

$contrasts
 contrast estimate SE df z.ratio p.value
 Week1 - Week2 0.3940 0.0573 Inf 6.881 <.0001
 Week1 - Week3 0.4535 0.0598 Inf 7.586 <.0001
 Week1 - Week4 0.3757 0.0602 Inf 6.241 <.0001
 Week1 - Week5 -0.0477 0.0551 Inf -0.865 0.9097
 Week2 - Week3 0.0595 0.0651 Inf 0.914 0.8915
 Week2 - Week4 -0.0183 0.0655 Inf -0.279 0.9987
 Week2 - Week5 -0.4417 0.0608 Inf -7.259 <.0001
 Week3 - Week4 -0.0778 0.0671 Inf -1.160 0.7742
 Week3 - Week5 -0.5012 0.0626 Inf -8.005 <.0001
 Week4 - Week5 -0.4234 0.0623 Inf -6.801 <.0001

Results are averaged over the levels of: Focal_Treatment, Rival_Treatment
Results are given on the log (not the response) scale.
P value adjustment: tukey method for comparing a family of 5 estimates

##Orco Food Line
Orco_Food$PercentSuccess <- Orco_Food$Success / 10
Orco_Food_subset <- subset(Orco_Food, PercentSuccess > 0)
Orco_Food_subset$Rival_Treatment <- factor(Orco_Food_subset$Rival_Treatment, levels = c("Single", "WingClip", "WingRemoved"))
Orco_Food_subset$Focal_Treatment <- factor(Orco_Food_subset$Focal_Treatment, levels = c("WT", "Orco"))
Orco_Food_subset$Interaction <- interaction(Orco_Food_subset$Focal_Treatment, Orco_Food_subset$Rival_Treatment)
Orco_Food_subset$Interaction <- factor(Orco_Food_subset$Interaction, levels = c("WT.Single", "WT.WingClip", "WT.WingRemoved", "Orco.Single", "Orco.WingClip", "Orco.WingRemoved"))

Orco_Food_Summary <- summarySE(Orco_Food_subset,
 measurevar = "PercentSuccess",
 groupvars = c("Week", "Interaction", "Focal_Treatment", "Rival_Treatment"))

Orco_Food_Line <- ggplot(Orco_Food_Summary) +
 aes(
 x = Week,
 y = PercentSuccess,
 colour = Interaction,
 group = Interaction
 ) +
 scale_colour_manual(name = "Treatment",
 labels = c("Wildtype kept alone",
 "Wildtype kept with Wing Clipped Rival",
 "Wildtype kept with Wing Removed Rival",
 "Orco2 kept alone",
 "Orco2 kept with Wing Clipped Rival",
 "Orco2 kept with Wing Removed Rival"),
 values = c("black",
 "grey70",
 "grey42",
 "black",
 "grey70",
 "grey42")) +
 geom_line(aes(linetype = Interaction), size = 3) +
 geom_point() +
 geom_errorbar(aes(ymin=PercentSuccess-se, ymax=PercentSuccess+se), width=0.2, size = 1.5) +
 scale_linetype_manual(name = "Treatment",
 labels = c("Wildtype kept alone",
 "Wildtype kept with Wing Clipped Rival",
 "Wildtype kept with Wing Removed Rival",
 "Orco2 kept alone",
 "Orco2 kept with Wing Clipped Rival",
 "Orco2 kept with Wing Removed Rival"),
 values = c("solid", "solid", "solid", "dotdash", "dotdash", "dotdash")) +
 labs(
 x = "Week post Eclosion",
 y = "Proportion of Time Spent on Food"
 ) +
 ylim(0, 1.05) +
 theme_bw() +
 guides(colour = guide_legend(override.aes = list(linetype = c("solid", "solid", "solid", "dotdash", "dotdash", "dotdash"))))

Warning: Using `size` aesthetic for lines was deprecated in ggplot2 3.4.0.
ℹ Please use `linewidth` instead.

Orco_Food_Line_Plot <- Orco_Food_Line + theme_bw() + theme(panel.grid.major = element_blank(), panel.grid.minor = element_blank()) +
 theme(axis.text=element_text(size=55, face="bold", colour="black"),
 axis.title=element_text(size=75, face="bold", colour="black"),
 legend.text=element_text(size=55, face="bold", colour="black"),
 legend.title=element_text(size=75, face="bold", colour="black"),
 strip.text=element_text(size=75, face="bold", colour="black")) + theme(axis.line = element_line(size = 5), panel.border = element_rect(size = 5)) + theme( axis.ticks.length = unit(0.5, "cm"), axis.ticks = element_line(size = 5))


Orco_Food_Line_Plot


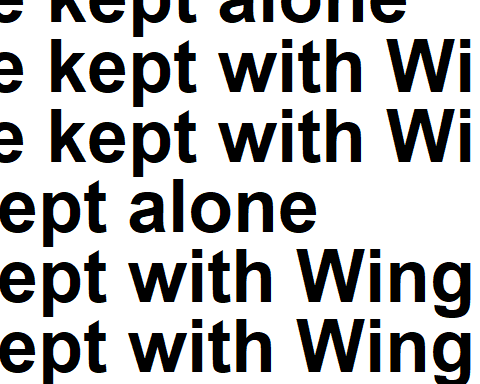


###Orco Walking
##Orco Walking Model
Orco_Walking$Week <- as.factor(Orco_Walking$Week)
OrcoWalking_glmmTMB1 <- glmmTMB(Success ~ Focal_Treatment * Rival_Treatment + Week + (1|FlyID), family=poisson(link = "log"), data = Orco_Walking)
OrcoWalking_glmmTMB2 <- glmmTMB(Success ~ Focal_Treatment + Rival_Treatment + Week + (1|FlyID), family=poisson(link = "log"), data = Orco_Walking)
anova(OrcoWalking_glmmTMB1, OrcoWalking_glmmTMB2, test="Chi")

Data: Orco_Walking
Models:
OrcoWalking_glmmTMB2: Success ~ Focal_Treatment + Rival_Treatment + Week + (1 | FlyID), zi=~0, disp=~1
OrcoWalking_glmmTMB1: Success ~ Focal_Treatment * Rival_Treatment + Week + (1 | FlyID), zi=~0, disp=~1
 Df AIC BIC logLik deviance Chisq Chi Df Pr(>Chisq)
OrcoWalking_glmmTMB2 9 5187.4 5234.1 -2584.7 5169.4
OrcoWalking_glmmTMB1 11 5184.6 5241.7 -2581.3 5162.6 6.7507 2 0.03421

OrcoWalking_glmmTMB2
OrcoWalking_glmmTMB1 *
---
Signif. codes: 0 '***' 0.001 '**' 0.01 '*' 0.05 '.' 0.1 ' ' 1

OrcoWalking_glmmTMB3 <- glmmTMB(Success ~ Rival_Treatment + Week + (1|FlyID), family=poisson(link = "log"), data = Orco_Walking)
anova(OrcoWalking_glmmTMB2, OrcoWalking_glmmTMB3, test="Chi")

Data: Orco_Walking
Models:
OrcoWalking_glmmTMB3: Success ~ Rival_Treatment + Week + (1 | FlyID), zi=~0, disp=~1
OrcoWalking_glmmTMB2: Success ~ Focal_Treatment + Rival_Treatment + Week + (1 | FlyID), zi=~0, disp=~1
 Df AIC BIC logLik deviance Chisq Chi Df Pr(>Chisq)
OrcoWalking_glmmTMB3 8 5304.6 5346.1 -2644.3 5288.6
OrcoWalking_glmmTMB2 9 5187.4 5234.1 -2584.7 5169.4 119.2 1 < 2.2e-16

OrcoWalking_glmmTMB3
OrcoWalking_glmmTMB2 ***
---
Signif. codes: 0 '***' 0.001 '**' 0.01 '*' 0.05 '.' 0.1 ' ' 1

OrcoWalking_glmmTMB4 <- glmmTMB(Success ~ Focal_Treatment + Week + (1|FlyID), family=poisson(link = "log"), data = Orco_Walking)
anova(OrcoWalking_glmmTMB2, OrcoWalking_glmmTMB4, test="Chi")

Data: Orco_Walking
Models:
OrcoWalking_glmmTMB4: Success ~ Focal_Treatment + Week + (1 | FlyID), zi=~0, disp=~1
OrcoWalking_glmmTMB2: Success ~ Focal_Treatment + Rival_Treatment + Week + (1 | FlyID), zi=~0, disp=~1
 Df AIC BIC logLik deviance Chisq Chi Df Pr(>Chisq)
OrcoWalking_glmmTMB4 7 5188.1 5224.4 -2587.1 5174.1
OrcoWalking_glmmTMB2 9 5187.4 5234.1 -2584.7 5169.4 4.7106 2 0.09487

OrcoWalking_glmmTMB4
OrcoWalking_glmmTMB2 .
---
Signif. codes: 0 '***' 0.001 '**' 0.01 '*' 0.05 '.' 0.1 ' ' 1

OrcoWalking_glmmTMB5 <- glmmTMB(Success ~ Focal_Treatment + Rival_Treatment + (1|FlyID), family=poisson(link = "log"), data = Orco_Walking)
anova(OrcoWalking_glmmTMB2, OrcoWalking_glmmTMB5, test="Chi")

Data: Orco_Walking
Models:
OrcoWalking_glmmTMB5: Success ~ Focal_Treatment + Rival_Treatment + (1 | FlyID), zi=~0, disp=~1
OrcoWalking_glmmTMB2: Success ~ Focal_Treatment + Rival_Treatment + Week + (1 | FlyID), zi=~0, disp=~1
 Df AIC BIC logLik deviance Chisq Chi Df Pr(>Chisq)
OrcoWalking_glmmTMB5 5 5264.8 5290.7 -2627.4 5254.8
OrcoWalking_glmmTMB2 9 5187.4 5234.1 -2584.7 5169.4 85.429 4 < 2.2e-16

OrcoWalking_glmmTMB5
OrcoWalking_glmmTMB2 ***
---
Signif. codes: 0 '***' 0.001 '**' 0.01 '*' 0.05 '.' 0.1 ' ' 1

OrcoWalking_glmmTMB_null <- glmmTMB(Success ~ (1|FlyID), family=poisson(link = "log"), data = Orco_Walking)
anova(OrcoWalking_glmmTMB1, OrcoWalking_glmmTMB_null, test="Chi")

Data: Orco_Walking
Models:
OrcoWalking_glmmTMB_null: Success ~ (1 | FlyID), zi=~0, disp=~1
OrcoWalking_glmmTMB1: Success ~ Focal_Treatment * Rival_Treatment + Week + (1 | FlyID), zi=~0, disp=~1
 Df AIC BIC logLik deviance Chisq Chi Df
OrcoWalking_glmmTMB_null 2 5378.1 5388.5 -2687.1 5374.1
OrcoWalking_glmmTMB1 11 5184.6 5241.7 -2581.3 5162.6 211.47 9
 Pr(>Chisq)
OrcoWalking_glmmTMB_null
OrcoWalking_glmmTMB1 < 2.2e-16 ***
---
Signif. codes: 0 '***' 0.001 '**' 0.01 '*' 0.05 '.' 0.1 ' ' 1

summary(OrcoWalking_glmmTMB1)

Family: poisson ( log )
Formula:
Success ~ Focal_Treatment * Rival_Treatment + Week + (1 | FlyID)
Data: Orco_Walking

 AIC BIC logLik deviance df.resid
 5184.6 5241.7 -2581.3 5162.6 1308

Random effects:

Conditional model:
 Groups Name Variance Std.Dev.
 FlyID (Intercept) 0.5485 0.7406
Number of obs: 1319, groups: FlyID, 300

Conditional model:
 Estimate Std. Error z value
(Intercept) -0.65577 0.14266 -4.597
Focal_TreatmentWT 1.58568 0.17571 9.025
Rival_TreatmentWingClip 0.08073 0.18653 0.433
Rival_TreatmentWingRemoved 0.08033 0.18702 0.430
Week2 0.36404 0.06307 5.772
Week3 0.37193 0.06377 5.832
Week4 0.46761 0.06359 7.354
Week5 0.53806 0.06361 8.458
Focal_TreatmentWT:Rival_TreatmentWingClip -0.51862 0.24720 -2.098
Focal_TreatmentWT:Rival_TreatmentWingRemoved -0.58892 0.24903 -2.365
 Pr(>|z|)
(Intercept) 4.29e-06 ***
Focal_TreatmentWT < 2e-16 ***
Rival_TreatmentWingClip 0.6652
Rival_TreatmentWingRemoved 0.6675
Week2 7.86e-09 ***
Week3 5.46e-09 ***
Week4 1.92e-13 ***
Week5 < 2e-16 ***
Focal_TreatmentWT:Rival_TreatmentWingClip 0.0359 *
Focal_TreatmentWT:Rival_TreatmentWingRemoved 0.0180 *
---
Signif. codes: 0 '***' 0.001 '**' 0.01 '*' 0.05 '.' 0.1 ' ' 1

lsmeans(OrcoWalking_glmmTMB2, pairwise ~ Focal_Treatment + Rival_Treatment * Week, adjust="tukey")

$lsmeans
 Focal_Treatment Rival_Treatment Week lsmean SE df asymp.LCL asymp.UCL
 Orco Single 1 -0.4505 0.116 Inf -0.677 -0.2236
 WT Single 1 0.7721 0.107 Inf 0.563 0.9811
 Orco WingClip 1 -0.6634 0.115 Inf -0.889 -0.4376
 WT WingClip 1 0.5592 0.107 Inf 0.349 0.7693
 Orco WingRemoved 1 -0.7028 0.116 Inf -0.931 -0.4749
 WT WingRemoved 1 0.5199 0.108 Inf 0.308 0.7322
 Orco Single 2 -0.0866 0.113 Inf -0.308 0.1349
 WT Single 2 1.1361 0.104 Inf 0.931 1.3408
 Orco WingClip 2 -0.2995 0.112 Inf -0.519 -0.0797
 WT WingClip 2 0.9232 0.105 Inf 0.718 1.1284
 Orco WingRemoved 2 -0.3388 0.114 Inf -0.562 -0.1157
 WT WingRemoved 2 0.8838 0.107 Inf 0.675 1.0927
 Orco Single 3 -0.0796 0.113 Inf -0.302 0.1427
 WT Single 3 1.1430 0.105 Inf 0.937 1.3489
 Orco WingClip 3 -0.2925 0.113 Inf -0.513 -0.0720
 WT WingClip 3 0.9301 0.105 Inf 0.724 1.1364
 Orco WingRemoved 3 -0.3318 0.114 Inf -0.556 -0.1079
 WT WingRemoved 3 0.8908 0.107 Inf 0.681 1.1008
 Orco Single 4 0.0154 0.113 Inf -0.207 0.2378
 WT Single 4 1.2380 0.105 Inf 1.032 1.4437
 Orco WingClip 4 -0.1975 0.113 Inf -0.418 0.0230
 WT WingClip 4 1.0251 0.105 Inf 0.819 1.2312
 Orco WingRemoved 4 -0.2368 0.114 Inf -0.461 -0.0129
 WT WingRemoved 4 0.9858 0.107 Inf 0.776 1.1955
 Orco Single 5 0.0854 0.114 Inf -0.137 0.3079
 WT Single 5 1.3080 0.105 Inf 1.102 1.5137
 Orco WingClip 5 -0.1275 0.113 Inf -0.349 0.0934
 WT WingClip 5 1.0951 0.105 Inf 0.889 1.3014
 Orco WingRemoved 5 -0.1669 0.114 Inf -0.391 0.0574
 WT WingRemoved 5 1.0558 0.107 Inf 0.846 1.2657

Results are given on the log (not the response) scale.
Confidence level used: 0.95

$contrasts
 contrast estimate SE df z.ratio
 Orco Single Week1 - WT Single Week1 -1.22266 0.1028 Inf -11.889
 Orco Single Week1 - Orco WingClip Week1 0.21289 0.1233 Inf 1.727
 Orco Single Week1 - WT WingClip Week1 -1.00977 0.1613 Inf -6.258
 Orco Single Week1 - Orco WingRemoved Week1 0.25221 0.1241 Inf 2.032
 Orco Single Week1 - WT WingRemoved Week1 -0.97045 0.1620 Inf -5.990
 Orco Single Week1 - Orco Single Week2 -0.36394 0.0631 Inf -5.769
 Orco Single Week1 - WT Single Week2 -1.58660 0.1214 Inf -13.073
 Orco Single Week1 - Orco WingClip Week2 -0.15105 0.1383 Inf -1.093
 Orco Single Week1 - WT WingClip Week2 -1.37371 0.1735 Inf -7.915
 Orco Single Week1 - Orco WingRemoved Week2 -0.11174 0.1395 Inf -0.801
 Orco Single Week1 - WT WingRemoved Week2 -1.33439 0.1746 Inf -7.644
 Orco Single Week1 - Orco Single Week3 -0.37091 0.0638 Inf -5.816
 Orco Single Week1 - WT Single Week3 -1.59356 0.1219 Inf -13.078
 Orco Single Week1 - Orco WingClip Week3 -0.15802 0.1385 Inf -1.141
 Orco Single Week1 - WT WingClip Week3 -1.38067 0.1738 Inf -7.942
 Orco Single Week1 - Orco WingRemoved Week3 -0.11870 0.1398 Inf -0.849
 Orco Single Week1 - WT WingRemoved Week3 -1.34136 0.1749 Inf -7.669
 Orco Single Week1 - Orco Single Week4 -0.46593 0.0636 Inf -7.328
 Orco Single Week1 - WT Single Week4 -1.68859 0.1216 Inf -13.882
 Orco Single Week1 - Orco WingClip Week4 -0.25304 0.1384 Inf -1.828
 Orco Single Week1 - WT WingClip Week4 -1.47570 0.1737 Inf -8.496
 Orco Single Week1 - Orco WingRemoved Week4 -0.21372 0.1397 Inf -1.530
 Orco Single Week1 - WT WingRemoved Week4 -1.43638 0.1747 Inf -8.221
 Orco Single Week1 - Orco Single Week5 -0.53590 0.0636 Inf -8.426
 Orco Single Week1 - WT Single Week5 -1.75856 0.1216 Inf -14.467
 Orco Single Week1 - Orco WingClip Week5 -0.32301 0.1385 Inf -2.332
 Orco Single Week1 - WT WingClip Week5 -1.54567 0.1737 Inf -8.898
 Orco Single Week1 - Orco WingRemoved Week5 -0.28370 0.1398 Inf -2.030
 Orco Single Week1 - WT WingRemoved Week5 -1.50635 0.1747 Inf -8.622
 WT Single Week1 - Orco WingClip Week1 1.43555 0.1598 Inf 8.985
 WT Single Week1 - WT WingClip Week1 0.21289 0.1233 Inf 1.727
 WT Single Week1 - Orco WingRemoved Week1 1.47486 0.1604 Inf 9.195
 WT Single Week1 - WT WingRemoved Week1 0.25221 0.1241 Inf 2.032
 WT Single Week1 - Orco Single Week2 0.85872 0.1199 Inf 7.161
 WT Single Week1 - WT Single Week2 -0.36394 0.0631 Inf -5.769
 WT Single Week1 - Orco WingClip Week2 1.07160 0.1711 Inf 6.264
 WT Single Week1 - WT WingClip Week2 -0.15105 0.1383 Inf -1.093
 WT Single Week1 - Orco WingRemoved Week2 1.11092 0.1721 Inf 6.456
 WT Single Week1 - WT WingRemoved Week2 -0.11174 0.1395 Inf -0.801
 WT Single Week1 - Orco Single Week3 0.85175 0.1202 Inf 7.089
 WT Single Week1 - WT Single Week3 -0.37091 0.0638 Inf -5.816
 WT Single Week1 - Orco WingClip Week3 1.06464 0.1712 Inf 6.220
 WT Single Week1 - WT WingClip Week3 -0.15802 0.1385 Inf -1.141
 WT Single Week1 - Orco WingRemoved Week3 1.10396 0.1722 Inf 6.410
 WT Single Week1 - WT WingRemoved Week3 -0.11870 0.1398 Inf -0.849
 WT Single Week1 - Orco Single Week4 0.75673 0.1202 Inf 6.297
 WT Single Week1 - WT Single Week4 -0.46593 0.0636 Inf -7.328
 WT Single Week1 - Orco WingClip Week4 0.96962 0.1712 Inf 5.664
 WT Single Week1 - WT WingClip Week4 -0.25304 0.1384 Inf -1.828
 WT Single Week1 - Orco WingRemoved Week4 1.00893 0.1722 Inf 5.859
 WT Single Week1 - WT WingRemoved Week4 -0.21372 0.1397 Inf -1.530
 WT Single Week1 - Orco Single Week5 0.68676 0.1203 Inf 5.710
 WT Single Week1 - WT Single Week5 -0.53590 0.0636 Inf -8.426
 WT Single Week1 - Orco WingClip Week5 0.89965 0.1713 Inf 5.251
 WT Single Week1 - WT WingClip Week5 -0.32301 0.1385 Inf -2.332
 WT Single Week1 - Orco WingRemoved Week5 0.93896 0.1723 Inf 5.449
 WT Single Week1 - WT WingRemoved Week5 -0.28370 0.1398 Inf -2.030
 Orco WingClip Week1 - WT WingClip Week1 -1.22266 0.1028 Inf -11.889
 Orco WingClip Week1 - Orco WingRemoved Week1 0.03932 0.1249 Inf 0.315
 Orco WingClip Week1 - WT WingRemoved Week1 -1.18334 0.1618 Inf -7.312
 Orco WingClip Week1 - Orco Single Week2 -0.57683 0.1387 Inf -4.158
 Orco WingClip Week1 - WT Single Week2 -1.79949 0.1725 Inf -10.434
 Orco WingClip Week1 - Orco WingClip Week2 -0.36394 0.0631 Inf -5.769
 Orco WingClip Week1 - WT WingClip Week2 -1.58660 0.1214 Inf -13.073
 Orco WingClip Week1 - Orco WingRemoved Week2 -0.32463 0.1405 Inf -2.311
 Orco WingClip Week1 - WT WingRemoved Week2 -1.54728 0.1746 Inf -8.862
 Orco WingClip Week1 - Orco Single Week3 -0.58379 0.1391 Inf -4.196
 Orco WingClip Week1 - WT Single Week3 -1.80645 0.1729 Inf -10.450
 Orco WingClip Week1 - Orco WingClip Week3 -0.37091 0.0638 Inf -5.816
 Orco WingClip Week1 - WT WingClip Week3 -1.59356 0.1219 Inf -13.078
 Orco WingClip Week1 - Orco WingRemoved Week3 -0.33159 0.1408 Inf -2.354
 Orco WingClip Week1 - WT WingRemoved Week3 -1.55425 0.1750 Inf -8.882
 Orco WingClip Week1 - Orco Single Week4 -0.67882 0.1390 Inf -4.882
 Orco WingClip Week1 - WT Single Week4 -1.90148 0.1727 Inf -11.009
 Orco WingClip Week1 - Orco WingClip Week4 -0.46593 0.0636 Inf -7.328
 Orco WingClip Week1 - WT WingClip Week4 -1.68859 0.1216 Inf -13.882
 Orco WingClip Week1 - Orco WingRemoved Week4 -0.42661 0.1407 Inf -3.032
 Orco WingClip Week1 - WT WingRemoved Week4 -1.64927 0.1748 Inf -9.435
 Orco WingClip Week1 - Orco Single Week5 -0.74879 0.1389 Inf -5.389
 Orco WingClip Week1 - WT Single Week5 -1.97145 0.1726 Inf -11.423
 Orco WingClip Week1 - Orco WingClip Week5 -0.53590 0.0636 Inf -8.426
 Orco WingClip Week1 - WT WingClip Week5 -1.75856 0.1216 Inf -14.467
 Orco WingClip Week1 - Orco WingRemoved Week5 -0.49659 0.1407 Inf -3.530
 Orco WingClip Week1 - WT WingRemoved Week5 -1.71924 0.1747 Inf -9.840
 WT WingClip Week1 - Orco WingRemoved Week1 1.26197 0.1618 Inf 7.800
 WT WingClip Week1 - WT WingRemoved Week1 0.03932 0.1249 Inf 0.315
 WT WingClip Week1 - Orco Single Week2 0.64583 0.1729 Inf 3.735
 WT WingClip Week1 - WT Single Week2 -0.57683 0.1387 Inf -4.158
 WT WingClip Week1 - Orco WingClip Week2 0.85872 0.1199 Inf 7.161
 WT WingClip Week1 - WT WingClip Week2 -0.36394 0.0631 Inf -5.769
 WT WingClip Week1 - Orco WingRemoved Week2 0.89803 0.1736 Inf 5.174
 WT WingClip Week1 - WT WingRemoved Week2 -0.32463 0.1405 Inf -2.311
 WT WingClip Week1 - Orco Single Week3 0.63886 0.1731 Inf 3.690
 WT WingClip Week1 - WT Single Week3 -0.58379 0.1391 Inf -4.196
 WT WingClip Week1 - Orco WingClip Week3 0.85175 0.1202 Inf 7.089
 WT WingClip Week1 - WT WingClip Week3 -0.37091 0.0638 Inf -5.816
 WT WingClip Week1 - Orco WingRemoved Week3 0.89107 0.1738 Inf 5.128
 WT WingClip Week1 - WT WingRemoved Week3 -0.33159 0.1408 Inf -2.354
 WT WingClip Week1 - Orco Single Week4 0.54384 0.1732 Inf 3.141
 WT WingClip Week1 - WT Single Week4 -0.67882 0.1390 Inf -4.882
 WT WingClip Week1 - Orco WingClip Week4 0.75673 0.1202 Inf 6.297
 WT WingClip Week1 - WT WingClip Week4 -0.46593 0.0636 Inf -7.328
 WT WingClip Week1 - Orco WingRemoved Week4 0.79604 0.1738 Inf 4.581
 WT WingClip Week1 - WT WingRemoved Week4 -0.42661 0.1407 Inf -3.032
 WT WingClip Week1 - Orco Single Week5 0.47387 0.1731 Inf 2.737
 WT WingClip Week1 - WT Single Week5 -0.74879 0.1389 Inf -5.389
 WT WingClip Week1 - Orco WingClip Week5 0.68676 0.1203 Inf 5.710
 WT WingClip Week1 - WT WingClip Week5 -0.53590 0.0636 Inf -8.426
 WT WingClip Week1 - Orco WingRemoved Week5 0.72607 0.1738 Inf 4.178
 WT WingClip Week1 - WT WingRemoved Week5 -0.49659 0.1407 Inf -3.530
 Orco WingRemoved Week1 - WT WingRemoved Week1 -1.22266 0.1028 Inf -11.889
 Orco WingRemoved Week1 - Orco Single Week2 -0.61615 0.1390 Inf -4.433
 Orco WingRemoved Week1 - WT Single Week2 -1.83881 0.1726 Inf -10.651
 Orco WingRemoved Week1 - Orco WingClip Week2 -0.40326 0.1394 Inf -2.892
 Orco WingRemoved Week1 - WT WingClip Week2 -1.62592 0.1738 Inf -9.358
 Orco WingRemoved Week1 - Orco WingRemoved Week2 -0.36394 0.0631 Inf -5.769
 Orco WingRemoved Week1 - WT WingRemoved Week2 -1.58660 0.1214 Inf -13.073
 Orco WingRemoved Week1 - Orco Single Week3 -0.62311 0.1393 Inf -4.473
 Orco WingRemoved Week1 - WT Single Week3 -1.84577 0.1730 Inf -10.670
 Orco WingRemoved Week1 - Orco WingClip Week3 -0.41022 0.1397 Inf -2.936
 Orco WingRemoved Week1 - WT WingClip Week3 -1.63288 0.1740 Inf -9.382
 Orco WingRemoved Week1 - Orco WingRemoved Week3 -0.37091 0.0638 Inf -5.816
 Orco WingRemoved Week1 - WT WingRemoved Week3 -1.59356 0.1219 Inf -13.078
 Orco WingRemoved Week1 - Orco Single Week4 -0.71814 0.1392 Inf -5.157
 Orco WingRemoved Week1 - WT Single Week4 -1.94079 0.1729 Inf -11.227
 Orco WingRemoved Week1 - Orco WingClip Week4 -0.50525 0.1396 Inf -3.618
 Orco WingRemoved Week1 - WT WingClip Week4 -1.72790 0.1739 Inf -9.935
 Orco WingRemoved Week1 - Orco WingRemoved Week4 -0.46593 0.0636 Inf -7.328
 Orco WingRemoved Week1 - WT WingRemoved Week4 -1.68859 0.1216 Inf -13.882
 Orco WingRemoved Week1 - Orco Single Week5 -0.78811 0.1392 Inf -5.662
 Orco WingRemoved Week1 - WT Single Week5 -2.01077 0.1728 Inf -11.639
 Orco WingRemoved Week1 - Orco WingClip Week5 -0.57522 0.1397 Inf -4.118
 Orco WingRemoved Week1 - WT WingClip Week5 -1.79788 0.1739 Inf -10.338
 Orco WingRemoved Week1 - Orco WingRemoved Week5 -0.53590 0.0636 Inf -8.426
 Orco WingRemoved Week1 - WT WingRemoved Week5 -1.75856 0.1216 Inf -14.467
 WT WingRemoved Week1 - Orco Single Week2 0.60651 0.1731 Inf 3.503
 WT WingRemoved Week1 - WT Single Week2 -0.61615 0.1390 Inf -4.433
 WT WingRemoved Week1 - Orco WingClip Week2 0.81940 0.1728 Inf 4.743
 WT WingRemoved Week1 - WT WingClip Week2 -0.40326 0.1394 Inf -2.892
 WT WingRemoved Week1 - Orco WingRemoved Week2 0.85872 0.1199 Inf 7.161
 WT WingRemoved Week1 - WT WingRemoved Week2 -0.36394 0.0631 Inf -5.769
 WT WingRemoved Week1 - Orco Single Week3 0.59955 0.1733 Inf 3.460
 WT WingRemoved Week1 - WT Single Week3 -0.62311 0.1393 Inf -4.473
 WT WingRemoved Week1 - Orco WingClip Week3 0.81244 0.1729 Inf 4.699
 WT WingRemoved Week1 - WT WingClip Week3 -0.41022 0.1397 Inf -2.936
 WT WingRemoved Week1 - Orco WingRemoved Week3 0.85175 0.1202 Inf 7.089
 WT WingRemoved Week1 - WT WingRemoved Week3 -0.37091 0.0638 Inf -5.816
 WT WingRemoved Week1 - Orco Single Week4 0.50452 0.1733 Inf 2.911
 WT WingRemoved Week1 - WT Single Week4 -0.71814 0.1392 Inf -5.157
 WT WingRemoved Week1 - Orco WingClip Week4 0.71741 0.1729 Inf 4.149
 WT WingRemoved Week1 - WT WingClip Week4 -0.50525 0.1396 Inf -3.618
 WT WingRemoved Week1 - Orco WingRemoved Week4 0.75673 0.1202 Inf 6.297
 WT WingRemoved Week1 - WT WingRemoved Week4 -0.46593 0.0636 Inf -7.328
 WT WingRemoved Week1 - Orco Single Week5 0.43455 0.1734 Inf 2.507
 WT WingRemoved Week1 - WT Single Week5 -0.78811 0.1392 Inf -5.662
 WT WingRemoved Week1 - Orco WingClip Week5 0.64744 0.1730 Inf 3.742
 WT WingRemoved Week1 - WT WingClip Week5 -0.57522 0.1397 Inf -4.118
 WT WingRemoved Week1 - Orco WingRemoved Week5 0.68676 0.1203 Inf 5.710
 WT WingRemoved Week1 - WT WingRemoved Week5 -0.53590 0.0636 Inf -8.426
 Orco Single Week2 - WT Single Week2 -1.22266 0.1028 Inf -11.889
 Orco Single Week2 - Orco WingClip Week2 0.21289 0.1233 Inf 1.727
 Orco Single Week2 - WT WingClip Week2 -1.00977 0.1613 Inf -6.258
 Orco Single Week2 - Orco WingRemoved Week2 0.25221 0.1241 Inf 2.032
 Orco Single Week2 - WT WingRemoved Week2 -0.97045 0.1620 Inf -5.990
 Orco Single Week2 - Orco Single Week3 -0.00696 0.0583 Inf -0.119
 Orco Single Week2 - WT Single Week3 -1.22962 0.1184 Inf -10.389
 Orco Single Week2 - Orco WingClip Week3 0.20593 0.1363 Inf 1.511
 Orco Single Week2 - WT WingClip Week3 -1.01673 0.1716 Inf -5.925
 Orco Single Week2 - Orco WingRemoved Week3 0.24524 0.1371 Inf 1.788
 Orco Single Week2 - WT WingRemoved Week3 -0.97741 0.1723 Inf -5.674
 Orco Single Week2 - Orco Single Week4 -0.10199 0.0581 Inf -1.755
 Orco Single Week2 - WT Single Week4 -1.32465 0.1181 Inf -11.213
 Orco Single Week2 - Orco WingClip Week4 0.11090 0.1362 Inf 0.814
 Orco Single Week2 - WT WingClip Week4 -1.11176 0.1714 Inf -6.484
 Orco Single Week2 - Orco WingRemoved Week4 0.15022 0.1370 Inf 1.096
 Orco Single Week2 - WT WingRemoved Week4 -1.07244 0.1721 Inf -6.232
 Orco Single Week2 - Orco Single Week5 -0.17196 0.0581 Inf -2.958
 Orco Single Week2 - WT Single Week5 -1.39462 0.1181 Inf -11.814
 Orco Single Week2 - Orco WingClip Week5 0.04093 0.1364 Inf 0.300
 Orco Single Week2 - WT WingClip Week5 -1.18173 0.1715 Inf -6.892
 Orco Single Week2 - Orco WingRemoved Week5 0.08025 0.1371 Inf 0.585
 Orco Single Week2 - WT WingRemoved Week5 -1.14241 0.1721 Inf -6.639
 WT Single Week2 - Orco WingClip Week2 1.43555 0.1598 Inf 8.985
 WT Single Week2 - WT WingClip Week2 0.21289 0.1233 Inf 1.727
 WT Single Week2 - Orco WingRemoved Week2 1.47486 0.1604 Inf 9.195
 WT Single Week2 - WT WingRemoved Week2 0.25221 0.1241 Inf 2.032
 WT Single Week2 - Orco Single Week3 1.21569 0.1181 Inf 10.294
 WT Single Week2 - WT Single Week3 -0.00696 0.0583 Inf -0.119
 WT Single Week2 - Orco WingClip Week3 1.42858 0.1699 Inf 8.407
 WT Single Week2 - WT WingClip Week3 0.20593 0.1363 Inf 1.511
 WT Single Week2 - Orco WingRemoved Week3 1.46790 0.1706 Inf 8.606
 WT Single Week2 - WT WingRemoved Week3 0.24524 0.1371 Inf 1.788
 WT Single Week2 - Orco Single Week4 1.12067 0.1181 Inf 9.488
 WT Single Week2 - WT Single Week4 -0.10199 0.0581 Inf -1.755
 WT Single Week2 - Orco WingClip Week4 1.33356 0.1699 Inf 7.847
 WT Single Week2 - WT WingClip Week4 0.11090 0.1362 Inf 0.814
 WT Single Week2 - Orco WingRemoved Week4 1.37288 0.1706 Inf 8.049
 WT Single Week2 - WT WingRemoved Week4 0.15022 0.1370 Inf 1.096
 WT Single Week2 - Orco Single Week5 1.05070 0.1182 Inf 8.889
 WT Single Week2 - WT Single Week5 -0.17196 0.0581 Inf -2.958
 WT Single Week2 - Orco WingClip Week5 1.26359 0.1701 Inf 7.429
 WT Single Week2 - WT WingClip Week5 0.04093 0.1364 Inf 0.300
 WT Single Week2 - Orco WingRemoved Week5 1.30290 0.1707 Inf 7.634
 WT Single Week2 - WT WingRemoved Week5 0.08025 0.1371 Inf 0.585
 Orco WingClip Week2 - WT WingClip Week2 -1.22266 0.1028 Inf -11.889
 Orco WingClip Week2 - Orco WingRemoved Week2 0.03932 0.1249 Inf 0.315
 Orco WingClip Week2 - WT WingRemoved Week2 -1.18334 0.1618 Inf -7.312
 Orco WingClip Week2 - Orco Single Week3 -0.21985 0.1365 Inf -1.611
 Orco WingClip Week2 - WT Single Week3 -1.44251 0.1702 Inf -8.474
 Orco WingClip Week2 - Orco WingClip Week3 -0.00696 0.0583 Inf -0.119
 Orco WingClip Week2 - WT WingClip Week3 -1.22962 0.1184 Inf -10.389
 Orco WingClip Week2 - Orco WingRemoved Week3 0.03235 0.1379 Inf 0.235
 Orco WingClip Week2 - WT WingRemoved Week3 -1.19030 0.1722 Inf -6.914
 Orco WingClip Week2 - Orco Single Week4 -0.31488 0.1364 Inf -2.309
 Orco WingClip Week2 - WT Single Week4 -1.53754 0.1701 Inf -9.041
 Orco WingClip Week2 - Orco WingClip Week4 -0.10199 0.0581 Inf -1.755
 Orco WingClip Week2 - WT WingClip Week4 -1.32465 0.1181 Inf -11.213
 Orco WingClip Week2 - Orco WingRemoved Week4 -0.06267 0.1378 Inf -0.455
 Orco WingClip Week2 - WT WingRemoved Week4 -1.28533 0.1720 Inf -7.474
 Orco WingClip Week2 - Orco Single Week5 -0.38485 0.1363 Inf -2.824
 Orco WingClip Week2 - WT Single Week5 -1.60751 0.1699 Inf -9.460
 Orco WingClip Week2 - Orco WingClip Week5 -0.17196 0.0581 Inf -2.958
 Orco WingClip Week2 - WT WingClip Week5 -1.39462 0.1181 Inf -11.814
 Orco WingClip Week2 - Orco WingRemoved Week5 -0.13264 0.1378 Inf -0.963
 Orco WingClip Week2 - WT WingRemoved Week5 -1.35530 0.1719 Inf -7.885
 WT WingClip Week2 - Orco WingRemoved Week2 1.26197 0.1618 Inf 7.800
 WT WingClip Week2 - WT WingRemoved Week2 0.03932 0.1249 Inf 0.315
 WT WingClip Week2 - Orco Single Week3 1.00280 0.1715 Inf 5.846
 WT WingClip Week2 - WT Single Week3 -0.21985 0.1365 Inf -1.611
 WT WingClip Week2 - Orco WingClip Week3 1.21569 0.1181 Inf 10.294
 WT WingClip Week2 - WT WingClip Week3 -0.00696 0.0583 Inf -0.119
 WT WingClip Week2 - Orco WingRemoved Week3 1.25501 0.1719 Inf 7.299
 WT WingClip Week2 - WT WingRemoved Week3 0.03235 0.1379 Inf 0.235
 WT WingClip Week2 - Orco Single Week4 0.90778 0.1715 Inf 5.292
 WT WingClip Week2 - WT Single Week4 -0.31488 0.1364 Inf -2.309
 WT WingClip Week2 - Orco WingClip Week4 1.12067 0.1181 Inf 9.488
 WT WingClip Week2 - WT WingClip Week4 -0.10199 0.0581 Inf -1.755
 WT WingClip Week2 - Orco WingRemoved Week4 1.15999 0.1719 Inf 6.747
 WT WingClip Week2 - WT WingRemoved Week4 -0.06267 0.1378 Inf -0.455
 WT WingClip Week2 - Orco Single Week5 0.83781 0.1715 Inf 4.885
 WT WingClip Week2 - WT Single Week5 -0.38485 0.1363 Inf -2.824
 WT WingClip Week2 - Orco WingClip Week5 1.05070 0.1182 Inf 8.889
 WT WingClip Week2 - WT WingClip Week5 -0.17196 0.0581 Inf -2.958
 WT WingClip Week2 - Orco WingRemoved Week5 1.09001 0.1720 Inf 6.339
 WT WingClip Week2 - WT WingRemoved Week5 -0.13264 0.1378 Inf -0.963
 Orco WingRemoved Week2 - WT WingRemoved Week2 -1.22266 0.1028 Inf -11.889
 Orco WingRemoved Week2 - Orco Single Week3 -0.25917 0.1372 Inf -1.889
 Orco WingRemoved Week2 - WT Single Week3 -1.48183 0.1708 Inf -8.677
 Orco WingRemoved Week2 - Orco WingClip Week3 -0.04628 0.1378 Inf -0.336
 Orco WingRemoved Week2 - WT WingClip Week3 -1.26894 0.1720 Inf -7.376
 Orco WingRemoved Week2 - Orco WingRemoved Week3 -0.00696 0.0583 Inf -0.119
 Orco WingRemoved Week2 - WT WingRemoved Week3 -1.22962 0.1184 Inf -10.389
 Orco WingRemoved Week2 - Orco Single Week4 -0.35419 0.1371 Inf -2.583
 Orco WingRemoved Week2 - WT Single Week4 -1.57685 0.1706 Inf -9.241
 Orco WingRemoved Week2 - Orco WingClip Week4 -0.14130 0.1378 Inf -1.026
 Orco WingRemoved Week2 - WT WingClip Week4 -1.36396 0.1719 Inf -7.934
 Orco WingRemoved Week2 - Orco WingRemoved Week4 -0.10199 0.0581 Inf -1.755
 Orco WingRemoved Week2 - WT WingRemoved Week4 -1.32465 0.1181 Inf -11.213
 Orco WingRemoved Week2 - Orco Single Week5 -0.42417 0.1371 Inf -3.095
 Orco WingRemoved Week2 - WT Single Week5 -1.64682 0.1705 Inf -9.657
 Orco WingRemoved Week2 - Orco WingClip Week5 -0.21128 0.1378 Inf -1.533
 Orco WingRemoved Week2 - WT WingClip Week5 -1.43393 0.1719 Inf -8.342
 Orco WingRemoved Week2 - Orco WingRemoved Week5 -0.17196 0.0581 Inf -2.958
 Orco WingRemoved Week2 - WT WingRemoved Week5 -1.39462 0.1181 Inf -11.814
 WT WingRemoved Week2 - Orco Single Week3 0.96349 0.1721 Inf 5.599
 WT WingRemoved Week2 - WT Single Week3 -0.25917 0.1372 Inf -1.889
 WT WingRemoved Week2 - Orco WingClip Week3 1.17638 0.1719 Inf 6.844
 WT WingRemoved Week2 - WT WingClip Week3 -0.04628 0.1378 Inf -0.336
 WT WingRemoved Week2 - Orco WingRemoved Week3 1.21569 0.1181 Inf 10.294
 WT WingRemoved Week2 - WT WingRemoved Week3 -0.00696 0.0583 Inf -0.119
 WT WingRemoved Week2 - Orco Single Week4 0.86846 0.1721 Inf 5.045
 WT WingRemoved Week2 - WT Single Week4 -0.35419 0.1371 Inf -2.583
 WT WingRemoved Week2 - Orco WingClip Week4 1.08135 0.1719 Inf 6.290
 WT WingRemoved Week2 - WT WingClip Week4 -0.14130 0.1378 Inf -1.026
 WT WingRemoved Week2 - Orco WingRemoved Week4 1.12067 0.1181 Inf 9.488
 WT WingRemoved Week2 - WT WingRemoved Week4 -0.10199 0.0581 Inf -1.755
 WT WingRemoved Week2 - Orco Single Week5 0.79849 0.1722 Inf 4.638
 WT WingRemoved Week2 - WT Single Week5 -0.42417 0.1371 Inf -3.095
 WT WingRemoved Week2 - Orco WingClip Week5 1.01138 0.1720 Inf 5.879
 WT WingRemoved Week2 - WT WingClip Week5 -0.21128 0.1378 Inf -1.533
 WT WingRemoved Week2 - Orco WingRemoved Week5 1.05070 0.1182 Inf 8.889
 WT WingRemoved Week2 - WT WingRemoved Week5 -0.17196 0.0581 Inf -2.958
 Orco Single Week3 - WT Single Week3 -1.22266 0.1028 Inf -11.889
 Orco Single Week3 - Orco WingClip Week3 0.21289 0.1233 Inf 1.727
 Orco Single Week3 - WT WingClip Week3 -1.00977 0.1613 Inf -6.258
 Orco Single Week3 - Orco WingRemoved Week3 0.25221 0.1241 Inf 2.032
 Orco Single Week3 - WT WingRemoved Week3 -0.97045 0.1620 Inf -5.990
 Orco Single Week3 - Orco Single Week4 -0.09503 0.0584 Inf -1.627
 Orco Single Week3 - WT Single Week4 -1.31768 0.1181 Inf -11.153
 Orco Single Week3 - Orco WingClip Week4 0.11786 0.1364 Inf 0.864
 Orco Single Week3 - WT WingClip Week4 -1.10479 0.1715 Inf -6.441
 Orco Single Week3 - Orco WingRemoved Week4 0.15718 0.1372 Inf 1.146
 Orco Single Week3 - WT WingRemoved Week4 -1.06548 0.1721 Inf -6.191
 Orco Single Week3 - Orco Single Week5 -0.16500 0.0585 Inf -2.823
 Orco Single Week3 - WT Single Week5 -1.38765 0.1181 Inf -11.752
 Orco Single Week3 - Orco WingClip Week5 0.04789 0.1366 Inf 0.351
 Orco Single Week3 - WT WingClip Week5 -1.17476 0.1715 Inf -6.848
 Orco Single Week3 - Orco WingRemoved Week5 0.08721 0.1372 Inf 0.635
 Orco Single Week3 - WT WingRemoved Week5 -1.13545 0.1721 Inf -6.598
 WT Single Week3 - Orco WingClip Week3 1.43555 0.1598 Inf 8.985
 WT Single Week3 - WT WingClip Week3 0.21289 0.1233 Inf 1.727
 WT Single Week3 - Orco WingRemoved Week3 1.47486 0.1604 Inf 9.195
 WT Single Week3 - WT WingRemoved Week3 0.25221 0.1241 Inf 2.032
 WT Single Week3 - Orco Single Week4 1.12763 0.1184 Inf 9.525
 WT Single Week3 - WT Single Week4 -0.09503 0.0584 Inf -1.627
 WT Single Week3 - Orco WingClip Week4 1.34052 0.1702 Inf 7.877
 WT Single Week3 - WT WingClip Week4 0.11786 0.1364 Inf 0.864
 WT Single Week3 - Orco WingRemoved Week4 1.37984 0.1708 Inf 8.081
 WT Single Week3 - WT WingRemoved Week4 0.15718 0.1372 Inf 1.146
 WT Single Week3 - Orco Single Week5 1.05766 0.1185 Inf 8.925
 WT Single Week3 - WT Single Week5 -0.16500 0.0585 Inf -2.823
 WT Single Week3 - Orco WingClip Week5 1.27055 0.1704 Inf 7.458
 WT Single Week3 - WT WingClip Week5 0.04789 0.1366 Inf 0.351
 WT Single Week3 - Orco WingRemoved Week5 1.30987 0.1709 Inf 7.665
 WT Single Week3 - WT WingRemoved Week5 0.08721 0.1372 Inf 0.635
 Orco WingClip Week3 - WT WingClip Week3 -1.22266 0.1028 Inf -11.889
 Orco WingClip Week3 - Orco WingRemoved Week3 0.03932 0.1249 Inf 0.315
 Orco WingClip Week3 - WT WingRemoved Week3 -1.18334 0.1618 Inf -7.312
 Orco WingClip Week3 - Orco Single Week4 -0.30791 0.1364 Inf -2.257
 Orco WingClip Week3 - WT Single Week4 -1.53057 0.1700 Inf -9.002
 Orco WingClip Week3 - Orco WingClip Week4 -0.09503 0.0584 Inf -1.627
 Orco WingClip Week3 - WT WingClip Week4 -1.31768 0.1181 Inf -11.153
 Orco WingClip Week3 - Orco WingRemoved Week4 -0.05571 0.1379 Inf -0.404
 Orco WingClip Week3 - WT WingRemoved Week4 -1.27837 0.1719 Inf -7.435
 Orco WingClip Week3 - Orco Single Week5 -0.37789 0.1363 Inf -2.771
 Orco WingClip Week3 - WT Single Week5 -1.60054 0.1699 Inf -9.421
 Orco WingClip Week3 - Orco WingClip Week5 -0.16500 0.0585 Inf -2.823
 Orco WingClip Week3 - WT WingClip Week5 -1.38765 0.1181 Inf -11.752
 Orco WingClip Week3 - Orco WingRemoved Week5 -0.12568 0.1379 Inf -0.912
 Orco WingClip Week3 - WT WingRemoved Week5 -1.34834 0.1719 Inf -7.846
 WT WingClip Week3 - Orco WingRemoved Week3 1.26197 0.1618 Inf 7.800
 WT WingClip Week3 - WT WingRemoved Week3 0.03932 0.1249 Inf 0.315
 WT WingClip Week3 - Orco Single Week4 0.91474 0.1717 Inf 5.328
 WT WingClip Week3 - WT Single Week4 -0.30791 0.1364 Inf -2.257
 WT WingClip Week3 - Orco WingClip Week4 1.12763 0.1184 Inf 9.525
 WT WingClip Week3 - WT WingClip Week4 -0.09503 0.0584 Inf -1.627
 WT WingClip Week3 - Orco WingRemoved Week4 1.16695 0.1721 Inf 6.782
 WT WingClip Week3 - WT WingRemoved Week4 -0.05571 0.1379 Inf -0.404
 WT WingClip Week3 - Orco Single Week5 0.84477 0.1717 Inf 4.921
 WT WingClip Week3 - WT Single Week5 -0.37789 0.1363 Inf -2.771
 WT WingClip Week3 - Orco WingClip Week5 1.05766 0.1185 Inf 8.925
 WT WingClip Week3 - WT WingClip Week5 -0.16500 0.0585 Inf -2.823
 WT WingClip Week3 - Orco WingRemoved Week5 1.09698 0.1721 Inf 6.374
 WT WingClip Week3 - WT WingRemoved Week5 -0.12568 0.1379 Inf -0.912
 Orco WingRemoved Week3 - WT WingRemoved Week3 -1.22266 0.1028 Inf -11.889
 Orco WingRemoved Week3 - Orco Single Week4 -0.34723 0.1372 Inf -2.530
 Orco WingRemoved Week3 - WT Single Week4 -1.56989 0.1706 Inf -9.200
 Orco WingRemoved Week3 - Orco WingClip Week4 -0.13434 0.1379 Inf -0.974
 Orco WingRemoved Week3 - WT WingClip Week4 -1.35700 0.1720 Inf -7.891
 Orco WingRemoved Week3 - Orco WingRemoved Week4 -0.09503 0.0584 Inf -1.627
 Orco WingRemoved Week3 - WT WingRemoved Week4 -1.31768 0.1181 Inf -11.153
 Orco WingRemoved Week3 - Orco Single Week5 -0.41720 0.1372 Inf -3.041
 Orco WingRemoved Week3 - WT Single Week5 -1.63986 0.1705 Inf -9.615
 Orco WingRemoved Week3 - Orco WingClip Week5 -0.20431 0.1380 Inf -1.480
 Orco WingRemoved Week3 - WT WingClip Week5 -1.42697 0.1720 Inf -8.299
 Orco WingRemoved Week3 - Orco WingRemoved Week5 -0.16500 0.0585 Inf -2.823
 Orco WingRemoved Week3 - WT WingRemoved Week5 -1.38765 0.1181 Inf -11.752
 WT WingRemoved Week3 - Orco Single Week4 0.87543 0.1723 Inf 5.080
 WT WingRemoved Week3 - WT Single Week4 -0.34723 0.1372 Inf -2.530
 WT WingRemoved Week3 - Orco WingClip Week4 1.08832 0.1722 Inf 6.322
 WT WingRemoved Week3 - WT WingClip Week4 -0.13434 0.1379 Inf -0.974
 WT WingRemoved Week3 - Orco WingRemoved Week4 1.12763 0.1184 Inf 9.525
 WT WingRemoved Week3 - WT WingRemoved Week4 -0.09503 0.0584 Inf -1.627
 WT WingRemoved Week3 - Orco Single Week5 0.80545 0.1723 Inf 4.673
 WT WingRemoved Week3 - WT Single Week5 -0.41720 0.1372 Inf -3.041
 WT WingRemoved Week3 - Orco WingClip Week5 1.01834 0.1723 Inf 5.911
 WT WingRemoved Week3 - WT WingClip Week5 -0.20431 0.1380 Inf -1.480
 WT WingRemoved Week3 - Orco WingRemoved Week5 1.05766 0.1185 Inf 8.925
 WT WingRemoved Week3 - WT WingRemoved Week5 -0.16500 0.0585 Inf -2.823
 Orco Single Week4 - WT Single Week4 -1.22266 0.1028 Inf -11.889
 Orco Single Week4 - Orco WingClip Week4 0.21289 0.1233 Inf 1.727
 Orco Single Week4 - WT WingClip Week4 -1.00977 0.1613 Inf -6.258
 Orco Single Week4 - Orco WingRemoved Week4 0.25221 0.1241 Inf 2.032
 Orco Single Week4 - WT WingRemoved Week4 -0.97045 0.1620 Inf -5.990
 Orco Single Week4 - Orco Single Week5 -0.06997 0.0576 Inf -1.214
 Orco Single Week4 - WT Single Week5 -1.29263 0.1178 Inf -10.973
 Orco Single Week4 - Orco WingClip Week5 0.14292 0.1362 Inf 1.049
 Orco Single Week4 - WT WingClip Week5 -1.07974 0.1714 Inf -6.301
 Orco Single Week4 - Orco WingRemoved Week5 0.18223 0.1369 Inf 1.331
 Orco Single Week4 - WT WingRemoved Week5 -1.04042 0.1719 Inf -6.051
 WT Single Week4 - Orco WingClip Week4 1.43555 0.1598 Inf 8.985
 WT Single Week4 - WT WingClip Week4 0.21289 0.1233 Inf 1.727
 WT Single Week4 - Orco WingRemoved Week4 1.47486 0.1604 Inf 9.195
 WT Single Week4 - WT WingRemoved Week4 0.25221 0.1241 Inf 2.032
 WT Single Week4 - Orco Single Week5 1.15269 0.1180 Inf 9.770
 WT Single Week4 - WT Single Week5 -0.06997 0.0576 Inf -1.214
 WT Single Week4 - Orco WingClip Week5 1.36558 0.1700 Inf 8.033
 WT Single Week4 - WT WingClip Week5 0.14292 0.1362 Inf 1.049
 WT Single Week4 - Orco WingRemoved Week5 1.40489 0.1706 Inf 8.237
 WT Single Week4 - WT WingRemoved Week5 0.18223 0.1369 Inf 1.331
 Orco WingClip Week4 - WT WingClip Week4 -1.22266 0.1028 Inf -11.889
 Orco WingClip Week4 - Orco WingRemoved Week4 0.03932 0.1249 Inf 0.315
 Orco WingClip Week4 - WT WingRemoved Week4 -1.18334 0.1618 Inf -7.312
 Orco WingClip Week4 - Orco Single Week5 -0.28286 0.1360 Inf -2.080
 Orco WingClip Week4 - WT Single Week5 -1.50552 0.1697 Inf -8.872
 Orco WingClip Week4 - Orco WingClip Week5 -0.06997 0.0576 Inf -1.214
 Orco WingClip Week4 - WT WingClip Week5 -1.29263 0.1178 Inf -10.973
 Orco WingClip Week4 - Orco WingRemoved Week5 -0.03066 0.1375 Inf -0.223
 Orco WingClip Week4 - WT WingRemoved Week5 -1.25331 0.1717 Inf -7.300
 WT WingClip Week4 - Orco WingRemoved Week4 1.26197 0.1618 Inf 7.800
 WT WingClip Week4 - WT WingRemoved Week4 0.03932 0.1249 Inf 0.315
 WT WingClip Week4 - Orco Single Week5 0.93980 0.1713 Inf 5.486
 WT WingClip Week4 - WT Single Week5 -0.28286 0.1360 Inf -2.080
 WT WingClip Week4 - Orco WingClip Week5 1.15269 0.1180 Inf 9.770
 WT WingClip Week4 - WT WingClip Week5 -0.06997 0.0576 Inf -1.214
 WT WingClip Week4 - Orco WingRemoved Week5 1.19200 0.1718 Inf 6.939
 WT WingClip Week4 - WT WingRemoved Week5 -0.03066 0.1375 Inf -0.223
 Orco WingRemoved Week4 - WT WingRemoved Week4 -1.22266 0.1028 Inf -11.889
 Orco WingRemoved Week4 - Orco Single Week5 -0.32218 0.1368 Inf -2.355
 Orco WingRemoved Week4 - WT Single Week5 -1.54484 0.1703 Inf -9.070
 Orco WingRemoved Week4 - Orco WingClip Week5 -0.10929 0.1376 Inf -0.794
 Orco WingRemoved Week4 - WT WingClip Week5 -1.33195 0.1717 Inf -7.756
 Orco WingRemoved Week4 - Orco WingRemoved Week5 -0.06997 0.0576 Inf -1.214
 Orco WingRemoved Week4 - WT WingRemoved Week5 -1.29263 0.1178 Inf -10.973
 WT WingRemoved Week4 - Orco Single Week5 0.90048 0.1720 Inf 5.236
 WT WingRemoved Week4 - WT Single Week5 -0.32218 0.1368 Inf -2.355
 WT WingRemoved Week4 - Orco WingClip Week5 1.11337 0.1719 Inf 6.478
 WT WingRemoved Week4 - WT WingClip Week5 -0.10929 0.1376 Inf -0.794
 WT WingRemoved Week4 - Orco WingRemoved Week5 1.15269 0.1180 Inf 9.770
 WT WingRemoved Week4 - WT WingRemoved Week5 -0.06997 0.0576 Inf -1.214
 Orco Single Week5 - WT Single Week5 -1.22266 0.1028 Inf -11.889
 Orco Single Week5 - Orco WingClip Week5 0.21289 0.1233 Inf 1.727
 Orco Single Week5 - WT WingClip Week5 -1.00977 0.1613 Inf -6.258
 Orco Single Week5 - Orco WingRemoved Week5 0.25221 0.1241 Inf 2.032
 Orco Single Week5 - WT WingRemoved Week5 -0.97045 0.1620 Inf -5.990
 WT Single Week5 - Orco WingClip Week5 1.43555 0.1598 Inf 8.985
 WT Single Week5 - WT WingClip Week5 0.21289 0.1233 Inf 1.727
 WT Single Week5 - Orco WingRemoved Week5 1.47486 0.1604 Inf 9.195
 WT Single Week5 - WT WingRemoved Week5 0.25221 0.1241 Inf 2.032
 Orco WingClip Week5 - WT WingClip Week5 -1.22266 0.1028 Inf -11.889
 Orco WingClip Week5 - Orco WingRemoved Week5 0.03932 0.1249 Inf 0.315
 Orco WingClip Week5 - WT WingRemoved Week5 -1.18334 0.1618 Inf -7.312
 WT WingClip Week5 - Orco WingRemoved Week5 1.26197 0.1618 Inf 7.800
 WT WingClip Week5 - WT WingRemoved Week5 0.03932 0.1249 Inf 0.315
 Orco WingRemoved Week5 - WT WingRemoved Week5 -1.22266 0.1028 Inf -11.889
 p.value
 <.0001
 0.9976
 <.0001
 0.9743
 <.0001
 <.0001
 <.0001
 1.0000
 <.0001
 1.0000
 <.0001
 <.0001
 <.0001
 1.0000
 <.0001
 1.0000
 <.0001
 <.0001
 <.0001
 0.9941
 <.0001
 0.9997
 <.0001
 <.0001
 <.0001
 0.8802
 <.0001
 0.9746
 <.0001
 <.0001
 0.9976
 <.0001
 0.9743
 <.0001
 <.0001
 <.0001
 1.0000
 <.0001
 1.0000
 <.0001
 <.0001
 <.0001
 1.0000
 <.0001
 1.0000
 <.0001
 <.0001
 <.0001
 0.9941
 <.0001
 0.9997
 <.0001
 <.0001
 0.0001
 0.8802
 <.0001
 0.9746
 <.0001
 1.0000
 <.0001
 0.0109
 <.0001
 <.0001
 <.0001
 0.8899
 <.0001
 0.0093
 <.0001
 <.0001
 <.0001
 0.8687
 <.0001
 0.0004
 <.0001
 <.0001
 <.0001
 0.3604
 <.0001
 <.0001
 <.0001
 <.0001
 <.0001
 0.1011
 <.0001
 <.0001
 1.0000
 0.0524
 0.0109
 <.0001
 <.0001
 0.0001
 0.8899
 0.0609
 0.0093
 <.0001
 <.0001
 0.0001
 0.8687
 0.2849
 0.0004
 <.0001
 <.0001
 0.0018
 0.3604
 0.5982
 <.0001
 <.0001
 <.0001
 0.0101
 0.1011
 <.0001
 0.0034
 <.0001
 0.4696
 <.0001
 <.0001
 <.0001
 0.0029
 <.0001
 0.4337
 <.0001
 <.0001
 <.0001
 0.0001
 <.0001
 0.0769
 <.0001
 <.0001
 <.0001
 <.0001
 <.0001
 0.0128
 <.0001
 <.0001
 <.0001
 0.1096
 0.0034
 0.0008
 0.4696
 <.0001
 <.0001
 0.1246
 0.0029
 0.0010
 0.4337
 <.0001
 <.0001
 0.4544
 0.0001
 0.0113
 0.0769
 <.0001
 <.0001
 0.7766
 <.0001
 0.0512
 0.0128
 <.0001
 <.0001
 <.0001
 0.9976
 <.0001
 0.9743
 <.0001
 1.0000
 <.0001
 0.9998
 <.0001
 0.9958
 <.0001
 0.9969
 <.0001
 1.0000
 <.0001
 1.0000
 <.0001
 0.4165
 <.0001
 1.0000
 <.0001
 1.0000
 <.0001
 <.0001
 0.9976
 <.0001
 0.9743
 <.0001
 1.0000
 <.0001
 0.9998
 <.0001
 0.9958
 <.0001
 0.9969
 <.0001
 1.0000
 <.0001
 1.0000
 <.0001
 0.4165
 <.0001
 1.0000
 <.0001
 1.0000
 <.0001
 1.0000
 <.0001
 0.9993
 <.0001
 1.0000
 <.0001
 1.0000
 <.0001
 0.8910
 <.0001
 0.9969
 <.0001
 1.0000
 <.0001
 0.5256
 <.0001
 0.4165
 <.0001
 1.0000
 <.0001
 <.0001
 1.0000
 <.0001
 0.9993
 <.0001
 1.0000
 <.0001
 1.0000
 0.0001
 0.8910
 <.0001
 0.9969
 <.0001
 1.0000
 0.0004
 0.5256
 <.0001
 0.4165
 <.0001
 1.0000
 <.0001
 0.9904
 <.0001
 1.0000
 <.0001
 1.0000
 <.0001
 0.7211
 <.0001
 1.0000
 <.0001
 0.9969
 <.0001
 0.3156
 <.0001
 0.9997
 <.0001
 0.4165
 <.0001
 <.0001
 0.9904
 <.0001
 1.0000
 <.0001
 1.0000
 0.0002
 0.7211
 <.0001
 1.0000
 <.0001
 0.9969
 0.0014
 0.3156
 <.0001
 0.9997
 <.0001
 0.4165
 <.0001
 0.9976
 <.0001
 0.9743
 <.0001
 0.9991
 <.0001
 1.0000
 <.0001
 1.0000
 <.0001
 0.5266
 <.0001
 1.0000
 <.0001
 1.0000
 <.0001
 <.0001
 0.9976
 <.0001
 0.9743
 <.0001
 0.9991
 <.0001
 1.0000
 <.0001
 1.0000
 <.0001
 0.5266
 <.0001
 1.0000
 <.0001
 1.0000
 <.0001
 1.0000
 <.0001
 0.9132
 <.0001
 0.9991
 <.0001
 1.0000
 <.0001
 0.5694
 <.0001
 0.5266
 <.0001
 1.0000
 <.0001
 <.0001
 1.0000
 <.0001
 0.9132
 <.0001
 0.9991
 <.0001
 1.0000
 0.0003
 0.5694
 <.0001
 0.5266
 <.0001
 1.0000
 <.0001
 0.7599
 <.0001
 1.0000
 <.0001
 0.9991
 <.0001
 0.3536
 <.0001
 0.9998
 <.0001
 0.5266
 <.0001
 0.0002
 0.7599
 <.0001
 1.0000
 <.0001
 0.9991
 0.0012
 0.3536
 <.0001
 0.9998
 <.0001
 0.5266
 <.0001
 0.9976
 <.0001
 0.9743
 <.0001
 1.0000
 <.0001
 1.0000
 <.0001
 1.0000
 <.0001
 <.0001
 0.9976
 <.0001
 0.9743
 <.0001
 1.0000
 <.0001
 1.0000
 <.0001
 1.0000
 <.0001
 1.0000
 <.0001
 0.9655
 <.0001
 1.0000
 <.0001
 1.0000
 <.0001
 <.0001
 1.0000
 <.0001
 0.9655
 <.0001
 1.0000
 <.0001
 1.0000
 <.0001
 0.8684
 <.0001
 1.0000
 <.0001
 1.0000
 <.0001
 0.0001
 0.8684
 <.0001
 1.0000
 <.0001
 1.0000
 <.0001
 0.9976
 <.0001
 0.9743
 <.0001
 <.0001
 0.9976
 <.0001
 0.9743
 <.0001
 1.0000
 <.0001
 <.0001
 1.0000
 <.0001

Results are given on the log (not the response) scale.
P value adjustment: tukey method for comparing a family of 30 estimates

lsmeans(OrcoWalking_glmmTMB2, pairwise ~ Focal_Treatment + Rival_Treatment, adjust="tukey")

$lsmeans
 Focal_Treatment Rival_Treatment lsmean SE df asymp.LCL asymp.UCL
 Orco Single -0.103 0.1073 Inf -0.313 0.107
 WT Single 1.119 0.0980 Inf 0.927 1.312
 Orco WingClip -0.316 0.1064 Inf -0.525 -0.108
 WT WingClip 0.907 0.0984 Inf 0.714 1.099
 Orco WingRemoved -0.355 0.1081 Inf -0.567 -0.144
 WT WingRemoved 0.867 0.1002 Inf 0.671 1.064

Results are averaged over the levels of: Week
Results are given on the log (not the response) scale.
Confidence level used: 0.95

$contrasts
 contrast estimate SE df z.ratio p.value
 Orco Single - WT Single -1.2227 0.103 Inf -11.889 <.0001
 Orco Single - Orco WingClip 0.2129 0.123 Inf 1.727 0.5140
 Orco Single - WT WingClip -1.0098 0.161 Inf -6.258 <.0001
 Orco Single - Orco WingRemoved 0.2522 0.124 Inf 2.032 0.3242
 Orco Single - WT WingRemoved -0.9705 0.162 Inf -5.990 <.0001
 WT Single - Orco WingClip 1.4355 0.160 Inf 8.985 <.0001
 WT Single - WT WingClip 0.2129 0.123 Inf 1.727 0.5140
 WT Single - Orco WingRemoved 1.4749 0.160 Inf 9.195 <.0001
 WT Single - WT WingRemoved 0.2522 0.124 Inf 2.032 0.3242
 Orco WingClip - WT WingClip -1.2227 0.103 Inf -11.889 <.0001
 Orco WingClip - Orco WingRemoved 0.0393 0.125 Inf 0.315 0.9996
 Orco WingClip - WT WingRemoved -1.1833 0.162 Inf -7.312 <.0001
 WT WingClip - Orco WingRemoved 1.2620 0.162 Inf 7.800 <.0001
 WT WingClip - WT WingRemoved 0.0393 0.125 Inf 0.315 0.9996
 Orco WingRemoved - WT WingRemoved -1.2227 0.103 Inf -11.889 <.0001

Results are averaged over the levels of: Week
Results are given on the log (not the response) scale.
P value adjustment: tukey method for comparing a family of 6 estimates

lsmeans(OrcoWalking_glmmTMB2, pairwise ~ Focal_Treatment, adjust="tukey")

$lsmeans
 Focal_Treatment lsmean SE df asymp.LCL asymp.UCL
 Orco -0.258 0.0798 Inf -0.415 -0.102
 WT 0.964 0.0681 Inf 0.831 1.098

Results are averaged over the levels of: Rival_Treatment, Week
Results are given on the log (not the response) scale.
Confidence level used: 0.95

$contrasts
 contrast estimate SE df z.ratio p.value
 Orco - WT -1.22 0.103 Inf -11.889 <.0001

Results are averaged over the levels of: Rival_Treatment, Week
Results are given on the log (not the response) scale.

lsmeans(OrcoWalking_glmmTMB2, pairwise ~ Rival_Treatment, adjust="tukey")

$lsmeans
 Rival_Treatment lsmean SE df asymp.LCL asymp.UCL
 Single 0.508 0.0890 Inf 0.3338 0.682
 WingClip 0.295 0.0886 Inf 0.1215 0.469
 WingRemoved 0.256 0.0906 Inf 0.0783 0.434

Results are averaged over the levels of: Focal_Treatment, Week
Results are given on the log (not the response) scale.
Confidence level used: 0.95

$contrasts
 contrast estimate SE df z.ratio p.value
 Single - WingClip 0.2129 0.123 Inf 1.727 0.1952
 Single - WingRemoved 0.2522 0.124 Inf 2.032 0.1047
 WingClip - WingRemoved 0.0393 0.125 Inf 0.315 0.9469

Results are averaged over the levels of: Focal_Treatment, Week
Results are given on the log (not the response) scale.
P value adjustment: tukey method for comparing a family of 3 estimates

lsmeans(OrcoWalking_glmmTMB2, pairwise ~ Week, adjust="tukey")

$lsmeans
 Week lsmean SE df asymp.LCL asymp.UCL
 1 0.00575 0.0685 Inf -0.128 0.140
 2 0.36969 0.0644 Inf 0.244 0.496
 3 0.37665 0.0652 Inf 0.249 0.504
 4 0.47168 0.0651 Inf 0.344 0.599
 5 0.54165 0.0653 Inf 0.414 0.670

Results are averaged over the levels of: Focal_Treatment, Rival_Treatment
Results are given on the log (not the response) scale.
Confidence level used: 0.95

$contrasts
 contrast estimate SE df z.ratio p.value
 Week1 - Week2 -0.36394 0.0631 Inf -5.769 <.0001
 Week1 - Week3 -0.37091 0.0638 Inf -5.816 <.0001
 Week1 - Week4 -0.46593 0.0636 Inf -7.328 <.0001
 Week1 - Week5 -0.53590 0.0636 Inf -8.426 <.0001
 Week2 - Week3 -0.00696 0.0583 Inf -0.119 1.0000
 Week2 - Week4 -0.10199 0.0581 Inf -1.755 0.4002
 Week2 - Week5 -0.17196 0.0581 Inf -2.958 0.0257
 Week3 - Week4 -0.09503 0.0584 Inf -1.627 0.4800
 Week3 - Week5 -0.16500 0.0585 Inf -2.823 0.0383
 Week4 - Week5 -0.06997 0.0576 Inf -1.214 0.7432

Results are averaged over the levels of: Focal_Treatment, Rival_Treatment
Results are given on the log (not the response) scale.
P value adjustment: tukey method for comparing a family of 5 estimates

##Orco Walking Line
Orco_Walking$PercentSuccess <- Orco_Walking$Success / 10
Orco_Walking_subset <- subset(Orco_Walking, PercentSuccess > 0)
Orco_Walking_subset$Rival_Treatment <- factor(Orco_Walking_subset$Rival_Treatment, levels = c("Single", "WingClip", "WingRemoved"))
Orco_Walking_subset$Focal_Treatment <- factor(Orco_Walking_subset$Focal_Treatment, levels = c("WT", "Orco"))
Orco_Walking_subset$Interaction <- interaction(Orco_Walking_subset$Focal_Treatment, Orco_Walking_subset$Rival_Treatment)
Orco_Walking_subset$Interaction <- factor(Orco_Walking_subset$Interaction, levels = c("WT.Single", "WT.WingClip", "WT.WingRemoved", "Orco.Single", "Orco.WingClip", "Orco.WingRemoved"))

Orco_Walking_Summary <- summarySE(Orco_Walking_subset,
 measurevar = "PercentSuccess",
 groupvars = c("Week", "Interaction", "Focal_Treatment", "Rival_Treatment"))

Orco_Walking_Line <- ggplot(Orco_Walking_Summary) +
 aes(
 x = Week,
 y = PercentSuccess,
 colour = Interaction,
 group = Interaction
 ) +
 scale_colour_manual(name = "Treatment",
 labels = c("Wildtype kept alone",
 "Wildtype kept with Wing Clipped Rival",
 "Wildtype kept with Wing Removed Rival",
 "Orco2 kept alone",
 "Orco2 kept with Wing Clipped Rival",
 "Orco2 kept with Wing Removed Rival"),
 values = c("black",
 "grey70",
 "grey42",
 "black",
 "grey70",
 "grey42")) +
 geom_line(aes(linetype = Interaction), size = 3) +
 geom_point() +
 geom_errorbar(aes(ymin=PercentSuccess-se, ymax=PercentSuccess+se), width=0.2, size = 1.5) +
 scale_linetype_manual(name = "Treatment",
 labels = c("Wildtype kept alone",
 "Wildtype kept with Wing Clipped Rival",
 "Wildtype kept with Wing Removed Rival",
 "Orco2 kept alone",
 "Orco2 kept with Wing Clipped Rival",
 "Orco2 kept with Wing Removed Rival"),
 values = c("solid", "solid", "solid", "dotdash", "dotdash", "dotdash")) +
 labs(
 x = "Week post Eclosion",
 y = "Proportion of Time Spent Walking"
 ) +
 ylim(0, 1.05) +
 theme_bw() +
 guides(colour = guide_legend(override.aes = list(linetype = c("solid", "solid", "solid", "dotdash", "dotdash", "dotdash"))))

Orco_Walking_Line_Plot <- Orco_Walking_Line + theme_bw() + theme(panel.grid.major = element_blank(), panel.grid.minor = element_blank()) +
 theme(axis.text=element_text(size=55, face="bold", colour="black"),
 axis.title=element_text(size=75, face="bold", colour="black"),
 legend.text=element_text(size=55, face="bold", colour="black"),
 legend.title=element_text(size=75, face="bold", colour="black"),
 strip.text=element_text(size=75, face="bold", colour="black")) + theme(axis.line = element_line(size = 5), panel.border = element_rect(size = 5)) + theme( axis.ticks.length = unit(0.5, "cm"), axis.ticks = element_line(size = 5))
Orco_Walking_Line_Plot


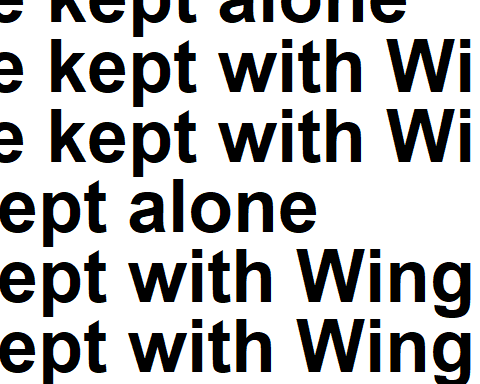


###Orco Grooming
##Orco Grooming Model
Orco_Grooming$Week <- as.factor(Orco_Grooming$Week)
OrcoGrooming_glmmTMB1 <- glmmTMB(Success ~ Focal_Treatment * Rival_Treatment + Week + (1|FlyID), family=poisson(link = "log"), data = Orco_Grooming)
OrcoGrooming_glmmTMB2 <- glmmTMB(Success ~ Focal_Treatment + Rival_Treatment + Week + (1|FlyID), family=poisson(link = "log"), data = Orco_Grooming)
anova(OrcoGrooming_glmmTMB1, OrcoGrooming_glmmTMB2, test="Chi")

Data: Orco_Grooming
Models:
OrcoGrooming_glmmTMB2: Success ~ Focal_Treatment + Rival_Treatment + Week + (1 | FlyID), zi=~0, disp=~1
OrcoGrooming_glmmTMB1: Success ~ Focal_Treatment * Rival_Treatment + Week + (1 | FlyID), zi=~0, disp=~1
 Df AIC BIC logLik deviance Chisq Chi Df
OrcoGrooming_glmmTMB2 9 838.61 885.28 -410.31 820.61
OrcoGrooming_glmmTMB1 11 838.36 895.39 -408.18 816.36 4.2556 2
 Pr(>Chisq)
OrcoGrooming_glmmTMB2
OrcoGrooming_glmmTMB1 0.1191

OrcoGrooming_glmmTMB3 <- glmmTMB(Success ~ Rival_Treatment + Week + (1|FlyID), family=poisson(link = "log"), data = Orco_Grooming)
anova(OrcoGrooming_glmmTMB2, OrcoGrooming_glmmTMB3, test="Chi")

Data: Orco_Grooming
Models:
OrcoGrooming_glmmTMB3: Success ~ Rival_Treatment + Week + (1 | FlyID), zi=~0, disp=~1
OrcoGrooming_glmmTMB2: Success ~ Focal_Treatment + Rival_Treatment + Week + (1 | FlyID), zi=~0, disp=~1
 Df AIC BIC logLik deviance Chisq Chi Df
OrcoGrooming_glmmTMB3 8 838.27 879.75 -411.13 822.27
OrcoGrooming_glmmTMB2 9 838.61 885.28 -410.31 820.61 1.6545 1
 Pr(>Chisq)
OrcoGrooming_glmmTMB3
OrcoGrooming_glmmTMB2 0.1984

OrcoGrooming_glmmTMB4 <- glmmTMB(Success ~ Focal_Treatment + Week + (1|FlyID), family=poisson(link = "log"), data = Orco_Grooming)
anova(OrcoGrooming_glmmTMB2, OrcoGrooming_glmmTMB4, test="Chi")

Data: Orco_Grooming
Models:
OrcoGrooming_glmmTMB4: Success ~ Focal_Treatment + Week + (1 | FlyID), zi=~0, disp=~1
OrcoGrooming_glmmTMB2: Success ~ Focal_Treatment + Rival_Treatment + Week + (1 | FlyID), zi=~0, disp=~1
 Df AIC BIC logLik deviance Chisq Chi Df
OrcoGrooming_glmmTMB4 7 845.58 881.87 -415.79 831.58
OrcoGrooming_glmmTMB2 9 838.61 885.28 -410.31 820.61 10.964 2
 Pr(>Chisq)
OrcoGrooming_glmmTMB4
OrcoGrooming_glmmTMB2 0.004161 **
---
Signif. codes: 0 '***' 0.001 '**' 0.01 '*' 0.05 '.' 0.1 ' ' 1

OrcoGrooming_glmmTMB_null <- glmmTMB(Success ~ (1|FlyID), family=poisson(link = "log"), data = Orco_Grooming)
anova(OrcoGrooming_glmmTMB2, OrcoGrooming_glmmTMB_null, test="Chi")

Data: Orco_Grooming
Models:
OrcoGrooming_glmmTMB_null: Success ~ (1 | FlyID), zi=~0, disp=~1
OrcoGrooming_glmmTMB2: Success ~ Focal_Treatment + Rival_Treatment + Week + (1 | FlyID), zi=~0, disp=~1
 Df AIC BIC logLik deviance Chisq Chi Df
OrcoGrooming_glmmTMB_null 2 846.38 856.74 -421.19 842.38
OrcoGrooming_glmmTMB2 9 838.61 885.28 -410.31 820.61 21.762 7
 Pr(>Chisq)
OrcoGrooming_glmmTMB_null
OrcoGrooming_glmmTMB2 0.002792 **
---
Signif. codes: 0 '***' 0.001 '**' 0.01 '*' 0.05 '.' 0.1 ' ' 1

lsmeans(OrcoGrooming_glmmTMB2, pairwise ~ Focal_Treatment * Rival_Treatment * Week, adjust="tukey")

$lsmeans
 Focal_Treatment Rival_Treatment Week lsmean SE df asymp.LCL asymp.UCL
 Orco Single 1 -2.10 0.259 Inf -2.61 -1.59
 WT Single 1 -1.85 0.254 Inf -2.35 -1.35
 Orco WingClip 1 -2.83 0.295 Inf -3.41 -2.25
 WT WingClip 1 -2.58 0.288 Inf -3.14 -2.01
 Orco WingRemoved 1 -2.66 0.285 Inf -3.22 -2.10
 WT WingRemoved 1 -2.41 0.279 Inf -2.96 -1.86
 Orco Single 2 -2.73 0.313 Inf -3.34 -2.11
 WT Single 2 -2.48 0.310 Inf -3.09 -1.87
 Orco WingClip 2 -3.46 0.343 Inf -4.13 -2.78
 WT WingClip 2 -3.21 0.338 Inf -3.87 -2.54
 Orco WingRemoved 2 -3.29 0.335 Inf -3.94 -2.63
 WT WingRemoved 2 -3.04 0.331 Inf -3.69 -2.39
 Orco Single 3 -2.01 0.259 Inf -2.52 -1.50
 WT Single 3 -1.76 0.257 Inf -2.27 -1.26
 Orco WingClip 3 -2.74 0.294 Inf -3.32 -2.16
 WT WingClip 3 -2.49 0.290 Inf -3.06 -1.92
 Orco WingRemoved 3 -2.57 0.285 Inf -3.13 -2.02
 WT WingRemoved 3 -2.32 0.282 Inf -2.88 -1.77
 Orco Single 4 -2.54 0.307 Inf -3.14 -1.94
 WT Single 4 -2.29 0.305 Inf -2.89 -1.70
 Orco WingClip 4 -3.27 0.337 Inf -3.93 -2.61
 WT WingClip 4 -3.02 0.333 Inf -3.67 -2.37
 Orco WingRemoved 4 -3.10 0.329 Inf -3.75 -2.46
 WT WingRemoved 4 -2.85 0.326 Inf -3.49 -2.21
 Orco Single 5 -2.05 0.273 Inf -2.58 -1.51
 WT Single 5 -1.80 0.270 Inf -2.33 -1.27
 Orco WingClip 5 -2.77 0.307 Inf -3.38 -2.17
 WT WingClip 5 -2.52 0.303 Inf -3.12 -1.93
 Orco WingRemoved 5 -2.61 0.298 Inf -3.19 -2.02
 WT WingRemoved 5 -2.36 0.295 Inf -2.94 -1.78

Results are given on the log (not the response) scale.
Confidence level used: 0.95

$contrasts
 contrast estimate SE df z.ratio
 Orco Single Week1 - WT Single Week1 -0.24888 0.193 Inf -1.290
 Orco Single Week1 - Orco WingClip Week1 0.72715 0.238 Inf 3.051
 Orco Single Week1 - WT WingClip Week1 0.47827 0.304 Inf 1.573
 Orco Single Week1 - Orco WingRemoved Week1 0.56020 0.229 Inf 2.442
 Orco Single Week1 - WT WingRemoved Week1 0.31132 0.299 Inf 1.040
 Orco Single Week1 - Orco Single Week2 0.62839 0.306 Inf 2.051
 Orco Single Week1 - WT Single Week2 0.37951 0.363 Inf 1.046
 Orco Single Week1 - Orco WingClip Week2 1.35554 0.388 Inf 3.497
 Orco Single Week1 - WT WingClip Week2 1.10666 0.432 Inf 2.562
 Orco Single Week1 - Orco WingRemoved Week2 1.18858 0.383 Inf 3.105
 Orco Single Week1 - WT WingRemoved Week2 0.93970 0.429 Inf 2.190
 Orco Single Week1 - Orco Single Week3 -0.08699 0.252 Inf -0.345
 Orco Single Week1 - WT Single Week3 -0.33587 0.320 Inf -1.050
 Orco Single Week1 - Orco WingClip Week3 0.64017 0.346 Inf 1.848
 Orco Single Week1 - WT WingClip Week3 0.39129 0.396 Inf 0.987
 Orco Single Week1 - Orco WingRemoved Week3 0.47321 0.341 Inf 1.388
 Orco Single Week1 - WT WingRemoved Week3 0.22433 0.393 Inf 0.570
 Orco Single Week1 - Orco Single Week4 0.44271 0.301 Inf 1.473
 Orco Single Week1 - WT Single Week4 0.19383 0.359 Inf 0.540
 Orco Single Week1 - Orco WingClip Week4 1.16986 0.383 Inf 3.054
 Orco Single Week1 - WT WingClip Week4 0.92098 0.429 Inf 2.148
 Orco Single Week1 - Orco WingRemoved Week4 1.00291 0.378 Inf 2.653
 Orco Single Week1 - WT WingRemoved Week4 0.75403 0.426 Inf 1.771
 Orco Single Week1 - Orco Single Week5 -0.05385 0.265 Inf -0.204
 Orco Single Week1 - WT Single Week5 -0.30273 0.329 Inf -0.919
 Orco Single Week1 - Orco WingClip Week5 0.67331 0.356 Inf 1.890
 Orco Single Week1 - WT WingClip Week5 0.42443 0.405 Inf 1.048
 Orco Single Week1 - Orco WingRemoved Week5 0.50635 0.351 Inf 1.444
 Orco Single Week1 - WT WingRemoved Week5 0.25747 0.402 Inf 0.641
 WT Single Week1 - Orco WingClip Week1 0.97603 0.309 Inf 3.156
 WT Single Week1 - WT WingClip Week1 0.72715 0.238 Inf 3.051
 WT Single Week1 - Orco WingRemoved Week1 0.80908 0.300 Inf 2.694
 WT Single Week1 - WT WingRemoved Week1 0.56020 0.229 Inf 2.442
 WT Single Week1 - Orco Single Week2 0.87727 0.361 Inf 2.429
 WT Single Week1 - WT Single Week2 0.62839 0.306 Inf 2.051
 WT Single Week1 - Orco WingClip Week2 1.60442 0.434 Inf 3.696
 WT Single Week1 - WT WingClip Week2 1.35554 0.388 Inf 3.497
 WT Single Week1 - Orco WingRemoved Week2 1.43746 0.428 Inf 3.357
 WT Single Week1 - WT WingRemoved Week2 1.18858 0.383 Inf 3.105
 WT Single Week1 - Orco Single Week3 0.16189 0.315 Inf 0.513
 WT Single Week1 - WT Single Week3 -0.08699 0.252 Inf -0.345
 WT Single Week1 - Orco WingClip Week3 0.88905 0.397 Inf 2.241
 WT Single Week1 - WT WingClip Week3 0.64017 0.346 Inf 1.848
 WT Single Week1 - Orco WingRemoved Week3 0.72209 0.390 Inf 1.850
 WT Single Week1 - WT WingRemoved Week3 0.47321 0.341 Inf 1.388
 WT Single Week1 - Orco Single Week4 0.69159 0.355 Inf 1.947
 WT Single Week1 - WT Single Week4 0.44271 0.301 Inf 1.473
 WT Single Week1 - Orco WingClip Week4 1.41874 0.429 Inf 3.306
 WT Single Week1 - WT WingClip Week4 1.16986 0.383 Inf 3.054
 WT Single Week1 - Orco WingRemoved Week4 1.25179 0.423 Inf 2.959
 WT Single Week1 - WT WingRemoved Week4 1.00291 0.378 Inf 2.653
 WT Single Week1 - Orco Single Week5 0.19503 0.326 Inf 0.599
 WT Single Week1 - WT Single Week5 -0.05385 0.265 Inf -0.204
 WT Single Week1 - Orco WingClip Week5 0.92219 0.406 Inf 2.274
 WT Single Week1 - WT WingClip Week5 0.67331 0.356 Inf 1.890
 WT Single Week1 - Orco WingRemoved Week5 0.75523 0.399 Inf 1.892
 WT Single Week1 - WT WingRemoved Week5 0.50635 0.351 Inf 1.444
 Orco WingClip Week1 - WT WingClip Week1 -0.24888 0.193 Inf -1.290
 Orco WingClip Week1 - Orco WingRemoved Week1 -0.16696 0.265 Inf -0.629
 Orco WingClip Week1 - WT WingRemoved Week1 -0.41584 0.330 Inf -1.259
 Orco WingClip Week1 - Orco Single Week2 -0.09877 0.389 Inf -0.254
 Orco WingClip Week1 - WT Single Week2 -0.34765 0.436 Inf -0.797
 Orco WingClip Week1 - Orco WingClip Week2 0.62839 0.306 Inf 2.051
 Orco WingClip Week1 - WT WingClip Week2 0.37951 0.363 Inf 1.046
 Orco WingClip Week1 - Orco WingRemoved Week2 0.46143 0.406 Inf 1.137
 Orco WingClip Week1 - WT WingRemoved Week2 0.21255 0.452 Inf 0.471
 Orco WingClip Week1 - Orco Single Week3 -0.81414 0.348 Inf -2.341
 Orco WingClip Week1 - WT Single Week3 -1.06302 0.402 Inf -2.647
 Orco WingClip Week1 - Orco WingClip Week3 -0.08699 0.252 Inf -0.345
 Orco WingClip Week1 - WT WingClip Week3 -0.33587 0.320 Inf -1.050
 Orco WingClip Week1 - Orco WingRemoved Week3 -0.25394 0.367 Inf -0.692
 Orco WingClip Week1 - WT WingRemoved Week3 -0.50282 0.418 Inf -1.203
 Orco WingClip Week1 - Orco Single Week4 -0.28445 0.384 Inf -0.741
 Orco WingClip Week1 - WT Single Week4 -0.53333 0.433 Inf -1.231
 Orco WingClip Week1 - Orco WingClip Week4 0.44271 0.301 Inf 1.473
 Orco WingClip Week1 - WT WingClip Week4 0.19383 0.359 Inf 0.540
 Orco WingClip Week1 - Orco WingRemoved Week4 0.27575 0.401 Inf 0.687
 Orco WingClip Week1 - WT WingRemoved Week4 0.02687 0.448 Inf 0.060
 Orco WingClip Week1 - Orco Single Week5 -0.78100 0.356 Inf -2.194
 Orco WingClip Week1 - WT Single Week5 -1.02988 0.408 Inf -2.522
 Orco WingClip Week1 - Orco WingClip Week5 -0.05385 0.265 Inf -0.204
 Orco WingClip Week1 - WT WingClip Week5 -0.30273 0.329 Inf -0.919
 Orco WingClip Week1 - Orco WingRemoved Week5 -0.22080 0.375 Inf -0.589
 Orco WingClip Week1 - WT WingRemoved Week5 -0.46968 0.425 Inf -1.105
 WT WingClip Week1 - Orco WingRemoved Week1 0.08192 0.326 Inf 0.251
 WT WingClip Week1 - WT WingRemoved Week1 -0.16696 0.265 Inf -0.629
 WT WingClip Week1 - Orco Single Week2 0.15011 0.431 Inf 0.348
 WT WingClip Week1 - WT Single Week2 -0.09877 0.389 Inf -0.254
 WT WingClip Week1 - Orco WingClip Week2 0.87727 0.361 Inf 2.429
 WT WingClip Week1 - WT WingClip Week2 0.62839 0.306 Inf 2.051
 WT WingClip Week1 - Orco WingRemoved Week2 0.71031 0.447 Inf 1.588
 WT WingClip Week1 - WT WingRemoved Week2 0.46143 0.406 Inf 1.137
 WT WingClip Week1 - Orco Single Week3 -0.56526 0.394 Inf -1.435
 WT WingClip Week1 - WT Single Week3 -0.81414 0.348 Inf -2.341
 WT WingClip Week1 - Orco WingClip Week3 0.16189 0.315 Inf 0.513
 WT WingClip Week1 - WT WingClip Week3 -0.08699 0.252 Inf -0.345
 WT WingClip Week1 - Orco WingRemoved Week3 -0.00506 0.411 Inf -0.012
 WT WingClip Week1 - WT WingRemoved Week3 -0.25394 0.367 Inf -0.692
 WT WingClip Week1 - Orco Single Week4 -0.03557 0.426 Inf -0.083
 WT WingClip Week1 - WT Single Week4 -0.28445 0.384 Inf -0.741
 WT WingClip Week1 - Orco WingClip Week4 0.69159 0.355 Inf 1.947
 WT WingClip Week1 - WT WingClip Week4 0.44271 0.301 Inf 1.473
 WT WingClip Week1 - Orco WingRemoved Week4 0.52463 0.442 Inf 1.186
 WT WingClip Week1 - WT WingRemoved Week4 0.27575 0.401 Inf 0.687
 WT WingClip Week1 - Orco Single Week5 -0.53212 0.401 Inf -1.326
 WT WingClip Week1 - WT Single Week5 -0.78100 0.356 Inf -2.194
 WT WingClip Week1 - Orco WingClip Week5 0.19503 0.326 Inf 0.599
 WT WingClip Week1 - WT WingClip Week5 -0.05385 0.265 Inf -0.204
 WT WingClip Week1 - Orco WingRemoved Week5 0.02808 0.419 Inf 0.067
 WT WingClip Week1 - WT WingRemoved Week5 -0.22080 0.375 Inf -0.589
 Orco WingRemoved Week1 - WT WingRemoved Week1 -0.24888 0.193 Inf -1.290
 Orco WingRemoved Week1 - Orco Single Week2 0.06819 0.383 Inf 0.178
 Orco WingRemoved Week1 - WT Single Week2 -0.18069 0.430 Inf -0.421
 Orco WingRemoved Week1 - Orco WingClip Week2 0.79534 0.405 Inf 1.964
 Orco WingRemoved Week1 - WT WingClip Week2 0.54646 0.448 Inf 1.220
 Orco WingRemoved Week1 - Orco WingRemoved Week2 0.62839 0.306 Inf 2.051
 Orco WingRemoved Week1 - WT WingRemoved Week2 0.37951 0.363 Inf 1.046
 Orco WingRemoved Week1 - Orco Single Week3 -0.64719 0.341 Inf -1.898
 Orco WingRemoved Week1 - WT Single Week3 -0.89607 0.394 Inf -2.274
 Orco WingRemoved Week1 - Orco WingClip Week3 0.07997 0.366 Inf 0.219
 Orco WingRemoved Week1 - WT WingClip Week3 -0.16891 0.414 Inf -0.408
 Orco WingRemoved Week1 - Orco WingRemoved Week3 -0.08699 0.252 Inf -0.345
 Orco WingRemoved Week1 - WT WingRemoved Week3 -0.33587 0.320 Inf -1.050
 Orco WingRemoved Week1 - Orco Single Week4 -0.11749 0.378 Inf -0.311
 Orco WingRemoved Week1 - WT Single Week4 -0.36637 0.427 Inf -0.859
 Orco WingRemoved Week1 - Orco WingClip Week4 0.60966 0.401 Inf 1.522
 Orco WingRemoved Week1 - WT WingClip Week4 0.36078 0.445 Inf 0.811
 Orco WingRemoved Week1 - Orco WingRemoved Week4 0.44271 0.301 Inf 1.473
 Orco WingRemoved Week1 - WT WingRemoved Week4 0.19383 0.359 Inf 0.540
 Orco WingRemoved Week1 - Orco Single Week5 -0.61405 0.350 Inf -1.756
 Orco WingRemoved Week1 - WT Single Week5 -0.86293 0.401 Inf -2.150
 Orco WingRemoved Week1 - Orco WingClip Week5 0.11311 0.374 Inf 0.302
 Orco WingRemoved Week1 - WT WingClip Week5 -0.13577 0.421 Inf -0.322
 Orco WingRemoved Week1 - Orco WingRemoved Week5 -0.05385 0.265 Inf -0.204
 Orco WingRemoved Week1 - WT WingRemoved Week5 -0.30273 0.329 Inf -0.919
 WT WingRemoved Week1 - Orco Single Week2 0.31707 0.427 Inf 0.742
 WT WingRemoved Week1 - WT Single Week2 0.06819 0.383 Inf 0.178
 WT WingRemoved Week1 - Orco WingClip Week2 1.04422 0.449 Inf 2.325
 WT WingRemoved Week1 - WT WingClip Week2 0.79534 0.405 Inf 1.964
 WT WingRemoved Week1 - Orco WingRemoved Week2 0.87727 0.361 Inf 2.429
 WT WingRemoved Week1 - WT WingRemoved Week2 0.62839 0.306 Inf 2.051
 WT WingRemoved Week1 - Orco Single Week3 -0.39831 0.390 Inf -1.022
 WT WingRemoved Week1 - WT Single Week3 -0.64719 0.341 Inf -1.898
 WT WingRemoved Week1 - Orco WingClip Week3 0.32885 0.413 Inf 0.796
 WT WingRemoved Week1 - WT WingClip Week3 0.07997 0.366 Inf 0.219
 WT WingRemoved Week1 - Orco WingRemoved Week3 0.16189 0.315 Inf 0.513
 WT WingRemoved Week1 - WT WingRemoved Week3 -0.08699 0.252 Inf -0.345
 WT WingRemoved Week1 - Orco Single Week4 0.13139 0.423 Inf 0.311
 WT WingRemoved Week1 - WT Single Week4 -0.11749 0.378 Inf -0.311
 WT WingRemoved Week1 - Orco WingClip Week4 0.85854 0.444 Inf 1.932
 WT WingRemoved Week1 - WT WingClip Week4 0.60966 0.401 Inf 1.522
 WT WingRemoved Week1 - Orco WingRemoved Week4 0.69159 0.355 Inf 1.947
 WT WingRemoved Week1 - WT WingRemoved Week4 0.44271 0.301 Inf 1.473
 WT WingRemoved Week1 - Orco Single Week5 -0.36517 0.397 Inf -0.919
 WT WingRemoved Week1 - WT Single Week5 -0.61405 0.350 Inf -1.756
 WT WingRemoved Week1 - Orco WingClip Week5 0.36199 0.421 Inf 0.859
 WT WingRemoved Week1 - WT WingClip Week5 0.11311 0.374 Inf 0.302
 WT WingRemoved Week1 - Orco WingRemoved Week5 0.19503 0.326 Inf 0.599
 WT WingRemoved Week1 - WT WingRemoved Week5 -0.05385 0.265 Inf -0.204
 Orco Single Week2 - WT Single Week2 -0.24888 0.193 Inf -1.290
 Orco Single Week2 - Orco WingClip Week2 0.72715 0.238 Inf 3.051
 Orco Single Week2 - WT WingClip Week2 0.47827 0.304 Inf 1.573
 Orco Single Week2 - Orco WingRemoved Week2 0.56020 0.229 Inf 2.442
 Orco Single Week2 - WT WingRemoved Week2 0.31132 0.299 Inf 1.040
 Orco Single Week2 - Orco Single Week3 -0.71537 0.308 Inf -2.323
 Orco Single Week2 - WT Single Week3 -0.96425 0.365 Inf -2.645
 Orco Single Week2 - Orco WingClip Week3 0.01178 0.389 Inf 0.030
 Orco Single Week2 - WT WingClip Week3 -0.23710 0.434 Inf -0.547
 Orco Single Week2 - Orco WingRemoved Week3 -0.15518 0.384 Inf -0.404
 Orco Single Week2 - WT WingRemoved Week3 -0.40406 0.430 Inf -0.939
 Orco Single Week2 - Orco Single Week4 -0.18568 0.349 Inf -0.533
 Orco Single Week2 - WT Single Week4 -0.43456 0.399 Inf -1.088
 Orco Single Week2 - Orco WingClip Week4 0.54148 0.422 Inf 1.283
 Orco Single Week2 - WT WingClip Week4 0.29260 0.463 Inf 0.632
 Orco Single Week2 - Orco WingRemoved Week4 0.37452 0.417 Inf 0.898
 Orco Single Week2 - WT WingRemoved Week4 0.12564 0.460 Inf 0.273
 Orco Single Week2 - Orco Single Week5 -0.68223 0.318 Inf -2.145
 Orco Single Week2 - WT Single Week5 -0.93111 0.373 Inf -2.497
 Orco Single Week2 - Orco WingClip Week5 0.04492 0.398 Inf 0.113
 Orco Single Week2 - WT WingClip Week5 -0.20396 0.441 Inf -0.462
 Orco Single Week2 - Orco WingRemoved Week5 -0.12203 0.393 Inf -0.311
 Orco Single Week2 - WT WingRemoved Week5 -0.37091 0.438 Inf -0.847
 WT Single Week2 - Orco WingClip Week2 0.97603 0.309 Inf 3.156
 WT Single Week2 - WT WingClip Week2 0.72715 0.238 Inf 3.051
 WT Single Week2 - Orco WingRemoved Week2 0.80908 0.300 Inf 2.694
 WT Single Week2 - WT WingRemoved Week2 0.56020 0.229 Inf 2.442
 WT Single Week2 - Orco Single Week3 -0.46649 0.362 Inf -1.288
 WT Single Week2 - WT Single Week3 -0.71537 0.308 Inf -2.323
 WT Single Week2 - Orco WingClip Week3 0.26066 0.435 Inf 0.599
 WT Single Week2 - WT WingClip Week3 0.01178 0.389 Inf 0.030
 WT Single Week2 - Orco WingRemoved Week3 0.09370 0.429 Inf 0.218
 WT Single Week2 - WT WingRemoved Week3 -0.15518 0.384 Inf -0.404
 WT Single Week2 - Orco Single Week4 0.06320 0.397 Inf 0.159
 WT Single Week2 - WT Single Week4 -0.18568 0.349 Inf -0.533
 WT Single Week2 - Orco WingClip Week4 0.79036 0.465 Inf 1.699
 WT Single Week2 - WT WingClip Week4 0.54148 0.422 Inf 1.283
 WT Single Week2 - Orco WingRemoved Week4 0.62340 0.459 Inf 1.358
 WT Single Week2 - WT WingRemoved Week4 0.37452 0.417 Inf 0.898
 WT Single Week2 - Orco Single Week5 -0.43335 0.371 Inf -1.167
 WT Single Week2 - WT Single Week5 -0.68223 0.318 Inf -2.145
 WT Single Week2 - Orco WingClip Week5 0.29380 0.443 Inf 0.663
 WT Single Week2 - WT WingClip Week5 0.04492 0.398 Inf 0.113
 WT Single Week2 - Orco WingRemoved Week5 0.12685 0.437 Inf 0.290
 WT Single Week2 - WT WingRemoved Week5 -0.12203 0.393 Inf -0.311
 Orco WingClip Week2 - WT WingClip Week2 -0.24888 0.193 Inf -1.290
 Orco WingClip Week2 - Orco WingRemoved Week2 -0.16696 0.265 Inf -0.629
 Orco WingClip Week2 - WT WingRemoved Week2 -0.41584 0.330 Inf -1.259
 Orco WingClip Week2 - Orco Single Week3 -1.44253 0.390 Inf -3.703
 Orco WingClip Week2 - WT Single Week3 -1.69141 0.437 Inf -3.866
 Orco WingClip Week2 - Orco WingClip Week3 -0.71537 0.308 Inf -2.323
 Orco WingClip Week2 - WT WingClip Week3 -0.96425 0.365 Inf -2.645
 Orco WingClip Week2 - Orco WingRemoved Week3 -0.88233 0.407 Inf -2.170
 Orco WingClip Week2 - WT WingRemoved Week3 -1.13121 0.452 Inf -2.500
 Orco WingClip Week2 - Orco Single Week4 -0.91283 0.422 Inf -2.162
 Orco WingClip Week2 - WT Single Week4 -1.16171 0.467 Inf -2.489
 Orco WingClip Week2 - Orco WingClip Week4 -0.18568 0.349 Inf -0.533
 Orco WingClip Week2 - WT WingClip Week4 -0.43456 0.399 Inf -1.088
 Orco WingClip Week2 - Orco WingRemoved Week4 -0.35263 0.438 Inf -0.805
 Orco WingClip Week2 - WT WingRemoved Week4 -0.60151 0.481 Inf -1.251
 Orco WingClip Week2 - Orco Single Week5 -1.40939 0.397 Inf -3.551
 Orco WingClip Week2 - WT Single Week5 -1.65827 0.444 Inf -3.737
 Orco WingClip Week2 - Orco WingClip Week5 -0.68223 0.318 Inf -2.145
 Orco WingClip Week2 - WT WingClip Week5 -0.93111 0.373 Inf -2.497
 Orco WingClip Week2 - Orco WingRemoved Week5 -0.84919 0.414 Inf -2.051
 Orco WingClip Week2 - WT WingRemoved Week5 -1.09807 0.459 Inf -2.392
 WT WingClip Week2 - Orco WingRemoved Week2 0.08192 0.326 Inf 0.251
 WT WingClip Week2 - WT WingRemoved Week2 -0.16696 0.265 Inf -0.629
 WT WingClip Week2 - Orco Single Week3 -1.19365 0.432 Inf -2.764
 WT WingClip Week2 - WT Single Week3 -1.44253 0.390 Inf -3.703
 WT WingClip Week2 - Orco WingClip Week3 -0.46649 0.362 Inf -1.288
 WT WingClip Week2 - WT WingClip Week3 -0.71537 0.308 Inf -2.323
 WT WingClip Week2 - Orco WingRemoved Week3 -0.63345 0.448 Inf -1.415
 WT WingClip Week2 - WT WingRemoved Week3 -0.88233 0.407 Inf -2.170
 WT WingClip Week2 - Orco Single Week4 -0.66395 0.462 Inf -1.438
 WT WingClip Week2 - WT Single Week4 -0.91283 0.422 Inf -2.162
 WT WingClip Week2 - Orco WingClip Week4 0.06320 0.397 Inf 0.159
 WT WingClip Week2 - WT WingClip Week4 -0.18568 0.349 Inf -0.533
 WT WingClip Week2 - Orco WingRemoved Week4 -0.10375 0.477 Inf -0.218
 WT WingClip Week2 - WT WingRemoved Week4 -0.35263 0.438 Inf -0.805
 WT WingClip Week2 - Orco Single Week5 -1.16051 0.439 Inf -2.645
 WT WingClip Week2 - WT Single Week5 -1.40939 0.397 Inf -3.551
 WT WingClip Week2 - Orco WingClip Week5 -0.43335 0.371 Inf -1.167
 WT WingClip Week2 - WT WingClip Week5 -0.68223 0.318 Inf -2.145
 WT WingClip Week2 - Orco WingRemoved Week5 -0.60031 0.455 Inf -1.320
 WT WingClip Week2 - WT WingRemoved Week5 -0.84919 0.414 Inf -2.051
 Orco WingRemoved Week2 - WT WingRemoved Week2 -0.24888 0.193 Inf -1.290
 Orco WingRemoved Week2 - Orco Single Week3 -1.27557 0.384 Inf -3.321
 Orco WingRemoved Week2 - WT Single Week3 -1.52445 0.431 Inf -3.536
 Orco WingRemoved Week2 - Orco WingClip Week3 -0.54842 0.407 Inf -1.349
 Orco WingRemoved Week2 - WT WingClip Week3 -0.79730 0.450 Inf -1.774
 Orco WingRemoved Week2 - Orco WingRemoved Week3 -0.71537 0.308 Inf -2.323
 Orco WingRemoved Week2 - WT WingRemoved Week3 -0.96425 0.365 Inf -2.645
 Orco WingRemoved Week2 - Orco Single Week4 -0.74588 0.417 Inf -1.787
 Orco WingRemoved Week2 - WT Single Week4 -0.99476 0.461 Inf -2.158
 Orco WingRemoved Week2 - Orco WingClip Week4 -0.01872 0.438 Inf -0.043
 Orco WingRemoved Week2 - WT WingClip Week4 -0.26760 0.478 Inf -0.560
 Orco WingRemoved Week2 - Orco WingRemoved Week4 -0.18568 0.349 Inf -0.533
 Orco WingRemoved Week2 - WT WingRemoved Week4 -0.43456 0.399 Inf -1.088
 Orco WingRemoved Week2 - Orco Single Week5 -1.24243 0.392 Inf -3.171
 Orco WingRemoved Week2 - WT Single Week5 -1.49131 0.438 Inf -3.406
 Orco WingRemoved Week2 - Orco WingClip Week5 -0.51528 0.415 Inf -1.243
 Orco WingRemoved Week2 - WT WingClip Week5 -0.76416 0.457 Inf -1.674
 Orco WingRemoved Week2 - Orco WingRemoved Week5 -0.68223 0.318 Inf -2.145
 Orco WingRemoved Week2 - WT WingRemoved Week5 -0.93111 0.373 Inf -2.497
 WT WingRemoved Week2 - Orco Single Week3 -1.02669 0.429 Inf -2.396
 WT WingRemoved Week2 - WT Single Week3 -1.27557 0.384 Inf -3.321
 WT WingRemoved Week2 - Orco WingClip Week3 -0.29954 0.451 Inf -0.665
 WT WingRemoved Week2 - WT WingClip Week3 -0.54842 0.407 Inf -1.349
 WT WingRemoved Week2 - Orco WingRemoved Week3 -0.46649 0.362 Inf -1.288
 WT WingRemoved Week2 - WT WingRemoved Week3 -0.71537 0.308 Inf -2.323
 WT WingRemoved Week2 - Orco Single Week4 -0.49700 0.459 Inf -1.083
 WT WingRemoved Week2 - WT Single Week4 -0.74588 0.417 Inf -1.787
 WT WingRemoved Week2 - Orco WingClip Week4 0.23016 0.479 Inf 0.480
 WT WingRemoved Week2 - WT WingClip Week4 -0.01872 0.438 Inf -0.043
 WT WingRemoved Week2 - Orco WingRemoved Week4 0.06320 0.397 Inf 0.159
 WT WingRemoved Week2 - WT WingRemoved Week4 -0.18568 0.349 Inf -0.533
 WT WingRemoved Week2 - Orco Single Week5 -0.99355 0.436 Inf -2.281
 WT WingRemoved Week2 - WT Single Week5 -1.24243 0.392 Inf -3.171
 WT WingRemoved Week2 - Orco WingClip Week5 -0.26640 0.458 Inf -0.582
 WT WingRemoved Week2 - WT WingClip Week5 -0.51528 0.415 Inf -1.243
 WT WingRemoved Week2 - Orco WingRemoved Week5 -0.43335 0.371 Inf -1.167
 WT WingRemoved Week2 - WT WingRemoved Week5 -0.68223 0.318 Inf -2.145
 Orco Single Week3 - WT Single Week3 -0.24888 0.193 Inf -1.290
 Orco Single Week3 - Orco WingClip Week3 0.72715 0.238 Inf 3.051
 Orco Single Week3 - WT WingClip Week3 0.47827 0.304 Inf 1.573
 Orco Single Week3 - Orco WingRemoved Week3 0.56020 0.229 Inf 2.442
 Orco Single Week3 - WT WingRemoved Week3 0.31132 0.299 Inf 1.040
 Orco Single Week3 - Orco Single Week4 0.52970 0.302 Inf 1.754
 Orco Single Week3 - WT Single Week4 0.28082 0.358 Inf 0.784
 Orco Single Week3 - Orco WingClip Week4 1.25685 0.385 Inf 3.267
 Orco Single Week3 - WT WingClip Week4 1.00797 0.428 Inf 2.353
 Orco Single Week3 - Orco WingRemoved Week4 1.08990 0.379 Inf 2.875
 Orco Single Week3 - WT WingRemoved Week4 0.84102 0.425 Inf 1.979
 Orco Single Week3 - Orco Single Week5 0.03314 0.266 Inf 0.124
 Orco Single Week3 - WT Single Week5 -0.21574 0.329 Inf -0.657
 Orco Single Week3 - Orco WingClip Week5 0.76030 0.358 Inf 2.123
 Orco Single Week3 - WT WingClip Week5 0.51142 0.405 Inf 1.264
 Orco Single Week3 - Orco WingRemoved Week5 0.59334 0.352 Inf 1.686
 Orco Single Week3 - WT WingRemoved Week5 0.34446 0.401 Inf 0.859
 WT Single Week3 - Orco WingClip Week3 0.97603 0.309 Inf 3.156
 WT Single Week3 - WT WingClip Week3 0.72715 0.238 Inf 3.051
 WT Single Week3 - Orco WingRemoved Week3 0.80908 0.300 Inf 2.694
 WT Single Week3 - WT WingRemoved Week3 0.56020 0.229 Inf 2.442
 WT Single Week3 - Orco Single Week4 0.77858 0.358 Inf 2.172
 WT Single Week3 - WT Single Week4 0.52970 0.302 Inf 1.754
 WT Single Week3 - Orco WingClip Week4 1.50573 0.432 Inf 3.483
 WT Single Week3 - WT WingClip Week4 1.25685 0.385 Inf 3.267
 WT Single Week3 - Orco WingRemoved Week4 1.33878 0.426 Inf 3.144
 WT Single Week3 - WT WingRemoved Week4 1.08990 0.379 Inf 2.875
 WT Single Week3 - Orco Single Week5 0.28202 0.329 Inf 0.857
 WT Single Week3 - WT Single Week5 0.03314 0.266 Inf 0.124
 WT Single Week3 - Orco WingClip Week5 1.00918 0.409 Inf 2.467
 WT Single Week3 - WT WingClip Week5 0.76030 0.358 Inf 2.123
 WT Single Week3 - Orco WingRemoved Week5 0.84222 0.402 Inf 2.095
 WT Single Week3 - WT WingRemoved Week5 0.59334 0.352 Inf 1.686
 Orco WingClip Week3 - WT WingClip Week3 -0.24888 0.193 Inf -1.290
 Orco WingClip Week3 - Orco WingRemoved Week3 -0.16696 0.265 Inf -0.629
 Orco WingClip Week3 - WT WingRemoved Week3 -0.41584 0.330 Inf -1.259
 Orco WingClip Week3 - Orco Single Week4 -0.19746 0.385 Inf -0.513
 Orco WingClip Week3 - WT Single Week4 -0.44634 0.432 Inf -1.033
 Orco WingClip Week3 - Orco WingClip Week4 0.52970 0.302 Inf 1.754
 Orco WingClip Week3 - WT WingClip Week4 0.28082 0.358 Inf 0.784
 Orco WingClip Week3 - Orco WingRemoved Week4 0.36274 0.402 Inf 0.902
 Orco WingClip Week3 - WT WingRemoved Week4 0.11386 0.447 Inf 0.255
 Orco WingClip Week3 - Orco Single Week5 -0.69401 0.357 Inf -1.947
 Orco WingClip Week3 - WT Single Week5 -0.94289 0.407 Inf -2.316
 Orco WingClip Week3 - Orco WingClip Week5 0.03314 0.266 Inf 0.124
 Orco WingClip Week3 - WT WingClip Week5 -0.21574 0.329 Inf -0.657
 Orco WingClip Week3 - Orco WingRemoved Week5 -0.13381 0.376 Inf -0.356
 Orco WingClip Week3 - WT WingRemoved Week5 -0.38269 0.424 Inf -0.903
 WT WingClip Week3 - Orco WingRemoved Week3 0.08192 0.326 Inf 0.251
 WT WingClip Week3 - WT WingRemoved Week3 -0.16696 0.265 Inf -0.629
 WT WingClip Week3 - Orco Single Week4 0.05142 0.429 Inf 0.120
 WT WingClip Week3 - WT Single Week4 -0.19746 0.385 Inf -0.513
 WT WingClip Week3 - Orco WingClip Week4 0.77858 0.358 Inf 2.172
 WT WingClip Week3 - WT WingClip Week4 0.52970 0.302 Inf 1.754
 WT WingClip Week3 - Orco WingRemoved Week4 0.61162 0.445 Inf 1.376
 WT WingClip Week3 - WT WingRemoved Week4 0.36274 0.402 Inf 0.902
 WT WingClip Week3 - Orco Single Week5 -0.44513 0.404 Inf -1.103
 WT WingClip Week3 - WT Single Week5 -0.69401 0.357 Inf -1.947
 WT WingClip Week3 - Orco WingClip Week5 0.28202 0.329 Inf 0.857
 WT WingClip Week3 - WT WingClip Week5 0.03314 0.266 Inf 0.124
 WT WingClip Week3 - Orco WingRemoved Week5 0.11507 0.421 Inf 0.273
 WT WingClip Week3 - WT WingRemoved Week5 -0.13381 0.376 Inf -0.356
 Orco WingRemoved Week3 - WT WingRemoved Week3 -0.24888 0.193 Inf -1.290
 Orco WingRemoved Week3 - Orco Single Week4 -0.03050 0.379 Inf -0.080
 Orco WingRemoved Week3 - WT Single Week4 -0.27938 0.426 Inf -0.656
 Orco WingRemoved Week3 - Orco WingClip Week4 0.69665 0.402 Inf 1.732
 Orco WingRemoved Week3 - WT WingClip Week4 0.44777 0.445 Inf 1.007
 Orco WingRemoved Week3 - Orco WingRemoved Week4 0.52970 0.302 Inf 1.754
 Orco WingRemoved Week3 - WT WingRemoved Week4 0.28082 0.358 Inf 0.784
 Orco WingRemoved Week3 - Orco Single Week5 -0.52706 0.351 Inf -1.502
 Orco WingRemoved Week3 - WT Single Week5 -0.77594 0.401 Inf -1.937
 Orco WingRemoved Week3 - Orco WingClip Week5 0.20010 0.376 Inf 0.532
 Orco WingRemoved Week3 - WT WingClip Week5 -0.04878 0.421 Inf -0.116
 Orco WingRemoved Week3 - Orco WingRemoved Week5 0.03314 0.266 Inf 0.124
 Orco WingRemoved Week3 - WT WingRemoved Week5 -0.21574 0.329 Inf -0.657
 WT WingRemoved Week3 - Orco Single Week4 0.21838 0.425 Inf 0.513
 WT WingRemoved Week3 - WT Single Week4 -0.03050 0.379 Inf -0.080
 WT WingRemoved Week3 - Orco WingClip Week4 0.94553 0.448 Inf 2.112
 WT WingRemoved Week3 - WT WingClip Week4 0.69665 0.402 Inf 1.732
 WT WingRemoved Week3 - Orco WingRemoved Week4 0.77858 0.358 Inf 2.172
 WT WingRemoved Week3 - WT WingRemoved Week4 0.52970 0.302 Inf 1.754
 WT WingRemoved Week3 - Orco Single Week5 -0.27818 0.400 Inf -0.695
 WT WingRemoved Week3 - WT Single Week5 -0.52706 0.351 Inf -1.502
 WT WingRemoved Week3 - Orco WingClip Week5 0.44898 0.425 Inf 1.057
 WT WingRemoved Week3 - WT WingClip Week5 0.20010 0.376 Inf 0.532
 WT WingRemoved Week3 - Orco WingRemoved Week5 0.28202 0.329 Inf 0.857
 WT WingRemoved Week3 - WT WingRemoved Week5 0.03314 0.266 Inf 0.124
 Orco Single Week4 - WT Single Week4 -0.24888 0.193 Inf -1.290
 Orco Single Week4 - Orco WingClip Week4 0.72715 0.238 Inf 3.051
 Orco Single Week4 - WT WingClip Week4 0.47827 0.304 Inf 1.573
 Orco Single Week4 - Orco WingRemoved Week4 0.56020 0.229 Inf 2.442
 Orco Single Week4 - WT WingRemoved Week4 0.31132 0.299 Inf 1.040
 Orco Single Week4 - Orco Single Week5 -0.49656 0.312 Inf -1.591
 Orco Single Week4 - WT Single Week5 -0.74544 0.367 Inf -2.033
 Orco Single Week4 - Orco WingClip Week5 0.23060 0.393 Inf 0.586
 Orco Single Week4 - WT WingClip Week5 -0.01828 0.436 Inf -0.042
 Orco Single Week4 - Orco WingRemoved Week5 0.06364 0.388 Inf 0.164
 Orco Single Week4 - WT WingRemoved Week5 -0.18524 0.433 Inf -0.428
 WT Single Week4 - Orco WingClip Week4 0.97603 0.309 Inf 3.156
 WT Single Week4 - WT WingClip Week4 0.72715 0.238 Inf 3.051
 WT Single Week4 - Orco WingRemoved Week4 0.80908 0.300 Inf 2.694
 WT Single Week4 - WT WingRemoved Week4 0.56020 0.229 Inf 2.442
 WT Single Week4 - Orco Single Week5 -0.24768 0.367 Inf -0.675
 WT Single Week4 - WT Single Week5 -0.49656 0.312 Inf -1.591
 WT Single Week4 - Orco WingClip Week5 0.47948 0.440 Inf 1.090
 WT Single Week4 - WT WingClip Week5 0.23060 0.393 Inf 0.586
 WT Single Week4 - Orco WingRemoved Week5 0.31252 0.434 Inf 0.721
 WT Single Week4 - WT WingRemoved Week5 0.06364 0.388 Inf 0.164
 Orco WingClip Week4 - WT WingClip Week4 -0.24888 0.193 Inf -1.290
 Orco WingClip Week4 - Orco WingRemoved Week4 -0.16696 0.265 Inf -0.629
 Orco WingClip Week4 - WT WingRemoved Week4 -0.41584 0.330 Inf -1.259
 Orco WingClip Week4 - Orco Single Week5 -1.22371 0.392 Inf -3.122
 Orco WingClip Week4 - WT Single Week5 -1.47259 0.439 Inf -3.358
 Orco WingClip Week4 - Orco WingClip Week5 -0.49656 0.312 Inf -1.591
 Orco WingClip Week4 - WT WingClip Week5 -0.74544 0.367 Inf -2.033
 Orco WingClip Week4 - Orco WingRemoved Week5 -0.66351 0.410 Inf -1.620
 Orco WingClip Week4 - WT WingRemoved Week5 -0.91239 0.454 Inf -2.009
 WT WingClip Week4 - Orco WingRemoved Week4 0.08192 0.326 Inf 0.251
 WT WingClip Week4 - WT WingRemoved Week4 -0.16696 0.265 Inf -0.629
 WT WingClip Week4 - Orco Single Week5 -0.97483 0.435 Inf -2.240
 WT WingClip Week4 - WT Single Week5 -1.22371 0.392 Inf -3.122
 WT WingClip Week4 - Orco WingClip Week5 -0.24768 0.367 Inf -0.675
 WT WingClip Week4 - WT WingClip Week5 -0.49656 0.312 Inf -1.591
 WT WingClip Week4 - Orco WingRemoved Week5 -0.41463 0.451 Inf -0.918
 WT WingClip Week4 - WT WingRemoved Week5 -0.66351 0.410 Inf -1.620
 Orco WingRemoved Week4 - WT WingRemoved Week4 -0.24888 0.193 Inf -1.290
 Orco WingRemoved Week4 - Orco Single Week5 -1.05675 0.387 Inf -2.733
 Orco WingRemoved Week4 - WT Single Week5 -1.30563 0.432 Inf -3.019
 Orco WingRemoved Week4 - Orco WingClip Week5 -0.32960 0.410 Inf -0.804
 Orco WingRemoved Week4 - WT WingClip Week5 -0.57848 0.451 Inf -1.281
 Orco WingRemoved Week4 - Orco WingRemoved Week5 -0.49656 0.312 Inf -1.591
 Orco WingRemoved Week4 - WT WingRemoved Week5 -0.74544 0.367 Inf -2.033
 WT WingRemoved Week4 - Orco Single Week5 -0.80787 0.432 Inf -1.870
 WT WingRemoved Week4 - WT Single Week5 -1.05675 0.387 Inf -2.733
 WT WingRemoved Week4 - Orco WingClip Week5 -0.08072 0.455 Inf -0.178
 WT WingRemoved Week4 - WT WingClip Week5 -0.32960 0.410 Inf -0.804
 WT WingRemoved Week4 - Orco WingRemoved Week5 -0.24768 0.367 Inf -0.675
 WT WingRemoved Week4 - WT WingRemoved Week5 -0.49656 0.312 Inf -1.591
 Orco Single Week5 - WT Single Week5 -0.24888 0.193 Inf -1.290
 Orco Single Week5 - Orco WingClip Week5 0.72715 0.238 Inf 3.051
 Orco Single Week5 - WT WingClip Week5 0.47827 0.304 Inf 1.573
 Orco Single Week5 - Orco WingRemoved Week5 0.56020 0.229 Inf 2.442
 Orco Single Week5 - WT WingRemoved Week5 0.31132 0.299 Inf 1.040
 WT Single Week5 - Orco WingClip Week5 0.97603 0.309 Inf 3.156
 WT Single Week5 - WT WingClip Week5 0.72715 0.238 Inf 3.051
 WT Single Week5 - Orco WingRemoved Week5 0.80908 0.300 Inf 2.694
 WT Single Week5 - WT WingRemoved Week5 0.56020 0.229 Inf 2.442
 Orco WingClip Week5 - WT WingClip Week5 -0.24888 0.193 Inf -1.290
 Orco WingClip Week5 - Orco WingRemoved Week5 -0.16696 0.265 Inf -0.629
 Orco WingClip Week5 - WT WingRemoved Week5 -0.41584 0.330 Inf -1.259
 WT WingClip Week5 - Orco WingRemoved Week5 0.08192 0.326 Inf 0.251
 WT WingClip Week5 - WT WingRemoved Week5 -0.16696 0.265 Inf -0.629
 Orco WingRemoved Week5 - WT WingRemoved Week5 -0.24888 0.193 Inf -1.290
 p.value
 1.0000
 0.3465
 0.9995
 0.8191
 1.0000
 0.9709
 1.0000
 0.1117
 0.7371
 0.3085
 0.9370
 1.0000
 1.0000
 0.9931
 1.0000
 1.0000
 1.0000
 0.9999
 1.0000
 0.3440
 0.9492
 0.6664
 0.9964
 1.0000
 1.0000
 0.9904
 1.0000
 0.9999
 1.0000
 0.2749
 0.3465
 0.6332
 0.8191
 0.8268
 0.9709
 0.0597
 0.1117
 0.1663
 0.3085
 1.0000
 1.0000
 0.9192
 0.9931
 0.9930
 1.0000
 0.9854
 0.9999
 0.1900
 0.3440
 0.4162
 0.6664
 1.0000
 1.0000
 0.9063
 0.9904
 0.9902
 0.9999
 1.0000
 1.0000
 1.0000
 1.0000
 1.0000
 0.9709
 1.0000
 1.0000
 1.0000
 0.8754
 0.6711
 1.0000
 1.0000
 1.0000
 1.0000
 1.0000
 1.0000
 0.9999
 1.0000
 1.0000
 1.0000
 0.9357
 0.7660
 1.0000
 1.0000
 1.0000
 1.0000
 1.0000
 1.0000
 1.0000
 1.0000
 0.8268
 0.9709
 0.9994
 1.0000
 0.9999
 0.8754
 1.0000
 1.0000
 1.0000
 1.0000
 1.0000
 1.0000
 0.9854
 0.9999
 1.0000
 1.0000
 1.0000
 0.9357
 1.0000
 1.0000
 1.0000
 1.0000
 1.0000
 1.0000
 1.0000
 0.9835
 1.0000
 0.9709
 1.0000
 0.9898
 0.9064
 1.0000
 1.0000
 1.0000
 1.0000
 1.0000
 1.0000
 0.9997
 1.0000
 0.9999
 1.0000
 0.9969
 0.9488
 1.0000
 1.0000
 1.0000
 1.0000
 1.0000
 1.0000
 0.8836
 0.9835
 0.8268
 0.9709
 1.0000
 0.9898
 1.0000
 1.0000
 1.0000
 1.0000
 1.0000
 1.0000
 0.9869
 0.9997
 0.9854
 0.9999
 1.0000
 0.9969
 1.0000
 1.0000
 1.0000
 1.0000
 1.0000
 0.3465
 0.9995
 0.8191
 1.0000
 0.8843
 0.6732
 1.0000
 1.0000
 1.0000
 1.0000
 1.0000
 1.0000
 1.0000
 1.0000
 1.0000
 1.0000
 0.9502
 0.7832
 1.0000
 1.0000
 1.0000
 1.0000
 0.2749
 0.3465
 0.6332
 0.8191
 1.0000
 0.8843
 1.0000
 1.0000
 1.0000
 1.0000
 1.0000
 1.0000
 0.9982
 1.0000
 1.0000
 1.0000
 1.0000
 0.9502
 1.0000
 1.0000
 1.0000
 1.0000
 1.0000
 1.0000
 1.0000
 0.0582
 0.0331
 0.8843
 0.6732
 0.9432
 0.7810
 0.9456
 0.7887
 1.0000
 1.0000
 1.0000
 1.0000
 0.0947
 0.0521
 0.9502
 0.7832
 0.9711
 0.8483
 1.0000
 1.0000
 0.5759
 0.0582
 1.0000
 0.8843
 0.9999
 0.9432
 0.9999
 0.9456
 1.0000
 1.0000
 1.0000
 1.0000
 0.6732
 0.0947
 1.0000
 0.9502
 1.0000
 0.9711
 1.0000
 0.1828
 0.0993
 1.0000
 0.9963
 0.8843
 0.6732
 0.9959
 0.9466
 1.0000
 1.0000
 1.0000
 1.0000
 0.2655
 0.1449
 1.0000
 0.9986
 0.9502
 0.7832
 0.8462
 0.1828
 1.0000
 1.0000
 1.0000
 0.8843
 1.0000
 0.9959
 1.0000
 1.0000
 1.0000
 1.0000
 0.9035
 0.2655
 1.0000
 1.0000
 1.0000
 0.9502
 1.0000
 0.3465
 0.9995
 0.8191
 1.0000
 0.9969
 1.0000
 0.2102
 0.8696
 0.4835
 0.9818
 1.0000
 1.0000
 0.9558
 1.0000
 0.9984
 1.0000
 0.2749
 0.3465
 0.6332
 0.8191
 0.9426
 0.9969
 0.1164
 0.2102
 0.2831
 0.4835
 1.0000
 1.0000
 0.8028
 0.9558
 0.9623
 0.9984
 1.0000
 1.0000
 1.0000
 1.0000
 1.0000
 0.9969
 1.0000
 1.0000
 1.0000
 0.9854
 0.8876
 1.0000
 1.0000
 1.0000
 1.0000
 1.0000
 1.0000
 1.0000
 1.0000
 0.9426
 0.9969
 1.0000
 1.0000
 1.0000
 0.9854
 1.0000
 1.0000
 1.0000
 1.0000
 1.0000
 1.0000
 1.0000
 0.9975
 1.0000
 0.9969
 1.0000
 0.9998
 0.9864
 1.0000
 1.0000
 1.0000
 1.0000
 1.0000
 1.0000
 0.9584
 0.9975
 0.9426
 0.9969
 1.0000
 0.9998
 1.0000
 1.0000
 1.0000
 1.0000
 1.0000
 0.3465
 0.9995
 0.8191
 1.0000
 0.9994
 0.9741
 1.0000
 1.0000
 1.0000
 1.0000
 0.2749
 0.3465
 0.6332
 0.8191
 1.0000
 0.9994
 1.0000
 1.0000
 1.0000
 1.0000
 1.0000
 1.0000
 1.0000
 0.2971
 0.1657
 0.9994
 0.9741
 0.9992
 0.9777
 1.0000
 1.0000
 0.9197
 0.2971
 1.0000
 0.9994
 1.0000
 0.9992
 1.0000
 0.6017
 0.3697
 1.0000
 1.0000
 0.9994
 0.9741
 0.9918
 0.6017
 1.0000
 1.0000
 1.0000
 0.9994
 1.0000
 0.3465
 0.9995
 0.8191
 1.0000
 0.2749
 0.3465
 0.6332
 0.8191
 1.0000
 1.0000
 1.0000
 1.0000
 1.0000
 1.0000

Results are given on the log (not the response) scale.
P value adjustment: tukey method for comparing a family of 30 estimates

lsmeans(OrcoGrooming_glmmTMB2, pairwise ~ Focal_Treatment * Rival_Treatment, adjust="tukey")

$lsmeans
 Focal_Treatment Rival_Treatment lsmean SE df asymp.LCL asymp.UCL
 Orco Single -2.29 0.211 Inf -2.70 -1.87
 WT Single -2.04 0.207 Inf -2.44 -1.63
 Orco WingClip -3.01 0.253 Inf -3.51 -2.52
 WT WingClip -2.76 0.247 Inf -3.25 -2.28
 Orco WingRemoved -2.85 0.242 Inf -3.32 -2.37
 WT WingRemoved -2.60 0.237 Inf -3.06 -2.13

Results are averaged over the levels of: Week
Results are given on the log (not the response) scale.
Confidence level used: 0.95

$contrasts
 contrast estimate SE df z.ratio p.value
 Orco Single - WT Single -0.2489 0.193 Inf -1.290 0.7909
 Orco Single - Orco WingClip 0.7272 0.238 Inf 3.051 0.0277
 Orco Single - WT WingClip 0.4783 0.304 Inf 1.573 0.6166
 Orco Single - Orco WingRemoved 0.5602 0.229 Inf 2.442 0.1420
 Orco Single - WT WingRemoved 0.3113 0.299 Inf 1.040 0.9044
 WT Single - Orco WingClip 0.9760 0.309 Inf 3.156 0.0199
 WT Single - WT WingClip 0.7272 0.238 Inf 3.051 0.0277
 WT Single - Orco WingRemoved 0.8091 0.300 Inf 2.694 0.0763
 WT Single - WT WingRemoved 0.5602 0.229 Inf 2.442 0.1420
 Orco WingClip - WT WingClip -0.2489 0.193 Inf -1.290 0.7909
 Orco WingClip - Orco WingRemoved -0.1670 0.265 Inf -0.629 0.9889
 Orco WingClip - WT WingRemoved -0.4158 0.330 Inf -1.259 0.8070
 WT WingClip - Orco WingRemoved 0.0819 0.326 Inf 0.251 0.9999
 WT WingClip - WT WingRemoved -0.1670 0.265 Inf -0.629 0.9889
 Orco WingRemoved - WT WingRemoved -0.2489 0.193 Inf -1.290 0.7909

Results are averaged over the levels of: Week
Results are given on the log (not the response) scale.
P value adjustment: tukey method for comparing a family of 6 estimates

lsmeans(OrcoGrooming_glmmTMB2, pairwise ~ Focal_Treatment, adjust="tukey")

$lsmeans
 Focal_Treatment lsmean SE df asymp.LCL asymp.UCL
 Orco -2.72 0.189 Inf -3.09 -2.35
 WT -2.47 0.183 Inf -2.82 -2.11

Results are averaged over the levels of: Rival_Treatment, Week
Results are given on the log (not the response) scale.
Confidence level used: 0.95

$contrasts
 contrast estimate SE df z.ratio p.value
 Orco - WT -0.249 0.193 Inf -1.290 0.1972

Results are averaged over the levels of: Rival_Treatment, Week
Results are given on the log (not the response) scale.

lsmeans(OrcoGrooming_glmmTMB2, pairwise ~ Rival_Treatment, adjust="tukey")

$lsmeans
 Rival_Treatment lsmean SE df asymp.LCL asymp.UCL
 Single -2.16 0.185 Inf -2.52 -1.80
 WingClip -2.89 0.231 Inf -3.34 -2.44
 WingRemoved -2.72 0.219 Inf -3.15 -2.29

Results are averaged over the levels of: Focal_Treatment, Week
Results are given on the log (not the response) scale.
Confidence level used: 0.95

$contrasts
 contrast estimate SE df z.ratio p.value
 Single - WingClip 0.727 0.238 Inf 3.051 0.0065
 Single - WingRemoved 0.560 0.229 Inf 2.442 0.0388
 WingClip - WingRemoved -0.167 0.265 Inf -0.629 0.8042

Results are averaged over the levels of: Focal_Treatment, Week
Results are given on the log (not the response) scale.
P value adjustment: tukey method for comparing a family of 3 estimates

lsmeans(OrcoGrooming_glmmTMB2, pairwise ~ Week, adjust="tukey")

$lsmeans
 Week lsmean SE df asymp.LCL asymp.UCL
 1 -2.40 0.218 Inf -2.83 -1.98
 2 -3.03 0.281 Inf -3.58 -2.48
 3 -2.32 0.219 Inf -2.75 -1.89
 4 -2.85 0.274 Inf -3.38 -2.31
 5 -2.35 0.236 Inf -2.81 -1.89

Results are averaged over the levels of: Focal_Treatment, Rival_Treatment
Results are given on the log (not the response) scale.
Confidence level used: 0.95

$contrasts
 contrast estimate SE df z.ratio p.value
 Week1 - Week2 0.6284 0.306 Inf 2.051 0.2416
 Week1 - Week3 -0.0870 0.252 Inf -0.345 0.9970
 Week1 - Week4 0.4427 0.301 Inf 1.473 0.5800
 Week1 - Week5 -0.0538 0.265 Inf -0.204 0.9996
 Week2 - Week3 -0.7154 0.308 Inf -2.323 0.1375
 Week2 - Week4 -0.1857 0.349 Inf -0.533 0.9840
 Week2 - Week5 -0.6822 0.318 Inf -2.145 0.2012
 Week3 - Week4 0.5297 0.302 Inf 1.754 0.4006
 Week3 - Week5 0.0331 0.266 Inf 0.124 0.9999
 Week4 - Week5 -0.4966 0.312 Inf -1.591 0.5029

Results are averaged over the levels of: Focal_Treatment, Rival_Treatment
Results are given on the log (not the response) scale.
P value adjustment: tukey method for comparing a family of 5 estimates

###Orco Inactive
##Orco Inactive Model
Orco_Inactive$Week <- as.factor(Orco_Inactive$Week)
OrcoInactive_glmmTMB1 <- glmmTMB(Success ~ Focal_Treatment * Rival_Treatment + Week + (1|FlyID), family=poisson(link = "log"), data = Orco_Inactive)
OrcoInactive_glmmTMB2 <- glmmTMB(Success ~ Focal_Treatment + Rival_Treatment + Week + (1|FlyID), family=poisson(link = "log"), data = Orco_Inactive)
anova(OrcoInactive_glmmTMB1, OrcoInactive_glmmTMB2, test="Chi")

Data: Orco_Inactive
Models:
OrcoInactive_glmmTMB2: Success ~ Focal_Treatment + Rival_Treatment + Week + (1 | FlyID), zi=~0, disp=~1
OrcoInactive_glmmTMB1: Success ~ Focal_Treatment * Rival_Treatment + Week + (1 | FlyID), zi=~0, disp=~1
 Df AIC BIC logLik deviance Chisq Chi Df
OrcoInactive_glmmTMB2 9 7070.7 7117.3 -3526.3 7052.7
OrcoInactive_glmmTMB1 11 7060.7 7117.7 -3519.3 7038.7 14.002 2
 Pr(>Chisq)
OrcoInactive_glmmTMB2
OrcoInactive_glmmTMB1 0.0009112 ***
---
Signif. codes: 0 '***' 0.001 '**' 0.01 '*' 0.05 '.' 0.1 ' ' 1

OrcoInactive_glmmTMB3 <- glmmTMB(Success ~ Rival_Treatment + Week + (1|FlyID), family=poisson(link = "log"), data = Orco_Inactive)
anova(OrcoInactive_glmmTMB1, OrcoInactive_glmmTMB3, test="Chi")

Data: Orco_Inactive
Models:
OrcoInactive_glmmTMB3: Success ~ Rival_Treatment + Week + (1 | FlyID), zi=~0, disp=~1
OrcoInactive_glmmTMB1: Success ~ Focal_Treatment * Rival_Treatment + Week + (1 | FlyID), zi=~0, disp=~1
 Df AIC BIC logLik deviance Chisq Chi Df
OrcoInactive_glmmTMB3 8 7170.4 7211.9 -3577.2 7154.4
OrcoInactive_glmmTMB1 11 7060.7 7117.7 -3519.3 7038.7 115.73 3
 Pr(>Chisq)
OrcoInactive_glmmTMB3
OrcoInactive_glmmTMB1 < 2.2e-16 ***
---
Signif. codes: 0 '***' 0.001 '**' 0.01 '*' 0.05 '.' 0.1 ' ' 1

OrcoInactive_glmmTMB4 <- glmmTMB(Success ~ Focal_Treatment + Week + (1|FlyID), family=poisson(link = "log"), data = Orco_Inactive)
anova(OrcoInactive_glmmTMB1, OrcoInactive_glmmTMB4, test="Chi")

Data: Orco_Inactive
Models:
OrcoInactive_glmmTMB4: Success ~ Focal_Treatment + Week + (1 | FlyID), zi=~0, disp=~1
OrcoInactive_glmmTMB1: Success ~ Focal_Treatment * Rival_Treatment + Week + (1 | FlyID), zi=~0, disp=~1
 Df AIC BIC logLik deviance Chisq Chi Df
OrcoInactive_glmmTMB4 7 7079.5 7115.8 -3532.7 7065.5
OrcoInactive_glmmTMB1 11 7060.7 7117.7 -3519.3 7038.7 26.813 4
 Pr(>Chisq)
OrcoInactive_glmmTMB4
OrcoInactive_glmmTMB1 2.168e-05 ***
---
Signif. codes: 0 '***' 0.001 '**' 0.01 '*' 0.05 '.' 0.1 ' ' 1

OrcoInactive_glmmTMB5 <- glmmTMB(Success ~ Focal_Treatment + Rival_Treatment + + (1|FlyID), family=poisson(link = "log"), data = Orco_Inactive)
anova(OrcoInactive_glmmTMB1, OrcoInactive_glmmTMB5, test="Chi")

Data: Orco_Inactive
Models:
OrcoInactive_glmmTMB5: Success ~ Focal_Treatment + Rival_Treatment + +(1 | FlyID), zi=~0, disp=~1
OrcoInactive_glmmTMB1: Success ~ Focal_Treatment * Rival_Treatment + Week + (1 | FlyID), zi=~0, disp=~1
 Df AIC BIC logLik deviance Chisq Chi Df
OrcoInactive_glmmTMB5 5 7125.4 7151.3 -3557.7 7115.4
OrcoInactive_glmmTMB1 11 7060.7 7117.7 -3519.3 7038.7 76.718 6
 Pr(>Chisq)
OrcoInactive_glmmTMB5
OrcoInactive_glmmTMB1 1.699e-14 ***
---
Signif. codes: 0 '***' 0.001 '**' 0.01 '*' 0.05 '.' 0.1 ' ' 1

OrcoInactive_glmmTMB_null <- glmmTMB(Success ~ (1|FlyID), family=poisson(link = "log"), data = Orco_Inactive)
anova(OrcoInactive_glmmTMB1, OrcoInactive_glmmTMB_null, test="Chi")

Data: Orco_Inactive
Models:
OrcoInactive_glmmTMB_null: Success ~ (1 | FlyID), zi=~0, disp=~1
OrcoInactive_glmmTMB1: Success ~ Focal_Treatment * Rival_Treatment + Week + (1 | FlyID), zi=~0, disp=~1
 Df AIC BIC logLik deviance Chisq Chi Df
OrcoInactive_glmmTMB_null 2 7229.9 7240.3 -3613.0 7225.9
OrcoInactive_glmmTMB1 11 7060.7 7117.7 -3519.3 7038.7 187.22 9
 Pr(>Chisq)
OrcoInactive_glmmTMB_null
OrcoInactive_glmmTMB1 < 2.2e-16 ***
---
Signif. codes: 0 '***' 0.001 '**' 0.01 '*' 0.05 '.' 0.1 ' ' 1

summary(OrcoInactive_glmmTMB1)

Family: poisson ( log )
Formula:
Success ~ Focal_Treatment * Rival_Treatment + Week + (1 | FlyID)
Data: Orco_Inactive

 AIC BIC logLik deviance df.resid
 7060.7 7117.7 -3519.3 7038.7 1308

Random effects:

Conditional model:
 Groups Name Variance Std.Dev.
 FlyID (Intercept) 0.1043 0.3229
Number of obs: 1319, groups: FlyID, 300

Conditional model:
 Estimate Std. Error z value
(Intercept) 2.02284 0.05639 35.87
Focal_TreatmentWT -0.60255 0.07781 -7.74
Rival_TreatmentWingClip -0.17349 0.07455 -2.33
Rival_TreatmentWingRemoved -0.25253 0.07523 -3.36
Week2 0.05154 0.03504 1.47
Week3 0.05480 0.03556 1.54
Week4 0.01442 0.03682 0.39
Week5 -0.22496 0.04046 -5.56
Focal_TreatmentWT:Rival_TreatmentWingClip -0.07046 0.11166 -0.63
Focal_TreatmentWT:Rival_TreatmentWingRemoved 0.32681 0.11095 2.95
 Pr(>|z|)
(Intercept) < 2e-16 ***
Focal_TreatmentWT 9.64e-15 ***
Rival_TreatmentWingClip 0.019966 *
Rival_TreatmentWingRemoved 0.000788 ***
Week2 0.141276
Week3 0.123325
Week4 0.695382
Week5 2.70e-08 ***
Focal_TreatmentWT:Rival_TreatmentWingClip 0.528033
Focal_TreatmentWT:Rival_TreatmentWingRemoved 0.003224 **
---
Signif. codes: 0 '***' 0.001 '**' 0.01 '*' 0.05 '.' 0.1 ' ' 1

lsmeans(OrcoInactive_glmmTMB1, pairwise ~ Focal_Treatment * Rival_Treatment * Week, adjust="tukey")

$lsmeans
 Focal_Treatment Rival_Treatment Week lsmean SE df asymp.LCL asymp.UCL
 Orco Single 1 2.023 0.0564 Inf 1.912 2.13
 WT Single 1 1.420 0.0617 Inf 1.299 1.54
 Orco WingClip 1 1.849 0.0577 Inf 1.736 1.96
 WT WingClip 1 1.176 0.0636 Inf 1.052 1.30
 Orco WingRemoved 1 1.770 0.0585 Inf 1.656 1.89
 WT WingRemoved 1 1.495 0.0612 Inf 1.375 1.61
 Orco Single 2 2.074 0.0565 Inf 1.964 2.19
 WT Single 2 1.472 0.0619 Inf 1.350 1.59
 Orco WingClip 2 1.901 0.0577 Inf 1.788 2.01
 WT WingClip 2 1.228 0.0637 Inf 1.103 1.35
 Orco WingRemoved 2 1.822 0.0587 Inf 1.707 1.94
 WT WingRemoved 2 1.546 0.0616 Inf 1.425 1.67
 Orco Single 3 2.078 0.0566 Inf 1.967 2.19
 WT Single 3 1.475 0.0624 Inf 1.353 1.60
 Orco WingClip 3 1.904 0.0580 Inf 1.790 2.02
 WT WingClip 3 1.231 0.0640 Inf 1.106 1.36
 Orco WingRemoved 3 1.825 0.0591 Inf 1.709 1.94
 WT WingRemoved 3 1.549 0.0619 Inf 1.428 1.67
 Orco Single 4 2.037 0.0572 Inf 1.925 2.15
 WT Single 4 1.435 0.0632 Inf 1.311 1.56
 Orco WingClip 4 1.864 0.0590 Inf 1.748 1.98
 WT WingClip 4 1.191 0.0645 Inf 1.064 1.32
 Orco WingRemoved 4 1.785 0.0599 Inf 1.667 1.90
 WT WingRemoved 4 1.509 0.0625 Inf 1.386 1.63
 Orco Single 5 1.798 0.0596 Inf 1.681 1.91
 WT Single 5 1.195 0.0654 Inf 1.067 1.32
 Orco WingClip 5 1.624 0.0615 Inf 1.504 1.74
 WT WingClip 5 0.951 0.0666 Inf 0.821 1.08
 Orco WingRemoved 5 1.545 0.0622 Inf 1.423 1.67
 WT WingRemoved 5 1.270 0.0647 Inf 1.143 1.40

Results are given on the log (not the response) scale.
Confidence level used: 0.95

$contrasts
 contrast estimate SE df z.ratio
 Orco Single Week1 - WT Single Week1 0.602547 0.0778 Inf 7.744
 Orco Single Week1 - Orco WingClip Week1 0.173486 0.0746 Inf 2.327
 Orco Single Week1 - WT WingClip Week1 0.846489 0.0791 Inf 10.706
 Orco Single Week1 - Orco WingRemoved Week1 0.252526 0.0752 Inf 3.357
 Orco Single Week1 - WT WingRemoved Week1 0.528264 0.0774 Inf 6.825
 Orco Single Week1 - Orco Single Week2 -0.051544 0.0350 Inf -1.471
 Orco Single Week1 - WT Single Week2 0.551002 0.0855 Inf 6.447
 Orco Single Week1 - Orco WingClip Week2 0.121942 0.0823 Inf 1.481
 Orco Single Week1 - WT WingClip Week2 0.794945 0.0865 Inf 9.188
 Orco Single Week1 - Orco WingRemoved Week2 0.200982 0.0831 Inf 2.419
 Orco Single Week1 - WT WingRemoved Week2 0.476720 0.0852 Inf 5.598
 Orco Single Week1 - Orco Single Week3 -0.054800 0.0356 Inf -1.541
 Orco Single Week1 - WT Single Week3 0.547746 0.0859 Inf 6.374
 Orco Single Week1 - Orco WingClip Week3 0.118686 0.0827 Inf 1.435
 Orco Single Week1 - WT WingClip Week3 0.791689 0.0869 Inf 9.115
 Orco Single Week1 - Orco WingRemoved Week3 0.197725 0.0835 Inf 2.369
 Orco Single Week1 - WT WingRemoved Week3 0.473463 0.0855 Inf 5.536
 Orco Single Week1 - Orco Single Week4 -0.014416 0.0368 Inf -0.392
 Orco Single Week1 - WT Single Week4 0.588131 0.0866 Inf 6.791
 Orco Single Week1 - Orco WingClip Week4 0.159071 0.0834 Inf 1.906
 Orco Single Week1 - WT WingClip Week4 0.832073 0.0873 Inf 9.526
 Orco Single Week1 - Orco WingRemoved Week4 0.238110 0.0841 Inf 2.830
 Orco Single Week1 - WT WingRemoved Week4 0.513848 0.0861 Inf 5.971
 Orco Single Week1 - Orco Single Week5 0.224957 0.0405 Inf 5.560
 Orco Single Week1 - WT Single Week5 0.827504 0.0883 Inf 9.374
 Orco Single Week1 - Orco WingClip Week5 0.398444 0.0853 Inf 4.670
 Orco Single Week1 - WT WingClip Week5 1.071446 0.0889 Inf 12.046
 Orco Single Week1 - Orco WingRemoved Week5 0.477483 0.0858 Inf 5.563
 Orco Single Week1 - WT WingRemoved Week5 0.753221 0.0877 Inf 8.584
 WT Single Week1 - Orco WingClip Week1 -0.429060 0.0789 Inf -5.440
 WT Single Week1 - WT WingClip Week1 0.243942 0.0831 Inf 2.935
 WT Single Week1 - Orco WingRemoved Week1 -0.350021 0.0795 Inf -4.405
 WT Single Week1 - WT WingRemoved Week1 -0.074283 0.0815 Inf -0.911
 WT Single Week1 - Orco Single Week2 -0.654091 0.0852 Inf -7.677
 WT Single Week1 - WT Single Week2 -0.051544 0.0350 Inf -1.471
 WT Single Week1 - Orco WingClip Week2 -0.480604 0.0861 Inf -5.581
 WT Single Week1 - WT WingClip Week2 0.192398 0.0901 Inf 2.136
 WT Single Week1 - Orco WingRemoved Week2 -0.401565 0.0868 Inf -4.627
 WT Single Week1 - WT WingRemoved Week2 -0.125827 0.0888 Inf -1.417
 WT Single Week1 - Orco Single Week3 -0.657347 0.0852 Inf -7.719
 WT Single Week1 - WT Single Week3 -0.054800 0.0356 Inf -1.541
 WT Single Week1 - Orco WingClip Week3 -0.483860 0.0862 Inf -5.613
 WT Single Week1 - WT WingClip Week3 0.189142 0.0902 Inf 2.097
 WT Single Week1 - Orco WingRemoved Week3 -0.404821 0.0869 Inf -4.657
 WT Single Week1 - WT WingRemoved Week3 -0.129083 0.0889 Inf -1.452
 WT Single Week1 - Orco Single Week4 -0.616962 0.0856 Inf -7.211
 WT Single Week1 - WT Single Week4 -0.014416 0.0368 Inf -0.392
 WT Single Week1 - Orco WingClip Week4 -0.443476 0.0868 Inf -5.109
 WT Single Week1 - WT WingClip Week4 0.229527 0.0905 Inf 2.536
 WT Single Week1 - Orco WingRemoved Week4 -0.364437 0.0874 Inf -4.169
 WT Single Week1 - WT WingRemoved Week4 -0.088699 0.0893 Inf -0.993
 WT Single Week1 - Orco Single Week5 -0.377589 0.0871 Inf -4.334
 WT Single Week1 - WT Single Week5 0.224957 0.0405 Inf 5.560
 WT Single Week1 - Orco WingClip Week5 -0.204103 0.0885 Inf -2.305
 WT Single Week1 - WT WingClip Week5 0.468900 0.0920 Inf 5.096
 WT Single Week1 - Orco WingRemoved Week5 -0.125064 0.0890 Inf -1.405
 WT Single Week1 - WT WingRemoved Week5 0.150674 0.0909 Inf 1.658
 Orco WingClip Week1 - WT WingClip Week1 0.673002 0.0801 Inf 8.400
 Orco WingClip Week1 - Orco WingRemoved Week1 0.079039 0.0763 Inf 1.036
 Orco WingClip Week1 - WT WingRemoved Week1 0.354777 0.0785 Inf 4.521
 Orco WingClip Week1 - Orco Single Week2 -0.225031 0.0824 Inf -2.730
 Orco WingClip Week1 - WT Single Week2 0.377516 0.0865 Inf 4.365
 Orco WingClip Week1 - Orco WingClip Week2 -0.051544 0.0350 Inf -1.471
 Orco WingClip Week1 - WT WingClip Week2 0.621458 0.0875 Inf 7.101
 Orco WingClip Week1 - Orco WingRemoved Week2 0.027495 0.0841 Inf 0.327
 Orco WingClip Week1 - WT WingRemoved Week2 0.303233 0.0862 Inf 3.518
 Orco WingClip Week1 - Orco Single Week3 -0.228287 0.0825 Inf -2.766
 Orco WingClip Week1 - WT Single Week3 0.374260 0.0868 Inf 4.311
 Orco WingClip Week1 - Orco WingClip Week3 -0.054800 0.0356 Inf -1.541
 Orco WingClip Week1 - WT WingClip Week3 0.618202 0.0877 Inf 7.046
 Orco WingClip Week1 - Orco WingRemoved Week3 0.024239 0.0844 Inf 0.287
 Orco WingClip Week1 - WT WingRemoved Week3 0.299977 0.0864 Inf 3.471
 Orco WingClip Week1 - Orco Single Week4 -0.187902 0.0829 Inf -2.268
 Orco WingClip Week1 - WT Single Week4 0.414644 0.0873 Inf 4.751
 Orco WingClip Week1 - Orco WingClip Week4 -0.014416 0.0368 Inf -0.392
 Orco WingClip Week1 - WT WingClip Week4 0.658587 0.0880 Inf 7.482
 Orco WingClip Week1 - Orco WingRemoved Week4 0.064623 0.0848 Inf 0.762
 Orco WingClip Week1 - WT WingRemoved Week4 0.340361 0.0868 Inf 3.923
 Orco WingClip Week1 - Orco Single Week5 0.051471 0.0843 Inf 0.610
 Orco WingClip Week1 - WT Single Week5 0.654017 0.0887 Inf 7.370
 Orco WingClip Week1 - Orco WingClip Week5 0.224957 0.0405 Inf 5.560
 Orco WingClip Week1 - WT WingClip Week5 0.897960 0.0894 Inf 10.042
 Orco WingClip Week1 - Orco WingRemoved Week5 0.303996 0.0863 Inf 3.522
 Orco WingClip Week1 - WT WingRemoved Week5 0.579734 0.0882 Inf 6.571
 WT WingClip Week1 - Orco WingRemoved Week1 -0.593963 0.0807 Inf -7.360
 WT WingClip Week1 - WT WingRemoved Week1 -0.318225 0.0827 Inf -3.846
 WT WingClip Week1 - Orco Single Week2 -0.898033 0.0864 Inf -10.388
 WT WingClip Week1 - WT Single Week2 -0.295486 0.0903 Inf -3.273
 WT WingClip Week1 - Orco WingClip Week2 -0.724546 0.0874 Inf -8.293
 WT WingClip Week1 - WT WingClip Week2 -0.051544 0.0350 Inf -1.471
 WT WingClip Week1 - Orco WingRemoved Week2 -0.645507 0.0880 Inf -7.333
 WT WingClip Week1 - WT WingRemoved Week2 -0.369769 0.0900 Inf -4.108
 WT WingClip Week1 - Orco Single Week3 -0.901289 0.0865 Inf -10.415
 WT WingClip Week1 - WT Single Week3 -0.298743 0.0906 Inf -3.297
 WT WingClip Week1 - Orco WingClip Week3 -0.727803 0.0876 Inf -8.311
 WT WingClip Week1 - WT WingClip Week3 -0.054800 0.0356 Inf -1.541
 WT WingClip Week1 - Orco WingRemoved Week3 -0.648764 0.0883 Inf -7.349
 WT WingClip Week1 - WT WingRemoved Week3 -0.373026 0.0902 Inf -4.134
 WT WingClip Week1 - Orco Single Week4 -0.860905 0.0871 Inf -9.885
 WT WingClip Week1 - WT Single Week4 -0.258358 0.0913 Inf -2.831
 WT WingClip Week1 - Orco WingClip Week4 -0.687418 0.0883 Inf -7.783
 WT WingClip Week1 - WT WingClip Week4 -0.014416 0.0368 Inf -0.392
 WT WingClip Week1 - Orco WingRemoved Week4 -0.608379 0.0889 Inf -6.841
 WT WingClip Week1 - WT WingRemoved Week4 -0.332641 0.0908 Inf -3.665
 WT WingClip Week1 - Orco Single Week5 -0.621532 0.0887 Inf -7.008
 WT WingClip Week1 - WT Single Week5 -0.018985 0.0929 Inf -0.204
 WT WingClip Week1 - Orco WingClip Week5 -0.448045 0.0901 Inf -4.973
 WT WingClip Week1 - WT WingClip Week5 0.224957 0.0405 Inf 5.560
 WT WingClip Week1 - Orco WingRemoved Week5 -0.369006 0.0905 Inf -4.075
 WT WingClip Week1 - WT WingRemoved Week5 -0.093268 0.0924 Inf -1.010
 Orco WingRemoved Week1 - WT WingRemoved Week1 0.275738 0.0791 Inf 3.487
 Orco WingRemoved Week1 - Orco Single Week2 -0.304070 0.0829 Inf -3.668
 Orco WingRemoved Week1 - WT Single Week2 0.298477 0.0869 Inf 3.435
 Orco WingRemoved Week1 - Orco WingClip Week2 -0.130583 0.0839 Inf -1.557
 Orco WingRemoved Week1 - WT WingClip Week2 0.542419 0.0879 Inf 6.169
 Orco WingRemoved Week1 - Orco WingRemoved Week2 -0.051544 0.0350 Inf -1.471
 Orco WingRemoved Week1 - WT WingRemoved Week2 0.224194 0.0866 Inf 2.588
 Orco WingRemoved Week1 - Orco Single Week3 -0.307326 0.0829 Inf -3.705
 Orco WingRemoved Week1 - WT Single Week3 0.295221 0.0872 Inf 3.386
 Orco WingRemoved Week1 - Orco WingClip Week3 -0.133839 0.0840 Inf -1.593
 Orco WingRemoved Week1 - WT WingClip Week3 0.539163 0.0881 Inf 6.120
 Orco WingRemoved Week1 - Orco WingRemoved Week3 -0.054800 0.0356 Inf -1.541
 Orco WingRemoved Week1 - WT WingRemoved Week3 0.220938 0.0868 Inf 2.545
 Orco WingRemoved Week1 - Orco Single Week4 -0.266941 0.0834 Inf -3.202
 Orco WingRemoved Week1 - WT Single Week4 0.335605 0.0877 Inf 3.825
 Orco WingRemoved Week1 - Orco WingClip Week4 -0.093455 0.0847 Inf -1.104
 Orco WingRemoved Week1 - WT WingClip Week4 0.579547 0.0885 Inf 6.551
 Orco WingRemoved Week1 - Orco WingRemoved Week4 -0.014416 0.0368 Inf -0.392
 Orco WingRemoved Week1 - WT WingRemoved Week4 0.261322 0.0872 Inf 2.996
 Orco WingRemoved Week1 - Orco Single Week5 -0.027568 0.0850 Inf -0.324
 Orco WingRemoved Week1 - WT Single Week5 0.574978 0.0893 Inf 6.436
 Orco WingRemoved Week1 - Orco WingClip Week5 0.145918 0.0865 Inf 1.688
 Orco WingRemoved Week1 - WT WingClip Week5 0.818921 0.0900 Inf 9.099
 Orco WingRemoved Week1 - Orco WingRemoved Week5 0.224957 0.0405 Inf 5.560
 Orco WingRemoved Week1 - WT WingRemoved Week5 0.500695 0.0888 Inf 5.636
 WT WingRemoved Week1 - Orco Single Week2 -0.579808 0.0848 Inf -6.841
 WT WingRemoved Week1 - WT Single Week2 0.022739 0.0887 Inf 0.256
 WT WingRemoved Week1 - Orco WingClip Week2 -0.406321 0.0857 Inf -4.741
 WT WingRemoved Week1 - WT WingClip Week2 0.266681 0.0897 Inf 2.973
 WT WingRemoved Week1 - Orco WingRemoved Week2 -0.327282 0.0864 Inf -3.789
 WT WingRemoved Week1 - WT WingRemoved Week2 -0.051544 0.0350 Inf -1.471
 WT WingRemoved Week1 - Orco Single Week3 -0.583064 0.0848 Inf -6.873
 WT WingRemoved Week1 - WT Single Week3 0.019483 0.0890 Inf 0.219
 WT WingRemoved Week1 - Orco WingClip Week3 -0.409577 0.0859 Inf -4.769
 WT WingRemoved Week1 - WT WingClip Week3 0.263425 0.0899 Inf 2.931
 WT WingRemoved Week1 - Orco WingRemoved Week3 -0.330538 0.0866 Inf -3.816
 WT WingRemoved Week1 - WT WingRemoved Week3 -0.054800 0.0356 Inf -1.541
 WT WingRemoved Week1 - Orco Single Week4 -0.542679 0.0854 Inf -6.358
 WT WingRemoved Week1 - WT Single Week4 0.059867 0.0896 Inf 0.668
 WT WingRemoved Week1 - Orco WingClip Week4 -0.369193 0.0866 Inf -4.263
 WT WingRemoved Week1 - WT WingClip Week4 0.303810 0.0903 Inf 3.363
 WT WingRemoved Week1 - Orco WingRemoved Week4 -0.290154 0.0872 Inf -3.326
 WT WingRemoved Week1 - WT WingRemoved Week4 -0.014416 0.0368 Inf -0.392
 WT WingRemoved Week1 - Orco Single Week5 -0.303306 0.0869 Inf -3.489
 WT WingRemoved Week1 - WT Single Week5 0.299240 0.0912 Inf 3.282
 WT WingRemoved Week1 - Orco WingClip Week5 -0.129820 0.0884 Inf -1.469
 WT WingRemoved Week1 - WT WingClip Week5 0.543183 0.0918 Inf 5.915
 WT WingRemoved Week1 - Orco WingRemoved Week5 -0.050781 0.0888 Inf -0.572
 WT WingRemoved Week1 - WT WingRemoved Week5 0.224957 0.0405 Inf 5.560
 Orco Single Week2 - WT Single Week2 0.602547 0.0778 Inf 7.744
 Orco Single Week2 - Orco WingClip Week2 0.173486 0.0746 Inf 2.327
 Orco Single Week2 - WT WingClip Week2 0.846489 0.0791 Inf 10.706
 Orco Single Week2 - Orco WingRemoved Week2 0.252526 0.0752 Inf 3.357
 Orco Single Week2 - WT WingRemoved Week2 0.528264 0.0774 Inf 6.825
 Orco Single Week2 - Orco Single Week3 -0.003256 0.0354 Inf -0.092
 Orco Single Week2 - WT Single Week3 0.599290 0.0857 Inf 6.990
 Orco Single Week2 - Orco WingClip Week3 0.170230 0.0827 Inf 2.060
 Orco Single Week2 - WT WingClip Week3 0.843233 0.0868 Inf 9.720
 Orco Single Week2 - Orco WingRemoved Week3 0.249270 0.0833 Inf 2.992
 Orco Single Week2 - WT WingRemoved Week3 0.525007 0.0852 Inf 6.158
 Orco Single Week2 - Orco Single Week4 0.037128 0.0367 Inf 1.013
 Orco Single Week2 - WT Single Week4 0.639675 0.0864 Inf 7.404
 Orco Single Week2 - Orco WingClip Week4 0.210615 0.0834 Inf 2.525
 Orco Single Week2 - WT WingClip Week4 0.883617 0.0872 Inf 10.128
 Orco Single Week2 - Orco WingRemoved Week4 0.289654 0.0840 Inf 3.450
 Orco Single Week2 - WT WingRemoved Week4 0.565392 0.0858 Inf 6.590
 Orco Single Week2 - Orco Single Week5 0.276501 0.0403 Inf 6.859
 Orco Single Week2 - WT Single Week5 0.879048 0.0881 Inf 9.981
 Orco Single Week2 - Orco WingClip Week5 0.449988 0.0853 Inf 5.276
 Orco Single Week2 - WT WingClip Week5 1.122990 0.0888 Inf 12.639
 Orco Single Week2 - Orco WingRemoved Week5 0.529027 0.0857 Inf 6.174
 Orco Single Week2 - WT WingRemoved Week5 0.804765 0.0875 Inf 9.199
 WT Single Week2 - Orco WingClip Week2 -0.429060 0.0789 Inf -5.440
 WT Single Week2 - WT WingClip Week2 0.243942 0.0831 Inf 2.935
 WT Single Week2 - Orco WingRemoved Week2 -0.350021 0.0795 Inf -4.405
 WT Single Week2 - WT WingRemoved Week2 -0.074283 0.0815 Inf -0.911
 WT Single Week2 - Orco Single Week3 -0.605803 0.0852 Inf -7.108
 WT Single Week2 - WT Single Week3 -0.003256 0.0354 Inf -0.092
 WT Single Week2 - Orco WingClip Week3 -0.432316 0.0863 Inf -5.009
 WT Single Week2 - WT WingClip Week3 0.240686 0.0902 Inf 2.668
 WT Single Week2 - Orco WingRemoved Week3 -0.353277 0.0869 Inf -4.065
 WT Single Week2 - WT WingRemoved Week3 -0.077539 0.0888 Inf -0.873
 WT Single Week2 - Orco Single Week4 -0.565418 0.0856 Inf -6.603
 WT Single Week2 - WT Single Week4 0.037128 0.0367 Inf 1.013
 WT Single Week2 - Orco WingClip Week4 -0.391932 0.0869 Inf -4.510
 WT Single Week2 - WT WingClip Week4 0.281071 0.0906 Inf 3.104
 WT Single Week2 - Orco WingRemoved Week4 -0.312893 0.0874 Inf -3.580
 WT Single Week2 - WT WingRemoved Week4 -0.037155 0.0892 Inf -0.417
 WT Single Week2 - Orco Single Week5 -0.326045 0.0872 Inf -3.740
 WT Single Week2 - WT Single Week5 0.276501 0.0403 Inf 6.859
 WT Single Week2 - Orco WingClip Week5 -0.152559 0.0886 Inf -1.721
 WT Single Week2 - WT WingClip Week5 0.520444 0.0920 Inf 5.655
 WT Single Week2 - Orco WingRemoved Week5 -0.073520 0.0890 Inf -0.826
 WT Single Week2 - WT WingRemoved Week5 0.202218 0.0907 Inf 2.229
 Orco WingClip Week2 - WT WingClip Week2 0.673002 0.0801 Inf 8.400
 Orco WingClip Week2 - Orco WingRemoved Week2 0.079039 0.0763 Inf 1.036
 Orco WingClip Week2 - WT WingRemoved Week2 0.354777 0.0785 Inf 4.521
 Orco WingClip Week2 - Orco Single Week3 -0.176743 0.0824 Inf -2.145
 Orco WingClip Week2 - WT Single Week3 0.425804 0.0866 Inf 4.918
 Orco WingClip Week2 - Orco WingClip Week3 -0.003256 0.0354 Inf -0.092
 Orco WingClip Week2 - WT WingClip Week3 0.669746 0.0876 Inf 7.646
 Orco WingClip Week2 - Orco WingRemoved Week3 0.075783 0.0842 Inf 0.900
 Orco WingClip Week2 - WT WingRemoved Week3 0.351521 0.0861 Inf 4.082
 Orco WingClip Week2 - Orco Single Week4 -0.136358 0.0827 Inf -1.648
 Orco WingClip Week2 - WT Single Week4 0.466188 0.0870 Inf 5.357
 Orco WingClip Week2 - Orco WingClip Week4 0.037128 0.0367 Inf 1.013
 Orco WingClip Week2 - WT WingClip Week4 0.710131 0.0879 Inf 8.080
 Orco WingClip Week2 - Orco WingRemoved Week4 0.116167 0.0846 Inf 1.373
 Orco WingClip Week2 - WT WingRemoved Week4 0.391905 0.0864 Inf 4.534
 Orco WingClip Week2 - Orco Single Week5 0.103015 0.0842 Inf 1.223
 Orco WingClip Week2 - WT Single Week5 0.705561 0.0885 Inf 7.972
 Orco WingClip Week2 - Orco WingClip Week5 0.276501 0.0403 Inf 6.859
 Orco WingClip Week2 - WT WingClip Week5 0.949504 0.0893 Inf 10.635
 Orco WingClip Week2 - Orco WingRemoved Week5 0.355540 0.0861 Inf 4.128
 Orco WingClip Week2 - WT WingRemoved Week5 0.631278 0.0879 Inf 7.180
 WT WingClip Week2 - Orco WingRemoved Week2 -0.593963 0.0807 Inf -7.360
 WT WingClip Week2 - WT WingRemoved Week2 -0.318225 0.0827 Inf -3.846
 WT WingClip Week2 - Orco Single Week3 -0.849745 0.0865 Inf -9.824
 WT WingClip Week2 - WT Single Week3 -0.247199 0.0904 Inf -2.733
 WT WingClip Week2 - Orco WingClip Week3 -0.676259 0.0876 Inf -7.722
 WT WingClip Week2 - WT WingClip Week3 -0.003256 0.0354 Inf -0.092
 WT WingClip Week2 - Orco WingRemoved Week3 -0.597220 0.0882 Inf -6.774
 WT WingClip Week2 - WT WingRemoved Week3 -0.321482 0.0900 Inf -3.572
 WT WingClip Week2 - Orco Single Week4 -0.809361 0.0871 Inf -9.297
 WT WingClip Week2 - WT Single Week4 -0.206814 0.0911 Inf -2.270
 WT WingClip Week2 - Orco WingClip Week4 -0.635874 0.0883 Inf -7.199
 WT WingClip Week2 - WT WingClip Week4 0.037128 0.0367 Inf 1.013
 WT WingClip Week2 - Orco WingRemoved Week4 -0.556835 0.0888 Inf -6.270
 WT WingClip Week2 - WT WingRemoved Week4 -0.281097 0.0905 Inf -3.104
 WT WingClip Week2 - Orco Single Week5 -0.569988 0.0887 Inf -6.429
 WT WingClip Week2 - WT Single Week5 0.032559 0.0927 Inf 0.351
 WT WingClip Week2 - Orco WingClip Week5 -0.396501 0.0901 Inf -4.401
 WT WingClip Week2 - WT WingClip Week5 0.276501 0.0403 Inf 6.859
 WT WingClip Week2 - Orco WingRemoved Week5 -0.317462 0.0904 Inf -3.511
 WT WingClip Week2 - WT WingRemoved Week5 -0.041724 0.0921 Inf -0.453
 Orco WingRemoved Week2 - WT WingRemoved Week2 0.275738 0.0791 Inf 3.487
 Orco WingRemoved Week2 - Orco Single Week3 -0.255782 0.0830 Inf -3.083
 Orco WingRemoved Week2 - WT Single Week3 0.346765 0.0871 Inf 3.983
 Orco WingRemoved Week2 - Orco WingClip Week3 -0.082295 0.0841 Inf -0.979
 Orco WingRemoved Week2 - WT WingClip Week3 0.590707 0.0881 Inf 6.706
 Orco WingRemoved Week2 - Orco WingRemoved Week3 -0.003256 0.0354 Inf -0.092
 Orco WingRemoved Week2 - WT WingRemoved Week3 0.272482 0.0866 Inf 3.146
 Orco WingRemoved Week2 - Orco Single Week4 -0.215397 0.0834 Inf -2.583
 Orco WingRemoved Week2 - WT Single Week4 0.387149 0.0876 Inf 4.419
 Orco WingRemoved Week2 - Orco WingClip Week4 -0.041911 0.0847 Inf -0.495
 Orco WingRemoved Week2 - WT WingClip Week4 0.631092 0.0885 Inf 7.134
 Orco WingRemoved Week2 - Orco WingRemoved Week4 0.037128 0.0367 Inf 1.013
 Orco WingRemoved Week2 - WT WingRemoved Week4 0.312866 0.0870 Inf 3.595
 Orco WingRemoved Week2 - Orco Single Week5 0.023976 0.0850 Inf 0.282
 Orco WingRemoved Week2 - WT Single Week5 0.626522 0.0892 Inf 7.022
 Orco WingRemoved Week2 - Orco WingClip Week5 0.197462 0.0865 Inf 2.282
 Orco WingRemoved Week2 - WT WingClip Week5 0.870465 0.0900 Inf 9.673
 Orco WingRemoved Week2 - Orco WingRemoved Week5 0.276501 0.0403 Inf 6.859
 Orco WingRemoved Week2 - WT WingRemoved Week5 0.552239 0.0887 Inf 6.229
 WT WingRemoved Week2 - Orco Single Week3 -0.531520 0.0850 Inf -6.256
 WT WingRemoved Week2 - WT Single Week3 0.071027 0.0890 Inf 0.798
 WT WingRemoved Week2 - Orco WingClip Week3 -0.358033 0.0861 Inf -4.160
 WT WingRemoved Week2 - WT WingClip Week3 0.314969 0.0900 Inf 3.500
 WT WingRemoved Week2 - Orco WingRemoved Week3 -0.278994 0.0867 Inf -3.219
 WT WingRemoved Week2 - WT WingRemoved Week3 -0.003256 0.0354 Inf -0.092
 WT WingRemoved Week2 - Orco Single Week4 -0.491135 0.0855 Inf -5.745
 WT WingRemoved Week2 - WT Single Week4 0.111411 0.0896 Inf 1.243
 WT WingRemoved Week2 - Orco WingClip Week4 -0.317649 0.0868 Inf -3.660
 WT WingRemoved Week2 - WT WingClip Week4 0.355354 0.0904 Inf 3.929
 WT WingRemoved Week2 - Orco WingRemoved Week4 -0.238610 0.0873 Inf -2.734
 WT WingRemoved Week2 - WT WingRemoved Week4 0.037128 0.0367 Inf 1.013
 WT WingRemoved Week2 - Orco Single Week5 -0.251762 0.0871 Inf -2.892
 WT WingRemoved Week2 - WT Single Week5 0.350784 0.0912 Inf 3.847
 WT WingRemoved Week2 - Orco WingClip Week5 -0.078276 0.0885 Inf -0.884
 WT WingRemoved Week2 - WT WingClip Week5 0.594727 0.0919 Inf 6.469
 WT WingRemoved Week2 - Orco WingRemoved Week5 0.000763 0.0889 Inf 0.009
 WT WingRemoved Week2 - WT WingRemoved Week5 0.276501 0.0403 Inf 6.859
 Orco Single Week3 - WT Single Week3 0.602547 0.0778 Inf 7.744
 Orco Single Week3 - Orco WingClip Week3 0.173486 0.0746 Inf 2.327
 Orco Single Week3 - WT WingClip Week3 0.846489 0.0791 Inf 10.706
 Orco Single Week3 - Orco WingRemoved Week3 0.252526 0.0752 Inf 3.357
 Orco Single Week3 - WT WingRemoved Week3 0.528264 0.0774 Inf 6.825
 Orco Single Week3 - Orco Single Week4 0.040384 0.0369 Inf 1.094
 Orco Single Week3 - WT Single Week4 0.642931 0.0863 Inf 7.454
 Orco Single Week3 - Orco WingClip Week4 0.213871 0.0834 Inf 2.564
 Orco Single Week3 - WT WingClip Week4 0.886873 0.0872 Inf 10.168
 Orco Single Week3 - Orco WingRemoved Week4 0.292910 0.0839 Inf 3.491
 Orco Single Week3 - WT WingRemoved Week4 0.568648 0.0858 Inf 6.631
 Orco Single Week3 - Orco Single Week5 0.279757 0.0406 Inf 6.897
 Orco Single Week3 - WT Single Week5 0.882304 0.0879 Inf 10.033
 Orco Single Week3 - Orco WingClip Week5 0.453244 0.0853 Inf 5.314
 Orco Single Week3 - WT WingClip Week5 1.126246 0.0888 Inf 12.678
 Orco Single Week3 - Orco WingRemoved Week5 0.532283 0.0856 Inf 6.216
 Orco Single Week3 - WT WingRemoved Week5 0.808021 0.0875 Inf 9.239
 WT Single Week3 - Orco WingClip Week3 -0.429060 0.0789 Inf -5.440
 WT Single Week3 - WT WingClip Week3 0.243942 0.0831 Inf 2.935
 WT Single Week3 - Orco WingRemoved Week3 -0.350021 0.0795 Inf -4.405
 WT Single Week3 - WT WingRemoved Week3 -0.074283 0.0815 Inf -0.911
 WT Single Week3 - Orco Single Week4 -0.562162 0.0860 Inf -6.538
 WT Single Week3 - WT Single Week4 0.040384 0.0369 Inf 1.094
 WT Single Week3 - Orco WingClip Week4 -0.388676 0.0871 Inf -4.460
 WT Single Week3 - WT WingClip Week4 0.284327 0.0908 Inf 3.132
 WT Single Week3 - Orco WingRemoved Week4 -0.309637 0.0876 Inf -3.535
 WT Single Week3 - WT WingRemoved Week4 -0.033899 0.0894 Inf -0.379
 WT Single Week3 - Orco Single Week5 -0.322789 0.0875 Inf -3.687
 WT Single Week3 - WT Single Week5 0.279757 0.0406 Inf 6.897
 WT Single Week3 - Orco WingClip Week5 -0.149303 0.0889 Inf -1.680
 WT Single Week3 - WT WingClip Week5 0.523700 0.0923 Inf 5.676
 WT Single Week3 - Orco WingRemoved Week5 -0.070264 0.0892 Inf -0.788
 WT Single Week3 - WT WingRemoved Week5 0.205474 0.0909 Inf 2.259
 Orco WingClip Week3 - WT WingClip Week3 0.673002 0.0801 Inf 8.400
 Orco WingClip Week3 - Orco WingRemoved Week3 0.079039 0.0763 Inf 1.036
 Orco WingClip Week3 - WT WingRemoved Week3 0.354777 0.0785 Inf 4.521
 Orco WingClip Week3 - Orco Single Week4 -0.133102 0.0830 Inf -1.604
 Orco WingClip Week3 - WT Single Week4 0.469444 0.0870 Inf 5.395
 Orco WingClip Week3 - Orco WingClip Week4 0.040384 0.0369 Inf 1.094
 Orco WingClip Week3 - WT WingClip Week4 0.713387 0.0880 Inf 8.109
 Orco WingClip Week3 - Orco WingRemoved Week4 0.119423 0.0847 Inf 1.410
 Orco WingClip Week3 - WT WingRemoved Week4 0.395161 0.0865 Inf 4.567
 Orco WingClip Week3 - Orco Single Week5 0.106271 0.0845 Inf 1.258
 Orco WingClip Week3 - WT Single Week5 0.708817 0.0885 Inf 8.011
 Orco WingClip Week3 - Orco WingClip Week5 0.279757 0.0406 Inf 6.897
 Orco WingClip Week3 - WT WingClip Week5 0.952760 0.0894 Inf 10.659
 Orco WingClip Week3 - Orco WingRemoved Week5 0.358797 0.0862 Inf 4.163
 Orco WingClip Week3 - WT WingRemoved Week5 0.634534 0.0880 Inf 7.209
 WT WingClip Week3 - Orco WingRemoved Week3 -0.593963 0.0807 Inf -7.360
 WT WingClip Week3 - WT WingRemoved Week3 -0.318225 0.0827 Inf -3.846
 WT WingClip Week3 - Orco Single Week4 -0.806105 0.0873 Inf -9.235
 WT WingClip Week3 - WT Single Week4 -0.203558 0.0911 Inf -2.235
 WT WingClip Week3 - Orco WingClip Week4 -0.632618 0.0884 Inf -7.153
 WT WingClip Week3 - WT WingClip Week4 0.040384 0.0369 Inf 1.094
 WT WingClip Week3 - Orco WingRemoved Week4 -0.553579 0.0889 Inf -6.229
 WT WingClip Week3 - WT WingRemoved Week4 -0.277841 0.0906 Inf -3.065
 WT WingClip Week3 - Orco Single Week5 -0.566732 0.0889 Inf -6.376
 WT WingClip Week3 - WT Single Week5 0.035815 0.0927 Inf 0.386
 WT WingClip Week3 - Orco WingClip Week5 -0.393245 0.0902 Inf -4.359
 WT WingClip Week3 - WT WingClip Week5 0.279757 0.0406 Inf 6.897
 WT WingClip Week3 - Orco WingRemoved Week5 -0.314206 0.0905 Inf -3.472
 WT WingClip Week3 - WT WingRemoved Week5 -0.038468 0.0922 Inf -0.417
 Orco WingRemoved Week3 - WT WingRemoved Week3 0.275738 0.0791 Inf 3.487
 Orco WingRemoved Week3 - Orco Single Week4 -0.212141 0.0837 Inf -2.535
 Orco WingRemoved Week3 - WT Single Week4 0.390405 0.0876 Inf 4.454
 Orco WingRemoved Week3 - Orco WingClip Week4 -0.038655 0.0849 Inf -0.455
 Orco WingRemoved Week3 - WT WingClip Week4 0.634348 0.0886 Inf 7.159
 Orco WingRemoved Week3 - Orco WingRemoved Week4 0.040384 0.0369 Inf 1.094
 Orco WingRemoved Week3 - WT WingRemoved Week4 0.316122 0.0872 Inf 3.626
 Orco WingRemoved Week3 - Orco Single Week5 0.027232 0.0853 Inf 0.319
 Orco WingRemoved Week3 - WT Single Week5 0.629778 0.0893 Inf 7.056
 Orco WingRemoved Week3 - Orco WingClip Week5 0.200718 0.0867 Inf 2.316
 Orco WingRemoved Week3 - WT WingClip Week5 0.873721 0.0901 Inf 9.693
 Orco WingRemoved Week3 - Orco WingRemoved Week5 0.279757 0.0406 Inf 6.897
 Orco WingRemoved Week3 - WT WingRemoved Week5 0.555495 0.0888 Inf 6.256
 WT WingRemoved Week3 - Orco Single Week4 -0.487879 0.0857 Inf -5.690
 WT WingRemoved Week3 - WT Single Week4 0.114667 0.0896 Inf 1.279
 WT WingRemoved Week3 - Orco WingClip Week4 -0.314393 0.0869 Inf -3.617
 WT WingRemoved Week3 - WT WingClip Week4 0.358610 0.0906 Inf 3.960
 WT WingRemoved Week3 - Orco WingRemoved Week4 -0.235354 0.0874 Inf -2.694
 WT WingRemoved Week3 - WT WingRemoved Week4 0.040384 0.0369 Inf 1.094
 WT WingRemoved Week3 - Orco Single Week5 -0.248506 0.0873 Inf -2.846
 WT WingRemoved Week3 - WT Single Week5 0.354040 0.0912 Inf 3.883
 WT WingRemoved Week3 - Orco WingClip Week5 -0.075020 0.0887 Inf -0.846
 WT WingRemoved Week3 - WT WingClip Week5 0.597983 0.0920 Inf 6.497
 WT WingRemoved Week3 - Orco WingRemoved Week5 0.004019 0.0890 Inf 0.045
 WT WingRemoved Week3 - WT WingRemoved Week5 0.279757 0.0406 Inf 6.897
 Orco Single Week4 - WT Single Week4 0.602547 0.0778 Inf 7.744
 Orco Single Week4 - Orco WingClip Week4 0.173486 0.0746 Inf 2.327
 Orco Single Week4 - WT WingClip Week4 0.846489 0.0791 Inf 10.706
 Orco Single Week4 - Orco WingRemoved Week4 0.252526 0.0752 Inf 3.357
 Orco Single Week4 - WT WingRemoved Week4 0.528264 0.0774 Inf 6.825
 Orco Single Week4 - Orco Single Week5 0.239373 0.0414 Inf 5.785
 Orco Single Week4 - WT Single Week5 0.841920 0.0882 Inf 9.547
 Orco Single Week4 - Orco WingClip Week5 0.412860 0.0855 Inf 4.830
 Orco Single Week4 - WT WingClip Week5 1.085862 0.0892 Inf 12.167
 Orco Single Week4 - Orco WingRemoved Week5 0.491899 0.0859 Inf 5.726
 Orco Single Week4 - WT WingRemoved Week5 0.767637 0.0878 Inf 8.740
 WT Single Week4 - Orco WingClip Week4 -0.429060 0.0789 Inf -5.440
 WT Single Week4 - WT WingClip Week4 0.243942 0.0831 Inf 2.935
 WT Single Week4 - Orco WingRemoved Week4 -0.350021 0.0795 Inf -4.405
 WT Single Week4 - WT WingRemoved Week4 -0.074283 0.0815 Inf -0.911
 WT Single Week4 - Orco Single Week5 -0.363174 0.0881 Inf -4.124
 WT Single Week4 - WT Single Week5 0.239373 0.0414 Inf 5.785
 WT Single Week4 - Orco WingClip Week5 -0.189687 0.0892 Inf -2.127
 WT Single Week4 - WT WingClip Week5 0.483316 0.0928 Inf 5.209
 WT Single Week4 - Orco WingRemoved Week5 -0.110648 0.0896 Inf -1.235
 WT Single Week4 - WT WingRemoved Week5 0.165090 0.0914 Inf 1.806
 Orco WingClip Week4 - WT WingClip Week4 0.673002 0.0801 Inf 8.400
 Orco WingClip Week4 - Orco WingRemoved Week4 0.079039 0.0763 Inf 1.036
 Orco WingClip Week4 - WT WingRemoved Week4 0.354777 0.0785 Inf 4.521
 Orco WingClip Week4 - Orco Single Week5 0.065887 0.0851 Inf 0.775
 Orco WingClip Week4 - WT Single Week5 0.668433 0.0889 Inf 7.516
 Orco WingClip Week4 - Orco WingClip Week5 0.239373 0.0414 Inf 5.785
 Orco WingClip Week4 - WT WingClip Week5 0.912376 0.0900 Inf 10.139
 Orco WingClip Week4 - Orco WingRemoved Week5 0.318412 0.0867 Inf 3.674
 Orco WingClip Week4 - WT WingRemoved Week5 0.594150 0.0886 Inf 6.707
 WT WingClip Week4 - Orco WingRemoved Week4 -0.593963 0.0807 Inf -7.360
 WT WingClip Week4 - WT WingRemoved Week4 -0.318225 0.0827 Inf -3.846
 WT WingClip Week4 - Orco Single Week5 -0.607116 0.0892 Inf -6.804
 WT WingClip Week4 - WT Single Week5 -0.004569 0.0929 Inf -0.049
 WT WingClip Week4 - Orco WingClip Week5 -0.433630 0.0904 Inf -4.799
 WT WingClip Week4 - WT WingClip Week5 0.239373 0.0414 Inf 5.785
 WT WingClip Week4 - Orco WingRemoved Week5 -0.354590 0.0907 Inf -3.908
 WT WingClip Week4 - WT WingRemoved Week5 -0.078852 0.0926 Inf -0.852
 Orco WingRemoved Week4 - WT WingRemoved Week4 0.275738 0.0791 Inf 3.487
 Orco WingRemoved Week4 - Orco Single Week5 -0.013153 0.0858 Inf -0.153
 Orco WingRemoved Week4 - WT Single Week5 0.589394 0.0896 Inf 6.578
 Orco WingRemoved Week4 - Orco WingClip Week5 0.160334 0.0870 Inf 1.844
 Orco WingRemoved Week4 - WT WingClip Week5 0.833336 0.0906 Inf 9.194
 Orco WingRemoved Week4 - Orco WingRemoved Week5 0.239373 0.0414 Inf 5.785
 Orco WingRemoved Week4 - WT WingRemoved Week5 0.515111 0.0893 Inf 5.770
 WT WingRemoved Week4 - Orco Single Week5 -0.288890 0.0877 Inf -3.294
 WT WingRemoved Week4 - WT Single Week5 0.313656 0.0914 Inf 3.431
 WT WingRemoved Week4 - Orco WingClip Week5 -0.115404 0.0888 Inf -1.299
 WT WingRemoved Week4 - WT WingClip Week5 0.557598 0.0925 Inf 6.031
 WT WingRemoved Week4 - Orco WingRemoved Week5 -0.036365 0.0892 Inf -0.407
 WT WingRemoved Week4 - WT WingRemoved Week5 0.239373 0.0414 Inf 5.785
 Orco Single Week5 - WT Single Week5 0.602547 0.0778 Inf 7.744
 Orco Single Week5 - Orco WingClip Week5 0.173486 0.0746 Inf 2.327
 Orco Single Week5 - WT WingClip Week5 0.846489 0.0791 Inf 10.706
 Orco Single Week5 - Orco WingRemoved Week5 0.252526 0.0752 Inf 3.357
 Orco Single Week5 - WT WingRemoved Week5 0.528264 0.0774 Inf 6.825
 WT Single Week5 - Orco WingClip Week5 -0.429060 0.0789 Inf -5.440
 WT Single Week5 - WT WingClip Week5 0.243942 0.0831 Inf 2.935
 WT Single Week5 - Orco WingRemoved Week5 -0.350021 0.0795 Inf -4.405
 WT Single Week5 - WT WingRemoved Week5 -0.074283 0.0815 Inf -0.911
 Orco WingClip Week5 - WT WingClip Week5 0.673002 0.0801 Inf 8.400
 Orco WingClip Week5 - Orco WingRemoved Week5 0.079039 0.0763 Inf 1.036
 Orco WingClip Week5 - WT WingRemoved Week5 0.354777 0.0785 Inf 4.521
 WT WingClip Week5 - Orco WingRemoved Week5 -0.593963 0.0807 Inf -7.360
 WT WingClip Week5 - WT WingRemoved Week5 -0.318225 0.0827 Inf -3.846
 Orco WingRemoved Week5 - WT WingRemoved Week5 0.275738 0.0791 Inf 3.487
 p.value
 <.0001
 0.8824
 <.0001
 0.1662
 <.0001
 0.9999
 <.0001
 0.9998
 <.0001
 0.8327
 <.0001
 0.9997
 <.0001
 0.9999
 <.0001
 0.8612
 <.0001
 1.0000
 <.0001
 0.9891
 <.0001
 0.5202
 <.0001
 <.0001
 <.0001
 0.0012
 <.0001
 <.0001
 <.0001
 <.0001
 0.4346
 0.0039
 1.0000
 <.0001
 0.9999
 <.0001
 0.9526
 0.0014
 0.9999
 <.0001
 0.9997
 <.0001
 0.9618
 0.0012
 0.9999
 <.0001
 1.0000
 0.0001
 0.7562
 0.0104
 1.0000
 0.0053
 <.0001
 0.8926
 0.0001
 0.9999
 0.9988
 <.0001
 1.0000
 0.0023
 0.6037
 0.0046
 0.9999
 <.0001
 1.0000
 0.1047
 0.5736
 0.0058
 0.9997
 <.0001
 1.0000
 0.1205
 0.9088
 0.0008
 1.0000
 <.0001
 1.0000
 0.0269
 1.0000
 <.0001
 <.0001
 <.0001
 0.1036
 <.0001
 <.0001
 0.0356
 <.0001
 0.2073
 <.0001
 0.9999
 <.0001
 0.0133
 <.0001
 0.1947
 <.0001
 0.9997
 <.0001
 0.0120
 <.0001
 0.5199
 <.0001
 1.0000
 <.0001
 0.0662
 <.0001
 1.0000
 0.0003
 <.0001
 0.0151
 1.0000
 0.1150
 0.0654
 0.1337
 0.9996
 <.0001
 0.9999
 0.7172
 0.0579
 0.1534
 0.9994
 <.0001
 0.9997
 0.7491
 0.2472
 0.0383
 1.0000
 <.0001
 1.0000
 0.3873
 1.0000
 <.0001
 0.9984
 <.0001
 <.0001
 <.0001
 <.0001
 1.0000
 0.0008
 0.4047
 0.0435
 0.9999
 <.0001
 1.0000
 0.0007
 0.4383
 0.0396
 0.9997
 <.0001
 1.0000
 0.0071
 0.1635
 0.1806
 1.0000
 0.1141
 0.2025
 0.9999
 <.0001
 1.0000
 <.0001
 <.0001
 0.8824
 <.0001
 0.1662
 <.0001
 1.0000
 <.0001
 0.9694
 <.0001
 0.3904
 <.0001
 1.0000
 <.0001
 0.7638
 <.0001
 0.1282
 <.0001
 <.0001
 <.0001
 0.0001
 <.0001
 <.0001
 <.0001
 <.0001
 0.4346
 0.0039
 1.0000
 <.0001
 1.0000
 0.0002
 0.6547
 0.0158
 1.0000
 <.0001
 1.0000
 0.0024
 0.3094
 0.0867
 1.0000
 0.0516
 <.0001
 0.9977
 <.0001
 1.0000
 0.9239
 <.0001
 1.0000
 0.0023
 0.9502
 0.0004
 1.0000
 <.0001
 1.0000
 0.0147
 0.9989
 <.0001
 1.0000
 <.0001
 1.0000
 0.0022
 1.0000
 <.0001
 <.0001
 <.0001
 0.0123
 <.0001
 <.0001
 0.0356
 <.0001
 0.6012
 <.0001
 1.0000
 <.0001
 0.0889
 <.0001
 0.9079
 <.0001
 1.0000
 <.0001
 0.3090
 <.0001
 1.0000
 0.0039
 <.0001
 0.1071
 1.0000
 0.1150
 0.3237
 0.0216
 1.0000
 <.0001
 1.0000
 0.2815
 0.7214
 0.0037
 1.0000
 <.0001
 1.0000
 0.0828
 1.0000
 <.0001
 0.9027
 <.0001
 <.0001
 <.0001
 <.0001
 1.0000
 0.0108
 0.1105
 0.2370
 1.0000
 <.0001
 1.0000
 0.0671
 0.0264
 0.6009
 1.0000
 0.4695
 0.0355
 1.0000
 <.0001
 1.0000
 <.0001
 <.0001
 0.8824
 <.0001
 0.1662
 <.0001
 1.0000
 <.0001
 0.7353
 <.0001
 0.1136
 <.0001
 <.0001
 <.0001
 <.0001
 <.0001
 <.0001
 <.0001
 <.0001
 0.4346
 0.0039
 1.0000
 <.0001
 1.0000
 0.0030
 0.2904
 0.0995
 1.0000
 0.0615
 <.0001
 0.9985
 <.0001
 1.0000
 0.9123
 <.0001
 1.0000
 0.0023
 0.9993
 <.0001
 1.0000
 <.0001
 0.9999
 0.0019
 1.0000
 <.0001
 <.0001
 <.0001
 0.0107
 <.0001
 <.0001
 0.0356
 <.0001
 0.9217
 <.0001
 1.0000
 <.0001
 0.3362
 <.0001
 1.0000
 0.0047
 <.0001
 0.1201
 1.0000
 0.1150
 0.7567
 0.0031
 1.0000
 <.0001
 1.0000
 0.0749
 1.0000
 <.0001
 0.8879
 <.0001
 <.0001
 <.0001
 <.0001
 1.0000
 0.0771
 0.0235
 0.6336
 1.0000
 0.5070
 0.0312
 1.0000
 <.0001
 1.0000
 <.0001
 <.0001
 0.8824
 <.0001
 0.1662
 <.0001
 <.0001
 <.0001
 0.0005
 <.0001
 <.0001
 <.0001
 <.0001
 0.4346
 0.0039
 1.0000
 0.0125
 <.0001
 0.9549
 0.0001
 1.0000
 0.9952
 <.0001
 1.0000
 0.0023
 1.0000
 <.0001
 <.0001
 <.0001
 0.0642
 <.0001
 <.0001
 0.0356
 <.0001
 1.0000
 0.0006
 <.0001
 0.0285
 1.0000
 0.1150
 1.0000
 <.0001
 0.9933
 <.0001
 <.0001
 <.0001
 0.1962
 0.1354
 1.0000
 <.0001
 1.0000
 <.0001
 <.0001
 0.8824
 <.0001
 0.1662
 <.0001
 <.0001
 0.4346
 0.0039
 1.0000
 <.0001
 1.0000
 0.0023
 <.0001
 0.0356
 0.1150

Results are given on the log (not the response) scale.
P value adjustment: tukey method for comparing a family of 30 estimates

lsmeans(OrcoInactive_glmmTMB1, pairwise ~ Focal_Treatment * Rival_Treatment, adjust="tukey")

$lsmeans
 Focal_Treatment Rival_Treatment lsmean SE df asymp.LCL asymp.UCL
 Orco Single 2.00 0.0520 Inf 1.90 2.10
 WT Single 1.40 0.0582 Inf 1.29 1.51
 Orco WingClip 1.83 0.0537 Inf 1.72 1.93
 WT WingClip 1.16 0.0598 Inf 1.04 1.27
 Orco WingRemoved 1.75 0.0547 Inf 1.64 1.86
 WT WingRemoved 1.47 0.0576 Inf 1.36 1.59

Results are averaged over the levels of: Week
Results are given on the log (not the response) scale.
Confidence level used: 0.95

$contrasts
 contrast estimate SE df z.ratio p.value
 Orco Single - WT Single 0.6025 0.0778 Inf 7.744 <.0001
 Orco Single - Orco WingClip 0.1735 0.0746 Inf 2.327 0.1831
 Orco Single - WT WingClip 0.8465 0.0791 Inf 10.706 <.0001
 Orco Single - Orco WingRemoved 0.2525 0.0752 Inf 3.357 0.0102
 Orco Single - WT WingRemoved 0.5283 0.0774 Inf 6.825 <.0001
 WT Single - Orco WingClip -0.4291 0.0789 Inf -5.440 <.0001
 WT Single - WT WingClip 0.2439 0.0831 Inf 2.935 0.0391
 WT Single - Orco WingRemoved -0.3500 0.0795 Inf -4.405 0.0002
 WT Single - WT WingRemoved -0.0743 0.0815 Inf -0.911 0.9438
 Orco WingClip - WT WingClip 0.6730 0.0801 Inf 8.400 <.0001
 Orco WingClip - Orco WingRemoved 0.0790 0.0763 Inf 1.036 0.9060
 Orco WingClip - WT WingRemoved 0.3548 0.0785 Inf 4.521 0.0001
 WT WingClip - Orco WingRemoved -0.5940 0.0807 Inf -7.360 <.0001
 WT WingClip - WT WingRemoved -0.3182 0.0827 Inf -3.846 0.0017
 Orco WingRemoved - WT WingRemoved 0.2757 0.0791 Inf 3.487 0.0065

Results are averaged over the levels of: Week
Results are given on the log (not the response) scale.
P value adjustment: tukey method for comparing a family of 6 estimates

lsmeans(OrcoInactive_glmmTMB1, pairwise ~ Focal_Treatment, adjust="tukey")

NOTE: Results may be misleading due to involvement in interactions

$lsmeans
 Focal_Treatment lsmean SE df asymp.LCL asymp.UCL
 Orco 1.86 0.0310 Inf 1.80 1.92
 WT 1.34 0.0341 Inf 1.28 1.41

Results are averaged over the levels of: Rival_Treatment, Week
Results are given on the log (not the response) scale.
Confidence level used: 0.95

$contrasts
 contrast estimate SE df z.ratio p.value
 Orco - WT 0.517 0.0456 Inf 11.335 <.0001

Results are averaged over the levels of: Rival_Treatment, Week
Results are given on the log (not the response) scale.

lsmeans(OrcoInactive_glmmTMB1, pairwise ~ Rival_Treatment, adjust="tukey")

NOTE: Results may be misleading due to involvement in interactions

$lsmeans
 Rival_Treatment lsmean SE df asymp.LCL asymp.UCL
 Single 1.70 0.0391 Inf 1.62 1.78
 WingClip 1.49 0.0403 Inf 1.41 1.57
 WingRemoved 1.61 0.0399 Inf 1.53 1.69

Results are averaged over the levels of: Focal_Treatment, Week
Results are given on the log (not the response) scale.
Confidence level used: 0.95

$contrasts
 contrast estimate SE df z.ratio p.value
 Single - WingClip 0.2087 0.0558 Inf 3.739 0.0005
 Single - WingRemoved 0.0891 0.0555 Inf 1.607 0.2427
 WingClip - WingRemoved -0.1196 0.0563 Inf -2.125 0.0848

Results are averaged over the levels of: Focal_Treatment, Week
Results are given on the log (not the response) scale.
P value adjustment: tukey method for comparing a family of 3 estimates

lsmeans(OrcoInactive_glmmTMB1, pairwise ~ Week, adjust="tukey")

$lsmeans
 Week lsmean SE df asymp.LCL asymp.UCL
 1 1.62 0.0315 Inf 1.56 1.68
 2 1.67 0.0318 Inf 1.61 1.74
 3 1.68 0.0323 Inf 1.61 1.74
 4 1.64 0.0337 Inf 1.57 1.70
 5 1.40 0.0376 Inf 1.32 1.47

Results are averaged over the levels of: Focal_Treatment, Rival_Treatment
Results are given on the log (not the response) scale.
Confidence level used: 0.95

$contrasts
 contrast estimate SE df z.ratio p.value
 Week1 - Week2 -0.05154 0.0350 Inf -1.471 0.5814
 Week1 - Week3 -0.05480 0.0356 Inf -1.541 0.5357
 Week1 - Week4 -0.01442 0.0368 Inf -0.392 0.9950
 Week1 - Week5 0.22496 0.0405 Inf 5.560 <.0001
 Week2 - Week3 -0.00326 0.0354 Inf -0.092 1.0000
 Week2 - Week4 0.03713 0.0367 Inf 1.013 0.8496
 Week2 - Week5 0.27650 0.0403 Inf 6.859 <.0001
 Week3 - Week4 0.04038 0.0369 Inf 1.094 0.8097
 Week3 - Week5 0.27976 0.0406 Inf 6.897 <.0001
 Week4 - Week5 0.23937 0.0414 Inf 5.785 <.0001

Results are averaged over the levels of: Focal_Treatment, Rival_Treatment
Results are given on the log (not the response) scale.
P value adjustment: tukey method for comparing a family of 5 estimates

##Orco Inactive Boxplot
Orco_Inactive$PercentSuccess <- Orco_Inactive$Success / 10
Orco_Inactive_subset <- subset(Orco_Inactive, PercentSuccess > 0)
Orco_Inactive_subset$Rival_Treatment <- factor(Orco_Inactive_subset$Rival_Treatment, levels = c("Single", "WingClip", "WingRemoved"))
Orco_Inactive_subset$Focal_Treatment <- factor(Orco_Inactive_subset$Focal_Treatment, levels = c("WT", "Orco"))
Orco_Inactive_subset$Interaction <- interaction(Orco_Inactive_subset$Focal_Treatment, Orco_Inactive_subset$Rival_Treatment)
Orco_Inactive_subset$Interaction <- factor(Orco_Inactive_subset$Interaction, levels = c("WT.Single", "WT.WingClip", "WT.WingRemoved", "Orco.Single", "Orco.WingClip", "Orco.WingRemoved"))

Orco_Inactive_Summary <- summarySE(Orco_Inactive_subset,
 measurevar = "PercentSuccess",
 groupvars = c("Week", "Interaction", "Focal_Treatment", "Rival_Treatment"))

Orco_Inactive_Line <- ggplot(Orco_Inactive_Summary) +
 aes(
 x = Week,
 y = PercentSuccess,
 colour = Interaction,
 group = Interaction
 ) +
 scale_colour_manual(name = "Treatment",
 labels = c("Wildtype kept alone",
 "Wildtype kept with Wing Clipped Rival",
 "Wildtype kept with Wing Removed Rival",
 "Orco2 kept alone",
 "Orco2 kept with Wing Clipped Rival",
 "Orco2 kept with Wing Removed Rival"),
 values = c("black",
 "grey70",
 "grey42",
 "black",
 "grey70",
 "grey42")) +
 geom_line(aes(linetype = Interaction), size = 3) +
 geom_point() +
 geom_errorbar(aes(ymin=PercentSuccess-se, ymax=PercentSuccess+se), width=0.2, size = 1.5) +
 scale_linetype_manual(name = "Treatment",
 labels = c("Wildtype kept alone",
 "Wildtype kept with Wing Clipped Rival",
 "Wildtype kept with Wing Removed Rival",
 "Orco2 kept alone",
 "Orco2 kept with Wing Clipped Rival",
 "Orco2 kept with Wing Removed Rival"),
 values = c("solid", "solid", "solid", "dotdash", "dotdash", "dotdash")) +
 labs(
 x = "Week post Eclosion",
 y = "Proportion of Time Spent being Inactive"
 ) +
 ylim(0, 1.05) +
 theme_bw() +
 guides(colour = guide_legend(override.aes = list(linetype = c("solid", "solid", "solid", "dotdash", "dotdash", "dotdash"))))

Orco_Inactive_Line_Plot <- Orco_Inactive_Line + theme_bw() + theme(panel.grid.major = element_blank(), panel.grid.minor = element_blank()) +
 theme(axis.text=element_text(size=55, face="bold", colour="black"),
 axis.title=element_text(size=75, face="bold", colour="black"),
 legend.text=element_text(size=55, face="bold", colour="black"),
 legend.title=element_text(size=75, face="bold", colour="black"),
 strip.text=element_text(size=75, face="bold", colour="black")) + theme(axis.line = element_line(size = 5), panel.border = element_rect(size = 5)) + theme( axis.ticks.length = unit(0.5, "cm"), axis.ticks = element_line(size = 5))
Orco_Inactive_Line_Plot


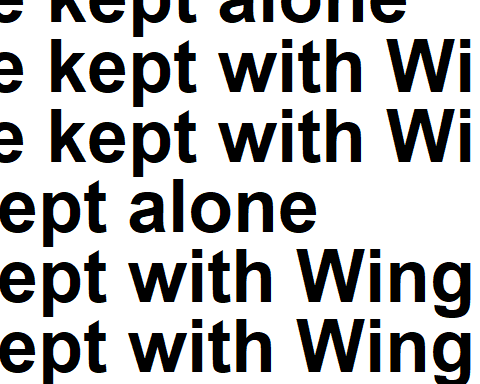


###Orco Aggression
##Orco Aggression Model
Orco_Aggression$Week <- as.factor(Orco_Aggression$Week)
Orco_Aggression_2 <- subset(Orco_Aggression, Rival_Treatment == "WingClip")
Orco_Aggression_3 <- subset(Orco_Aggression, Rival_Treatment == "WingRemoved")
Orco_Aggression_Final <- rbind(Orco_Aggression_2, Orco_Aggression_3)


Orco_Aggression$Week <- as.factor(Orco_Aggression$Week)
OrcoAggression_glmmTMB1 <- glmmTMB(Success ~ Focal_Treatment * Rival_Treatment + Week + (1|FlyID), family=poisson(link = "log"), data = Orco_Aggression_Final)
OrcoAggression_glmmTMB2 <- glmmTMB(Success ~ Focal_Treatment + Rival_Treatment + Week + (1|FlyID), family=poisson(link = "log"), data = Orco_Aggression_Final)
anova(OrcoAggression_glmmTMB1, OrcoAggression_glmmTMB2, test="Chi")

Data: Orco_Aggression_Final
Models:
OrcoAggression_glmmTMB2: Success ~ Focal_Treatment + Rival_Treatment + Week + (1 | FlyID), zi=~0, disp=~1
OrcoAggression_glmmTMB1: Success ~ Focal_Treatment * Rival_Treatment + Week + (1 | FlyID), zi=~0, disp=~1
 Df AIC BIC logLik deviance Chisq Chi Df
OrcoAggression_glmmTMB2 8 416.95 455.12 -200.48 400.95
OrcoAggression_glmmTMB1 9 416.75 459.69 -199.38 398.75 2.2029 1
 Pr(>Chisq)
OrcoAggression_glmmTMB2
OrcoAggression_glmmTMB1 0.1377

OrcoAggression_glmmTMB3 <- glmmTMB(Success ~ Rival_Treatment + Week + (1|FlyID), family=poisson(link = "log"), data = Orco_Aggression_Final)
anova(OrcoAggression_glmmTMB2, OrcoAggression_glmmTMB3, test="Chi")

Data: Orco_Aggression_Final
Models:
OrcoAggression_glmmTMB3: Success ~ Rival_Treatment + Week + (1 | FlyID), zi=~0, disp=~1
OrcoAggression_glmmTMB2: Success ~ Focal_Treatment + Rival_Treatment + Week + (1 | FlyID), zi=~0, disp=~1
 Df AIC BIC logLik deviance Chisq Chi Df
OrcoAggression_glmmTMB3 7 415.42 448.82 -200.71 401.42
OrcoAggression_glmmTMB2 8 416.95 455.12 -200.48 400.95 0.4725 1
 Pr(>Chisq)
OrcoAggression_glmmTMB3
OrcoAggression_glmmTMB2 0.4919

OrcoAggression_glmmTMB4 <- glmmTMB(Success ~ Focal_Treatment + Week + (1|FlyID), family=poisson(link = "log"), data = Orco_Aggression_Final)
anova(OrcoAggression_glmmTMB2, OrcoAggression_glmmTMB4, test="Chi")

Data: Orco_Aggression_Final
Models:
OrcoAggression_glmmTMB4: Success ~ Focal_Treatment + Week + (1 | FlyID), zi=~0, disp=~1
OrcoAggression_glmmTMB2: Success ~ Focal_Treatment + Rival_Treatment + Week + (1 | FlyID), zi=~0, disp=~1
 Df AIC BIC logLik deviance Chisq Chi Df
OrcoAggression_glmmTMB4 7 420.52 453.92 -203.26 406.52
OrcoAggression_glmmTMB2 8 416.95 455.12 -200.48 400.95 5.5698 1
 Pr(>Chisq)
OrcoAggression_glmmTMB4
OrcoAggression_glmmTMB2 0.01827 *
---
Signif. codes: 0 '***' 0.001 '**' 0.01 '*' 0.05 '.' 0.1 ' ' 1

OrcoAggression_glmmTMB5 <- glmmTMB(Success ~ Focal_Treatment + Rival_Treatment + (1|FlyID), family=poisson(link = "log"), data = Orco_Aggression_Final)
anova(OrcoAggression_glmmTMB2, OrcoAggression_glmmTMB5, test="Chi")

Data: Orco_Aggression_Final
Models:
OrcoAggression_glmmTMB5: Success ~ Focal_Treatment + Rival_Treatment + (1 | FlyID), zi=~0, disp=~1
OrcoAggression_glmmTMB2: Success ~ Focal_Treatment + Rival_Treatment + Week + (1 | FlyID), zi=~0, disp=~1
 Df AIC BIC logLik deviance Chisq Chi Df
OrcoAggression_glmmTMB5 4 451.19 470.28 -221.60 443.19
OrcoAggression_glmmTMB2 8 416.95 455.12 -200.48 400.95 42.24 4
 Pr(>Chisq)
OrcoAggression_glmmTMB5
OrcoAggression_glmmTMB2 1.488e-08 ***
---
Signif. codes: 0 '***' 0.001 '**' 0.01 '*' 0.05 '.' 0.1 ' ' 1

OrcoAggression_glmmTMB_null <- glmmTMB(Success ~ (1|FlyID), family=poisson(link = "log"), data = Orco_Aggression_Final)
anova(OrcoAggression_glmmTMB1, OrcoAggression_glmmTMB_null, test="Chi")

Data: Orco_Aggression_Final
Models:
OrcoAggression_glmmTMB_null: Success ~ (1 | FlyID), zi=~0, disp=~1
OrcoAggression_glmmTMB1: Success ~ Focal_Treatment * Rival_Treatment + Week + (1 | FlyID), zi=~0, disp=~1
 Df AIC BIC logLik deviance Chisq Chi Df
OrcoAggression_glmmTMB_null 2 453.13 462.67 -224.56 449.13
OrcoAggression_glmmTMB1 9 416.75 459.69 -199.38 398.75 50.381 7
 Pr(>Chisq)
OrcoAggression_glmmTMB_null
OrcoAggression_glmmTMB1 1.216e-08 ***
---
Signif. codes: 0 '***' 0.001 '**' 0.01 '*' 0.05 '.' 0.1 ' ' 1

summary(OrcoAggression_glmmTMB3)

Family: poisson ( log )
Formula: Success ~ Rival_Treatment + Week + (1 | FlyID)
Data: Orco_Aggression_Final

 AIC BIC logLik deviance df.resid
 415.4 448.8 -200.7 401.4 865

Random effects:

Conditional model:
 Groups Name Variance Std.Dev.
 FlyID (Intercept) 3.182 1.784
Number of obs: 872, groups: FlyID, 200

Conditional model:
 Estimate Std. Error z value Pr(>|z|)
(Intercept) -2.9251 0.4494 -6.509 7.57e-11 ***
Rival_TreatmentWingRemoved -1.0039 0.4453 -2.254 0.02418 *
Week2 -0.1170 0.2908 -0.402 0.68753
Week3 -0.6465 0.3516 -1.839 0.06595 .
Week4 -20.7994 7157.6102 -0.003 0.99768
Week5 -1.8891 0.6126 -3.084 0.00205 **
---
Signif. codes: 0 '***' 0.001 '**' 0.01 '*' 0.05 '.' 0.1 ' ' 1

lsmeans(OrcoAggression_glmmTMB2, pairwise ~ Focal_Treatment + Rival_Treatment + Week, adjust="tukey")

$lsmeans
 Focal_Treatment Rival_Treatment Week lsmean SE df asymp.LCL asymp.UCL
 Orco WingClip 1 -3.05 0.490 Inf -4.01 -2.08
 WT WingClip 1 -2.75 0.495 Inf -3.73 -1.78
 Orco WingRemoved 1 -4.06 0.597 Inf -5.23 -2.89
 WT WingRemoved 1 -3.77 0.584 Inf -4.91 -2.62
 Orco WingClip 2 -3.16 0.495 Inf -4.13 -2.19
 WT WingClip 2 -2.87 0.500 Inf -3.85 -1.89
 Orco WingRemoved 2 -4.18 0.600 Inf -5.35 -3.00
 WT WingRemoved 2 -3.88 0.587 Inf -5.04 -2.73
 Orco WingClip 3 -3.69 0.531 Inf -4.74 -2.65
 WT WingClip 3 -3.40 0.535 Inf -4.45 -2.35
 Orco WingRemoved 3 -4.71 0.629 Inf -5.94 -3.48
 WT WingRemoved 3 -4.42 0.616 Inf -5.62 -3.21
 Orco WingClip 4 -24.08 8048.982 Inf -15799.80 15751.63
 WT WingClip 4 -23.79 8048.982 Inf -15799.51 15751.92
 Orco WingRemoved 4 -25.10 8048.982 Inf -15800.81 15750.62
 WT WingRemoved 4 -24.80 8048.982 Inf -15800.52 15750.91
 Orco WingClip 5 -4.94 0.734 Inf -6.38 -3.50
 WT WingClip 5 -4.65 0.733 Inf -6.09 -3.21
 Orco WingRemoved 5 -5.95 0.807 Inf -7.54 -4.37
 WT WingRemoved 5 -5.66 0.794 Inf -7.22 -4.11

Results are given on the log (not the response) scale.
Confidence level used: 0.95

$contrasts
 contrast estimate SE df z.ratio
 Orco WingClip Week1 - WT WingClip Week1 -0.2920 0.423 Inf -0.690
 Orco WingClip Week1 - Orco WingRemoved Week1 1.0131 0.443 Inf 2.285
 Orco WingClip Week1 - WT WingRemoved Week1 0.7212 0.596 Inf 1.210
 Orco WingClip Week1 - Orco WingClip Week2 0.1169 0.291 Inf 0.402
 Orco WingClip Week1 - WT WingClip Week2 -0.1750 0.514 Inf -0.341
 Orco WingClip Week1 - Orco WingRemoved Week2 1.1301 0.528 Inf 2.138
 Orco WingClip Week1 - WT WingRemoved Week2 0.8381 0.662 Inf 1.265
 Orco WingClip Week1 - Orco WingClip Week3 0.6475 0.352 Inf 1.842
 Orco WingClip Week1 - WT WingClip Week3 0.3555 0.550 Inf 0.647
 Orco WingClip Week1 - Orco WingRemoved Week3 1.6607 0.563 Inf 2.950
 Orco WingClip Week1 - WT WingRemoved Week3 1.3687 0.689 Inf 1.985
 Orco WingClip Week1 - Orco WingClip Week4 21.0374 8048.982 Inf 0.003
 Orco WingClip Week1 - WT WingClip Week4 20.7455 8048.982 Inf 0.003
 Orco WingClip Week1 - Orco WingRemoved Week4 22.0506 8048.982 Inf 0.003
 Orco WingClip Week1 - WT WingRemoved Week4 21.7586 8048.982 Inf 0.003
 Orco WingClip Week1 - Orco WingClip Week5 1.8942 0.613 Inf 3.092
 Orco WingClip Week1 - WT WingClip Week5 1.6023 0.741 Inf 2.163
 Orco WingClip Week1 - Orco WingRemoved Week5 2.9074 0.753 Inf 3.859
 Orco WingClip Week1 - WT WingRemoved Week5 2.6154 0.849 Inf 3.081
 WT WingClip Week1 - Orco WingRemoved Week1 1.3051 0.629 Inf 2.075
 WT WingClip Week1 - WT WingRemoved Week1 1.0131 0.443 Inf 2.285
 WT WingClip Week1 - Orco WingClip Week2 0.4089 0.513 Inf 0.797
 WT WingClip Week1 - WT WingClip Week2 0.1169 0.291 Inf 0.402
 WT WingClip Week1 - Orco WingRemoved Week2 1.4221 0.691 Inf 2.057
 WT WingClip Week1 - WT WingRemoved Week2 1.1301 0.528 Inf 2.138
 WT WingClip Week1 - Orco WingClip Week3 0.9395 0.551 Inf 1.706
 WT WingClip Week1 - WT WingClip Week3 0.6475 0.352 Inf 1.842
 WT WingClip Week1 - Orco WingRemoved Week3 1.9526 0.719 Inf 2.717
 WT WingClip Week1 - WT WingRemoved Week3 1.6607 0.563 Inf 2.950
 WT WingClip Week1 - Orco WingClip Week4 21.3294 8048.982 Inf 0.003
 WT WingClip Week1 - WT WingClip Week4 21.0374 8048.982 Inf 0.003
 WT WingClip Week1 - Orco WingRemoved Week4 22.3426 8048.982 Inf 0.003
 WT WingClip Week1 - WT WingRemoved Week4 22.0506 8048.982 Inf 0.003
 WT WingClip Week1 - Orco WingClip Week5 2.1862 0.748 Inf 2.921
 WT WingClip Week1 - WT WingClip Week5 1.8942 0.613 Inf 3.092
 WT WingClip Week1 - Orco WingRemoved Week5 3.1994 0.879 Inf 3.640
 WT WingClip Week1 - WT WingRemoved Week5 2.9074 0.753 Inf 3.859
 Orco WingRemoved Week1 - WT WingRemoved Week1 -0.2920 0.423 Inf -0.690
 Orco WingRemoved Week1 - Orco WingClip Week2 -0.8962 0.532 Inf -1.685
 Orco WingRemoved Week1 - WT WingClip Week2 -1.1882 0.695 Inf -1.710
 Orco WingRemoved Week1 - Orco WingRemoved Week2 0.1169 0.291 Inf 0.402
 Orco WingRemoved Week1 - WT WingRemoved Week2 -0.1750 0.514 Inf -0.341
 Orco WingRemoved Week1 - Orco WingClip Week3 -0.3656 0.569 Inf -0.643
 Orco WingRemoved Week1 - WT WingClip Week3 -0.6576 0.723 Inf -0.910
 Orco WingRemoved Week1 - Orco WingRemoved Week3 0.6475 0.352 Inf 1.842
 Orco WingRemoved Week1 - WT WingRemoved Week3 0.3555 0.550 Inf 0.647
 Orco WingRemoved Week1 - Orco WingClip Week4 20.0243 8048.982 Inf 0.002
 Orco WingRemoved Week1 - WT WingClip Week4 19.7323 8048.982 Inf 0.002
 Orco WingRemoved Week1 - Orco WingRemoved Week4 21.0374 8048.982 Inf 0.003
 Orco WingRemoved Week1 - WT WingRemoved Week4 20.7455 8048.982 Inf 0.003
 Orco WingRemoved Week1 - Orco WingClip Week5 0.8811 0.759 Inf 1.161
 Orco WingRemoved Week1 - WT WingClip Week5 0.5891 0.877 Inf 0.672
 Orco WingRemoved Week1 - Orco WingRemoved Week5 1.8942 0.613 Inf 3.092
 Orco WingRemoved Week1 - WT WingRemoved Week5 1.6023 0.741 Inf 2.163
 WT WingRemoved Week1 - Orco WingClip Week2 -0.6042 0.664 Inf -0.910
 WT WingRemoved Week1 - WT WingClip Week2 -0.8962 0.532 Inf -1.685
 WT WingRemoved Week1 - Orco WingRemoved Week2 0.4089 0.513 Inf 0.797
 WT WingRemoved Week1 - WT WingRemoved Week2 0.1169 0.291 Inf 0.402
 WT WingRemoved Week1 - Orco WingClip Week3 -0.0737 0.695 Inf -0.106
 WT WingRemoved Week1 - WT WingClip Week3 -0.3656 0.569 Inf -0.643
 WT WingRemoved Week1 - Orco WingRemoved Week3 0.9395 0.551 Inf 1.706
 WT WingRemoved Week1 - WT WingRemoved Week3 0.6475 0.352 Inf 1.842
 WT WingRemoved Week1 - Orco WingClip Week4 20.3163 8048.982 Inf 0.003
 WT WingRemoved Week1 - WT WingClip Week4 20.0243 8048.982 Inf 0.002
 WT WingRemoved Week1 - Orco WingRemoved Week4 21.3294 8048.982 Inf 0.003
 WT WingRemoved Week1 - WT WingRemoved Week4 21.0374 8048.982 Inf 0.003
 WT WingRemoved Week1 - Orco WingClip Week5 1.1731 0.861 Inf 1.363
 WT WingRemoved Week1 - WT WingClip Week5 0.8811 0.759 Inf 1.161
 WT WingRemoved Week1 - Orco WingRemoved Week5 2.1862 0.748 Inf 2.921
 WT WingRemoved Week1 - WT WingRemoved Week5 1.8942 0.613 Inf 3.092
 Orco WingClip Week2 - WT WingClip Week2 -0.2920 0.423 Inf -0.690
 Orco WingClip Week2 - Orco WingRemoved Week2 1.0131 0.443 Inf 2.285
 Orco WingClip Week2 - WT WingRemoved Week2 0.7212 0.596 Inf 1.210
 Orco WingClip Week2 - Orco WingClip Week3 0.5306 0.361 Inf 1.472
 Orco WingClip Week2 - WT WingClip Week3 0.2386 0.555 Inf 0.430
 Orco WingClip Week2 - Orco WingRemoved Week3 1.5437 0.570 Inf 2.708
 Orco WingClip Week2 - WT WingRemoved Week3 1.2517 0.695 Inf 1.801
 Orco WingClip Week2 - Orco WingClip Week4 20.9205 8048.982 Inf 0.003
 Orco WingClip Week2 - WT WingClip Week4 20.6285 8048.982 Inf 0.003
 Orco WingClip Week2 - Orco WingRemoved Week4 21.9336 8048.982 Inf 0.003
 Orco WingClip Week2 - WT WingRemoved Week4 21.6417 8048.982 Inf 0.003
 Orco WingClip Week2 - Orco WingClip Week5 1.7773 0.618 Inf 2.877
 Orco WingClip Week2 - WT WingClip Week5 1.4853 0.745 Inf 1.995
 Orco WingClip Week2 - Orco WingRemoved Week5 2.7904 0.759 Inf 3.678
 Orco WingClip Week2 - WT WingRemoved Week5 2.4985 0.853 Inf 2.928
 WT WingClip Week2 - Orco WingRemoved Week2 1.3051 0.629 Inf 2.075
 WT WingClip Week2 - WT WingRemoved Week2 1.0131 0.443 Inf 2.285
 WT WingClip Week2 - Orco WingClip Week3 0.8225 0.557 Inf 1.477
 WT WingClip Week2 - WT WingClip Week3 0.5306 0.361 Inf 1.472
 WT WingClip Week2 - Orco WingRemoved Week3 1.8357 0.725 Inf 2.533
 WT WingClip Week2 - WT WingRemoved Week3 1.5437 0.570 Inf 2.708
 WT WingClip Week2 - Orco WingClip Week4 21.2125 8048.982 Inf 0.003
 WT WingClip Week2 - WT WingClip Week4 20.9205 8048.982 Inf 0.003
 WT WingClip Week2 - Orco WingRemoved Week4 22.2256 8048.982 Inf 0.003
 WT WingClip Week2 - WT WingRemoved Week4 21.9336 8048.982 Inf 0.003
 WT WingClip Week2 - Orco WingClip Week5 2.0693 0.753 Inf 2.748
 WT WingClip Week2 - WT WingClip Week5 1.7773 0.618 Inf 2.877
 WT WingClip Week2 - Orco WingRemoved Week5 3.0824 0.884 Inf 3.488
 WT WingClip Week2 - WT WingRemoved Week5 2.7904 0.759 Inf 3.678
 Orco WingRemoved Week2 - WT WingRemoved Week2 -0.2920 0.423 Inf -0.690
 Orco WingRemoved Week2 - Orco WingClip Week3 -0.4826 0.573 Inf -0.843
 Orco WingRemoved Week2 - WT WingClip Week3 -0.7746 0.725 Inf -1.068
 Orco WingRemoved Week2 - Orco WingRemoved Week3 0.5306 0.361 Inf 1.472
 Orco WingRemoved Week2 - WT WingRemoved Week3 0.2386 0.555 Inf 0.430
 Orco WingRemoved Week2 - Orco WingClip Week4 19.9074 8048.982 Inf 0.002
 Orco WingRemoved Week2 - WT WingClip Week4 19.6154 8048.982 Inf 0.002
 Orco WingRemoved Week2 - Orco WingRemoved Week4 20.9205 8048.982 Inf 0.003
 Orco WingRemoved Week2 - WT WingRemoved Week4 20.6285 8048.982 Inf 0.003
 Orco WingRemoved Week2 - Orco WingClip Week5 0.7641 0.762 Inf 1.003
 Orco WingRemoved Week2 - WT WingClip Week5 0.4722 0.879 Inf 0.537
 Orco WingRemoved Week2 - Orco WingRemoved Week5 1.7773 0.618 Inf 2.877
 Orco WingRemoved Week2 - WT WingRemoved Week5 1.4853 0.745 Inf 1.995
 WT WingRemoved Week2 - Orco WingClip Week3 -0.1906 0.699 Inf -0.273
 WT WingRemoved Week2 - WT WingClip Week3 -0.4826 0.573 Inf -0.843
 WT WingRemoved Week2 - Orco WingRemoved Week3 0.8225 0.557 Inf 1.477
 WT WingRemoved Week2 - WT WingRemoved Week3 0.5306 0.361 Inf 1.472
 WT WingRemoved Week2 - Orco WingClip Week4 20.1993 8048.982 Inf 0.003
 WT WingRemoved Week2 - WT WingClip Week4 19.9074 8048.982 Inf 0.002
 WT WingRemoved Week2 - Orco WingRemoved Week4 21.2125 8048.982 Inf 0.003
 WT WingRemoved Week2 - WT WingRemoved Week4 20.9205 8048.982 Inf 0.003
 WT WingRemoved Week2 - Orco WingClip Week5 1.0561 0.864 Inf 1.223
 WT WingRemoved Week2 - WT WingClip Week5 0.7641 0.762 Inf 1.003
 WT WingRemoved Week2 - Orco WingRemoved Week5 2.0693 0.753 Inf 2.748
 WT WingRemoved Week2 - WT WingRemoved Week5 1.7773 0.618 Inf 2.877
 Orco WingClip Week3 - WT WingClip Week3 -0.2920 0.423 Inf -0.690
 Orco WingClip Week3 - Orco WingRemoved Week3 1.0131 0.443 Inf 2.285
 Orco WingClip Week3 - WT WingRemoved Week3 0.7212 0.596 Inf 1.210
 Orco WingClip Week3 - Orco WingClip Week4 20.3899 8048.982 Inf 0.003
 Orco WingClip Week3 - WT WingClip Week4 20.0980 8048.982 Inf 0.002
 Orco WingClip Week3 - Orco WingRemoved Week4 21.4031 8048.982 Inf 0.003
 Orco WingClip Week3 - WT WingRemoved Week4 21.1111 8048.982 Inf 0.003
 Orco WingClip Week3 - Orco WingClip Week5 1.2467 0.647 Inf 1.927
 Orco WingClip Week3 - WT WingClip Week5 0.9547 0.770 Inf 1.241
 Orco WingClip Week3 - Orco WingRemoved Week5 2.2599 0.784 Inf 2.884
 Orco WingClip Week3 - WT WingRemoved Week5 1.9679 0.876 Inf 2.246
 WT WingClip Week3 - Orco WingRemoved Week3 1.3051 0.629 Inf 2.075
 WT WingClip Week3 - WT WingRemoved Week3 1.0131 0.443 Inf 2.285
 WT WingClip Week3 - Orco WingClip Week4 20.6819 8048.982 Inf 0.003
 WT WingClip Week3 - WT WingClip Week4 20.3899 8048.982 Inf 0.003
 WT WingClip Week3 - Orco WingRemoved Week4 21.6951 8048.982 Inf 0.003
 WT WingClip Week3 - WT WingRemoved Week4 21.4031 8048.982 Inf 0.003
 WT WingClip Week3 - Orco WingClip Week5 1.5387 0.776 Inf 1.982
 WT WingClip Week3 - WT WingClip Week5 1.2467 0.647 Inf 1.927
 WT WingClip Week3 - Orco WingRemoved Week5 2.5518 0.905 Inf 2.821
 WT WingClip Week3 - WT WingRemoved Week5 2.2599 0.784 Inf 2.884
 Orco WingRemoved Week3 - WT WingRemoved Week3 -0.2920 0.423 Inf -0.690
 Orco WingRemoved Week3 - Orco WingClip Week4 19.3768 8048.982 Inf 0.002
 Orco WingRemoved Week3 - WT WingClip Week4 19.0848 8048.982 Inf 0.002
 Orco WingRemoved Week3 - Orco WingRemoved Week4 20.3899 8048.982 Inf 0.003
 Orco WingRemoved Week3 - WT WingRemoved Week4 20.0980 8048.982 Inf 0.002
 Orco WingRemoved Week3 - Orco WingClip Week5 0.2336 0.785 Inf 0.298
 Orco WingRemoved Week3 - WT WingClip Week5 -0.0584 0.900 Inf -0.065
 Orco WingRemoved Week3 - Orco WingRemoved Week5 1.2467 0.647 Inf 1.927
 Orco WingRemoved Week3 - WT WingRemoved Week5 0.9547 0.770 Inf 1.241
 WT WingRemoved Week3 - Orco WingClip Week4 19.6688 8048.982 Inf 0.002
 WT WingRemoved Week3 - WT WingClip Week4 19.3768 8048.982 Inf 0.002
 WT WingRemoved Week3 - Orco WingRemoved Week4 20.6819 8048.982 Inf 0.003
 WT WingRemoved Week3 - WT WingRemoved Week4 20.3899 8048.982 Inf 0.003
 WT WingRemoved Week3 - Orco WingClip Week5 0.5255 0.883 Inf 0.595
 WT WingRemoved Week3 - WT WingClip Week5 0.2336 0.785 Inf 0.298
 WT WingRemoved Week3 - Orco WingRemoved Week5 1.5387 0.776 Inf 1.982
 WT WingRemoved Week3 - WT WingRemoved Week5 1.2467 0.647 Inf 1.927
 Orco WingClip Week4 - WT WingClip Week4 -0.2920 0.423 Inf -0.690
 Orco WingClip Week4 - Orco WingRemoved Week4 1.0131 0.443 Inf 2.285
 Orco WingClip Week4 - WT WingRemoved Week4 0.7212 0.596 Inf 1.210
 Orco WingClip Week4 - Orco WingClip Week5 -19.1432 8048.982 Inf -0.002
 Orco WingClip Week4 - WT WingClip Week5 -19.4352 8048.982 Inf -0.002
 Orco WingClip Week4 - Orco WingRemoved Week5 -18.1301 8048.982 Inf -0.002
 Orco WingClip Week4 - WT WingRemoved Week5 -18.4220 8048.982 Inf -0.002
 WT WingClip Week4 - Orco WingRemoved Week4 1.3051 0.629 Inf 2.075
 WT WingClip Week4 - WT WingRemoved Week4 1.0131 0.443 Inf 2.285
 WT WingClip Week4 - Orco WingClip Week5 -18.8512 8048.982 Inf -0.002
 WT WingClip Week4 - WT WingClip Week5 -19.1432 8048.982 Inf -0.002
 WT WingClip Week4 - Orco WingRemoved Week5 -17.8381 8048.982 Inf -0.002
 WT WingClip Week4 - WT WingRemoved Week5 -18.1301 8048.982 Inf -0.002
 Orco WingRemoved Week4 - WT WingRemoved Week4 -0.2920 0.423 Inf -0.690
 Orco WingRemoved Week4 - Orco WingClip Week5 -20.1564 8048.982 Inf -0.003
 Orco WingRemoved Week4 - WT WingClip Week5 -20.4483 8048.982 Inf -0.003
 Orco WingRemoved Week4 - Orco WingRemoved Week5 -19.1432 8048.982 Inf -0.002
 Orco WingRemoved Week4 - WT WingRemoved Week5 -19.4352 8048.982 Inf -0.002
 WT WingRemoved Week4 - Orco WingClip Week5 -19.8644 8048.982 Inf -0.002
 WT WingRemoved Week4 - WT WingClip Week5 -20.1564 8048.982 Inf -0.003
 WT WingRemoved Week4 - Orco WingRemoved Week5 -18.8512 8048.982 Inf -0.002
 WT WingRemoved Week4 - WT WingRemoved Week5 -19.1432 8048.982 Inf -0.002
 Orco WingClip Week5 - WT WingClip Week5 -0.2920 0.423 Inf -0.690
 Orco WingClip Week5 - Orco WingRemoved Week5 1.0131 0.443 Inf 2.285
 Orco WingClip Week5 - WT WingRemoved Week5 0.7212 0.596 Inf 1.210
 WT WingClip Week5 - Orco WingRemoved Week5 1.3051 0.629 Inf 2.075
 WT WingClip Week5 - WT WingRemoved Week5 1.0131 0.443 Inf 2.285
 Orco WingRemoved Week5 - WT WingRemoved Week5 -0.2920 0.423 Inf -0.690
 p.value
 1.0000
 0.7450
 0.9997
 1.0000
 1.0000
 0.8369
 0.9995
 0.9536
 1.0000
 0.2593
 0.9090
 1.0000
 1.0000
 1.0000
 1.0000
 0.1849
 0.8227
 0.0166
 0.1902
 0.8701
 0.7450
 1.0000
 1.0000
 0.8785
 0.8369
 0.9786
 0.9536
 0.4154
 0.2593
 1.0000
 1.0000
 1.0000
 1.0000
 0.2766
 0.1849
 0.0363
 0.0166
 1.0000
 0.9813
 0.9780
 1.0000
 1.0000
 1.0000
 1.0000
 0.9536
 1.0000
 1.0000
 1.0000
 1.0000
 1.0000
 0.9998
 1.0000
 0.1849
 0.8227
 1.0000
 0.9813
 1.0000
 1.0000
 1.0000
 1.0000
 0.9786
 0.9536
 1.0000
 1.0000
 1.0000
 1.0000
 0.9986
 0.9998
 0.2766
 0.1849
 1.0000
 0.7450
 0.9997
 0.9961
 1.0000
 0.4222
 0.9627
 1.0000
 1.0000
 1.0000
 1.0000
 0.3040
 0.9052
 0.0318
 0.2726
 0.8701
 0.7450
 0.9959
 0.9961
 0.5579
 0.4222
 1.0000
 1.0000
 1.0000
 1.0000
 0.3924
 0.3040
 0.0599
 0.0318
 1.0000
 1.0000
 1.0000
 0.9961
 1.0000
 1.0000
 1.0000
 1.0000
 1.0000
 1.0000
 1.0000
 0.3040
 0.9052
 1.0000
 1.0000
 0.9959
 0.9961
 1.0000
 1.0000
 1.0000
 1.0000
 0.9997
 1.0000
 0.3924
 0.3040
 1.0000
 0.7450
 0.9997
 1.0000
 1.0000
 1.0000
 1.0000
 0.9296
 0.9996
 0.2994
 0.7714
 0.8701
 0.7450
 1.0000
 1.0000
 1.0000
 1.0000
 0.9103
 0.9296
 0.3412
 0.2994
 1.0000
 1.0000
 1.0000
 1.0000
 1.0000
 1.0000
 1.0000
 0.9296
 0.9996
 1.0000
 1.0000
 1.0000
 1.0000
 1.0000
 1.0000
 0.9103
 0.9296
 1.0000
 0.7450
 0.9997
 1.0000
 1.0000
 1.0000
 1.0000
 0.8701
 0.7450
 1.0000
 1.0000
 1.0000
 1.0000
 1.0000
 1.0000
 1.0000
 1.0000
 1.0000
 1.0000
 1.0000
 1.0000
 1.0000
 1.0000
 0.7450
 0.9997
 0.8701
 0.7450
 1.0000

Results are given on the log (not the response) scale.
P value adjustment: tukey method for comparing a family of 20 estimates

lsmeans(OrcoAggression_glmmTMB2, pairwise ~ Focal_Treatment + Rival_Treatment, adjust="tukey")

$lsmeans
 Focal_Treatment Rival_Treatment lsmean SE df asymp.LCL asymp.UCL
 Orco WingClip -7.79 1610 Inf -3163 3147
 WT WingClip -7.49 1610 Inf -3163 3148
 Orco WingRemoved -8.80 1610 Inf -3164 3146
 WT WingRemoved -8.51 1610 Inf -3164 3147

Results are averaged over the levels of: Week
Results are given on the log (not the response) scale.
Confidence level used: 0.95

$contrasts
 contrast estimate SE df z.ratio p.value
 Orco WingClip - WT WingClip -0.292 0.423 Inf -0.690 0.9010
 Orco WingClip - Orco WingRemoved 1.013 0.443 Inf 2.285 0.1014
 Orco WingClip - WT WingRemoved 0.721 0.596 Inf 1.210 0.6207
 WT WingClip - Orco WingRemoved 1.305 0.629 Inf 2.075 0.1614
 WT WingClip - WT WingRemoved 1.013 0.443 Inf 2.285 0.1014
 Orco WingRemoved - WT WingRemoved -0.292 0.423 Inf -0.690 0.9010

Results are averaged over the levels of: Week
Results are given on the log (not the response) scale.
P value adjustment: tukey method for comparing a family of 4 estimates

lsmeans(OrcoAggression_glmmTMB2, pairwise ~ Focal_Treatment, adjust="tukey")

$lsmeans
 Focal_Treatment lsmean SE df asymp.LCL asymp.UCL
 Orco -8.29 1610 Inf -3163 3147
 WT -8.00 1610 Inf -3163 3147

Results are averaged over the levels of: Rival_Treatment, Week
Results are given on the log (not the response) scale.
Confidence level used: 0.95

$contrasts
 contrast estimate SE df z.ratio p.value
 Orco - WT -0.292 0.423 Inf -0.690 0.4902

Results are averaged over the levels of: Rival_Treatment, Week
Results are given on the log (not the response) scale.

lsmeans(OrcoAggression_glmmTMB2, pairwise ~ Rival_Treatment, adjust="tukey")

$lsmeans
 Rival_Treatment lsmean SE df asymp.LCL asymp.UCL
 WingClip -7.64 1610 Inf -3163 3148
 WingRemoved -8.65 1610 Inf -3164 3146

Results are averaged over the levels of: Focal_Treatment, Week
Results are given on the log (not the response) scale.
Confidence level used: 0.95

$contrasts
 contrast estimate SE df z.ratio p.value
 WingClip - WingRemoved 1.01 0.443 Inf 2.285 0.0223

Results are averaged over the levels of: Focal_Treatment, Week
Results are given on the log (not the response) scale.

lsmeans(OrcoAggression_glmmTMB2, pairwise ~ Week, adjust="tukey")

$lsmeans
 Week lsmean SE df asymp.LCL asymp.UCL
 1 -3.41 0.449 Inf -4.29 -2.53
 2 -3.52 0.454 Inf -4.41 -2.63
 3 -4.05 0.492 Inf -5.02 -3.09
 4 -24.44 8048.982 Inf -15800.16 15751.27
 5 -5.30 0.704 Inf -6.68 -3.92

Results are averaged over the levels of: Focal_Treatment, Rival_Treatment
Results are given on the log (not the response) scale.
Confidence level used: 0.95

$contrasts
 contrast estimate SE df z.ratio p.value
 Week1 - Week2 0.117 0.291 Inf 0.402 0.9945
 Week1 - Week3 0.648 0.352 Inf 1.842 0.3493
 Week1 - Week4 21.037 8048.982 Inf 0.003 1.0000
 Week1 - Week5 1.894 0.613 Inf 3.092 0.0170
 Week2 - Week3 0.531 0.361 Inf 1.472 0.5810
 Week2 - Week4 20.921 8048.982 Inf 0.003 1.0000
 Week2 - Week5 1.777 0.618 Inf 2.877 0.0327
 Week3 - Week4 20.390 8048.982 Inf 0.003 1.0000
 Week3 - Week5 1.247 0.647 Inf 1.927 0.3026
 Week4 - Week5 -19.143 8048.982 Inf -0.002 1.0000

Results are averaged over the levels of: Focal_Treatment, Rival_Treatment
Results are given on the log (not the response) scale.
P value adjustment: tukey method for comparing a family of 5 estimates

#Orco Aggression Line
Orco_Aggression_Final$PercentSuccess <- Orco_Aggression_Final$Success / 10
Orco_Aggression_subset <- subset(Orco_Aggression_Final, PercentSuccess > 0)
Orco_Aggression_subset$Rival_Treatment <- factor(Orco_Aggression_subset$Rival_Treatment, levels = c("WingClip", "WingRemoved"))
Orco_Aggression_subset$Focal_Treatment <- factor(Orco_Aggression_subset$Focal_Treatment, levels = c("WT", "Orco"))
Orco_Aggression_subset$Interaction <- interaction(Orco_Aggression_subset$Focal_Treatment, Orco_Aggression_subset$Rival_Treatment)
Orco_Aggression_subset$Interaction <- factor(Orco_Aggression_subset$Interaction, levels = c("WT.WingClip", "WT.WingRemoved", "Orco.WingClip", "Orco.WingRemoved"))

Orco_Aggression_Summary <- summarySE(Orco_Aggression_subset,
 measurevar = "PercentSuccess",
 groupvars = c("Week", "Interaction", "Focal_Treatment", "Rival_Treatment"))

Warning in qt(conf.interval/2 + 0.5, datac$N - 1): NaNs produced

# Adding in Zeros
Orco_Aggression_all_combinations <- expand.grid(
 Week = 1:5,
 Focal_Treatment = c("WT", "Orco"),
 Rival_Treatment = c("WingClip", "WingRemoved")
)

Orco_Aggression_all_combinations$Interaction <- str_c(Orco_Aggression_all_combinations$Focal_Treatment, ".", Orco_Aggression_all_combinations$Rival_Treatment)

Orco_Aggression_Summary <- merge(Orco_Aggression_all_combinations, Orco_Aggression_Summary,
 by = c("Week", "Interaction", "Focal_Treatment", "Rival_Treatment"),
 all.x = TRUE)

# Fill NAs
Orco_Aggression_Summary$N[is.na(Orco_Aggression_Summary$N)] <- 0
Orco_Aggression_Summary$PercentSuccess[is.na(Orco_Aggression_Summary$PercentSuccess)] <- 0
Orco_Aggression_Summary$sd[is.na(Orco_Aggression_Summary$sd)] <- NA
Orco_Aggression_Summary$se[is.na(Orco_Aggression_Summary$se)] <- NA
Orco_Aggression_Summary$ci[is.na(Orco_Aggression_Summary$ci)] <- NA


Orco_Aggression_Line <- ggplot(Orco_Aggression_Summary) +
 aes(
 x = Week,
 y = PercentSuccess,
 colour = Interaction,
 group = Interaction
 ) +
 scale_colour_manual(name = "Treatment",
 labels = c("Wildtype kept with Wing Clipped Rival",
 "Wildtype kept with Wing Removed Rival",
 "Orco2 kept with Wing Clipped Rival",
 "Orco2 kept with Wing Removed Rival"),
 values = c("grey70",
 "grey42",
 "grey70",
 "grey42")) +
 geom_line(aes(linetype = Interaction), size = 3) +
 geom_point() +
 geom_errorbar(aes(ymin=PercentSuccess-se, ymax=PercentSuccess+se), width=0.2, size = 1.5) +
 scale_linetype_manual(name = "Treatment",
 labels = c("Wildtype kept with Wing Clipped Rival",
 "Wildtype kept with Wing Removed Rival",
 "Orco2 kept with Wing Clipped Rival",
 "Orco2 kept with Wing Removed Rival"),
 values = c("solid", "solid", "dotdash", "dotdash")) +
 labs(
 x = "Week post Eclosion",
 y = "Proportion of Time Spent being Aggressive"
 ) +
 ylim(0, 0.55) +
 theme_bw() +
 guides(colour = guide_legend(override.aes = list(linetype = c("solid", "solid", "dotdash", "dotdash"))))

Orco_Aggression_Line_Plot <- Orco_Aggression_Line + theme_bw() + theme(panel.grid.major = element_blank(), panel.grid.minor = element_blank()) +
 theme(axis.text=element_text(size=55, face="bold", colour="black"),
 axis.title=element_text(size=75, face="bold", colour="black"),
 legend.text=element_text(size=55, face="bold", colour="black"),
 legend.title=element_text(size=75, face="bold", colour="black"),
 strip.text=element_text(size=75, face="bold", colour="black")) + theme(axis.line = element_line(size = 5), panel.border = element_rect(size = 5)) + theme( axis.ticks.length = unit(0.5, "cm"), axis.ticks = element_line(size = 5))
Orco_Aggression_Line_Plot


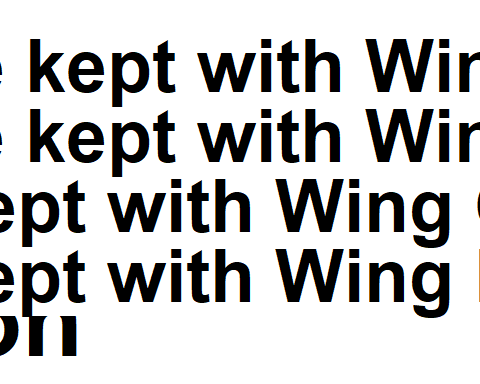


###Orco WBL
##Orco WBL Model
Orco_WBL$Week <- as.factor(Orco_WBL$Week)
OrcoWBL_glmmTMB1 <- glmmTMB(Success ~ Focal_Treatment * Rival_Treatment + Week + (1|FlyID), family=poisson(link = "log"), data = Orco_WBL)
OrcoWBL_glmmTMB2 <- glmmTMB(Success ~ Focal_Treatment + Rival_Treatment + Week + (1|FlyID), family=poisson(link = "log"), data = Orco_WBL)
anova(OrcoWBL_glmmTMB1, OrcoWBL_glmmTMB2, test="Chi")

Data: Orco_WBL
Models:
OrcoWBL_glmmTMB2: Success ~ Focal_Treatment + Rival_Treatment + Week + (1 | FlyID), zi=~0, disp=~1
OrcoWBL_glmmTMB1: Success ~ Focal_Treatment * Rival_Treatment + Week + (1 | FlyID), zi=~0, disp=~1
 Df AIC BIC logLik deviance Chisq Chi Df Pr(>Chisq)
OrcoWBL_glmmTMB2 8 2712.6 2750.8 -1348.3 2696.6
OrcoWBL_glmmTMB1 9 2712.6 2755.6 -1347.3 2694.6 2.0279 1 0.1544

OrcoWBL_glmmTMB3 <- glmmTMB(Success ~ Rival_Treatment + Week + (1|FlyID), family=poisson(link = "log"), data = Orco_WBL)
anova(OrcoWBL_glmmTMB2, OrcoWBL_glmmTMB3, test="Chi")

Data: Orco_WBL
Models:
OrcoWBL_glmmTMB3: Success ~ Rival_Treatment + Week + (1 | FlyID), zi=~0, disp=~1
OrcoWBL_glmmTMB2: Success ~ Focal_Treatment + Rival_Treatment + Week + (1 | FlyID), zi=~0, disp=~1
 Df AIC BIC logLik deviance Chisq Chi Df Pr(>Chisq)
OrcoWBL_glmmTMB3 7 2711.8 2745.2 -1348.9 2697.8
OrcoWBL_glmmTMB2 8 2712.6 2750.8 -1348.3 2696.6 1.1507 1 0.2834

OrcoWBL_glmmTMB4 <- glmmTMB(Success ~ Focal_Treatment + Week + (1|FlyID), family=poisson(link = "log"), data = Orco_WBL)
anova(OrcoWBL_glmmTMB2, OrcoWBL_glmmTMB4, test="Chi")

Data: Orco_WBL
Models:
OrcoWBL_glmmTMB4: Success ~ Focal_Treatment + Week + (1 | FlyID), zi=~0, disp=~1
OrcoWBL_glmmTMB2: Success ~ Focal_Treatment + Rival_Treatment + Week + (1 | FlyID), zi=~0, disp=~1
 Df AIC BIC logLik deviance Chisq Chi Df Pr(>Chisq)
OrcoWBL_glmmTMB4 7 2710.7 2744.1 -1348.3 2696.7
OrcoWBL_glmmTMB2 8 2712.6 2750.8 -1348.3 2696.6 0.0229 1 0.8796

OrcoWBL_glmmTMB5 <- glmmTMB(Success ~ Week + (1|FlyID), family=poisson(link = "log"), data = Orco_WBL)
anova(OrcoWBL_glmmTMB2, OrcoWBL_glmmTMB5, test="Chi")

Data: Orco_WBL
Models:
OrcoWBL_glmmTMB5: Success ~ Week + (1 | FlyID), zi=~0, disp=~1
OrcoWBL_glmmTMB2: Success ~ Focal_Treatment + Rival_Treatment + Week + (1 | FlyID), zi=~0, disp=~1
 Df AIC BIC logLik deviance Chisq Chi Df Pr(>Chisq)
OrcoWBL_glmmTMB5 6 2709.8 2738.4 -1348.9 2697.8
OrcoWBL_glmmTMB2 8 2712.6 2750.8 -1348.3 2696.6 1.1676 2 0.5578

OrcoWBL_glmmTMB_nul <- glmmTMB(Success ~ (1|FlyID), family=poisson(link = "log"), data = Orco_WBL)
anova(OrcoWBL_glmmTMB2, OrcoWBL_glmmTMB_nul, test="Chi")

Data: Orco_WBL
Models:
OrcoWBL_glmmTMB_nul: Success ~ (1 | FlyID), zi=~0, disp=~1
OrcoWBL_glmmTMB2: Success ~ Focal_Treatment + Rival_Treatment + Week + (1 | FlyID), zi=~0, disp=~1
 Df AIC BIC logLik deviance Chisq Chi Df Pr(>Chisq)
OrcoWBL_glmmTMB_nul 2 2725.4 2734.9 -1360.7 2721.4
OrcoWBL_glmmTMB2 8 2712.6 2750.8 -1348.3 2696.6 24.768 6 0.0003769

OrcoWBL_glmmTMB_nul
OrcoWBL_glmmTMB2 ***
---
Signif. codes: 0 '***' 0.001 '**' 0.01 '*' 0.05 '.' 0.1 ' ' 1

summary(OrcoWBL_glmmTMB1)

Family: poisson ( log )
Formula:
Success ~ Focal_Treatment * Rival_Treatment + Week + (1 | FlyID)
Data: Orco_WBL

 AIC BIC logLik deviance df.resid
 2712.6 2755.5 -1347.3 2694.6 863

Random effects:

Conditional model:
 Groups Name Variance Std.Dev.
 FlyID (Intercept) 0.5538 0.7442
Number of obs: 872, groups: FlyID, 200

Conditional model:
 Estimate Std. Error z value
(Intercept) -0.34037 0.14807 -2.299
Focal_TreatmentWT 0.04215 0.18508 0.228
Rival_TreatmentWingRemoved 0.16488 0.18464 0.893
Week2 -0.08496 0.10852 -0.783
Week3 0.17963 0.10443 1.720
Week4 0.04311 0.11046 0.390
Week5 0.38263 0.10361 3.693
Focal_TreatmentWT:Rival_TreatmentWingRemoved -0.37780 0.26439 -1.429
 Pr(>|z|)
(Intercept) 0.021523 *
Focal_TreatmentWT 0.819860
Rival_TreatmentWingRemoved 0.371871
Week2 0.433693
Week3 0.085392 .
Week4 0.696354
Week5 0.000222 ***
Focal_TreatmentWT:Rival_TreatmentWingRemoved 0.153018
---
Signif. codes: 0 '***' 0.001 '**' 0.01 '*' 0.05 '.' 0.1 ' ' 1

lsmeans(OrcoWBL_glmmTMB2, pairwise ~ Focal_Treatment + Rival_Treatment + Week, adjust="tukey")

$lsmeans
 Focal_Treatment Rival_Treatment Week lsmean SE df asymp.LCL asymp.UCL
 Orco WingClip 1 -0.25026 0.133 Inf -0.512 0.01100
 WT WingClip 1 -0.39332 0.134 Inf -0.655 -0.13156
 Orco WingRemoved 1 -0.27046 0.134 Inf -0.534 -0.00696
 WT WingRemoved 1 -0.41351 0.138 Inf -0.684 -0.14321
 Orco WingClip 2 -0.33503 0.136 Inf -0.602 -0.06843
 WT WingClip 2 -0.47809 0.137 Inf -0.746 -0.21008
 Orco WingRemoved 2 -0.35523 0.138 Inf -0.625 -0.08540
 WT WingRemoved 2 -0.49828 0.142 Inf -0.776 -0.22092
 Orco WingClip 3 -0.07022 0.132 Inf -0.330 0.18905
 WT WingClip 3 -0.21328 0.133 Inf -0.473 0.04663
 Orco WingRemoved 3 -0.09042 0.134 Inf -0.353 0.17169
 WT WingRemoved 3 -0.23347 0.137 Inf -0.503 0.03560
 Orco WingClip 4 -0.20624 0.138 Inf -0.476 0.06338
 WT WingClip 4 -0.34930 0.137 Inf -0.618 -0.08052
 Orco WingRemoved 4 -0.22644 0.139 Inf -0.499 0.04573
 WT WingRemoved 4 -0.36949 0.142 Inf -0.647 -0.09202
 Orco WingClip 5 0.13395 0.133 Inf -0.126 0.39434
 WT WingClip 5 -0.00911 0.132 Inf -0.267 0.24928
 Orco WingRemoved 5 0.11375 0.134 Inf -0.149 0.37646
 WT WingRemoved 5 -0.02930 0.136 Inf -0.296 0.23781

Results are given on the log (not the response) scale.
Confidence level used: 0.95

$contrasts
 contrast estimate SE df z.ratio
 Orco WingClip Week1 - WT WingClip Week1 0.14306 0.133 Inf 1.075
 Orco WingClip Week1 - Orco WingRemoved Week1 0.02019 0.133 Inf 0.151
 Orco WingClip Week1 - WT WingRemoved Week1 0.16325 0.191 Inf 0.856
 Orco WingClip Week1 - Orco WingClip Week2 0.08477 0.109 Inf 0.781
 Orco WingClip Week1 - WT WingClip Week2 0.22783 0.172 Inf 1.324
 Orco WingClip Week1 - Orco WingRemoved Week2 0.10497 0.172 Inf 0.609
 Orco WingClip Week1 - WT WingRemoved Week2 0.24802 0.220 Inf 1.127
 Orco WingClip Week1 - Orco WingClip Week3 -0.18004 0.104 Inf -1.724
 Orco WingClip Week1 - WT WingClip Week3 -0.03698 0.169 Inf -0.219
 Orco WingClip Week1 - Orco WingRemoved Week3 -0.15984 0.170 Inf -0.942
 Orco WingClip Week1 - WT WingRemoved Week3 -0.01679 0.218 Inf -0.077
 Orco WingClip Week1 - Orco WingClip Week4 -0.04402 0.110 Inf -0.398
 Orco WingClip Week1 - WT WingClip Week4 0.09904 0.172 Inf 0.574
 Orco WingClip Week1 - Orco WingRemoved Week4 -0.02383 0.173 Inf -0.137
 Orco WingClip Week1 - WT WingRemoved Week4 0.11923 0.220 Inf 0.542
 Orco WingClip Week1 - Orco WingClip Week5 -0.38421 0.104 Inf -3.707
 Orco WingClip Week1 - WT WingClip Week5 -0.24115 0.168 Inf -1.438
 Orco WingClip Week1 - Orco WingRemoved Week5 -0.36401 0.169 Inf -2.155
 Orco WingClip Week1 - WT WingRemoved Week5 -0.22096 0.216 Inf -1.021
 WT WingClip Week1 - Orco WingRemoved Week1 -0.12286 0.186 Inf -0.660
 WT WingClip Week1 - WT WingRemoved Week1 0.02019 0.133 Inf 0.151
 WT WingClip Week1 - Orco WingClip Week2 -0.05828 0.171 Inf -0.340
 WT WingClip Week1 - WT WingClip Week2 0.08477 0.109 Inf 0.781
 WT WingClip Week1 - Orco WingRemoved Week2 -0.03809 0.215 Inf -0.177
 WT WingClip Week1 - WT WingRemoved Week2 0.10497 0.172 Inf 0.609
 WT WingClip Week1 - Orco WingClip Week3 -0.32309 0.169 Inf -1.911
 WT WingClip Week1 - WT WingClip Week3 -0.18004 0.104 Inf -1.724
 WT WingClip Week1 - Orco WingRemoved Week3 -0.30290 0.214 Inf -1.419
 WT WingClip Week1 - WT WingRemoved Week3 -0.15984 0.170 Inf -0.942
 WT WingClip Week1 - Orco WingClip Week4 -0.18708 0.173 Inf -1.078
 WT WingClip Week1 - WT WingClip Week4 -0.04402 0.110 Inf -0.398
 WT WingClip Week1 - Orco WingRemoved Week4 -0.16688 0.217 Inf -0.769
 WT WingClip Week1 - WT WingRemoved Week4 -0.02383 0.173 Inf -0.137
 WT WingClip Week1 - Orco WingClip Week5 -0.52726 0.170 Inf -3.108
 WT WingClip Week1 - WT WingClip Week5 -0.38421 0.104 Inf -3.707
 WT WingClip Week1 - Orco WingRemoved Week5 -0.50707 0.214 Inf -2.372
 WT WingClip Week1 - WT WingRemoved Week5 -0.36401 0.169 Inf -2.155
 Orco WingRemoved Week1 - WT WingRemoved Week1 0.14306 0.133 Inf 1.075
 Orco WingRemoved Week1 - Orco WingClip Week2 0.06458 0.172 Inf 0.376
 Orco WingRemoved Week1 - WT WingClip Week2 0.20763 0.215 Inf 0.964
 Orco WingRemoved Week1 - Orco WingRemoved Week2 0.08477 0.109 Inf 0.781
 Orco WingRemoved Week1 - WT WingRemoved Week2 0.22783 0.172 Inf 1.324
 Orco WingRemoved Week1 - Orco WingClip Week3 -0.20023 0.169 Inf -1.183
 Orco WingRemoved Week1 - WT WingClip Week3 -0.05718 0.213 Inf -0.268
 Orco WingRemoved Week1 - Orco WingRemoved Week3 -0.18004 0.104 Inf -1.724
 Orco WingRemoved Week1 - WT WingRemoved Week3 -0.03698 0.169 Inf -0.219
 Orco WingRemoved Week1 - Orco WingClip Week4 -0.06421 0.173 Inf -0.371
 Orco WingRemoved Week1 - WT WingClip Week4 0.07884 0.216 Inf 0.365
 Orco WingRemoved Week1 - Orco WingRemoved Week4 -0.04402 0.110 Inf -0.398
 Orco WingRemoved Week1 - WT WingRemoved Week4 0.09904 0.172 Inf 0.574
 Orco WingRemoved Week1 - Orco WingClip Week5 -0.40440 0.169 Inf -2.394
 Orco WingRemoved Week1 - WT WingClip Week5 -0.26135 0.212 Inf -1.232
 Orco WingRemoved Week1 - Orco WingRemoved Week5 -0.38421 0.104 Inf -3.707
 Orco WingRemoved Week1 - WT WingRemoved Week5 -0.24115 0.168 Inf -1.438
 WT WingRemoved Week1 - Orco WingClip Week2 -0.07848 0.219 Inf -0.359
 WT WingRemoved Week1 - WT WingClip Week2 0.06458 0.172 Inf 0.376
 WT WingRemoved Week1 - Orco WingRemoved Week2 -0.05828 0.171 Inf -0.340
 WT WingRemoved Week1 - WT WingRemoved Week2 0.08477 0.109 Inf 0.781
 WT WingRemoved Week1 - Orco WingClip Week3 -0.34329 0.217 Inf -1.580
 WT WingRemoved Week1 - WT WingClip Week3 -0.20023 0.169 Inf -1.183
 WT WingRemoved Week1 - Orco WingRemoved Week3 -0.32309 0.169 Inf -1.911
 WT WingRemoved Week1 - WT WingRemoved Week3 -0.18004 0.104 Inf -1.724
 WT WingRemoved Week1 - Orco WingClip Week4 -0.20727 0.221 Inf -0.939
 WT WingRemoved Week1 - WT WingClip Week4 -0.06421 0.173 Inf -0.371
 WT WingRemoved Week1 - Orco WingRemoved Week4 -0.18708 0.173 Inf -1.078
 WT WingRemoved Week1 - WT WingRemoved Week4 -0.04402 0.110 Inf -0.398
 WT WingRemoved Week1 - Orco WingClip Week5 -0.54746 0.218 Inf -2.513
 WT WingRemoved Week1 - WT WingClip Week5 -0.40440 0.169 Inf -2.394
 WT WingRemoved Week1 - Orco WingRemoved Week5 -0.52726 0.170 Inf -3.108
 WT WingRemoved Week1 - WT WingRemoved Week5 -0.38421 0.104 Inf -3.707
 Orco WingClip Week2 - WT WingClip Week2 0.14306 0.133 Inf 1.075
 Orco WingClip Week2 - Orco WingRemoved Week2 0.02019 0.133 Inf 0.151
 Orco WingClip Week2 - WT WingRemoved Week2 0.16325 0.191 Inf 0.856
 Orco WingClip Week2 - Orco WingClip Week3 -0.26481 0.108 Inf -2.456
 Orco WingClip Week2 - WT WingClip Week3 -0.12175 0.171 Inf -0.712
 Orco WingClip Week2 - Orco WingRemoved Week3 -0.24462 0.171 Inf -1.428
 Orco WingClip Week2 - WT WingRemoved Week3 -0.10156 0.219 Inf -0.464
 Orco WingClip Week2 - Orco WingClip Week4 -0.12879 0.114 Inf -1.133
 Orco WingClip Week2 - WT WingClip Week4 0.01426 0.174 Inf 0.082
 Orco WingClip Week2 - Orco WingRemoved Week4 -0.10860 0.175 Inf -0.621
 Orco WingClip Week2 - WT WingRemoved Week4 0.03446 0.221 Inf 0.156
 Orco WingClip Week2 - Orco WingClip Week5 -0.46898 0.107 Inf -4.380
 Orco WingClip Week2 - WT WingClip Week5 -0.32593 0.169 Inf -1.924
 Orco WingClip Week2 - Orco WingRemoved Week5 -0.44879 0.171 Inf -2.630
 Orco WingClip Week2 - WT WingRemoved Week5 -0.30573 0.217 Inf -1.407
 WT WingClip Week2 - Orco WingRemoved Week2 -0.12286 0.186 Inf -0.660
 WT WingClip Week2 - WT WingRemoved Week2 0.02019 0.133 Inf 0.151
 WT WingClip Week2 - Orco WingClip Week3 -0.40787 0.172 Inf -2.377
 WT WingClip Week2 - WT WingClip Week3 -0.26481 0.108 Inf -2.456
 WT WingClip Week2 - Orco WingRemoved Week3 -0.38767 0.215 Inf -1.802
 WT WingClip Week2 - WT WingRemoved Week3 -0.24462 0.171 Inf -1.428
 WT WingClip Week2 - Orco WingClip Week4 -0.27185 0.176 Inf -1.545
 WT WingClip Week2 - WT WingClip Week4 -0.12879 0.114 Inf -1.133
 WT WingClip Week2 - Orco WingRemoved Week4 -0.25165 0.219 Inf -1.151
 WT WingClip Week2 - WT WingRemoved Week4 -0.10860 0.175 Inf -0.621
 WT WingClip Week2 - Orco WingClip Week5 -0.61204 0.172 Inf -3.555
 WT WingClip Week2 - WT WingClip Week5 -0.46898 0.107 Inf -4.380
 WT WingClip Week2 - Orco WingRemoved Week5 -0.59184 0.215 Inf -2.747
 WT WingClip Week2 - WT WingRemoved Week5 -0.44879 0.171 Inf -2.630
 Orco WingRemoved Week2 - WT WingRemoved Week2 0.14306 0.133 Inf 1.075
 Orco WingRemoved Week2 - Orco WingClip Week3 -0.28500 0.172 Inf -1.660
 Orco WingRemoved Week2 - WT WingClip Week3 -0.14195 0.215 Inf -0.660
 Orco WingRemoved Week2 - Orco WingRemoved Week3 -0.26481 0.108 Inf -2.456
 Orco WingRemoved Week2 - WT WingRemoved Week3 -0.12175 0.171 Inf -0.712
 Orco WingRemoved Week2 - Orco WingClip Week4 -0.14899 0.176 Inf -0.849
 Orco WingRemoved Week2 - WT WingClip Week4 -0.00593 0.218 Inf -0.027
 Orco WingRemoved Week2 - Orco WingRemoved Week4 -0.12879 0.114 Inf -1.133
 Orco WingRemoved Week2 - WT WingRemoved Week4 0.01426 0.174 Inf 0.082
 Orco WingRemoved Week2 - Orco WingClip Week5 -0.48918 0.171 Inf -2.853
 Orco WingRemoved Week2 - WT WingClip Week5 -0.34612 0.214 Inf -1.618
 Orco WingRemoved Week2 - Orco WingRemoved Week5 -0.46898 0.107 Inf -4.380
 Orco WingRemoved Week2 - WT WingRemoved Week5 -0.32593 0.169 Inf -1.924
 WT WingRemoved Week2 - Orco WingClip Week3 -0.42806 0.219 Inf -1.950
 WT WingRemoved Week2 - WT WingClip Week3 -0.28500 0.172 Inf -1.660
 WT WingRemoved Week2 - Orco WingRemoved Week3 -0.40787 0.172 Inf -2.377
 WT WingRemoved Week2 - WT WingRemoved Week3 -0.26481 0.108 Inf -2.456
 WT WingRemoved Week2 - Orco WingClip Week4 -0.29204 0.223 Inf -1.310
 WT WingRemoved Week2 - WT WingClip Week4 -0.14899 0.176 Inf -0.849
 WT WingRemoved Week2 - Orco WingRemoved Week4 -0.27185 0.176 Inf -1.545
 WT WingRemoved Week2 - WT WingRemoved Week4 -0.12879 0.114 Inf -1.133
 WT WingRemoved Week2 - Orco WingClip Week5 -0.63223 0.220 Inf -2.872
 WT WingRemoved Week2 - WT WingClip Week5 -0.48918 0.171 Inf -2.853
 WT WingRemoved Week2 - Orco WingRemoved Week5 -0.61204 0.172 Inf -3.555
 WT WingRemoved Week2 - WT WingRemoved Week5 -0.46898 0.107 Inf -4.380
 Orco WingClip Week3 - WT WingClip Week3 0.14306 0.133 Inf 1.075
 Orco WingClip Week3 - Orco WingRemoved Week3 0.02019 0.133 Inf 0.151
 Orco WingClip Week3 - WT WingRemoved Week3 0.16325 0.191 Inf 0.856
 Orco WingClip Week3 - Orco WingClip Week4 0.13602 0.108 Inf 1.256
 Orco WingClip Week3 - WT WingClip Week4 0.27907 0.171 Inf 1.632
 Orco WingClip Week3 - Orco WingRemoved Week4 0.15621 0.172 Inf 0.909
 Orco WingClip Week3 - WT WingRemoved Week4 0.29927 0.219 Inf 1.368
 Orco WingClip Week3 - Orco WingClip Week5 -0.20417 0.101 Inf -2.014
 Orco WingClip Week3 - WT WingClip Week5 -0.06112 0.166 Inf -0.368
 Orco WingClip Week3 - Orco WingRemoved Week5 -0.18398 0.167 Inf -1.099
 Orco WingClip Week3 - WT WingRemoved Week5 -0.04092 0.215 Inf -0.190
 WT WingClip Week3 - Orco WingRemoved Week3 -0.12286 0.186 Inf -0.660
 WT WingClip Week3 - WT WingRemoved Week3 0.02019 0.133 Inf 0.151
 WT WingClip Week3 - Orco WingClip Week4 -0.00704 0.172 Inf -0.041
 WT WingClip Week3 - WT WingClip Week4 0.13602 0.108 Inf 1.256
 WT WingClip Week3 - Orco WingRemoved Week4 0.01316 0.216 Inf 0.061
 WT WingClip Week3 - WT WingRemoved Week4 0.15621 0.172 Inf 0.909
 WT WingClip Week3 - Orco WingClip Week5 -0.34723 0.168 Inf -2.063
 WT WingClip Week3 - WT WingClip Week5 -0.20417 0.101 Inf -2.014
 WT WingClip Week3 - Orco WingRemoved Week5 -0.32703 0.213 Inf -1.538
 WT WingClip Week3 - WT WingRemoved Week5 -0.18398 0.167 Inf -1.099
 Orco WingRemoved Week3 - WT WingRemoved Week3 0.14306 0.133 Inf 1.075
 Orco WingRemoved Week3 - Orco WingClip Week4 0.11582 0.172 Inf 0.674
 Orco WingRemoved Week3 - WT WingClip Week4 0.25888 0.215 Inf 1.205
 Orco WingRemoved Week3 - Orco WingRemoved Week4 0.13602 0.108 Inf 1.256
 Orco WingRemoved Week3 - WT WingRemoved Week4 0.27907 0.171 Inf 1.632
 Orco WingRemoved Week3 - Orco WingClip Week5 -0.22437 0.168 Inf -1.337
 Orco WingRemoved Week3 - WT WingClip Week5 -0.08131 0.211 Inf -0.385
 Orco WingRemoved Week3 - Orco WingRemoved Week5 -0.20417 0.101 Inf -2.014
 Orco WingRemoved Week3 - WT WingRemoved Week5 -0.06112 0.166 Inf -0.368
 WT WingRemoved Week3 - Orco WingClip Week4 -0.02723 0.220 Inf -0.124
 WT WingRemoved Week3 - WT WingClip Week4 0.11582 0.172 Inf 0.674
 WT WingRemoved Week3 - Orco WingRemoved Week4 -0.00704 0.172 Inf -0.041
 WT WingRemoved Week3 - WT WingRemoved Week4 0.13602 0.108 Inf 1.256
 WT WingRemoved Week3 - Orco WingClip Week5 -0.36742 0.217 Inf -1.693
 WT WingRemoved Week3 - WT WingClip Week5 -0.22437 0.168 Inf -1.337
 WT WingRemoved Week3 - Orco WingRemoved Week5 -0.34723 0.168 Inf -2.063
 WT WingRemoved Week3 - WT WingRemoved Week5 -0.20417 0.101 Inf -2.014
 Orco WingClip Week4 - WT WingClip Week4 0.14306 0.133 Inf 1.075
 Orco WingClip Week4 - Orco WingRemoved Week4 0.02019 0.133 Inf 0.151
 Orco WingClip Week4 - WT WingRemoved Week4 0.16325 0.191 Inf 0.856
 Orco WingClip Week4 - Orco WingClip Week5 -0.34019 0.107 Inf -3.191
 Orco WingClip Week4 - WT WingClip Week5 -0.19713 0.170 Inf -1.159
 Orco WingClip Week4 - Orco WingRemoved Week5 -0.31999 0.171 Inf -1.875
 Orco WingClip Week4 - WT WingRemoved Week5 -0.17694 0.218 Inf -0.811
 WT WingClip Week4 - Orco WingRemoved Week4 -0.12286 0.186 Inf -0.660
 WT WingClip Week4 - WT WingRemoved Week4 0.02019 0.133 Inf 0.151
 WT WingClip Week4 - Orco WingClip Week5 -0.48324 0.171 Inf -2.827
 WT WingClip Week4 - WT WingClip Week5 -0.34019 0.107 Inf -3.191
 WT WingClip Week4 - Orco WingRemoved Week5 -0.46305 0.215 Inf -2.157
 WT WingClip Week4 - WT WingRemoved Week5 -0.31999 0.171 Inf -1.875
 Orco WingRemoved Week4 - WT WingRemoved Week4 0.14306 0.133 Inf 1.075
 Orco WingRemoved Week4 - Orco WingClip Week5 -0.36038 0.171 Inf -2.109
 Orco WingRemoved Week4 - WT WingClip Week5 -0.21733 0.214 Inf -1.015
 Orco WingRemoved Week4 - Orco WingRemoved Week5 -0.34019 0.107 Inf -3.191
 Orco WingRemoved Week4 - WT WingRemoved Week5 -0.19713 0.170 Inf -1.159
 WT WingRemoved Week4 - Orco WingClip Week5 -0.50344 0.219 Inf -2.299
 WT WingRemoved Week4 - WT WingClip Week5 -0.36038 0.171 Inf -2.109
 WT WingRemoved Week4 - Orco WingRemoved Week5 -0.48324 0.171 Inf -2.827
 WT WingRemoved Week4 - WT WingRemoved Week5 -0.34019 0.107 Inf -3.191
 Orco WingClip Week5 - WT WingClip Week5 0.14306 0.133 Inf 1.075
 Orco WingClip Week5 - Orco WingRemoved Week5 0.02019 0.133 Inf 0.151
 Orco WingClip Week5 - WT WingRemoved Week5 0.16325 0.191 Inf 0.856
 WT WingClip Week5 - Orco WingRemoved Week5 -0.12286 0.186 Inf -0.660
 WT WingClip Week5 - WT WingRemoved Week5 0.02019 0.133 Inf 0.151
 Orco WingRemoved Week5 - WT WingRemoved Week5 0.14306 0.133 Inf 1.075
 p.value
 1.0000
 1.0000
 1.0000
 1.0000
 0.9990
 1.0000
 0.9999
 0.9762
 1.0000
 1.0000
 1.0000
 1.0000
 1.0000
 1.0000
 1.0000
 0.0288
 0.9971
 0.8279
 1.0000
 1.0000
 1.0000
 1.0000
 1.0000
 1.0000
 1.0000
 0.9349
 0.9762
 0.9976
 1.0000
 0.9999
 1.0000
 1.0000
 1.0000
 0.1778
 0.0288
 0.6829
 0.8279
 1.0000
 1.0000
 1.0000
 1.0000
 0.9990
 0.9998
 1.0000
 0.9762
 1.0000
 1.0000
 1.0000
 1.0000
 1.0000
 0.6658
 0.9996
 0.0288
 0.9971
 1.0000
 1.0000
 1.0000
 1.0000
 0.9909
 0.9998
 0.9349
 0.9762
 1.0000
 1.0000
 0.9999
 1.0000
 0.5734
 0.6658
 0.1778
 0.0288
 1.0000
 1.0000
 1.0000
 0.6185
 1.0000
 0.9974
 1.0000
 0.9999
 1.0000
 1.0000
 1.0000
 0.0020
 0.9308
 0.4818
 0.9978
 1.0000
 1.0000
 0.6789
 0.6185
 0.9626
 0.9974
 0.9930
 0.9999
 0.9999
 1.0000
 0.0482
 0.0020
 0.3932
 0.4818
 1.0000
 0.9841
 1.0000
 0.6185
 1.0000
 1.0000
 1.0000
 0.9999
 1.0000
 0.3195
 0.9880
 0.0020
 0.9308
 0.9219
 0.9841
 0.6789
 0.6185
 0.9992
 1.0000
 0.9930
 0.9999
 0.3070
 0.3195
 0.0482
 0.0020
 1.0000
 1.0000
 1.0000
 0.9995
 0.9868
 1.0000
 0.9985
 0.8976
 1.0000
 0.9999
 1.0000
 1.0000
 1.0000
 1.0000
 0.9995
 1.0000
 1.0000
 0.8759
 0.8976
 0.9934
 0.9999
 1.0000
 1.0000
 0.9997
 0.9995
 0.9868
 0.9989
 1.0000
 0.8976
 1.0000
 1.0000
 1.0000
 1.0000
 0.9995
 0.9803
 0.9989
 0.8759
 0.8976
 1.0000
 1.0000
 1.0000
 0.1430
 0.9999
 0.9451
 1.0000
 1.0000
 1.0000
 0.3370
 0.1430
 0.8266
 0.9451
 1.0000
 0.8528
 1.0000
 0.1430
 0.9999
 0.7353
 0.8528
 0.3370
 0.1430
 1.0000
 1.0000
 1.0000
 1.0000
 1.0000
 1.0000

Results are given on the log (not the response) scale.
P value adjustment: tukey method for comparing a family of 20 estimates

lsmeans(OrcoWBL_glmmTMB2, pairwise ~ Focal_Treatment + Rival_Treatment, adjust="tukey")

$lsmeans
 Focal_Treatment Rival_Treatment lsmean SE df asymp.LCL asymp.UCL
 Orco WingClip -0.146 0.116 Inf -0.373 0.0819
 WT WingClip -0.289 0.116 Inf -0.516 -0.0612
 Orco WingRemoved -0.166 0.118 Inf -0.396 0.0647
 WT WingRemoved -0.309 0.121 Inf -0.546 -0.0712

Results are averaged over the levels of: Week
Results are given on the log (not the response) scale.
Confidence level used: 0.95

$contrasts
 contrast estimate SE df z.ratio p.value
 Orco WingClip - WT WingClip 0.1431 0.133 Inf 1.075 0.7047
 Orco WingClip - Orco WingRemoved 0.0202 0.133 Inf 0.151 0.9988
 Orco WingClip - WT WingRemoved 0.1632 0.191 Inf 0.856 0.8275
 WT WingClip - Orco WingRemoved -0.1229 0.186 Inf -0.660 0.9119
 WT WingClip - WT WingRemoved 0.0202 0.133 Inf 0.151 0.9988
 Orco WingRemoved - WT WingRemoved 0.1431 0.133 Inf 1.075 0.7047

Results are averaged over the levels of: Week
Results are given on the log (not the response) scale.
P value adjustment: tukey method for comparing a family of 4 estimates

lsmeans(OrcoWBL_glmmTMB2, pairwise ~ Focal_Treatment, adjust="tukey")

$lsmeans
 Focal_Treatment lsmean SE df asymp.LCL asymp.UCL
 Orco -0.156 0.0959 Inf -0.344 0.0323
 WT -0.299 0.0981 Inf -0.491 -0.1064

Results are averaged over the levels of: Rival_Treatment, Week
Results are given on the log (not the response) scale.
Confidence level used: 0.95

$contrasts
 contrast estimate SE df z.ratio p.value
 Orco - WT 0.143 0.133 Inf 1.075 0.2823

Results are averaged over the levels of: Rival_Treatment, Week
Results are given on the log (not the response) scale.

lsmeans(OrcoWBL_glmmTMB2, pairwise ~ Rival_Treatment, adjust="tukey")

$lsmeans
 Rival_Treatment lsmean SE df asymp.LCL asymp.UCL
 WingClip -0.217 0.0951 Inf -0.403 -0.0308
 WingRemoved -0.237 0.0992 Inf -0.432 -0.0429

Results are averaged over the levels of: Focal_Treatment, Week
Results are given on the log (not the response) scale.
Confidence level used: 0.95

$contrasts
 contrast estimate SE df z.ratio p.value
 WingClip - WingRemoved 0.0202 0.133 Inf 0.151 0.8797

Results are averaged over the levels of: Focal_Treatment, Week
Results are given on the log (not the response) scale.

lsmeans(OrcoWBL_glmmTMB2, pairwise ~ Week, adjust="tukey")

$lsmeans
 Week lsmean SE df asymp.LCL asymp.UCL
 1 -0.3319 0.0964 Inf -0.521 -0.1429
 2 -0.4167 0.1009 Inf -0.614 -0.2190
 3 -0.1518 0.0953 Inf -0.339 0.0349
 4 -0.2879 0.1019 Inf -0.488 -0.0881
 5 0.0523 0.0950 Inf -0.134 0.2384

Results are averaged over the levels of: Focal_Treatment, Rival_Treatment
Results are given on the log (not the response) scale.
Confidence level used: 0.95

$contrasts
 contrast estimate SE df z.ratio p.value
 Week1 - Week2 0.0848 0.109 Inf 0.781 0.9362
 Week1 - Week3 -0.1800 0.104 Inf -1.724 0.4193
 Week1 - Week4 -0.0440 0.110 Inf -0.398 0.9947
 Week1 - Week5 -0.3842 0.104 Inf -3.707 0.0020
 Week2 - Week3 -0.2648 0.108 Inf -2.456 0.1009
 Week2 - Week4 -0.1288 0.114 Inf -1.133 0.7892
 Week2 - Week5 -0.4690 0.107 Inf -4.380 0.0001
 Week3 - Week4 0.1360 0.108 Inf 1.256 0.7184
 Week3 - Week5 -0.2042 0.101 Inf -2.014 0.2593
 Week4 - Week5 -0.3402 0.107 Inf -3.191 0.0123

Results are averaged over the levels of: Focal_Treatment, Rival_Treatment
Results are given on the log (not the response) scale.
P value adjustment: tukey method for comparing a family of 5 estimates

##Orco WBL Line
Orco_WBL$PercentSuccess <- Orco_WBL$Success / 10
Orco_WBL_subset <- subset(Orco_WBL, PercentSuccess > 0)
Orco_WBL_subset$Rival_Treatment <- factor(Orco_WBL_subset$Rival_Treatment, levels = c("WingClip", "WingRemoved"))
Orco_WBL_subset$Focal_Treatment <- factor(Orco_WBL_subset$Focal_Treatment, levels = c("WT", "Orco"))
Orco_WBL_subset$Interaction <- interaction(Orco_WBL_subset$Focal_Treatment, Orco_WBL_subset$Rival_Treatment)
Orco_WBL_subset$Interaction <- factor(Orco_WBL_subset$Interaction, levels = c("WT.WingClip", "WT.WingRemoved", "Orco.WingClip", "Orco.WingRemoved"))

Orco_WBL_Summary <- summarySE(Orco_WBL_subset,
 measurevar = "PercentSuccess",
 groupvars = c("Week", "Interaction", "Focal_Treatment", "Rival_Treatment"))

Orco_WBL_Line <- ggplot(Orco_WBL_Summary) +
 aes(
 x = Week,
 y = PercentSuccess,
 colour = Interaction,
 group = Interaction
 ) +
 scale_colour_manual(name = "Treatment",
 labels = c("Wildtype kept with Wing Clipped Rival",
 "Wildtype kept with Wing Removed Rival",
 "Orco2 kept with Wing Clipped Rival",
 "Orco2 kept with Wing Removed Rival"),
 values = c("grey70",
 "grey42",
 "grey70",
 "grey42")) +
 geom_line(aes(linetype = Interaction), size = 3) +
 geom_point() +
 geom_errorbar(aes(ymin=PercentSuccess-se, ymax=PercentSuccess+se), width=0.2, size = 1.5) +
 scale_linetype_manual(name = "Treatment",
 labels = c("Wildtype kept with Wing Clipped Rival",
 "Wildtype kept with Wing Removed Rival",
 "Orco2 kept with Wing Clipped Rival",
 "Orco2 kept with Wing Removed Rival"),
 values = c("solid", "solid", "dotdash", "dotdash")) +
 labs(
 x = "Week post Eclosion",
 y = "Proportion of Time Spent with a Body Length of a Rival"
 ) +
 ylim(0, 0.55) +
 theme_bw() +
 guides(colour = guide_legend(override.aes = list(linetype = c("solid", "solid","dotdash", "dotdash"))))

Orco_WBL_Line_Plot <- Orco_WBL_Line + theme_bw() + theme(panel.grid.major = element_blank(), panel.grid.minor = element_blank()) +
 theme(axis.text=element_text(size=55, face="bold", colour="black"),
 axis.title=element_text(size=75, face="bold", colour="black"),
 legend.text=element_text(size=55, face="bold", colour="black"),
 legend.title=element_text(size=75, face="bold", colour="black"),
 strip.text=element_text(size=75, face="bold", colour="black")) + theme(axis.line = element_line(size = 5), panel.border = element_rect(size = 5)) + theme( axis.ticks.length = unit(0.5, "cm"), axis.ticks = element_line(size = 5))
Orco_WBL_Line_Plot


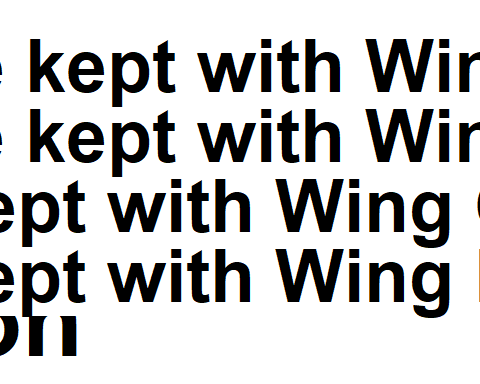


###Final Figure Diagrams
Orco_movement_plots <- ggarrange(Orco_Food_Line_Plot, Orco_Walking_Line_Plot, Orco_Inactive_Line_Plot,
 labels = c("A", "B", "C"),
 font.label = list(size = 80),
 ncol = 1, nrow = 3,
 common.legend = TRUE, legend = "right")
Orco_movement_plots


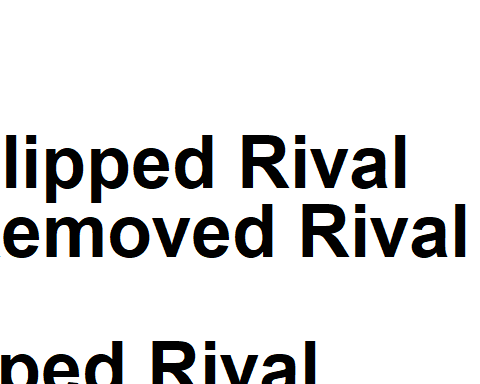


Orco_rival_plots <- ggarrange(Orco_Aggression_Line_Plot, Orco_WBL_Line_Plot,
 labels = c("A", "B"),
 font.label = list(size = 80),
 ncol=1, nrow = 2,
 common.legend = TRUE, legend = "right")
Orco_rival_plots


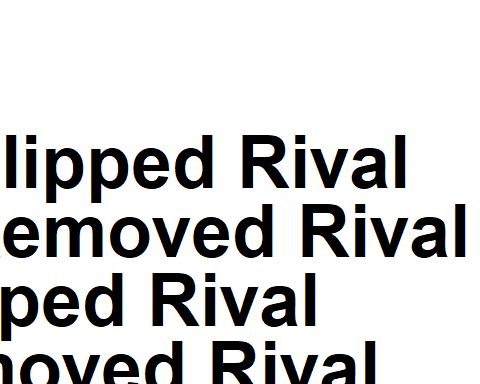


##Effect Sizes
#Food
Orco_group_stats_Food <- Orco_Food %>%
 group_by(Focal_Treatment, Rival_Treatment) %>%
 dplyr::summarise(mean = mean(Success),
 sd = sd(Success),
 N = n())

`summarise()` has grouped output by 'Focal_Treatment'. You can override using
the `.groups` argument.

OrcoExpEMD_WT_Single_Food <- Orco_Food %>%
 filter(Rival_Treatment == "Single", Focal_Treatment == "WT") %>%
 select(Success)
OrcoExpEMD_WT_WC_Food <- Orco_Food %>%
 filter(Rival_Treatment == "WingClip", Focal_Treatment == "WT") %>%
 select(Success)
OrcoExpEMD_WT_WR_Food <- Orco_Food %>%
 filter(Rival_Treatment == "WingRemoved", Focal_Treatment == "WT") %>%
 select(Success)
cohen.d(OrcoExpEMD_WT_Single_Food$Success, OrcoExpEMD_WT_WC_Food$Success)

Cohen's d

d estimate: -1.033034 (large)
95 percent confidence interval:
 lower upper
-1.2335435 -0.8325236

cohen.d(OrcoExpEMD_WT_Single_Food$Success, OrcoExpEMD_WT_WR_Food$Success)

Cohen's d

d estimate: -0.6098434 (medium)
95 percent confidence interval:
 lower upper
-0.8056117 -0.4140751

cohen.d(OrcoExpEMD_WT_WR_Food$Success, OrcoExpEMD_WT_WC_Food$Success)

Cohen's d

d estimate: -0.3847736 (small)
95 percent confidence interval:
 lower upper
-0.5748593 -0.1946879

OrcoExpEMD_Orco_Single_Food <- Orco_Food %>%
 filter(Rival_Treatment == "Single", Focal_Treatment == "Orco") %>%
 select(Success)
OrcoExpEMD_Orco_WC_Food <- Orco_Food %>%
 filter(Rival_Treatment == "WingClip", Focal_Treatment == "Orco") %>%
 select(Success)
OrcoExpEMD_Orco_WR_Food <- Orco_Food %>%
 filter(Rival_Treatment == "WingRemoved", Focal_Treatment == "Orco") %>%
 select(Success)
cohen.d(OrcoExpEMD_Orco_Single_Food$Success, OrcoExpEMD_Orco_WC_Food$Success)

Cohen's d

d estimate: -0.4550067 (small)
95 percent confidence interval:
 lower upper
-0.6419869 -0.2680264

cohen.d(OrcoExpEMD_Orco_Single_Food$Success, OrcoExpEMD_Orco_WR_Food$Success)

Cohen's d

d estimate: -0.5619479 (medium)
95 percent confidence interval:
 lower upper
-0.7501611 -0.3737348

cohen.d(OrcoExpEMD_Orco_WR_Food$Success, OrcoExpEMD_Orco_WC_Food$Success)

Cohen's d

d estimate: 0.1284611 (negligible)
95 percent confidence interval:
 lower upper
-0.05998848 0.31691064

#Walking
Orco_group_stats_Walking <- Orco_Walking %>%
 group_by(Focal_Treatment, Rival_Treatment) %>%
 dplyr::summarise(mean = mean(Success),
 sd = sd(Success),
 N = n())

`summarise()` has grouped output by 'Focal_Treatment'. You can override using
the `.groups` argument.

OrcoExpEMD_WT_Single_Walking <- Orco_Walking %>%
 filter(Rival_Treatment == "Single", Focal_Treatment == "WT") %>%
 select(Success)
OrcoExpEMD_WT_WC_Walking <- Orco_Walking %>%
 filter(Rival_Treatment == "WingClip", Focal_Treatment == "WT") %>%
 select(Success)
OrcoExpEMD_WT_WR_Walking <- Orco_Walking %>%
 filter(Rival_Treatment == "WingRemoved", Focal_Treatment == "WT") %>%
 select(Success)
cohen.d(OrcoExpEMD_WT_Single_Walking$Success, OrcoExpEMD_WT_WC_Walking$Success)

Cohen's d

d estimate: 0.5672294 (medium)
95 percent confidence interval:
 lower upper
0.3751303 0.7593285

cohen.d(OrcoExpEMD_WT_Single_Walking$Success, OrcoExpEMD_WT_WR_Walking$Success)

Cohen's d

d estimate: 0.5695624 (medium)
95 percent confidence interval:
 lower upper
0.3743503 0.7647745

cohen.d(OrcoExpEMD_WT_WR_Walking$Success, OrcoExpEMD_WT_WC_Walking$Success)

Cohen's d

d estimate: -0.014514 (negligible)
95 percent confidence interval:
 lower upper
-0.2028691 0.1738411

OrcoExpEMD_Orco_Single_Walking <- Orco_Walking %>%
 filter(Rival_Treatment == "Single", Focal_Treatment == "Orco") %>%
 select(Success)
OrcoExpEMD_Orco_WC_Walking <- Orco_Walking %>%
 filter(Rival_Treatment == "WingClip", Focal_Treatment == "Orco") %>%
 select(Success)
OrcoExpEMD_Orco_WR_Walking <- Orco_Walking %>%
 filter(Rival_Treatment == "WingRemoved", Focal_Treatment == "Orco") %>%
 select(Success)
cohen.d(OrcoExpEMD_Orco_Single_Walking$Success, OrcoExpEMD_Orco_WC_Walking$Success)

Cohen's d

d estimate: 0.1079494 (negligible)
95 percent confidence interval:
 lower upper
-0.07679518 0.29269407

cohen.d(OrcoExpEMD_Orco_Single_Walking$Success, OrcoExpEMD_Orco_WR_Walking$Success)

Cohen's d

d estimate: 0.08020302 (negligible)
95 percent confidence interval:
 lower upper
-0.1044815 0.2648875

cohen.d(OrcoExpEMD_Orco_WR_Walking$Success, OrcoExpEMD_Orco_WC_Walking$Success)

Cohen's d

d estimate: 0.03201246 (negligible)
95 percent confidence interval:
 lower upper
-0.1562551 0.2202800

#Grooming
Orco_group_stats_Grooming <- Orco_Grooming %>%
 group_by(Focal_Treatment, Rival_Treatment) %>%
 dplyr::summarise(mean = mean(Success),
 sd = sd(Success),
 N = n())

`summarise()` has grouped output by 'Focal_Treatment'. You can override using
the `.groups` argument.

OrcoExpEMD_WT_Single_Grooming <- Orco_Grooming %>%
 filter(Rival_Treatment == "Single", Focal_Treatment == "WT") %>%
 select(Success)
OrcoExpEMD_WT_WC_Grooming <- Orco_Grooming %>%
 filter(Rival_Treatment == "WingClip", Focal_Treatment == "WT") %>%
 select(Success)
OrcoExpEMD_WT_WR_Grooming <- Orco_Grooming %>%
 filter(Rival_Treatment == "WingRemoved", Focal_Treatment == "WT") %>%
 select(Success)
cohen.d(OrcoExpEMD_WT_Single_Grooming$Success, OrcoExpEMD_WT_WC_Grooming$Success)

Cohen's d

d estimate: 0.3273107 (small)
95 percent confidence interval:
 lower upper
0.1377024 0.5169190

cohen.d(OrcoExpEMD_WT_Single_Grooming$Success, OrcoExpEMD_WT_WR_Grooming$Success)

Cohen's d

d estimate: 0.2906115 (small)
95 percent confidence interval:
 lower upper
0.09823348 0.48298960

cohen.d(OrcoExpEMD_WT_WR_Grooming$Success, OrcoExpEMD_WT_WC_Grooming$Success)

Cohen's d

d estimate: 0.03212875 (negligible)
95 percent confidence interval:
 lower upper
-0.1562360 0.2204935

OrcoExpEMD_Orco_Single_Grooming <- Orco_Grooming %>%
 filter(Rival_Treatment == "Single", Focal_Treatment == "Orco") %>%
 select(Success)
OrcoExpEMD_Orco_WC_Grooming <- Orco_Grooming %>%
 filter(Rival_Treatment == "WingClip", Focal_Treatment == "Orco") %>%
 select(Success)
OrcoExpEMD_Orco_WR_Grooming <- Orco_Grooming %>%
 filter(Rival_Treatment == "WingRemoved", Focal_Treatment == "Orco") %>%
 select(Success)
cohen.d(OrcoExpEMD_Orco_Single_Grooming$Success, OrcoExpEMD_Orco_WC_Grooming$Success)

Cohen's d

d estimate: 0.07497999 (negligible)
95 percent confidence interval:
 lower upper
-0.1096952 0.2596552

cohen.d(OrcoExpEMD_Orco_Single_Grooming$Success, OrcoExpEMD_Orco_WR_Grooming$Success)

Cohen's d

d estimate: 0.03475173 (negligible)
95 percent confidence interval:
 lower upper
-0.1498726 0.2193761

cohen.d(OrcoExpEMD_Orco_WR_Grooming$Success, OrcoExpEMD_Orco_WC_Grooming$Success)

Cohen's d

d estimate: 0.04621498 (negligible)
95 percent confidence interval:
 lower upper
-0.1420656 0.2344956

#Inactive
Orco_group_stats_Inactive <- Orco_Inactive %>%
 group_by(Focal_Treatment, Rival_Treatment) %>%
 dplyr::summarise(mean = mean(Success),
 sd = sd(Success),
 N = n())

`summarise()` has grouped output by 'Focal_Treatment'. You can override using
the `.groups` argument.

OrcoExpEMD_WT_Single_Inactive <- Orco_Inactive %>%
 filter(Rival_Treatment == "Single", Focal_Treatment == "WT") %>%
 select(Success)
OrcoExpEMD_WT_WC_Inactive <- Orco_Inactive %>%
 filter(Rival_Treatment == "WingClip", Focal_Treatment == "WT") %>%
 select(Success)
OrcoExpEMD_WT_WR_Inactive <- Orco_Inactive %>%
 filter(Rival_Treatment == "WingRemoved", Focal_Treatment == "WT") %>%
 select(Success)
cohen.d(OrcoExpEMD_WT_Single_Inactive$Success, OrcoExpEMD_WT_WC_Inactive$Success)

Cohen's d

d estimate: 0.3041333 (small)
95 percent confidence interval:
 lower upper
0.1146960 0.4935705

cohen.d(OrcoExpEMD_WT_Single_Inactive$Success, OrcoExpEMD_WT_WR_Inactive$Success)

Cohen's d

d estimate: -0.09721822 (negligible)
95 percent confidence interval:
 lower upper
-0.28870181 0.09426537

cohen.d(OrcoExpEMD_WT_WR_Inactive$Success, OrcoExpEMD_WT_WC_Inactive$Success)

Cohen's d

d estimate: 0.388038 (small)
95 percent confidence interval:
 lower upper
0.1979229 0.5781531

OrcoExpEMD_Orco_Single_Inactive <- Orco_Inactive %>%
 filter(Rival_Treatment == "Single", Focal_Treatment == "Orco") %>%
 select(Success)
OrcoExpEMD_Orco_WC_Inactive <- Orco_Inactive %>%
 filter(Rival_Treatment == "WingClip", Focal_Treatment == "Orco") %>%
 select(Success)
OrcoExpEMD_Orco_WR_Inactive <- Orco_Inactive %>%
 filter(Rival_Treatment == "WingRemoved", Focal_Treatment == "Orco") %>%
 select(Success)
cohen.d(OrcoExpEMD_Orco_Single_Inactive$Success, OrcoExpEMD_Orco_WC_Inactive$Success)

Cohen's d

d estimate: 0.3538413 (small)
95 percent confidence interval:
 lower upper
0.1677941 0.5398885

cohen.d(OrcoExpEMD_Orco_Single_Inactive$Success, OrcoExpEMD_Orco_WR_Inactive$Success)

Cohen's d

d estimate: 0.4649522 (small)
95 percent confidence interval:
 lower upper
0.2778679 0.6520364

cohen.d(OrcoExpEMD_Orco_WR_Inactive$Success, OrcoExpEMD_Orco_WC_Inactive$Success)

Cohen's d

d estimate: -0.1259377 (negligible)
95 percent confidence interval:
 lower upper
-0.31437968 0.06250435

#Aggression
Orco_group_stats_Aggression <- Orco_Aggression %>%
 group_by(Focal_Treatment, Rival_Treatment) %>%
 dplyr::summarise(mean = mean(Success),
 sd = sd(Success),
 N = n())

`summarise()` has grouped output by 'Focal_Treatment'. You can override using
the `.groups` argument.

OrcoExpEMD_WT_WC_Aggression <- Orco_Aggression %>%
 filter(Rival_Treatment == "WingClip", Focal_Treatment == "WT") %>%
 select(Success)
OrcoExpEMD_WT_WR_Aggression <- Orco_Aggression %>%
 filter(Rival_Treatment == "WingRemoved", Focal_Treatment == "WT") %>%
 select(Success)
cohen.d(OrcoExpEMD_WT_WR_Aggression$Success, OrcoExpEMD_WT_WC_Aggression$Success)

Cohen's d

d estimate: -0.02792672 (negligible)
95 percent confidence interval:
 lower upper
-0.2162885 0.1604351

OrcoExpEMD_Orco_WC_Aggression <- Orco_Aggression %>%
 filter(Rival_Treatment == "WingClip", Focal_Treatment == "Orco") %>%
 select(Success)
OrcoExpEMD_Orco_WR_Aggression <- Orco_Aggression %>%
 filter(Rival_Treatment == "WingRemoved", Focal_Treatment == "Orco") %>%
 select(Success)
cohen.d(OrcoExpEMD_Orco_WR_Aggression$Success, OrcoExpEMD_Orco_WC_Aggression$Success)

Cohen's d

d estimate: -0.2962815 (small)
95 percent confidence interval:
 lower upper
-0.485567 -0.106996

#WBL
Orco_group_stats_WBL <- Orco_WBL %>%
 group_by(Focal_Treatment, Rival_Treatment) %>%
 dplyr::summarise(mean = mean(Success),
 sd = sd(Success),
 N = n())

`summarise()` has grouped output by 'Focal_Treatment'. You can override using
the `.groups` argument.

OrcoExpEMD_WT_WC_WBL <- Orco_WBL %>%
 filter(Rival_Treatment == "WingClip", Focal_Treatment == "WT") %>%
 select(Success)
OrcoExpEMD_WT_WR_WBL <- Orco_WBL %>%
 filter(Rival_Treatment == "WingRemoved", Focal_Treatment == "WT") %>%
 select(Success)
cohen.d(OrcoExpEMD_WT_WR_WBL$Success, OrcoExpEMD_WT_WC_WBL$Success)

Cohen's d

d estimate: -0.09538272 (negligible)
95 percent confidence interval:
 lower upper
-0.28384230 0.09307686

OrcoExpEMD_Orco_WC_WBL <- Orco_WBL %>%
 filter(Rival_Treatment == "WingClip", Focal_Treatment == "Orco") %>%
 select(Success)
OrcoExpEMD_Orco_WR_WBL <- Orco_WBL %>%
 filter(Rival_Treatment == "WingRemoved", Focal_Treatment == "Orco") %>%
 select(Success)
cohen.d(OrcoExpEMD_Orco_WR_WBL$Success, OrcoExpEMD_Orco_WC_WBL$Success)

Cohen's d

d estimate: 0.1462899 (negligible)
95 percent confidence interval:
 lower upper
-0.0422172 0.3347970

# Antennae Removed

Antennae Removed flies had their entire antennae removed 24h after eclosion under CO2 anesthesia. Only flies that woke up after their surgery were used in these experiments.

## Extended Mating Duration

**Figure 1B**

**Description of Data:** Experiment on mating duration comparing the mating times of WT and flies with Antennae Removed flies when kept solo versus when they were with a rival having a clipped wing or a completely removed wing.

**Factors:**

- FlyID-Unique ID for each fly within experiment
- DateEclosed-Date fly eclosed as an adult
- DateExperiment-Date mating experiment performed
- FocalTreatment-Manipulation to the focal fly. (WT=not manipulated, AntennaeRemoved=antennae surgically removed 24h post eclosion)
- RivalTreatment-Social Treatment. Single=focal fly kept in isolation post eclosion, WingClip=fly kept with single rival with a wing clip from eclosion to mating, WingRemoved=fly kept with a rival with wings surgical removed
- Treatment-Interaction between Focal and Rival Treatments
- Mated?-Flies mated within 3h (Y= succesfully mated, N= didn't mate within 3h, NA = excluded from experiment)
- TimeUp-Time when male aspirated into vial with female (to nearest minute)
- TimeMatingStart-Time when mating started (to nearest minute)
- TimeMatingEnd-Time when mating ended (to nearest minute)
- MatingLatency-Time taken to start mating (TimeMatingStart - TimeUp)
- MatingDuration-Mating Duration (TimeMatingEnd - TimeMatingStart)within a ten minute period (one scan per minute)

##Library
library(emmeans)
library(lme4)
library(ggplot2)
library(tidyverse)
library(ggsignif)
library(AER)
library(effsize)

##Importing Data
AntennaeEMD1 <- read.csv("Antennae_EMD_5.7.22.csv")
AntennaeEMD2 <- read.csv("Antennae_EMD_13.7.22.csv")
AntennaeEMD3 <- read.csv("Antennae_EMD_26.8.22.csv")

##Combining Data
AntennaeEMD_Full <- bind_rows(AntennaeEMD1, AntennaeEMD2, AntennaeEMD3)

#Removing Outliers
AntennaeEMD_5 <- subset(AntennaeEMD_Full, MatingDuration > 4)
AntennaeEMD_60 <- subset(AntennaeEMD_Full, MatingDuration < 50)
AntennaeEMD_All <- subset(AntennaeEMD_60, Mated. == "Y")

##Plotting the Data
#Ordering Factors2
AntennaeEMD_All$RivalTreatment <- factor(AntennaeEMD_All$RivalTreatment, levels = c("Single", "WingClip", "WingRemoved"))
AntennaeEMD_All$FocalTreatment <- factor(AntennaeEMD_All$FocalTreatment, levels = c("WT", "AntennaeRemoved"))


#Box Plot
#Box Plot
AntennaeEMD_All$Treatment <- gsub(" ", "", AntennaeEMD_All$Treatment)
AntennaeEMD_All$Treatment <- factor(AntennaeEMD_All$Treatment, levels = c("WTSingle", "WTWingClip", "WTWingRemoved", "AntennaeRemovedSingle", "AntennaeRemovedWingClip", "AntennaeRemovedWingRemoved"))
AntennaeEMD_All$RivalTreatment <- factor(AntennaeEMD_All$RivalTreatment, levels = c("Single", "WingClip", "WingRemoved"))
AntennaeEMD_All$FocalTreatment <- factor(AntennaeEMD_All$FocalTreatment, levels = c("WT", "AntennaeRemoved"))


Antennae_matingduration_sample_sizes <- AntennaeEMD_All %>%
 group_by(FocalTreatment, RivalTreatment, Treatment) %>%
 dplyr::summarise(
 Count = n(),
 Mean_MatingDuration = mean(MatingDuration)
 )

`summarise()` has grouped output by 'FocalTreatment', 'RivalTreatment'. You can
override using the `.groups` argument.

Antennae_matingduration_sample_sizes$Count <- as.character(Antennae_matingduration_sample_sizes$Count)

boxplot_Antennae_matingduration_bw <- ggplot(AntennaeEMD_All) +
 aes(
 x = FocalTreatment,
 y = MatingDuration,
 fill = RivalTreatment
 ) +
 geom_boxplot(size = 5) +
 scale_fill_manual(
 values = c(Single = "white",
 WingClip = "grey70",
 WingRemoved = "grey25"),
 labels=c("Single" = "Single",
 "WingClip" = "Wing Clipped",
 "WingRemoved" = "Wing Removed")
 ) +
 labs(
 x = "Focal Treatment",
 y = "Mating Duration (mins)",
 fill = "Social Treatment"
 ) +
 ylim(0,49)+
 geom_signif(y_position = c(43, 47, 43),
 xmin = c(0.75, 0.75, 1.75),
 xmax = c(1, 1.25, 2),
 annotation = c("*","***", "**"),
 tip_length = 0, textsize= 25,
 size = 5) +
 theme_bw()


boxplot_Antennae_matingduration_bw +
 theme_bw() + theme(panel.grid.major = element_blank(), panel.grid.minor = element_blank()) +
 theme(axis.text=element_text(size=55, face="bold", colour="black"),
 axis.title=element_text(size=75, face="bold", colour="black"),
 legend.text=element_text(size=55, face="bold", colour="black"),
 legend.title=element_text(size=75, face="bold", colour="black")) +
 theme(axis.line = element_line(size = 5),
 panel.border = element_rect(size = 5)) +
 theme(
 axis.ticks.length = unit(0.5, "cm"),
 axis.ticks = element_line(size = 5) ) +
 scale_x_discrete(labels=c("WT" = "Antennae \nIntact", "AntennaeRemoved" = "Antennae \nRemoved")) +
 geom_text(
 data = Antennae_matingduration_sample_sizes,
 aes(
 x = FocalTreatment,
 y = 0.1,
 label = paste0("n=", Count)
 ),
 position = position_dodge(width = 0.75),
 vjust = -0.5,
 size = 12
 )


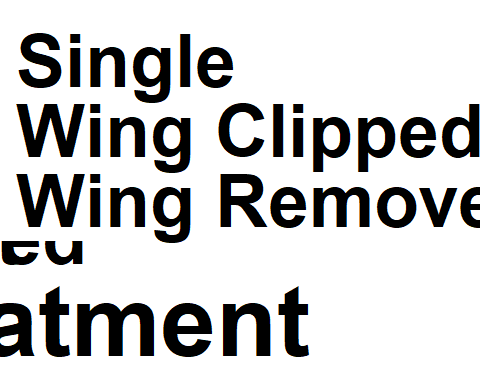


##Data Analysis
#testing data normality
qqnorm(AntennaeEMD_All$MatingDuration)


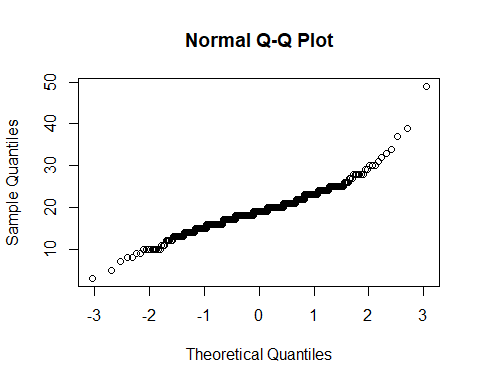


shapiro.test(AntennaeEMD_All$MatingDuration)

Shapiro-Wilk normality test

data: AntennaeEMD_All$MatingDuration
W = 0.94671, p-value = 2.045e-11

#data is not normal

ARmatingduration_glmer1 <- glmer(MatingDuration ~ FocalTreatment * RivalTreatment + (1|DateExperiment), data = AntennaeEMD_All,
 family = poisson(link=log))

boundary (singular) fit: see help('isSingular')

summary(ARmatingduration_glmer1)

Generalized linear mixed model fit by maximum likelihood (Laplace
 Approximation) [glmerMod]
 Family: poisson ( log )
Formula:
MatingDuration ~ FocalTreatment * RivalTreatment + (1 | DateExperiment)
 Data: AntennaeEMD_All

 AIC BIC logLik deviance df.resid
 2557.5 2586.1 -1271.8 2543.5 429

Scaled residuals:
 Min 1Q Median 3Q Max
-3.7438 -0.5969 -0.0335 0.5472 6.9501

Random effects:
 Groups Name Variance Std.Dev.
 DateExperiment (Intercept) 0 0
Number of obs: 436, groups: DateExperiment, 3

Fixed effects:
 Estimate Std. Error
(Intercept) 2.81900 0.02821
FocalTreatmentAntennaeRemoved 0.11678 0.04082
RivalTreatmentWingClip 0.15426 0.03777
RivalTreatmentWingRemoved 0.15886 0.03795
FocalTreatmentAntennaeRemoved:RivalTreatmentWingClip -0.03531 0.05498
FocalTreatmentAntennaeRemoved:RivalTreatmentWingRemoved -0.14250 0.05483
 z value Pr(>|z|)
(Intercept) 99.945 < 2e-16 ***
FocalTreatmentAntennaeRemoved 2.861 0.00422 **
RivalTreatmentWingClip 4.084 4.43e-05 ***
RivalTreatmentWingRemoved 4.186 2.83e-05 ***
FocalTreatmentAntennaeRemoved:RivalTreatmentWingClip -0.642 0.52068
FocalTreatmentAntennaeRemoved:RivalTreatmentWingRemoved -2.599 0.00935 **
---
Signif. codes: 0 '***' 0.001 '**' 0.01 '*' 0.05 '.' 0.1 ' ' 1

Correlation of Fixed Effects:
 (Intr) FclTAR RvlTWC RvlTWR FTAR:RTWC
FclTrtmntAR -0.691
RvlTrtmntWC -0.747 0.516
RvlTrtmntWR -0.743 0.514 0.555
FclTAR:RTWC 0.513 -0.742 -0.687 -0.381
FclTAR:RTWR 0.514 -0.744 -0.384 -0.692 0.553
optimizer (Nelder_Mead) convergence code: 0 (OK)
boundary (singular) fit: see help('isSingular')

ARmatingduration_glmer2 <- glmer(MatingDuration ~ FocalTreatment + RivalTreatment + (1|DateExperiment), data = AntennaeEMD_All,
 family = poisson(link=log))

boundary (singular) fit: see help('isSingular')

anova(ARmatingduration_glmer1, ARmatingduration_glmer2, test = "Chi") #no difference between + and * model

Data: AntennaeEMD_All
Models:
ARmatingduration_glmer2: MatingDuration ~ FocalTreatment + RivalTreatment + (1 | DateExperiment)
ARmatingduration_glmer1: MatingDuration ~ FocalTreatment * RivalTreatment + (1 | DateExperiment)
 npar AIC BIC logLik deviance Chisq Df
ARmatingduration_glmer2 5 2561.2 2581.6 -1275.6 2551.2
ARmatingduration_glmer1 7 2557.6 2586.1 -1271.8 2543.6 7.6619 2
 Pr(>Chisq)
ARmatingduration_glmer2
ARmatingduration_glmer1 0.02169 *
---
Signif. codes: 0 '***' 0.001 '**' 0.01 '*' 0.05 '.' 0.1 ' ' 1

ARmatingduration_glmer3 <- glmer(MatingDuration ~ FocalTreatment + (1|DateExperiment), data = AntennaeEMD_All,
 family = poisson(link=log))

boundary (singular) fit: see help('isSingular')

anova(ARmatingduration_glmer2, ARmatingduration_glmer3, test = "Chi") #difference when Rival Treatment is removed

Data: AntennaeEMD_All
Models:
ARmatingduration_glmer3: MatingDuration ~ FocalTreatment + (1 | DateExperiment)
ARmatingduration_glmer2: MatingDuration ~ FocalTreatment + RivalTreatment + (1 | DateExperiment)
 npar AIC BIC logLik deviance Chisq Df
ARmatingduration_glmer3 3 2582.9 2595.2 -1288.5 2576.9
ARmatingduration_glmer2 5 2561.2 2581.6 -1275.6 2551.2 25.729 2
 Pr(>Chisq)
ARmatingduration_glmer3
ARmatingduration_glmer2 2.588e-06 ***
---
Signif. codes: 0 '***' 0.001 '**' 0.01 '*' 0.05 '.' 0.1 ' ' 1

ARmatingduration_glmer4 <- glmer(MatingDuration ~ RivalTreatment + (1|DateExperiment), data = AntennaeEMD_All,
 family = poisson(link=log))

boundary (singular) fit: see help('isSingular')

anova(ARmatingduration_glmer2, ARmatingduration_glmer4, test = "Chi") #difference when Focal Treatment is removed

Data: AntennaeEMD_All
Models:
ARmatingduration_glmer4: MatingDuration ~ RivalTreatment + (1 | DateExperiment)
ARmatingduration_glmer2: MatingDuration ~ FocalTreatment + RivalTreatment + (1 | DateExperiment)
 npar AIC BIC logLik deviance Chisq Df
ARmatingduration_glmer4 4 2565.1 2581.4 -1278.5 2557.1
ARmatingduration_glmer2 5 2561.2 2581.6 -1275.6 2551.2 5.8813 1
 Pr(>Chisq)
ARmatingduration_glmer4
ARmatingduration_glmer2 0.0153 *
---
Signif. codes: 0 '***' 0.001 '**' 0.01 '*' 0.05 '.' 0.1 ' ' 1

ARmatingduration_glmer_null <- glmer(MatingDuration ~ (1|DateExperiment), data = AntennaeEMD_All)

Warning in glmer(MatingDuration ~ (1 | DateExperiment), data =
AntennaeEMD_All): calling glmer() with family=gaussian (identity link) as a
shortcut to lmer() is deprecated; please call lmer() directly

boundary (singular) fit: see help('isSingular')

anova(ARmatingduration_glmer2, ARmatingduration_glmer_null, test = "Chi")

refitting model(s) with ML (instead of REML)

Data: AntennaeEMD_All
Models:
ARmatingduration_glmer_null: MatingDuration ~ (1 | DateExperiment)
ARmatingduration_glmer2: MatingDuration ~ FocalTreatment + RivalTreatment + (1 | DateExperiment)
 npar AIC BIC logLik deviance Chisq Df
ARmatingduration_glmer_null 3 2596.6 2608.9 -1295.3 2590.6
ARmatingduration_glmer2 5 2561.2 2581.6 -1275.6 2551.2 39.436 2
 Pr(>Chisq)
ARmatingduration_glmer_null
ARmatingduration_glmer2 2.733e-09 ***
---
Signif. codes: 0 '***' 0.001 '**' 0.01 '*' 0.05 '.' 0.1 ' ' 1

#post hoc tests
lsmeans(ARmatingduration_glmer1, pairwise ~ FocalTreatment * RivalTreatment, adjust="tukey")

$lsmeans
 FocalTreatment RivalTreatment lsmean SE df asymp.LCL asymp.UCL
 WT Single 2.82 0.0282 Inf 2.76 2.87
 AntennaeRemoved Single 2.94 0.0295 Inf 2.88 2.99
 WT WingClip 2.97 0.0251 Inf 2.92 3.02
 AntennaeRemoved WingClip 3.05 0.0269 Inf 3.00 3.11
 WT WingRemoved 2.98 0.0254 Inf 2.93 3.03
 AntennaeRemoved WingRemoved 2.95 0.0264 Inf 2.90 3.00

Results are given on the log (not the response) scale.
Confidence level used: 0.95

$contrasts
 contrast estimate SE df
 WT Single - AntennaeRemoved Single -0.11678 0.0408 Inf
 WT Single - WT WingClip -0.15426 0.0378 Inf
 WT Single - AntennaeRemoved WingClip -0.23573 0.0390 Inf
 WT Single - WT WingRemoved -0.15886 0.0379 Inf
 WT Single - AntennaeRemoved WingRemoved -0.13313 0.0386 Inf
 AntennaeRemoved Single - WT WingClip -0.03749 0.0388 Inf
 AntennaeRemoved Single - AntennaeRemoved WingClip -0.11895 0.0399 Inf
 AntennaeRemoved Single - WT WingRemoved -0.04208 0.0389 Inf
 AntennaeRemoved Single - AntennaeRemoved WingRemoved -0.01636 0.0396 Inf
 WT WingClip - AntennaeRemoved WingClip -0.08147 0.0368 Inf
 WT WingClip - WT WingRemoved -0.00459 0.0357 Inf
 WT WingClip - AntennaeRemoved WingRemoved 0.02113 0.0364 Inf
 AntennaeRemoved WingClip - WT WingRemoved 0.07687 0.0370 Inf
 AntennaeRemoved WingClip - AntennaeRemoved WingRemoved 0.10260 0.0377 Inf
 WT WingRemoved - AntennaeRemoved WingRemoved 0.02572 0.0366 Inf
 z.ratio p.value
 -2.861 0.0484
 -4.084 0.0006
 -6.045 <.0001
 -4.186 0.0004
 -3.447 0.0075
 -0.967 0.9283
 -2.978 0.0345
 -1.081 0.8891
 -0.413 0.9985
 -2.212 0.2321
 -0.129 1.0000
 0.580 0.9924
 2.077 0.2992
 2.721 0.0711
 0.703 0.9817

Results are given on the log (not the response) scale.
P value adjustment: tukey method for comparing a family of 6 estimates

#effect sizes
AntennaeRemoved_group_stats_EMD <- AntennaeEMD_All %>%
 group_by(FocalTreatment, RivalTreatment) %>%
 dplyr::summarise(mean = mean(MatingDuration),
 sd = sd(MatingDuration),
 N = n())

`summarise()` has grouped output by 'FocalTreatment'. You can override using
the `.groups` argument.

AntennaeRemovedExpEMD_WT_Single <- AntennaeEMD_All %>%
 filter(RivalTreatment == "Single", FocalTreatment == "WT") %>%
 select(MatingDuration)
AntennaeRemovedExpEMD_WT_WC <- AntennaeEMD_All %>%
 filter(RivalTreatment == "WingClip", FocalTreatment == "WT") %>%
 select(MatingDuration)
AntennaeRemovedExpEMD_WT_WR <- AntennaeEMD_All %>%
 filter(RivalTreatment == "WingRemoved", FocalTreatment == "WT") %>%
 select(MatingDuration)

cohen.d(AntennaeRemovedExpEMD_WT_Single$MatingDuration, AntennaeRemovedExpEMD_WT_WC$MatingDuration)

Cohen's d

d estimate: -0.6765144 (medium)
95 percent confidence interval:
 lower upper
-1.0019961 -0.3510327

cohen.d(AntennaeRemovedExpEMD_WT_Single$MatingDuration, AntennaeRemovedExpEMD_WT_WR$MatingDuration)

Cohen's d

d estimate: -0.6687732 (medium)
95 percent confidence interval:
 lower upper
-0.9960694 -0.3414770

cohen.d(AntennaeRemovedExpEMD_WT_WR$MatingDuration, AntennaeRemovedExpEMD_WT_WC$MatingDuration)

Cohen's d

d estimate: 0.01870225 (negligible)
95 percent confidence interval:
 lower upper
-0.2936185 0.3310230

AntennaeRemovedExpEMD_AntennaeRemoved_Single <- AntennaeEMD_All %>%
 filter(RivalTreatment == "Single", FocalTreatment == "AntennaeRemoved") %>%
 select(MatingDuration)
AntennaeRemovedExpEMD_AntennaeRemoved_WC <- AntennaeEMD_All %>%
 filter(RivalTreatment == "WingClip", FocalTreatment == "AntennaeRemoved") %>%
 select(MatingDuration)
AntennaeRemovedExpEMD_AntennaeRemoved_WR <- AntennaeEMD_All %>%
 filter(RivalTreatment == "WingRemoved", FocalTreatment == "AntennaeRemoved") %>%
 select(MatingDuration)

cohen.d(AntennaeRemovedExpEMD_AntennaeRemoved_Single$MatingDuration, AntennaeRemovedExpEMD_AntennaeRemoved_WC$MatingDuration)

Cohen's d

d estimate: -0.4623866 (small)
95 percent confidence interval:
 lower upper
-0.8199000 -0.1048732

cohen.d(AntennaeRemovedExpEMD_AntennaeRemoved_Single$MatingDuration, AntennaeRemovedExpEMD_AntennaeRemoved_WR$MatingDuration)

Cohen's d

d estimate: -0.06519193 (negligible)
95 percent confidence interval:
 lower upper
-0.4062875 0.2759036

cohen.d(AntennaeRemovedExpEMD_AntennaeRemoved_WR$MatingDuration, AntennaeRemovedExpEMD_AntennaeRemoved_WC$MatingDuration)

Cohen's d

d estimate: -0.4860026 (small)
95 percent confidence interval:
 lower upper
-0.8259695 -0.1460356

## Lifespan

**Figure 2B**

**Description of Data:** Lifespan assay comparing effects of social treatment and senses of WT and Antennae Removed Flies flies when kept solo versus when they were with a rival having a clipped wing or a completely removed wing.

**Factors:**

- ID-Unique Fly ID
- Number-Unique Fly ID (this experiment)
- Focal_Treatment-Manipulation to the focal fly. (WT=not manipulated, AntennaeRemoved=antennae surgically removed 24h post eclosion)
- Rival_Treatment-Social Treatment. Single=focal fly kept in isolation post eclosion, WingClip=fly kept with single rival with a wing clip from eclosion to mating, WingRemoved=fly kept with a rival with wings surgical removed
- Treatment-Interaction between Focal and Rival Treatments
- Date_Set_Up-Day experiment started
- Date_Death-Day fly finished experiment (died or lost)
- Censoring_Status-Censoring Status (1=fly died naturally, 0=fly didn't die naturally (e.g. lost on transfer)
- Lifespan-Lifespan in days
- Number_Rival_Changes-Number of times rival fly replaced
- Rival_Changed?-Date of rival changes

###loading packages
library(ggplot2)
library(ggpubr)
library(survival)
library(survminer)
library(dplyr)
library(emmeans)
library(lme4)
library(effsize)

###importing data
AR_lifespan1 <- read.csv("AntennaeRemovalExperimentResults.csv", header = TRUE)
AR_lifespan2 <- read.csv("AntennaeRemovalRepeatLifespan.csv", header = TRUE)
AR_lifespan <- rbind(AR_lifespan1, AR_lifespan2)

##Survival Curve
surv_object_AR <- Surv(time=AR_lifespan$Lifespan, event=AR_lifespan$Censoring_Status)
fit_AR <- survfit(surv_object_AR ~ Treatment, data = AR_lifespan)
summary(fit_AR)

Call: survfit(formula = surv_object_AR ~ Treatment, data = AR_lifespan)

7 observations deleted due to missingness
 Treatment=AntennaeRemoved Single
 time n.risk n.event survival std.err lower 95% CI upper 95% CI
 14 97 1 0.9897 0.0103 0.96979 1.0000
 23 95 2 0.9689 0.0177 0.93478 1.0000
 25 93 1 0.9584 0.0203 0.91938 0.9992
 26 92 1 0.9480 0.0226 0.90468 0.9934
 27 91 1 0.9376 0.0247 0.89048 0.9872
 32 90 2 0.9168 0.0282 0.86317 0.9737
 33 88 1 0.9063 0.0297 0.84993 0.9665
 36 87 1 0.8959 0.0312 0.83691 0.9591
 37 86 2 0.8751 0.0337 0.81142 0.9438
 38 84 1 0.8647 0.0349 0.79891 0.9359
 41 83 1 0.8543 0.0360 0.78653 0.9278
 45 82 2 0.8334 0.0380 0.76214 0.9114
 46 80 2 0.8126 0.0398 0.73817 0.8945
 47 78 2 0.7918 0.0414 0.71456 0.8773
 48 76 2 0.7709 0.0429 0.69128 0.8597
 50 74 1 0.7605 0.0436 0.67975 0.8508
 51 73 2 0.7397 0.0448 0.65690 0.8329
 54 71 1 0.7292 0.0453 0.64557 0.8238
 55 70 5 0.6772 0.0477 0.58980 0.7774
 57 65 2 0.6563 0.0485 0.56788 0.7585
 58 63 1 0.6459 0.0488 0.55699 0.7490
 60 62 1 0.6355 0.0491 0.54615 0.7394
 61 61 3 0.6042 0.0499 0.51392 0.7104
 62 58 2 0.5834 0.0503 0.49267 0.6908
 65 56 7 0.5105 0.0510 0.41966 0.6209
 69 48 5 0.4573 0.0509 0.36759 0.5689
 71 43 2 0.4360 0.0507 0.34709 0.5478
 72 41 3 0.4041 0.0503 0.31669 0.5157
 73 38 2 0.3829 0.0498 0.29667 0.4941
 74 36 2 0.3616 0.0493 0.27684 0.4723
 75 34 3 0.3297 0.0482 0.24748 0.4392
 76 31 2 0.3084 0.0474 0.22817 0.4169
 77 29 4 0.2659 0.0454 0.19025 0.3715
 78 25 1 0.2552 0.0448 0.18093 0.3601
 79 24 2 0.2340 0.0435 0.16248 0.3369
 80 22 2 0.2127 0.0421 0.14432 0.3135
 81 19 1 0.2015 0.0413 0.13479 0.3012
 83 18 1 0.1903 0.0405 0.12537 0.2889
 84 17 2 0.1679 0.0387 0.10685 0.2639
 85 15 1 0.1567 0.0377 0.09777 0.2512
 87 14 2 0.1343 0.0355 0.08002 0.2255
 88 12 2 0.1119 0.0329 0.06290 0.1992
 89 10 1 0.1008 0.0315 0.05461 0.1859
 94 9 2 0.0784 0.0282 0.03872 0.1586
 97 7 1 0.0672 0.0263 0.03119 0.1446
 100 6 1 0.0560 0.0242 0.02401 0.1305
 101 5 1 0.0448 0.0218 0.01726 0.1162
 104 4 1 0.0336 0.0190 0.01108 0.1017
 105 3 1 0.0224 0.0156 0.00571 0.0879
 108 2 2 0.0000 NaN NA NA

 Treatment=AntennaeRemoved WingClip
 time n.risk n.event survival std.err lower 95% CI upper 95% CI
 2 99 1 0.9899 0.0100 0.97040 1.0000
 3 98 6 0.9293 0.0258 0.88015 0.9812
 5 92 2 0.9091 0.0289 0.85419 0.9675
 7 90 1 0.8990 0.0303 0.84155 0.9604
 9 89 1 0.8889 0.0316 0.82909 0.9530
 10 88 3 0.8586 0.0350 0.79262 0.9300
 11 85 2 0.8384 0.0370 0.76892 0.9141
 14 82 1 0.8282 0.0379 0.75706 0.9059
 16 81 1 0.8179 0.0388 0.74529 0.8977
 17 80 1 0.8077 0.0397 0.73361 0.8893
 21 79 2 0.7873 0.0412 0.71051 0.8723
 26 77 1 0.7770 0.0419 0.69908 0.8637
 27 76 1 0.7668 0.0426 0.68772 0.8550
 33 75 1 0.7566 0.0432 0.67642 0.8463
 39 74 1 0.7464 0.0438 0.66520 0.8374
 44 73 1 0.7361 0.0444 0.65403 0.8286
 45 72 1 0.7259 0.0450 0.64293 0.8196
 47 71 3 0.6952 0.0464 0.60997 0.7924
 51 68 3 0.6646 0.0476 0.57748 0.7648
 52 65 1 0.6543 0.0480 0.56675 0.7555
 53 64 1 0.6441 0.0483 0.55607 0.7461
 54 63 1 0.6339 0.0486 0.54544 0.7367
 57 62 1 0.6237 0.0489 0.53485 0.7273
 58 61 1 0.6135 0.0491 0.52431 0.7178
 59 60 1 0.6032 0.0494 0.51381 0.7082
 60 59 4 0.5623 0.0501 0.47225 0.6696
 61 55 3 0.5317 0.0504 0.44153 0.6402
 63 51 3 0.5004 0.0506 0.41049 0.6100
 66 48 2 0.4795 0.0506 0.39002 0.5896
 67 46 1 0.4691 0.0505 0.37985 0.5793
 68 45 2 0.4483 0.0504 0.35964 0.5587
 69 43 2 0.4274 0.0501 0.33961 0.5379
 70 40 3 0.3954 0.0497 0.30904 0.5058
 71 37 1 0.3847 0.0495 0.29896 0.4950
 72 36 4 0.3419 0.0484 0.25913 0.4512
 73 32 3 0.3099 0.0472 0.22983 0.4178
 74 29 2 0.2885 0.0463 0.21058 0.3953
 75 27 1 0.2778 0.0458 0.20105 0.3839
 76 26 2 0.2564 0.0447 0.18218 0.3610
 77 24 2 0.2351 0.0435 0.16359 0.3378
 78 22 1 0.2244 0.0428 0.15440 0.3261
 79 21 2 0.2030 0.0413 0.13626 0.3025
 80 19 1 0.1923 0.0405 0.12731 0.2906
 81 18 2 0.1710 0.0387 0.10969 0.2665
 82 16 1 0.1603 0.0377 0.10103 0.2543
 83 15 3 0.1282 0.0344 0.07575 0.2170
 84 12 2 0.1069 0.0318 0.05959 0.1916
 85 10 1 0.0962 0.0304 0.05176 0.1787
 87 9 1 0.0855 0.0288 0.04413 0.1656
 88 8 1 0.0748 0.0271 0.03673 0.1523
 89 7 2 0.0534 0.0232 0.02280 0.1252
 92 5 2 0.0321 0.0182 0.01054 0.0975
 94 3 1 0.0214 0.0149 0.00543 0.0841
 98 2 2 0.0000 NaN NA NA

 Treatment=AntennaeRemoved WingRemoved
 time n.risk n.event survival std.err lower 95% CI upper 95% CI
 1 100 1 0.9900 0.00995 0.97069 1.0000
 2 99 3 0.9600 0.01960 0.92235 0.9992
 5 96 1 0.9500 0.02179 0.90823 0.9937
 6 95 1 0.9400 0.02375 0.89459 0.9877
 7 94 3 0.9100 0.02862 0.85560 0.9679
 8 91 1 0.9000 0.03000 0.84308 0.9608
 11 90 1 0.8900 0.03129 0.83074 0.9535
 14 89 1 0.8800 0.03250 0.81856 0.9461
 21 88 1 0.8700 0.03363 0.80652 0.9385
 30 87 1 0.8600 0.03470 0.79461 0.9308
 32 86 1 0.8500 0.03571 0.78282 0.9229
 33 85 1 0.8400 0.03666 0.77113 0.9150
 34 84 2 0.8200 0.03842 0.74805 0.8989
 35 82 2 0.8000 0.04000 0.72532 0.8824
 36 80 1 0.7900 0.04073 0.71407 0.8740
 37 79 3 0.7600 0.04271 0.68074 0.8485
 39 76 1 0.7500 0.04330 0.66976 0.8399
 40 75 1 0.7400 0.04386 0.65884 0.8312
 44 74 1 0.7300 0.04440 0.64797 0.8224
 45 73 1 0.7200 0.04490 0.63716 0.8136
 46 72 2 0.7000 0.04583 0.61571 0.7958
 47 70 1 0.6900 0.04625 0.60505 0.7869
 51 69 2 0.6700 0.04702 0.58390 0.7688
 52 67 2 0.6500 0.04770 0.56293 0.7505
 54 65 2 0.6300 0.04828 0.54214 0.7321
 55 63 1 0.6200 0.04854 0.53181 0.7228
 56 62 1 0.6100 0.04877 0.52152 0.7135
 58 61 2 0.5900 0.04918 0.50107 0.6947
 60 59 1 0.5800 0.04936 0.49090 0.6853
 61 58 4 0.5400 0.04984 0.45064 0.6471
 62 54 2 0.5200 0.04996 0.43075 0.6277
 63 52 3 0.4900 0.04999 0.40120 0.5985
 65 49 3 0.4600 0.04984 0.37199 0.5688
 66 46 3 0.4300 0.04951 0.34314 0.5389
 67 43 1 0.4200 0.04936 0.33360 0.5288
 68 41 2 0.3995 0.04903 0.31410 0.5081
 69 39 1 0.3893 0.04883 0.30442 0.4978
 70 38 2 0.3688 0.04836 0.28520 0.4769
 71 36 3 0.3380 0.04747 0.25671 0.4452
 72 33 2 0.3176 0.04676 0.23796 0.4238
 74 31 2 0.2971 0.04593 0.21941 0.4022
 75 29 1 0.2868 0.04547 0.21022 0.3914
 76 28 3 0.2561 0.04393 0.18298 0.3584
 77 25 1 0.2459 0.04335 0.17402 0.3473
 78 23 4 0.2031 0.04074 0.13707 0.3009
 79 19 1 0.1924 0.03997 0.12805 0.2891
 81 18 1 0.1817 0.03916 0.11912 0.2772
 84 17 3 0.1497 0.03636 0.09295 0.2409
 85 14 2 0.1283 0.03416 0.07611 0.2162
 86 12 1 0.1176 0.03295 0.06789 0.2036
 87 11 3 0.0855 0.02870 0.04430 0.1651
 89 8 1 0.0748 0.02703 0.03686 0.1519
 90 7 3 0.0428 0.02084 0.01645 0.1112
 92 4 1 0.0321 0.01817 0.01056 0.0973
 93 3 1 0.0214 0.01493 0.00544 0.0840
 95 2 2 0.0000 NaN NA NA

 Treatment=WT Single
 time n.risk n.event survival std.err lower 95% CI upper 95% CI
 33 98 1 0.9898 0.0102 0.97010 1.0000
 34 97 2 0.9694 0.0174 0.93587 1.0000
 42 95 1 0.9592 0.0200 0.92080 0.9992
 45 94 1 0.9490 0.0222 0.90640 0.9936
 47 93 3 0.9184 0.0277 0.86573 0.9742
 53 90 1 0.9082 0.0292 0.85275 0.9672
 54 89 2 0.8878 0.0319 0.82741 0.9525
 55 86 2 0.8671 0.0343 0.80238 0.9371
 56 84 1 0.8568 0.0354 0.79008 0.9291
 57 83 1 0.8465 0.0365 0.77790 0.9211
 58 82 4 0.8052 0.0401 0.73026 0.8878
 60 78 1 0.7949 0.0409 0.71858 0.8792
 61 77 2 0.7742 0.0424 0.69545 0.8619
 62 75 2 0.7536 0.0437 0.67262 0.8442
 63 73 4 0.7123 0.0459 0.62773 0.8082
 64 69 2 0.6916 0.0468 0.60564 0.7898
 65 67 1 0.6813 0.0473 0.59468 0.7805
 67 66 1 0.6710 0.0477 0.58376 0.7712
 68 65 2 0.6503 0.0484 0.56209 0.7524
 69 63 3 0.6194 0.0493 0.52994 0.7239
 71 60 1 0.6090 0.0495 0.51932 0.7143
 72 59 3 0.5781 0.0501 0.48772 0.6852
 74 56 1 0.5678 0.0503 0.47728 0.6754
 75 55 3 0.5368 0.0506 0.44620 0.6458
 76 52 1 0.5265 0.0507 0.43592 0.6358
 77 51 1 0.5161 0.0507 0.42569 0.6258
 78 50 1 0.5058 0.0508 0.41550 0.6158
 80 49 4 0.4645 0.0506 0.37516 0.5752
 81 45 3 0.4336 0.0503 0.34534 0.5443
 82 42 4 0.3923 0.0496 0.30618 0.5025
 83 38 1 0.3819 0.0493 0.29651 0.4920
 84 37 2 0.3613 0.0488 0.27728 0.4708
 85 35 3 0.3303 0.0478 0.24880 0.4386
 86 32 2 0.3097 0.0470 0.23006 0.4169
 87 30 3 0.2787 0.0455 0.20234 0.3839
 88 27 3 0.2477 0.0439 0.17512 0.3505
 89 24 2 0.2271 0.0426 0.15729 0.3279
 90 22 2 0.2065 0.0411 0.13973 0.3050
 91 20 1 0.1961 0.0403 0.13107 0.2935
 94 19 3 0.1652 0.0377 0.10556 0.2584
 95 16 2 0.1445 0.0357 0.08903 0.2346
 97 14 1 0.1342 0.0346 0.08093 0.2225
 98 13 3 0.1032 0.0309 0.05740 0.1856
 100 10 1 0.0929 0.0295 0.04987 0.1731
 101 9 1 0.0826 0.0280 0.04253 0.1604
 102 7 1 0.0708 0.0263 0.03413 0.1468
 103 6 1 0.0590 0.0244 0.02618 0.1329
 104 5 1 0.0472 0.0222 0.01875 0.1188
 105 4 1 0.0354 0.0196 0.01199 0.1045
 107 3 1 0.0236 0.0162 0.00614 0.0907
 108 2 1 0.0118 0.0116 0.00171 0.0815
 116 1 1 0.0000 NaN NA NA

 Treatment=WT WingClip
 time n.risk n.event survival std.err lower 95% CI upper 95% CI
 4 98 1 0.9898 0.0102 0.97010 1.0000
 7 97 1 0.9796 0.0143 0.95199 1.0000
 12 96 1 0.9694 0.0174 0.93587 1.0000
 23 93 1 0.9590 0.0201 0.92038 0.9992
 32 91 1 0.9484 0.0225 0.90540 0.9935
 47 90 1 0.9379 0.0246 0.89096 0.9873
 48 89 1 0.9274 0.0265 0.87692 0.9807
 50 88 1 0.9168 0.0282 0.86322 0.9737
 51 87 3 0.8852 0.0326 0.82359 0.9514
 54 84 1 0.8747 0.0339 0.81076 0.9436
 55 83 1 0.8641 0.0350 0.79808 0.9356
 58 82 1 0.8536 0.0362 0.78555 0.9275
 60 80 1 0.8429 0.0373 0.77296 0.9192
 61 79 3 0.8109 0.0402 0.73588 0.8936
 63 76 2 0.7896 0.0418 0.71166 0.8760
 64 74 1 0.7789 0.0426 0.69968 0.8671
 65 73 5 0.7255 0.0459 0.64094 0.8213
 66 68 1 0.7149 0.0464 0.62940 0.8120
 67 67 1 0.7042 0.0470 0.61792 0.8025
 68 66 2 0.6829 0.0479 0.59515 0.7835
 69 64 4 0.6402 0.0494 0.55028 0.7448
 70 60 2 0.6188 0.0500 0.52816 0.7251
 71 58 3 0.5868 0.0507 0.49535 0.6952
 72 55 1 0.5762 0.0509 0.48451 0.6852
 73 54 2 0.5548 0.0512 0.46298 0.6649
 74 52 1 0.5442 0.0513 0.45228 0.6547
 75 51 3 0.5122 0.0515 0.42046 0.6238
 77 48 8 0.4268 0.0510 0.33763 0.5395
 78 40 2 0.4055 0.0507 0.31738 0.5180
 79 38 4 0.3628 0.0496 0.27747 0.4743
 80 34 3 0.3308 0.0486 0.24806 0.4410
 81 31 3 0.2988 0.0472 0.21913 0.4073
 82 28 2 0.2774 0.0462 0.20013 0.3845
 83 26 1 0.2667 0.0457 0.19072 0.3731
 84 25 5 0.2134 0.0423 0.14469 0.3147
 85 20 3 0.1814 0.0398 0.11800 0.2788
 86 17 3 0.1494 0.0368 0.09216 0.2421
 87 14 2 0.1280 0.0345 0.07550 0.2171
 88 12 1 0.1174 0.0332 0.06737 0.2045
 89 11 1 0.1067 0.0319 0.05940 0.1917
 90 10 3 0.0747 0.0272 0.03663 0.1523
 91 7 1 0.0640 0.0253 0.02952 0.1388
 92 6 1 0.0533 0.0232 0.02274 0.1252
 93 5 3 0.0213 0.0149 0.00542 0.0841
 94 2 2 0.0000 NaN NA NA

 Treatment=WT WingRemoved
 time n.risk n.event survival std.err lower 95% CI upper 95% CI
 1 99 1 0.9899 0.0100 0.9704 1.000
 5 97 1 0.9797 0.0142 0.9522 1.000
 13 96 1 0.9695 0.0173 0.9361 1.000
 21 95 1 0.9593 0.0199 0.9210 0.999
 28 94 1 0.9491 0.0222 0.9066 0.994
 32 93 1 0.9389 0.0242 0.8927 0.987
 41 92 1 0.9287 0.0260 0.8791 0.981
 45 91 1 0.9185 0.0276 0.8659 0.974
 49 90 1 0.9083 0.0291 0.8529 0.967
 50 89 1 0.8981 0.0306 0.8401 0.960
 51 88 1 0.8878 0.0319 0.8275 0.953
 52 86 1 0.8775 0.0331 0.8149 0.945
 53 85 1 0.8672 0.0343 0.8025 0.937
 54 84 2 0.8466 0.0365 0.7780 0.921
 55 82 1 0.8362 0.0375 0.7660 0.913
 56 81 2 0.8156 0.0393 0.7421 0.896
 57 79 1 0.8053 0.0401 0.7304 0.888
 58 78 1 0.7949 0.0409 0.7187 0.879
 60 77 2 0.7743 0.0424 0.6956 0.862
 61 75 1 0.7640 0.0430 0.6841 0.853
 62 74 1 0.7536 0.0437 0.6727 0.844
 63 73 3 0.7227 0.0454 0.6390 0.817
 64 69 1 0.7122 0.0459 0.6276 0.808
 65 68 4 0.6703 0.0478 0.5829 0.771
 66 64 2 0.6494 0.0485 0.5609 0.752
 67 62 2 0.6284 0.0492 0.5391 0.733
 68 60 2 0.6075 0.0497 0.5175 0.713
 69 58 5 0.5551 0.0506 0.4642 0.664
 70 53 2 0.5341 0.0508 0.4432 0.644
 71 51 1 0.5237 0.0509 0.4328 0.634
 72 50 2 0.5027 0.0510 0.4121 0.613
 74 48 1 0.4923 0.0510 0.4018 0.603
 76 47 2 0.4713 0.0509 0.3814 0.582
 77 45 2 0.4504 0.0508 0.3611 0.562
 79 43 3 0.4189 0.0504 0.3310 0.530
 81 40 2 0.3980 0.0500 0.3112 0.509
 82 38 1 0.3875 0.0497 0.3013 0.498
 83 37 8 0.3037 0.0470 0.2243 0.411
 84 29 3 0.2723 0.0455 0.1963 0.378
 85 26 2 0.2514 0.0443 0.1779 0.355
 86 24 2 0.2304 0.0430 0.1598 0.332
 87 22 2 0.2095 0.0416 0.1419 0.309
 88 20 3 0.1780 0.0391 0.1157 0.274
 89 17 3 0.1466 0.0362 0.0904 0.238
 90 13 1 0.1353 0.0351 0.0814 0.225
 91 12 2 0.1128 0.0327 0.0639 0.199
 92 10 3 0.0790 0.0281 0.0393 0.159
 93 7 2 0.0564 0.0242 0.0243 0.131
 95 5 1 0.0451 0.0218 0.0175 0.116
 98 4 1 0.0338 0.0191 0.0112 0.102

survcurv_AR <- ggsurvplot(fit_AR, data = AR_lifespan,
 linetype = c("solid", "twodash", "dotted", "solid", "twodash", "dotted"),
 legend.labs = c("Antennae kept Alone", "Antennae kept with Wing Clipped Rival", "Antennae kept with Wing Removed Rival", "Wildtype kept Singly", "Wildtype kept with Wing Clipped Rival", "Wildtype kept with Wing Removed Rival"),
 palette = c("gray73", "gray73", "gray73", "black", "black", "black"),
 pval = FALSE)
survcurv_AR_Figure <- ggpar(survcurv_AR, font.main=c(16, "bold", "black"),
 xlab = "Days Since Eclosion", ylab = "Cumulative Survival",
 legend = "right", legend.title = "",
 xlim = c (0 , 130))

Coordinate system already present. Adding new coordinate system, which will
replace the existing one.

survcurv_AR_Figure


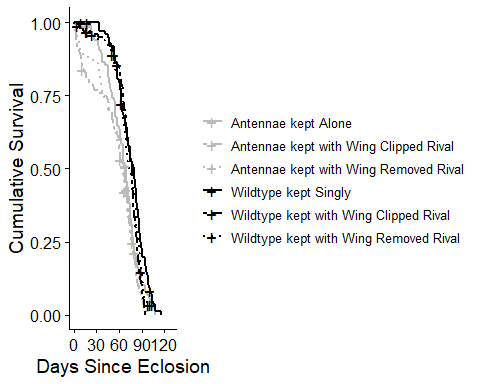


fit.coxph1_AR <- coxph(surv_object_AR ~ Focal_Treatment * Rival_Treatment,
 data = AR_lifespan)
summary(fit.coxph1_AR)

Call:
coxph(formula = surv_object_AR ~ Focal_Treatment * Rival_Treatment,
 data = AR_lifespan)

 n= 593, number of events= 570
 (7 observations deleted due to missingness)

 coef exp(coef) se(coef) z
Focal_TreatmentWT -0.45427 0.63491 0.14601 -3.111
Rival_TreatmentWingClip 0.32707 1.38690 0.14709 2.224
Rival_TreatmentWingRemoved 0.32171 1.37948 0.14663 2.194
Focal_TreatmentWT:Rival_TreatmentWingClip 0.05552 1.05709 0.20587 0.270
Focal_TreatmentWT:Rival_TreatmentWingRemoved -0.07192 0.93060 0.20564 -0.350
 Pr(>|z|)
Focal_TreatmentWT 0.00186 **
Rival_TreatmentWingClip 0.02617 *
Rival_TreatmentWingRemoved 0.02824 *
Focal_TreatmentWT:Rival_TreatmentWingClip 0.78740
Focal_TreatmentWT:Rival_TreatmentWingRemoved 0.72652
---
Signif. codes: 0 '***' 0.001 '**' 0.01 '*' 0.05 '.' 0.1 ' ' 1

 exp(coef) exp(-coef) lower .95
Focal_TreatmentWT 0.6349 1.5750 0.4769
Rival_TreatmentWingClip 1.3869 0.7210 1.0395
Rival_TreatmentWingRemoved 1.3795 0.7249 1.0349
Focal_TreatmentWT:Rival_TreatmentWingClip 1.0571 0.9460 0.7061
Focal_TreatmentWT:Rival_TreatmentWingRemoved 0.9306 1.0746 0.6219
 upper .95
Focal_TreatmentWT 0.8453
Rival_TreatmentWingClip 1.8503
Rival_TreatmentWingRemoved 1.8388
Focal_TreatmentWT:Rival_TreatmentWingClip 1.5825
Focal_TreatmentWT:Rival_TreatmentWingRemoved 1.3925

Concordance= 0.596 (se = 0.014 )
Likelihood ratio test= 42.59 on 5 df, p=4e-08
Wald test = 42.36 on 5 df, p=5e-08
Score (logrank) test = 43.55 on 5 df, p=3e-08

ggforest(fit.coxph1_AR, data = AR_lifespan)


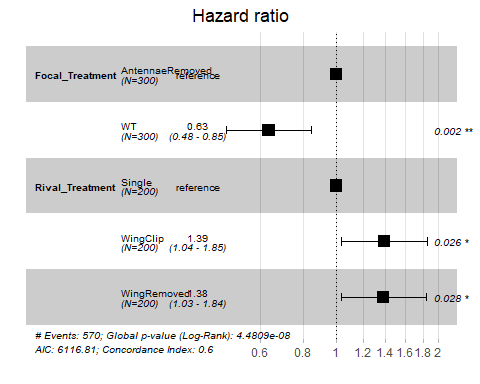


##Boxplot
AR_lifespan_Cens <- subset(AR_lifespan, Censoring_Status == "1")
#boxplot
AR_lifespan_Cens$Treatment <- gsub(" ", "", AR_lifespan_Cens$Treatment)
AR_lifespan_Cens$Rival_Treatment <- gsub(" ", "", AR_lifespan_Cens$Rival_Treatment)

AR_lifespan_Cens$Focal_Treatment <- factor(AR_lifespan_Cens$Focal_Treatment, levels = c("WT", "AntennaeRemoved"))
AR_lifespan_Cens$Rival_Treatment <- factor(AR_lifespan_Cens$Rival_Treatment, levels = c("Single", "WingClip", "WingRemoved"))
AR_lifespan_Cens$Treatment <- factor(AR_lifespan_Cens$Treatment, levels = c("WTSingle", "WTWingClip", "WTWingRemoved", "AntennaeRemovedSingle", "AntennaeRemovedWingClip", "AntennaeRemovedWingRemoved"))

AR_lifespan_sample_sizes <- AR_lifespan_Cens %>%
 group_by(Focal_Treatment, Rival_Treatment, Treatment) %>%
 dplyr::summarise(
 Count = n(),
 Mean_lifespan = mean(Lifespan))

`summarise()` has grouped output by 'Focal_Treatment', 'Rival_Treatment'. You
can override using the `.groups` argument.

AR_lifespan_sample_sizes$Count <- as.character(AR_lifespan_sample_sizes$Count)

boxplot_AR_lifespan_bw <- ggplot(AR_lifespan_Cens) +
 aes(
 x = Focal_Treatment,
 y = Lifespan,
 fill = Rival_Treatment
 ) +
 geom_boxplot(size = 5) +
 scale_fill_manual(
 values = c(Single = "white",
 WingClip = "grey70",
 WingRemoved = "grey25"),
 labels=c("Single" = "Single",
 "WingClip" = "Wing Clipped",
 "WingRemoved" = "Wing Removed")
 ) +
 labs(
 x = "Focal Treatment",
 y = "Days since Eclosion ",
 fill = "Social Treatment"
 ) +
 ylim(-2, 140) +
 geom_signif(y_position = c(125, 135, 125, 135),
 xmin = c(0.75, 0.75, 1.75, 1.75),
 xmax = c(1, 1.25, 2, 2.25), annotation = c("*", "***", "***", "***"),
 tip_length = 0, textsize = 25, size=5)

boxplot_AR_lifespan_bw +
 theme_bw() + theme(panel.grid.major = element_blank(), panel.grid.minor = element_blank()) +
 theme(axis.text=element_text(size=55, face="bold", colour="black"),
 axis.title=element_text(size=75, face="bold", colour="black"),
 legend.text=element_text(size=55, face="bold", colour="black"),
 legend.title=element_text(size=75, face="bold", colour="black")) +
 theme(axis.line = element_line(size = 5),
 panel.border = element_rect(size = 5)) +
 theme(
 axis.ticks.length = unit(0.5, "cm"),
 axis.ticks = element_line(size = 5) ) +
 scale_x_discrete(labels=c("WT" = "Antennae Intact", "AntennaeRemoved" = "Antennae Removed")) + theme(legend.position = "right") +
 geom_text(data = AR_lifespan_sample_sizes,
 aes(
 x = Focal_Treatment,
 y = -1,
 label = paste0("n=", Count)
 ),
 position = position_dodge(width = 0.75),
 vjust = -0.5,
 size = 12)


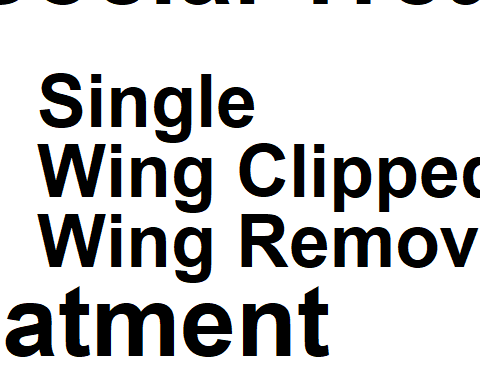


###Linear Models
#check for overdispersion

AR_Lifespan_glmer1 <- glmer(Lifespan ~ Focal_Treatment * Rival_Treatment + (1|Date_Set_Up), data = AR_lifespan_Cens,
 family = poisson(link=log))
AR_Lifespan_glmer_null <- glmer(Lifespan ~ (1|Date_Set_Up), data = AR_lifespan_Cens,
 family = poisson(link=log))
anova(AR_Lifespan_glmer1, AR_Lifespan_glmer_null, test = "Chi")

Data: AR_lifespan_Cens
Models:
AR_Lifespan_glmer_null: Lifespan ~ (1 | Date_Set_Up)
AR_Lifespan_glmer1: Lifespan ~ Focal_Treatment * Rival_Treatment + (1 | Date_Set_Up)
 npar AIC BIC logLik deviance Chisq Df Pr(>Chisq)
AR_Lifespan_glmer_null 2 8588.9 8597.6 -4292.4 8584.9
AR_Lifespan_glmer1 7 8392.3 8422.7 -4189.1 8378.3 206.56 5 < 2.2e-16

AR_Lifespan_glmer_null
AR_Lifespan_glmer1 ***
---
Signif. codes: 0 '***' 0.001 '**' 0.01 '*' 0.05 '.' 0.1 ' ' 1

AR_Lifespan_glmer2 <- glmer(Lifespan ~ Focal_Treatment + Rival_Treatment + (1|Date_Set_Up), data = AR_lifespan_Cens,
 family = poisson(link=log))
anova(AR_Lifespan_glmer1, AR_Lifespan_glmer2, test = "Chi")

Data: AR_lifespan_Cens
Models:
AR_Lifespan_glmer2: Lifespan ~ Focal_Treatment + Rival_Treatment + (1 | Date_Set_Up)
AR_Lifespan_glmer1: Lifespan ~ Focal_Treatment * Rival_Treatment + (1 | Date_Set_Up)
 npar AIC BIC logLik deviance Chisq Df Pr(>Chisq)
AR_Lifespan_glmer2 5 8408.2 8429.9 -4199.1 8398.2
AR_Lifespan_glmer1 7 8392.3 8422.7 -4189.1 8378.3 19.867 2 4.852e-05 ***
---
Signif. codes: 0 '***' 0.001 '**' 0.01 '*' 0.05 '.' 0.1 ' ' 1

AR_Lifespan_glmer3 <- glmer(Lifespan ~ Rival_Treatment + (1|Date_Set_Up), data = AR_lifespan_Cens,
 family = poisson(link=log))
anova(AR_Lifespan_glmer1, AR_Lifespan_glmer3, test = "Chi")

Data: AR_lifespan_Cens
Models:
AR_Lifespan_glmer3: Lifespan ~ Rival_Treatment + (1 | Date_Set_Up)
AR_Lifespan_glmer1: Lifespan ~ Focal_Treatment * Rival_Treatment + (1 | Date_Set_Up)
 npar AIC BIC logLik deviance Chisq Df Pr(>Chisq)
AR_Lifespan_glmer3 4 8504.2 8521.6 -4248.1 8496.2
AR_Lifespan_glmer1 7 8392.3 8422.7 -4189.1 8378.3 117.94 3 < 2.2e-16 ***
---
Signif. codes: 0 '***' 0.001 '**' 0.01 '*' 0.05 '.' 0.1 ' ' 1

AR_Lifespan_glmer4 <- glmer(Lifespan ~ Focal_Treatment + (1|Date_Set_Up), data = AR_lifespan_Cens,
 family = poisson(link=log))
anova(AR_Lifespan_glmer1, AR_Lifespan_glmer4, test = "Chi")

Data: AR_lifespan_Cens
Models:
AR_Lifespan_glmer4: Lifespan ~ Focal_Treatment + (1 | Date_Set_Up)
AR_Lifespan_glmer1: Lifespan ~ Focal_Treatment * Rival_Treatment + (1 | Date_Set_Up)
 npar AIC BIC logLik deviance Chisq Df Pr(>Chisq)
AR_Lifespan_glmer4 3 8490.0 8503.1 -4242.0 8484.0
AR_Lifespan_glmer1 7 8392.3 8422.7 -4189.1 8378.3 105.74 4 < 2.2e-16 ***
---
Signif. codes: 0 '***' 0.001 '**' 0.01 '*' 0.05 '.' 0.1 ' ' 1

AR_Lifespan_glmer_null <- glmer(Lifespan ~ (1|Date_Set_Up), data = AR_lifespan_Cens,
 family = poisson(link=log))
anova(AR_Lifespan_glmer1, AR_Lifespan_glmer_null, test = "F")

Data: AR_lifespan_Cens
Models:
AR_Lifespan_glmer_null: Lifespan ~ (1 | Date_Set_Up)
AR_Lifespan_glmer1: Lifespan ~ Focal_Treatment * Rival_Treatment + (1 | Date_Set_Up)
 npar AIC BIC logLik deviance Chisq Df Pr(>Chisq)
AR_Lifespan_glmer_null 2 8588.9 8597.6 -4292.4 8584.9
AR_Lifespan_glmer1 7 8392.3 8422.7 -4189.1 8378.3 206.56 5 < 2.2e-16

AR_Lifespan_glmer_null
AR_Lifespan_glmer1 ***
---
Signif. codes: 0 '***' 0.001 '**' 0.01 '*' 0.05 '.' 0.1 ' ' 1

#post hoc tests
lsmeans(AR_Lifespan_glmer1, pairwise ~ Focal_Treatment + Rival_Treatment, adjust="tukey")

$lsmeans
 Focal_Treatment Rival_Treatment lsmean SE df asymp.LCL asymp.UCL
 WT Single 4.16 0.0873 Inf 3.99 4.33
 AntennaeRemoved Single 4.09 0.0873 Inf 3.92 4.26
 WT WingClip 4.11 0.0874 Inf 3.94 4.28
 AntennaeRemoved WingClip 3.93 0.0874 Inf 3.75 4.10
 WT WingRemoved 4.08 0.0874 Inf 3.91 4.26
 AntennaeRemoved WingRemoved 3.97 0.0869 Inf 3.80 4.14

Results are given on the log (not the response) scale.
Confidence level used: 0.95

$contrasts
 contrast estimate SE df
 WT Single - AntennaeRemoved Single 0.06972 0.0185 Inf
 WT Single - WT WingClip 0.05423 0.0169 Inf
 WT Single - AntennaeRemoved WingClip 0.23602 0.0191 Inf
 WT Single - WT WingRemoved 0.07649 0.0171 Inf
 WT Single - AntennaeRemoved WingRemoved 0.19027 0.0193 Inf
 AntennaeRemoved Single - WT WingClip -0.01550 0.0188 Inf
 AntennaeRemoved Single - AntennaeRemoved WingClip 0.16629 0.0188 Inf
 AntennaeRemoved Single - WT WingRemoved 0.00677 0.0190 Inf
 AntennaeRemoved Single - AntennaeRemoved WingRemoved 0.12055 0.0191 Inf
 WT WingClip - AntennaeRemoved WingClip 0.18179 0.0194 Inf
 WT WingClip - WT WingRemoved 0.02227 0.0174 Inf
 WT WingClip - AntennaeRemoved WingRemoved 0.13605 0.0196 Inf
 AntennaeRemoved WingClip - WT WingRemoved -0.15952 0.0196 Inf
 AntennaeRemoved WingClip - AntennaeRemoved WingRemoved -0.04574 0.0197 Inf
 WT WingRemoved - AntennaeRemoved WingRemoved 0.11378 0.0198 Inf
 z.ratio p.value
 3.768 0.0023
 3.207 0.0169
 12.377 <.0001
 4.470 0.0001
 9.882 <.0001
 -0.823 0.9634
 8.869 <.0001
 0.356 0.9993
 6.308 <.0001
 9.381 <.0001
 1.278 0.7974
 6.955 <.0001
 -8.147 <.0001
 -2.319 0.1864
 5.757 <.0001

Results are given on the log (not the response) scale.
P value adjustment: tukey method for comparing a family of 6 estimates

lsmeans(AR_Lifespan_glmer1, pairwise ~ Rival_Treatment, adjust="tukey")

NOTE: Results may be misleading due to involvement in interactions

$lsmeans
 Rival_Treatment lsmean SE df asymp.LCL asymp.UCL
 Single 4.13 0.0868 Inf 3.96 4.30
 WingClip 4.02 0.0869 Inf 3.85 4.19
 WingRemoved 4.03 0.0866 Inf 3.86 4.20

Results are averaged over the levels of: Focal_Treatment
Results are given on the log (not the response) scale.
Confidence level used: 0.95

$contrasts
 contrast estimate SE df z.ratio p.value
 Single - WingClip 0.1103 0.0126 Inf 8.734 <.0001
 Single - WingRemoved 0.0985 0.0128 Inf 7.682 <.0001
 WingClip - WingRemoved -0.0117 0.0132 Inf -0.892 0.6454

Results are averaged over the levels of: Focal_Treatment
Results are given on the log (not the response) scale.
P value adjustment: tukey method for comparing a family of 3 estimates

lsmeans(AR_Lifespan_glmer1, pairwise ~ Focal_Treatment, adjust="tukey")

NOTE: Results may be misleading due to involvement in interactions

$lsmeans
 Focal_Treatment lsmean SE df asymp.LCL asymp.UCL
 WT 4.12 0.0868 Inf 3.95 4.29
 AntennaeRemoved 4.00 0.0865 Inf 3.83 4.17

Results are averaged over the levels of: Rival_Treatment
Results are given on the log (not the response) scale.
Confidence level used: 0.95

$contrasts
 contrast estimate SE df z.ratio p.value
 WT - AntennaeRemoved 0.122 0.0122 Inf 9.989 <.0001

Results are averaged over the levels of: Rival_Treatment
Results are given on the log (not the response) scale.

#effect sizes
AntennaeRemoved_group_stats_lifespan <- AR_lifespan_Cens %>%
 group_by(Focal_Treatment, Rival_Treatment) %>%
 dplyr::summarise(mean = mean(Lifespan),
 sd = sd(Lifespan),
 N = n())

`summarise()` has grouped output by 'Focal_Treatment'. You can override using
the `.groups` argument.

AntennaeRemovedExp_WT_Single <- AR_lifespan_Cens %>%
 filter(Rival_Treatment == "Single", Focal_Treatment == "WT") %>%
 select(Lifespan)
AntennaeRemovedExp_WT_WC <- AR_lifespan_Cens %>%
 filter(Rival_Treatment == "WingClip", Focal_Treatment == "WT") %>%
 select(Lifespan)
AntennaeRemovedExp_WT_WR <- AR_lifespan_Cens %>%
 filter(Rival_Treatment == "WingRemoved", Focal_Treatment == "WT") %>%
 select(Lifespan)
cohen.d(AntennaeRemovedExp_WT_Single$Lifespan, AntennaeRemovedExp_WT_WC$Lifespan)

Cohen's d

d estimate: 0.2283953 (small)
95 percent confidence interval:
 lower upper
-0.0587761 0.5155668

cohen.d(AntennaeRemovedExp_WT_Single$Lifespan, AntennaeRemovedExp_WT_WR$Lifespan)

Cohen's d

d estimate: 0.3070313 (small)
95 percent confidence interval:
 lower upper
0.01751359 0.59654901

cohen.d(AntennaeRemovedExp_WT_WR$Lifespan, AntennaeRemovedExp_WT_WC$Lifespan)

Cohen's d

d estimate: -0.089926 (negligible)
95 percent confidence interval:
 lower upper
-0.3794149 0.1995630

AntennaeRemovedExp_AntennaeRemoved_Single <- AR_lifespan_Cens %>%
 filter(Rival_Treatment == "Single", Focal_Treatment == "AntennaeRemoved") %>%
 select(Lifespan)
AntennaeRemovedExp_AntennaeRemoved_WC <- AR_lifespan_Cens %>%
 filter(Rival_Treatment == "WingClip", Focal_Treatment == "AntennaeRemoved") %>%
 select(Lifespan)
AntennaeRemovedExp_AntennaeRemoved_WR <- AR_lifespan_Cens %>%
 filter(Rival_Treatment == "WingRemoved", Focal_Treatment == "AntennaeRemoved") %>%
 select(Lifespan)
cohen.d(AntennaeRemovedExp_AntennaeRemoved_Single$Lifespan, AntennaeRemovedExp_AntennaeRemoved_WC$Lifespan)

Cohen's d

d estimate: 0.3848439 (small)
95 percent confidence interval:
 lower upper
0.09596692 0.67372092

cohen.d(AntennaeRemovedExp_AntennaeRemoved_Single$Lifespan, AntennaeRemovedExp_AntennaeRemoved_WR$Lifespan)

Cohen's d

d estimate: 0.3350834 (small)
95 percent confidence interval:
 lower upper
0.04832111 0.62184572

cohen.d(AntennaeRemovedExp_AntennaeRemoved_WR$Lifespan, AntennaeRemovedExp_AntennaeRemoved_WC$Lifespan)

Cohen's d

d estimate: 0.06500977 (negligible)
95 percent confidence interval:
 lower upper
-0.2182997 0.3483193

## Lifelong Behaviour

**Supplementary Figure 3 & 4**

**Description of Data:** Behavioural scans of social treatment and senses of WT and Antennae Removed Flies flies when kept solo versus when they were with a rival having a clipped wing or a completely removed wing.

**Factors:**

- FlyID-Unique fly ID
- Focal_Treatment-Manipulation to the focal fly. (WT=not manipulated, AntennaeRemoved=antennae surgically removed 24h post eclosion)
- Rival_Treatment-Social Treatment. Single=focal fly kept in isolation post eclosion, WingClip=fly kept with single rival with a wing clip from eclosion to mating, WingRemoved=fly kept with a rival with wings surgical removed
- Week-Week of observation (weeks since eclosion)
- Observation-Behaviour observed of focal fly (W=walking, F=inactive on food, I=inactive, A=aggressive behaviour, G=grooming [not analysed], NA = no recorded behaviour)
- Paired-Interactive behaviour of focal fly observed. (WBL = within one body length, 1=wing flicks, 2=chasing, 3=boxing)
- n-Number of times behaviour observed per fly, per week

###loading packages
library(tidyverse)
library(dplyr)
library(ggplot2)
library(lme4)
library(emmeans)
library(glmmTMB)
library(mgcv)
library(ggstance)
library(ggpubr)
library(effsize)
library(Rmisc)

###required data
AntennaeBehaviour_all <- read.csv("AntennaeBehaviour_all.csv")
AntennaeBehaviour_all <- AntennaeBehaviour_all[!is.na(AntennaeBehaviour_all$Week), ]
AntennaeGroup_all <- read.csv("AntennaeBehaviour_paired.csv")
AntennaeGroup_all <- AntennaeGroup_all[!is.na(AntennaeGroup_all$Week), ]

AntennaeBehaviour_WC <- subset(AntennaeBehaviour_all, Rival_Treatment == "WingClip")
AntennaeBehaviour_WR <- subset(AntennaeBehaviour_all, Rival_Treatment == "WingRemoved")


###Success & Failures
AntennaeBehaviour_all$Success <- AntennaeBehaviour_all$n
AntennaeBehaviour_all$Failure <- 10 - AntennaeBehaviour_all$n
AntennaeGroup_all$Success <- AntennaeGroup_all$n
AntennaeGroup_all$Failure <- 10 - AntennaeGroup_all$n

###Subsetting Data
Antennae_Food <- subset(AntennaeBehaviour_all, Behaviour == "F")
Antennae_Walking <- subset(AntennaeBehaviour_all, Behaviour == "W")
Antennae_Grooming <- subset(AntennaeBehaviour_all, Behaviour == "G")
Antennae_Inactive <- subset(AntennaeBehaviour_all, Behaviour == "I")
Antennae_Aggression <- subset(AntennaeBehaviour_all, Behaviour == "A")
Antennae_WBL <- subset(AntennaeGroup_all, Paired == "WBL")


###Antennae Food
##Antennae Food Model
Antennae_Food$Week <- as.factor(Antennae_Food$Week)
AntennaeFeeding_glmmTMB1 <- glmmTMB(Success ~ Focal_Treatment * Rival_Treatment + Week + (1|Fly_ID), family=poisson(link = "log"), data = Antennae_Food)
AntennaeFeeding_glmmTMB2 <- glmmTMB(Success ~ Focal_Treatment + Rival_Treatment + Week + (1|Fly_ID), family=poisson(link = "log"), data = Antennae_Food)
anova(AntennaeFeeding_glmmTMB1, AntennaeFeeding_glmmTMB2, test="Chi")

Data: Antennae_Food
Models:
AntennaeFeeding_glmmTMB2: Success ~ Focal_Treatment + Rival_Treatment + Week + (1 | Fly_ID), zi=~0, disp=~1
AntennaeFeeding_glmmTMB1: Success ~ Focal_Treatment * Rival_Treatment + Week + (1 | Fly_ID), zi=~0, disp=~1
 Df AIC BIC logLik deviance Chisq Chi Df
AntennaeFeeding_glmmTMB2 9 5178.9 5224.9 -2580.5 5160.9
AntennaeFeeding_glmmTMB1 11 5158.8 5215.0 -2568.4 5136.8 24.15 2
 Pr(>Chisq)
AntennaeFeeding_glmmTMB2
AntennaeFeeding_glmmTMB1 5.7e-06 ***
---
Signif. codes: 0 '***' 0.001 '**' 0.01 '*' 0.05 '.' 0.1 ' ' 1

AntennaeFeeding_glmmTMB3 <- glmmTMB(Success ~ Focal_Treatment + Week + (1|Fly_ID), family=poisson(link = "log"), data = Antennae_Food)
anova(AntennaeFeeding_glmmTMB1, AntennaeFeeding_glmmTMB3, test="Chi")

Data: Antennae_Food
Models:
AntennaeFeeding_glmmTMB3: Success ~ Focal_Treatment + Week + (1 | Fly_ID), zi=~0, disp=~1
AntennaeFeeding_glmmTMB1: Success ~ Focal_Treatment * Rival_Treatment + Week + (1 | Fly_ID), zi=~0, disp=~1
 Df AIC BIC logLik deviance Chisq Chi Df
AntennaeFeeding_glmmTMB3 7 5189.9 5225.7 -2587.9 5175.9
AntennaeFeeding_glmmTMB1 11 5158.8 5215.0 -2568.4 5136.8 39.124 4
 Pr(>Chisq)
AntennaeFeeding_glmmTMB3
AntennaeFeeding_glmmTMB1 6.567e-08 ***
---
Signif. codes: 0 '***' 0.001 '**' 0.01 '*' 0.05 '.' 0.1 ' ' 1

AntennaeFeeding_glmmTMB4 <- glmmTMB(Success ~ Rival_Treatment + Week + (1|Fly_ID), family=poisson(link = "log"), data = Antennae_Food)
anova(AntennaeFeeding_glmmTMB1, AntennaeFeeding_glmmTMB4, test="Chi")

Data: Antennae_Food
Models:
AntennaeFeeding_glmmTMB4: Success ~ Rival_Treatment + Week + (1 | Fly_ID), zi=~0, disp=~1
AntennaeFeeding_glmmTMB1: Success ~ Focal_Treatment * Rival_Treatment + Week + (1 | Fly_ID), zi=~0, disp=~1
 Df AIC BIC logLik deviance Chisq Chi Df
AntennaeFeeding_glmmTMB4 8 5252.4 5293.3 -2618.2 5236.4
AntennaeFeeding_glmmTMB1 11 5158.8 5215.0 -2568.4 5136.8 99.635 3
 Pr(>Chisq)
AntennaeFeeding_glmmTMB4
AntennaeFeeding_glmmTMB1 < 2.2e-16 ***
---
Signif. codes: 0 '***' 0.001 '**' 0.01 '*' 0.05 '.' 0.1 ' ' 1

AntennaeFeeding_glmmTMB5 <- glmmTMB(Success ~ Focal_Treatment * Rival_Treatment + (1|Fly_ID), family=poisson(link = "log"), data = Antennae_Food)
anova(AntennaeFeeding_glmmTMB1, AntennaeFeeding_glmmTMB5, test="Chi")

Data: Antennae_Food
Models:
AntennaeFeeding_glmmTMB5: Success ~ Focal_Treatment * Rival_Treatment + (1 | Fly_ID), zi=~0, disp=~1
AntennaeFeeding_glmmTMB1: Success ~ Focal_Treatment * Rival_Treatment + Week + (1 | Fly_ID), zi=~0, disp=~1
 Df AIC BIC logLik deviance Chisq Chi Df
AntennaeFeeding_glmmTMB5 7 5615.7 5651.5 -2800.8 5601.7
AntennaeFeeding_glmmTMB1 11 5158.8 5215.0 -2568.4 5136.8 464.92 4
 Pr(>Chisq)
AntennaeFeeding_glmmTMB5
AntennaeFeeding_glmmTMB1 < 2.2e-16 ***
---
Signif. codes: 0 '***' 0.001 '**' 0.01 '*' 0.05 '.' 0.1 ' ' 1

AntennaeFeeding_glmmTMB_null <- glmmTMB(Success ~ (1|Fly_ID), family=poisson(link = "log"), data = Antennae_Food)
anova(AntennaeFeeding_glmmTMB1, AntennaeFeeding_glmmTMB_null, test="Chi")

Data: Antennae_Food
Models:
AntennaeFeeding_glmmTMB_null: Success ~ (1 | Fly_ID), zi=~0, disp=~1
AntennaeFeeding_glmmTMB1: Success ~ Focal_Treatment * Rival_Treatment + Week + (1 | Fly_ID), zi=~0, disp=~1
 Df AIC BIC logLik deviance Chisq Chi Df
AntennaeFeeding_glmmTMB_null 2 5718.1 5728.3 -2857.0 5714.1
AntennaeFeeding_glmmTMB1 11 5158.8 5215.0 -2568.4 5136.8 577.32 9
 Pr(>Chisq)
AntennaeFeeding_glmmTMB_null
AntennaeFeeding_glmmTMB1 < 2.2e-16 ***
---
Signif. codes: 0 '***' 0.001 '**' 0.01 '*' 0.05 '.' 0.1 ' ' 1

summary(AntennaeFeeding_glmmTMB1)

Family: poisson ( log )
Formula:
Success ~ Focal_Treatment * Rival_Treatment + Week + (1 | Fly_ID)
Data: Antennae_Food

 AIC BIC logLik deviance df.resid
 5158.8 5215.0 -2568.4 5136.8 1216

Random effects:

Conditional model:
 Groups Name Variance Std.Dev.
 Fly_ID (Intercept) 0.1973 0.4442
Number of obs: 1227, groups: Fly_ID, 259

Conditional model:
 Estimate Std. Error z value
(Intercept) 1.310634 0.086651 15.125
Focal_TreatmentWT -1.094106 0.115609 -9.464
Rival_TreatmentWingClip -0.197023 0.118920 -1.657
Rival_TreatmentWingRemoved 0.009792 0.112309 0.087
Week2 -0.472578 0.060047 -7.870
Week3 0.113153 0.051451 2.199
Week4 0.476491 0.048111 9.904
Week5 0.531151 0.048536 10.943
Focal_TreatmentWT:Rival_TreatmentWingClip 0.769512 0.164097 4.689
Focal_TreatmentWT:Rival_TreatmentWingRemoved 0.611854 0.159420 3.838
 Pr(>|z|)
(Intercept) < 2e-16 ***
Focal_TreatmentWT < 2e-16 ***
Rival_TreatmentWingClip 0.097568 .
Rival_TreatmentWingRemoved 0.930524
Week2 3.54e-15 ***
Week3 0.027862 *
Week4 < 2e-16 ***
Week5 < 2e-16 ***
Focal_TreatmentWT:Rival_TreatmentWingClip 2.74e-06 ***
Focal_TreatmentWT:Rival_TreatmentWingRemoved 0.000124 ***
---
Signif. codes: 0 '***' 0.001 '**' 0.01 '*' 0.05 '.' 0.1 ' ' 1

lsmeans(AntennaeFeeding_glmmTMB1, pairwise ~ Focal_Treatment * Rival_Treatment * Week, adjust="tukey")

$lsmeans
 Focal_Treatment Rival_Treatment Week lsmean SE df asymp.LCL asymp.UCL
 AntennaeRemoved Single 1 1.311 0.0867 Inf 1.1408 1.4805
 WT Single 1 0.217 0.0905 Inf 0.0391 0.3940
 AntennaeRemoved WingClip 1 1.114 0.0944 Inf 0.9285 1.2987
 WT WingClip 1 0.789 0.0833 Inf 0.6257 0.9524
 AntennaeRemoved WingRemoved 1 1.320 0.0856 Inf 1.1527 1.4881
 WT WingRemoved 1 0.838 0.0837 Inf 0.6741 1.0022
 AntennaeRemoved Single 2 0.838 0.0917 Inf 0.6584 1.0178
 WT Single 2 -0.256 0.0951 Inf -0.4425 -0.0696
 AntennaeRemoved WingClip 2 0.641 0.0988 Inf 0.4473 0.8347
 WT WingClip 2 0.316 0.0886 Inf 0.1428 0.4901
 AntennaeRemoved WingRemoved 2 0.848 0.0903 Inf 0.6708 1.0249
 WT WingRemoved 2 0.366 0.0888 Inf 0.1916 0.5396
 AntennaeRemoved Single 3 1.424 0.0864 Inf 1.2545 1.5931
 WT Single 3 0.330 0.0899 Inf 0.1534 0.5060
 AntennaeRemoved WingClip 3 1.227 0.0939 Inf 1.0426 1.4109
 WT WingClip 3 0.902 0.0830 Inf 0.7394 1.0649
 AntennaeRemoved WingRemoved 3 1.434 0.0850 Inf 1.2670 1.6002
 WT WingRemoved 3 0.951 0.0831 Inf 0.7884 1.1142
 AntennaeRemoved Single 4 1.787 0.0842 Inf 1.6221 1.9522
 WT Single 4 0.693 0.0880 Inf 0.5205 0.8655
 AntennaeRemoved WingClip 4 1.590 0.0919 Inf 1.4099 1.7703
 WT WingClip 4 1.266 0.0810 Inf 1.1067 1.4243
 AntennaeRemoved WingRemoved 4 1.797 0.0833 Inf 1.6336 1.9602
 WT WingRemoved 4 1.315 0.0810 Inf 1.1559 1.4734
 AntennaeRemoved Single 5 1.842 0.0843 Inf 1.6765 2.0071
 WT Single 5 0.748 0.0881 Inf 0.5751 0.9203
 AntennaeRemoved WingClip 5 1.645 0.0919 Inf 1.4647 1.8248
 WT WingClip 5 1.320 0.0810 Inf 1.1614 1.4790
 AntennaeRemoved WingRemoved 5 1.852 0.0837 Inf 1.6875 2.0156
 WT WingRemoved 5 1.369 0.0810 Inf 1.2106 1.5281

Results are given on the log (not the response) scale.
Confidence level used: 0.95

$contrasts
 contrast
 AntennaeRemoved Single Week1 - WT Single Week1
 AntennaeRemoved Single Week1 - AntennaeRemoved WingClip Week1
 AntennaeRemoved Single Week1 - WT WingClip Week1
 AntennaeRemoved Single Week1 - AntennaeRemoved WingRemoved Week1
 AntennaeRemoved Single Week1 - WT WingRemoved Week1
 AntennaeRemoved Single Week1 - AntennaeRemoved Single Week2
 AntennaeRemoved Single Week1 - WT Single Week2
 AntennaeRemoved Single Week1 - AntennaeRemoved WingClip Week2
 AntennaeRemoved Single Week1 - WT WingClip Week2
 AntennaeRemoved Single Week1 - AntennaeRemoved WingRemoved Week2
 AntennaeRemoved Single Week1 - WT WingRemoved Week2
 AntennaeRemoved Single Week1 - AntennaeRemoved Single Week3
 AntennaeRemoved Single Week1 - WT Single Week3
 AntennaeRemoved Single Week1 - AntennaeRemoved WingClip Week3
 AntennaeRemoved Single Week1 - WT WingClip Week3
 AntennaeRemoved Single Week1 - AntennaeRemoved WingRemoved Week3
 AntennaeRemoved Single Week1 - WT WingRemoved Week3
 AntennaeRemoved Single Week1 - AntennaeRemoved Single Week4
 AntennaeRemoved Single Week1 - WT Single Week4
 AntennaeRemoved Single Week1 - AntennaeRemoved WingClip Week4
 AntennaeRemoved Single Week1 - WT WingClip Week4
 AntennaeRemoved Single Week1 - AntennaeRemoved WingRemoved Week4
 AntennaeRemoved Single Week1 - WT WingRemoved Week4
 AntennaeRemoved Single Week1 - AntennaeRemoved Single Week5
 AntennaeRemoved Single Week1 - WT Single Week5
 AntennaeRemoved Single Week1 - AntennaeRemoved WingClip Week5
 AntennaeRemoved Single Week1 - WT WingClip Week5
 AntennaeRemoved Single Week1 - AntennaeRemoved WingRemoved Week5
 AntennaeRemoved Single Week1 - WT WingRemoved Week5
 WT Single Week1 - AntennaeRemoved WingClip Week1
 WT Single Week1 - WT WingClip Week1
 WT Single Week1 - AntennaeRemoved WingRemoved Week1
 WT Single Week1 - WT WingRemoved Week1
 WT Single Week1 - AntennaeRemoved Single Week2
 WT Single Week1 - WT Single Week2
 WT Single Week1 - AntennaeRemoved WingClip Week2
 WT Single Week1 - WT WingClip Week2
 WT Single Week1 - AntennaeRemoved WingRemoved Week2
 WT Single Week1 - WT WingRemoved Week2
 WT Single Week1 - AntennaeRemoved Single Week3
 WT Single Week1 - WT Single Week3
 WT Single Week1 - AntennaeRemoved WingClip Week3
 WT Single Week1 - WT WingClip Week3
 WT Single Week1 - AntennaeRemoved WingRemoved Week3
 WT Single Week1 - WT WingRemoved Week3
 WT Single Week1 - AntennaeRemoved Single Week4
 WT Single Week1 - WT Single Week4
 WT Single Week1 - AntennaeRemoved WingClip Week4
 WT Single Week1 - WT WingClip Week4
 WT Single Week1 - AntennaeRemoved WingRemoved Week4
 WT Single Week1 - WT WingRemoved Week4
 WT Single Week1 - AntennaeRemoved Single Week5
 WT Single Week1 - WT Single Week5
 WT Single Week1 - AntennaeRemoved WingClip Week5
 WT Single Week1 - WT WingClip Week5
 WT Single Week1 - AntennaeRemoved WingRemoved Week5
 WT Single Week1 - WT WingRemoved Week5
 AntennaeRemoved WingClip Week1 - WT WingClip Week1
 AntennaeRemoved WingClip Week1 - AntennaeRemoved WingRemoved Week1
 AntennaeRemoved WingClip Week1 - WT WingRemoved Week1
 AntennaeRemoved WingClip Week1 - AntennaeRemoved Single Week2
 AntennaeRemoved WingClip Week1 - WT Single Week2
 AntennaeRemoved WingClip Week1 - AntennaeRemoved WingClip Week2
 AntennaeRemoved WingClip Week1 - WT WingClip Week2
 AntennaeRemoved WingClip Week1 - AntennaeRemoved WingRemoved Week2
 AntennaeRemoved WingClip Week1 - WT WingRemoved Week2
 AntennaeRemoved WingClip Week1 - AntennaeRemoved Single Week3
 AntennaeRemoved WingClip Week1 - WT Single Week3
 AntennaeRemoved WingClip Week1 - AntennaeRemoved WingClip Week3
 AntennaeRemoved WingClip Week1 - WT WingClip Week3
 AntennaeRemoved WingClip Week1 - AntennaeRemoved WingRemoved Week3
 AntennaeRemoved WingClip Week1 - WT WingRemoved Week3
 AntennaeRemoved WingClip Week1 - AntennaeRemoved Single Week4
 AntennaeRemoved WingClip Week1 - WT Single Week4
 AntennaeRemoved WingClip Week1 - AntennaeRemoved WingClip Week4
 AntennaeRemoved WingClip Week1 - WT WingClip Week4
 AntennaeRemoved WingClip Week1 - AntennaeRemoved WingRemoved Week4
 AntennaeRemoved WingClip Week1 - WT WingRemoved Week4
 AntennaeRemoved WingClip Week1 - AntennaeRemoved Single Week5
 AntennaeRemoved WingClip Week1 - WT Single Week5
 AntennaeRemoved WingClip Week1 - AntennaeRemoved WingClip Week5
 AntennaeRemoved WingClip Week1 - WT WingClip Week5
 AntennaeRemoved WingClip Week1 - AntennaeRemoved WingRemoved Week5
 AntennaeRemoved WingClip Week1 - WT WingRemoved Week5
 WT WingClip Week1 - AntennaeRemoved WingRemoved Week1
 WT WingClip Week1 - WT WingRemoved Week1
 WT WingClip Week1 - AntennaeRemoved Single Week2
 WT WingClip Week1 - WT Single Week2
 WT WingClip Week1 - AntennaeRemoved WingClip Week2
 WT WingClip Week1 - WT WingClip Week2
 WT WingClip Week1 - AntennaeRemoved WingRemoved Week2
 WT WingClip Week1 - WT WingRemoved Week2
 WT WingClip Week1 - AntennaeRemoved Single Week3
 WT WingClip Week1 - WT Single Week3
 WT WingClip Week1 - AntennaeRemoved WingClip Week3
 WT WingClip Week1 - WT WingClip Week3
 WT WingClip Week1 - AntennaeRemoved WingRemoved Week3
 WT WingClip Week1 - WT WingRemoved Week3
 WT WingClip Week1 - AntennaeRemoved Single Week4
 WT WingClip Week1 - WT Single Week4
 WT WingClip Week1 - AntennaeRemoved WingClip Week4
 WT WingClip Week1 - WT WingClip Week4
 WT WingClip Week1 - AntennaeRemoved WingRemoved Week4
 WT WingClip Week1 - WT WingRemoved Week4
 WT WingClip Week1 - AntennaeRemoved Single Week5
 WT WingClip Week1 - WT Single Week5
 WT WingClip Week1 - AntennaeRemoved WingClip Week5
 WT WingClip Week1 - WT WingClip Week5
 WT WingClip Week1 - AntennaeRemoved WingRemoved Week5
 WT WingClip Week1 - WT WingRemoved Week5
 AntennaeRemoved WingRemoved Week1 - WT WingRemoved Week1
 AntennaeRemoved WingRemoved Week1 - AntennaeRemoved Single Week2
 AntennaeRemoved WingRemoved Week1 - WT Single Week2
 AntennaeRemoved WingRemoved Week1 - AntennaeRemoved WingClip Week2
 AntennaeRemoved WingRemoved Week1 - WT WingClip Week2
 AntennaeRemoved WingRemoved Week1 - AntennaeRemoved WingRemoved Week2
 AntennaeRemoved WingRemoved Week1 - WT WingRemoved Week2
 AntennaeRemoved WingRemoved Week1 - AntennaeRemoved Single Week3
 AntennaeRemoved WingRemoved Week1 - WT Single Week3
 AntennaeRemoved WingRemoved Week1 - AntennaeRemoved WingClip Week3
 AntennaeRemoved WingRemoved Week1 - WT WingClip Week3
 AntennaeRemoved WingRemoved Week1 - AntennaeRemoved WingRemoved Week3
 AntennaeRemoved WingRemoved Week1 - WT WingRemoved Week3
 AntennaeRemoved WingRemoved Week1 - AntennaeRemoved Single Week4
 AntennaeRemoved WingRemoved Week1 - WT Single Week4
 AntennaeRemoved WingRemoved Week1 - AntennaeRemoved WingClip Week4
 AntennaeRemoved WingRemoved Week1 - WT WingClip Week4
 AntennaeRemoved WingRemoved Week1 - AntennaeRemoved WingRemoved Week4
 AntennaeRemoved WingRemoved Week1 - WT WingRemoved Week4
 AntennaeRemoved WingRemoved Week1 - AntennaeRemoved Single Week5
 AntennaeRemoved WingRemoved Week1 - WT Single Week5
 AntennaeRemoved WingRemoved Week1 - AntennaeRemoved WingClip Week5
 AntennaeRemoved WingRemoved Week1 - WT WingClip Week5
 AntennaeRemoved WingRemoved Week1 - AntennaeRemoved WingRemoved Week5
 AntennaeRemoved WingRemoved Week1 - WT WingRemoved Week5
 WT WingRemoved Week1 - AntennaeRemoved Single Week2
 WT WingRemoved Week1 - WT Single Week2
 WT WingRemoved Week1 - AntennaeRemoved WingClip Week2
 WT WingRemoved Week1 - WT WingClip Week2
 WT WingRemoved Week1 - AntennaeRemoved WingRemoved Week2
 WT WingRemoved Week1 - WT WingRemoved Week2
 WT WingRemoved Week1 - AntennaeRemoved Single Week3
 WT WingRemoved Week1 - WT Single Week3
 WT WingRemoved Week1 - AntennaeRemoved WingClip Week3
 WT WingRemoved Week1 - WT WingClip Week3
 WT WingRemoved Week1 - AntennaeRemoved WingRemoved Week3
 WT WingRemoved Week1 - WT WingRemoved Week3
 WT WingRemoved Week1 - AntennaeRemoved Single Week4
 WT WingRemoved Week1 - WT Single Week4
 WT WingRemoved Week1 - AntennaeRemoved WingClip Week4
 WT WingRemoved Week1 - WT WingClip Week4
 WT WingRemoved Week1 - AntennaeRemoved WingRemoved Week4
 WT WingRemoved Week1 - WT WingRemoved Week4
 WT WingRemoved Week1 - AntennaeRemoved Single Week5
 WT WingRemoved Week1 - WT Single Week5
 WT WingRemoved Week1 - AntennaeRemoved WingClip Week5
 WT WingRemoved Week1 - WT WingClip Week5
 WT WingRemoved Week1 - AntennaeRemoved WingRemoved Week5
 WT WingRemoved Week1 - WT WingRemoved Week5
 AntennaeRemoved Single Week2 - WT Single Week2
 AntennaeRemoved Single Week2 - AntennaeRemoved WingClip Week2
 AntennaeRemoved Single Week2 - WT WingClip Week2
 AntennaeRemoved Single Week2 - AntennaeRemoved WingRemoved Week2
 AntennaeRemoved Single Week2 - WT WingRemoved Week2
 AntennaeRemoved Single Week2 - AntennaeRemoved Single Week3
 AntennaeRemoved Single Week2 - WT Single Week3
 AntennaeRemoved Single Week2 - AntennaeRemoved WingClip Week3
 AntennaeRemoved Single Week2 - WT WingClip Week3
 AntennaeRemoved Single Week2 - AntennaeRemoved WingRemoved Week3
 AntennaeRemoved Single Week2 - WT WingRemoved Week3
 AntennaeRemoved Single Week2 - AntennaeRemoved Single Week4
 AntennaeRemoved Single Week2 - WT Single Week4
 AntennaeRemoved Single Week2 - AntennaeRemoved WingClip Week4
 AntennaeRemoved Single Week2 - WT WingClip Week4
 AntennaeRemoved Single Week2 - AntennaeRemoved WingRemoved Week4
 AntennaeRemoved Single Week2 - WT WingRemoved Week4
 AntennaeRemoved Single Week2 - AntennaeRemoved Single Week5
 AntennaeRemoved Single Week2 - WT Single Week5
 AntennaeRemoved Single Week2 - AntennaeRemoved WingClip Week5
 AntennaeRemoved Single Week2 - WT WingClip Week5
 AntennaeRemoved Single Week2 - AntennaeRemoved WingRemoved Week5
 AntennaeRemoved Single Week2 - WT WingRemoved Week5
 WT Single Week2 - AntennaeRemoved WingClip Week2
 WT Single Week2 - WT WingClip Week2
 WT Single Week2 - AntennaeRemoved WingRemoved Week2
 WT Single Week2 - WT WingRemoved Week2
 WT Single Week2 - AntennaeRemoved Single Week3
 WT Single Week2 - WT Single Week3
 WT Single Week2 - AntennaeRemoved WingClip Week3
 WT Single Week2 - WT WingClip Week3
 WT Single Week2 - AntennaeRemoved WingRemoved Week3
 WT Single Week2 - WT WingRemoved Week3
 WT Single Week2 - AntennaeRemoved Single Week4
 WT Single Week2 - WT Single Week4
 WT Single Week2 - AntennaeRemoved WingClip Week4
 WT Single Week2 - WT WingClip Week4
 WT Single Week2 - AntennaeRemoved WingRemoved Week4
 WT Single Week2 - WT WingRemoved Week4
 WT Single Week2 - AntennaeRemoved Single Week5
 WT Single Week2 - WT Single Week5
 WT Single Week2 - AntennaeRemoved WingClip Week5
 WT Single Week2 - WT WingClip Week5
 WT Single Week2 - AntennaeRemoved WingRemoved Week5
 WT Single Week2 - WT WingRemoved Week5
 AntennaeRemoved WingClip Week2 - WT WingClip Week2
 AntennaeRemoved WingClip Week2 - AntennaeRemoved WingRemoved Week2
 AntennaeRemoved WingClip Week2 - WT WingRemoved Week2
 AntennaeRemoved WingClip Week2 - AntennaeRemoved Single Week3
 AntennaeRemoved WingClip Week2 - WT Single Week3
 AntennaeRemoved WingClip Week2 - AntennaeRemoved WingClip Week3
 AntennaeRemoved WingClip Week2 - WT WingClip Week3
 AntennaeRemoved WingClip Week2 - AntennaeRemoved WingRemoved Week3
 AntennaeRemoved WingClip Week2 - WT WingRemoved Week3
 AntennaeRemoved WingClip Week2 - AntennaeRemoved Single Week4
 AntennaeRemoved WingClip Week2 - WT Single Week4
 AntennaeRemoved WingClip Week2 - AntennaeRemoved WingClip Week4
 AntennaeRemoved WingClip Week2 - WT WingClip Week4
 AntennaeRemoved WingClip Week2 - AntennaeRemoved WingRemoved Week4
 AntennaeRemoved WingClip Week2 - WT WingRemoved Week4
 AntennaeRemoved WingClip Week2 - AntennaeRemoved Single Week5
 AntennaeRemoved WingClip Week2 - WT Single Week5
 AntennaeRemoved WingClip Week2 - AntennaeRemoved WingClip Week5
 AntennaeRemoved WingClip Week2 - WT WingClip Week5
 AntennaeRemoved WingClip Week2 - AntennaeRemoved WingRemoved Week5
 AntennaeRemoved WingClip Week2 - WT WingRemoved Week5
 WT WingClip Week2 - AntennaeRemoved WingRemoved Week2
 WT WingClip Week2 - WT WingRemoved Week2
 WT WingClip Week2 - AntennaeRemoved Single Week3
 WT WingClip Week2 - WT Single Week3
 WT WingClip Week2 - AntennaeRemoved WingClip Week3
 WT WingClip Week2 - WT WingClip Week3
 WT WingClip Week2 - AntennaeRemoved WingRemoved Week3
 WT WingClip Week2 - WT WingRemoved Week3
 WT WingClip Week2 - AntennaeRemoved Single Week4
 WT WingClip Week2 - WT Single Week4
 WT WingClip Week2 - AntennaeRemoved WingClip Week4
 WT WingClip Week2 - WT WingClip Week4
 WT WingClip Week2 - AntennaeRemoved WingRemoved Week4
 WT WingClip Week2 - WT WingRemoved Week4
 WT WingClip Week2 - AntennaeRemoved Single Week5
 WT WingClip Week2 - WT Single Week5
 WT WingClip Week2 - AntennaeRemoved WingClip Week5
 WT WingClip Week2 - WT WingClip Week5
 WT WingClip Week2 - AntennaeRemoved WingRemoved Week5
 WT WingClip Week2 - WT WingRemoved Week5
 AntennaeRemoved WingRemoved Week2 - WT WingRemoved Week2
 AntennaeRemoved WingRemoved Week2 - AntennaeRemoved Single Week3
 AntennaeRemoved WingRemoved Week2 - WT Single Week3
 AntennaeRemoved WingRemoved Week2 - AntennaeRemoved WingClip Week3
 AntennaeRemoved WingRemoved Week2 - WT WingClip Week3
 AntennaeRemoved WingRemoved Week2 - AntennaeRemoved WingRemoved Week3
 AntennaeRemoved WingRemoved Week2 - WT WingRemoved Week3
 AntennaeRemoved WingRemoved Week2 - AntennaeRemoved Single Week4
 AntennaeRemoved WingRemoved Week2 - WT Single Week4
 AntennaeRemoved WingRemoved Week2 - AntennaeRemoved WingClip Week4
 AntennaeRemoved WingRemoved Week2 - WT WingClip Week4
 AntennaeRemoved WingRemoved Week2 - AntennaeRemoved WingRemoved Week4
 AntennaeRemoved WingRemoved Week2 - WT WingRemoved Week4
 AntennaeRemoved WingRemoved Week2 - AntennaeRemoved Single Week5
 AntennaeRemoved WingRemoved Week2 - WT Single Week5
 AntennaeRemoved WingRemoved Week2 - AntennaeRemoved WingClip Week5
 AntennaeRemoved WingRemoved Week2 - WT WingClip Week5
 AntennaeRemoved WingRemoved Week2 - AntennaeRemoved WingRemoved Week5
 AntennaeRemoved WingRemoved Week2 - WT WingRemoved Week5
 WT WingRemoved Week2 - AntennaeRemoved Single Week3
 WT WingRemoved Week2 - WT Single Week3
 WT WingRemoved Week2 - AntennaeRemoved WingClip Week3
 WT WingRemoved Week2 - WT WingClip Week3
 WT WingRemoved Week2 - AntennaeRemoved WingRemoved Week3
 WT WingRemoved Week2 - WT WingRemoved Week3
 WT WingRemoved Week2 - AntennaeRemoved Single Week4
 WT WingRemoved Week2 - WT Single Week4
 WT WingRemoved Week2 - AntennaeRemoved WingClip Week4
 WT WingRemoved Week2 - WT WingClip Week4
 WT WingRemoved Week2 - AntennaeRemoved WingRemoved Week4
 WT WingRemoved Week2 - WT WingRemoved Week4
 WT WingRemoved Week2 - AntennaeRemoved Single Week5
 WT WingRemoved Week2 - WT Single Week5
 WT WingRemoved Week2 - AntennaeRemoved WingClip Week5
 WT WingRemoved Week2 - WT WingClip Week5
 WT WingRemoved Week2 - AntennaeRemoved WingRemoved Week5
 WT WingRemoved Week2 - WT WingRemoved Week5
 AntennaeRemoved Single Week3 - WT Single Week3
 AntennaeRemoved Single Week3 - AntennaeRemoved WingClip Week3
 AntennaeRemoved Single Week3 - WT WingClip Week3
 AntennaeRemoved Single Week3 - AntennaeRemoved WingRemoved Week3
 AntennaeRemoved Single Week3 - WT WingRemoved Week3
 AntennaeRemoved Single Week3 - AntennaeRemoved Single Week4
 AntennaeRemoved Single Week3 - WT Single Week4
 AntennaeRemoved Single Week3 - AntennaeRemoved WingClip Week4
 AntennaeRemoved Single Week3 - WT WingClip Week4
 AntennaeRemoved Single Week3 - AntennaeRemoved WingRemoved Week4
 AntennaeRemoved Single Week3 - WT WingRemoved Week4
 AntennaeRemoved Single Week3 - AntennaeRemoved Single Week5
 AntennaeRemoved Single Week3 - WT Single Week5
 AntennaeRemoved Single Week3 - AntennaeRemoved WingClip Week5
 AntennaeRemoved Single Week3 - WT WingClip Week5
 AntennaeRemoved Single Week3 - AntennaeRemoved WingRemoved Week5
 AntennaeRemoved Single Week3 - WT WingRemoved Week5
 WT Single Week3 - AntennaeRemoved WingClip Week3
 WT Single Week3 - WT WingClip Week3
 WT Single Week3 - AntennaeRemoved WingRemoved Week3
 WT Single Week3 - WT WingRemoved Week3
 WT Single Week3 - AntennaeRemoved Single Week4
 WT Single Week3 - WT Single Week4
 WT Single Week3 - AntennaeRemoved WingClip Week4
 WT Single Week3 - WT WingClip Week4
 WT Single Week3 - AntennaeRemoved WingRemoved Week4
 WT Single Week3 - WT WingRemoved Week4
 WT Single Week3 - AntennaeRemoved Single Week5
 WT Single Week3 - WT Single Week5
 WT Single Week3 - AntennaeRemoved WingClip Week5
 WT Single Week3 - WT WingClip Week5
 WT Single Week3 - AntennaeRemoved WingRemoved Week5
 WT Single Week3 - WT WingRemoved Week5
 AntennaeRemoved WingClip Week3 - WT WingClip Week3
 AntennaeRemoved WingClip Week3 - AntennaeRemoved WingRemoved Week3
 AntennaeRemoved WingClip Week3 - WT WingRemoved Week3
 AntennaeRemoved WingClip Week3 - AntennaeRemoved Single Week4
 AntennaeRemoved WingClip Week3 - WT Single Week4
 AntennaeRemoved WingClip Week3 - AntennaeRemoved WingClip Week4
 AntennaeRemoved WingClip Week3 - WT WingClip Week4
 AntennaeRemoved WingClip Week3 - AntennaeRemoved WingRemoved Week4
 AntennaeRemoved WingClip Week3 - WT WingRemoved Week4
 AntennaeRemoved WingClip Week3 - AntennaeRemoved Single Week5
 AntennaeRemoved WingClip Week3 - WT Single Week5
 AntennaeRemoved WingClip Week3 - AntennaeRemoved WingClip Week5
 AntennaeRemoved WingClip Week3 - WT WingClip Week5
 AntennaeRemoved WingClip Week3 - AntennaeRemoved WingRemoved Week5
 AntennaeRemoved WingClip Week3 - WT WingRemoved Week5
 WT WingClip Week3 - AntennaeRemoved WingRemoved Week3
 WT WingClip Week3 - WT WingRemoved Week3
 WT WingClip Week3 - AntennaeRemoved Single Week4
 WT WingClip Week3 - WT Single Week4
 WT WingClip Week3 - AntennaeRemoved WingClip Week4
 WT WingClip Week3 - WT WingClip Week4
 WT WingClip Week3 - AntennaeRemoved WingRemoved Week4
 WT WingClip Week3 - WT WingRemoved Week4
 WT WingClip Week3 - AntennaeRemoved Single Week5
 WT WingClip Week3 - WT Single Week5
 WT WingClip Week3 - AntennaeRemoved WingClip Week5
 WT WingClip Week3 - WT WingClip Week5
 WT WingClip Week3 - AntennaeRemoved WingRemoved Week5
 WT WingClip Week3 - WT WingRemoved Week5
 AntennaeRemoved WingRemoved Week3 - WT WingRemoved Week3
 AntennaeRemoved WingRemoved Week3 - AntennaeRemoved Single Week4
 AntennaeRemoved WingRemoved Week3 - WT Single Week4
 AntennaeRemoved WingRemoved Week3 - AntennaeRemoved WingClip Week4
 AntennaeRemoved WingRemoved Week3 - WT WingClip Week4
 AntennaeRemoved WingRemoved Week3 - AntennaeRemoved WingRemoved Week4
 AntennaeRemoved WingRemoved Week3 - WT WingRemoved Week4
 AntennaeRemoved WingRemoved Week3 - AntennaeRemoved Single Week5
 AntennaeRemoved WingRemoved Week3 - WT Single Week5
 AntennaeRemoved WingRemoved Week3 - AntennaeRemoved WingClip Week5
 AntennaeRemoved WingRemoved Week3 - WT WingClip Week5
 AntennaeRemoved WingRemoved Week3 - AntennaeRemoved WingRemoved Week5
 AntennaeRemoved WingRemoved Week3 - WT WingRemoved Week5
 WT WingRemoved Week3 - AntennaeRemoved Single Week4
 WT WingRemoved Week3 - WT Single Week4
 WT WingRemoved Week3 - AntennaeRemoved WingClip Week4
 WT WingRemoved Week3 - WT WingClip Week4
 WT WingRemoved Week3 - AntennaeRemoved WingRemoved Week4
 WT WingRemoved Week3 - WT WingRemoved Week4
 WT WingRemoved Week3 - AntennaeRemoved Single Week5
 WT WingRemoved Week3 - WT Single Week5
 WT WingRemoved Week3 - AntennaeRemoved WingClip Week5
 WT WingRemoved Week3 - WT WingClip Week5
 WT WingRemoved Week3 - AntennaeRemoved WingRemoved Week5
 WT WingRemoved Week3 - WT WingRemoved Week5
 AntennaeRemoved Single Week4 - WT Single Week4
 AntennaeRemoved Single Week4 - AntennaeRemoved WingClip Week4
 AntennaeRemoved Single Week4 - WT WingClip Week4
 AntennaeRemoved Single Week4 - AntennaeRemoved WingRemoved Week4
 AntennaeRemoved Single Week4 - WT WingRemoved Week4
 AntennaeRemoved Single Week4 - AntennaeRemoved Single Week5
 AntennaeRemoved Single Week4 - WT Single Week5
 AntennaeRemoved Single Week4 - AntennaeRemoved WingClip Week5
 AntennaeRemoved Single Week4 - WT WingClip Week5
 AntennaeRemoved Single Week4 - AntennaeRemoved WingRemoved Week5
 AntennaeRemoved Single Week4 - WT WingRemoved Week5
 WT Single Week4 - AntennaeRemoved WingClip Week4
 WT Single Week4 - WT WingClip Week4
 WT Single Week4 - AntennaeRemoved WingRemoved Week4
 WT Single Week4 - WT WingRemoved Week4
 WT Single Week4 - AntennaeRemoved Single Week5
 WT Single Week4 - WT Single Week5
 WT Single Week4 - AntennaeRemoved WingClip Week5
 WT Single Week4 - WT WingClip Week5
 WT Single Week4 - AntennaeRemoved WingRemoved Week5
 WT Single Week4 - WT WingRemoved Week5
 AntennaeRemoved WingClip Week4 - WT WingClip Week4
 AntennaeRemoved WingClip Week4 - AntennaeRemoved WingRemoved Week4
 AntennaeRemoved WingClip Week4 - WT WingRemoved Week4
 AntennaeRemoved WingClip Week4 - AntennaeRemoved Single Week5
 AntennaeRemoved WingClip Week4 - WT Single Week5
 AntennaeRemoved WingClip Week4 - AntennaeRemoved WingClip Week5
 AntennaeRemoved WingClip Week4 - WT WingClip Week5
 AntennaeRemoved WingClip Week4 - AntennaeRemoved WingRemoved Week5
 AntennaeRemoved WingClip Week4 - WT WingRemoved Week5
 WT WingClip Week4 - AntennaeRemoved WingRemoved Week4
 WT WingClip Week4 - WT WingRemoved Week4
 WT WingClip Week4 - AntennaeRemoved Single Week5
 WT WingClip Week4 - WT Single Week5
 WT WingClip Week4 - AntennaeRemoved WingClip Week5
 WT WingClip Week4 - WT WingClip Week5
 WT WingClip Week4 - AntennaeRemoved WingRemoved Week5
 WT WingClip Week4 - WT WingRemoved Week5
 AntennaeRemoved WingRemoved Week4 - WT WingRemoved Week4
 AntennaeRemoved WingRemoved Week4 - AntennaeRemoved Single Week5
 AntennaeRemoved WingRemoved Week4 - WT Single Week5
 AntennaeRemoved WingRemoved Week4 - AntennaeRemoved WingClip Week5
 AntennaeRemoved WingRemoved Week4 - WT WingClip Week5
 AntennaeRemoved WingRemoved Week4 - AntennaeRemoved WingRemoved Week5
 AntennaeRemoved WingRemoved Week4 - WT WingRemoved Week5
 WT WingRemoved Week4 - AntennaeRemoved Single Week5
 WT WingRemoved Week4 - WT Single Week5
 WT WingRemoved Week4 - AntennaeRemoved WingClip Week5
 WT WingRemoved Week4 - WT WingClip Week5
 WT WingRemoved Week4 - AntennaeRemoved WingRemoved Week5
 WT WingRemoved Week4 - WT WingRemoved Week5
 AntennaeRemoved Single Week5 - WT Single Week5
 AntennaeRemoved Single Week5 - AntennaeRemoved WingClip Week5
 AntennaeRemoved Single Week5 - WT WingClip Week5
 AntennaeRemoved Single Week5 - AntennaeRemoved WingRemoved Week5
 AntennaeRemoved Single Week5 - WT WingRemoved Week5
 WT Single Week5 - AntennaeRemoved WingClip Week5
 WT Single Week5 - WT WingClip Week5
 WT Single Week5 - AntennaeRemoved WingRemoved Week5
 WT Single Week5 - WT WingRemoved Week5
 AntennaeRemoved WingClip Week5 - WT WingClip Week5
 AntennaeRemoved WingClip Week5 - AntennaeRemoved WingRemoved Week5
 AntennaeRemoved WingClip Week5 - WT WingRemoved Week5
 WT WingClip Week5 - AntennaeRemoved WingRemoved Week5
 WT WingClip Week5 - WT WingRemoved Week5
 AntennaeRemoved WingRemoved Week5 - WT WingRemoved Week5
 estimate SE df z.ratio p.value
 1.094106 0.1156 Inf 9.464 <.0001
 0.197023 0.1189 Inf 1.657 0.9988
 0.521617 0.1105 Inf 4.719 0.0009
 -0.009792 0.1123 Inf -0.087 1.0000
 0.472461 0.1106 Inf 4.270 0.0069
 0.472578 0.0600 Inf 7.870 <.0001
 1.566683 0.1301 Inf 12.041 <.0001
 0.669601 0.1330 Inf 5.034 0.0002
 0.994194 0.1258 Inf 7.901 <.0001
 0.462786 0.1271 Inf 3.641 0.0715
 0.945038 0.1258 Inf 7.513 <.0001
 -0.113153 0.0515 Inf -2.199 0.9341
 0.980953 0.1263 Inf 7.766 <.0001
 0.083870 0.1294 Inf 0.648 1.0000
 0.408464 0.1219 Inf 3.350 0.1692
 -0.122945 0.1233 Inf -0.997 1.0000
 0.359308 0.1218 Inf 2.950 0.4230
 -0.476491 0.0481 Inf -9.904 <.0001
 0.617615 0.1251 Inf 4.937 0.0003
 -0.279468 0.1281 Inf -2.182 0.9396
 0.045126 0.1207 Inf 0.374 1.0000
 -0.486282 0.1223 Inf -3.975 0.0222
 -0.004030 0.1205 Inf -0.033 1.0000
 -0.531151 0.0485 Inf -10.943 <.0001
 0.562955 0.1252 Inf 4.497 0.0026
 -0.334128 0.1281 Inf -2.608 0.7020
 -0.009534 0.1208 Inf -0.079 1.0000
 -0.540943 0.1227 Inf -4.409 0.0038
 -0.058690 0.1206 Inf -0.487 1.0000
 -0.897083 0.1214 Inf -7.392 <.0001
 -0.572489 0.1131 Inf -5.063 0.0002
 -1.103898 0.1149 Inf -9.604 <.0001
 -0.621645 0.1132 Inf -5.492 <.0001
 -0.621528 0.1304 Inf -4.765 0.0007
 0.472578 0.0600 Inf 7.870 <.0001
 -0.424505 0.1354 Inf -3.136 0.2880
 -0.099911 0.1282 Inf -0.779 1.0000
 -0.631320 0.1296 Inf -4.871 0.0004
 -0.149068 0.1282 Inf -1.163 1.0000
 -1.207259 0.1268 Inf -9.523 <.0001
 -0.113153 0.0515 Inf -2.199 0.9341
 -1.010236 0.1319 Inf -7.662 <.0001
 -0.685642 0.1245 Inf -5.509 <.0001
 -1.217051 0.1260 Inf -9.661 <.0001
 -0.734798 0.1244 Inf -5.908 <.0001
 -1.570596 0.1253 Inf -12.531 <.0001
 -0.476491 0.0481 Inf -9.904 <.0001
 -1.373574 0.1305 Inf -10.527 <.0001
 -1.048980 0.1231 Inf -8.518 <.0001
 -1.580388 0.1249 Inf -12.654 <.0001
 -1.098136 0.1230 Inf -8.928 <.0001
 -1.625257 0.1256 Inf -12.942 <.0001
 -0.531151 0.0485 Inf -10.943 <.0001
 -1.428234 0.1305 Inf -10.940 <.0001
 -1.103640 0.1233 Inf -8.952 <.0001
 -1.635049 0.1253 Inf -13.050 <.0001
 -1.152796 0.1231 Inf -9.362 <.0001
 0.324594 0.1165 Inf 2.785 0.5580
 -0.206815 0.1182 Inf -1.749 0.9971
 0.275438 0.1166 Inf 2.362 0.8649
 0.275555 0.1334 Inf 2.065 0.9683
 1.369661 0.1354 Inf 10.114 <.0001
 0.472578 0.0600 Inf 7.870 <.0001
 0.797172 0.1313 Inf 6.070 <.0001
 0.265763 0.1326 Inf 2.005 0.9783
 0.748015 0.1313 Inf 5.697 <.0001
 -0.310176 0.1298 Inf -2.391 0.8493
 0.783930 0.1318 Inf 5.949 <.0001
 -0.113153 0.0515 Inf -2.199 0.9341
 0.211441 0.1276 Inf 1.657 0.9988
 -0.319968 0.1289 Inf -2.482 0.7934
 0.162285 0.1274 Inf 1.273 1.0000
 -0.673513 0.1285 Inf -5.243 0.0001
 0.420592 0.1306 Inf 3.220 0.2361
 -0.476491 0.0481 Inf -9.904 <.0001
 -0.151897 0.1264 Inf -1.202 1.0000
 -0.683305 0.1280 Inf -5.339 <.0001
 -0.201053 0.1262 Inf -1.593 0.9994
 -0.728174 0.1288 Inf -5.654 <.0001
 0.365932 0.1308 Inf 2.797 0.5483
 -0.531151 0.0485 Inf -10.943 <.0001
 -0.206557 0.1266 Inf -1.631 0.9991
 -0.737966 0.1285 Inf -5.745 <.0001
 -0.255713 0.1264 Inf -2.022 0.9758
 -0.531409 0.1098 Inf -4.840 0.0005
 -0.049156 0.1081 Inf -0.455 1.0000
 -0.049039 0.1258 Inf -0.390 1.0000
 1.045067 0.1278 Inf 8.175 <.0001
 0.147984 0.1309 Inf 1.131 1.0000
 0.472578 0.0600 Inf 7.870 <.0001
 -0.058831 0.1249 Inf -0.471 1.0000
 0.423421 0.1235 Inf 3.428 0.1364
 -0.634770 0.1219 Inf -5.206 0.0001
 0.459336 0.1240 Inf 3.704 0.0580
 -0.437747 0.1272 Inf -3.441 0.1315
 -0.113153 0.0515 Inf -2.199 0.9341
 -0.644562 0.1211 Inf -5.324 <.0001
 -0.162309 0.1195 Inf -1.358 1.0000
 -0.998107 0.1204 Inf -8.289 <.0001
 0.095998 0.1226 Inf 0.783 1.0000
 -0.801084 0.1258 Inf -6.370 <.0001
 -0.476491 0.0481 Inf -9.904 <.0001
 -1.007899 0.1199 Inf -8.406 <.0001
 -0.525647 0.1180 Inf -4.454 0.0031
 -1.052768 0.1207 Inf -8.723 <.0001
 0.041338 0.1228 Inf 0.337 1.0000
 -0.855745 0.1259 Inf -6.799 <.0001
 -0.531151 0.0485 Inf -10.943 <.0001
 -1.062560 0.1203 Inf -8.830 <.0001
 -0.580307 0.1182 Inf -4.910 0.0004
 0.482252 0.1099 Inf 4.389 0.0042
 0.482370 0.1276 Inf 3.780 0.0448
 1.576475 0.1298 Inf 12.149 <.0001
 0.679392 0.1326 Inf 5.122 0.0001
 1.003986 0.1254 Inf 8.005 <.0001
 0.472578 0.0600 Inf 7.870 <.0001
 0.954830 0.1254 Inf 7.615 <.0001
 -0.103361 0.1237 Inf -0.835 1.0000
 0.990745 0.1259 Inf 7.869 <.0001
 0.093662 0.1289 Inf 0.726 1.0000
 0.418256 0.1214 Inf 3.444 0.1303
 -0.113153 0.0515 Inf -2.199 0.9341
 0.369099 0.1213 Inf 3.042 0.3528
 -0.466699 0.1220 Inf -3.825 0.0384
 0.627407 0.1243 Inf 5.047 0.0002
 -0.269676 0.1273 Inf -2.118 0.9569
 0.054918 0.1199 Inf 0.458 1.0000
 -0.476491 0.0481 Inf -9.904 <.0001
 0.005762 0.1197 Inf 0.048 1.0000
 -0.521359 0.1220 Inf -4.273 0.0068
 0.572747 0.1243 Inf 4.610 0.0015
 -0.324336 0.1271 Inf -2.551 0.7450
 0.000258 0.1198 Inf 0.002 1.0000
 -0.531151 0.0485 Inf -10.943 <.0001
 -0.048899 0.1196 Inf -0.409 1.0000
 0.000117 0.1260 Inf 0.001 1.0000
 1.094223 0.1281 Inf 8.545 <.0001
 0.197140 0.1311 Inf 1.504 0.9998
 0.521734 0.1237 Inf 4.216 0.0086
 -0.009675 0.1251 Inf -0.077 1.0000
 0.472578 0.0600 Inf 7.870 <.0001
 -0.585613 0.1222 Inf -4.791 0.0007
 0.508493 0.1243 Inf 4.090 0.0143
 -0.388591 0.1275 Inf -3.048 0.3487
 -0.063996 0.1199 Inf -0.534 1.0000
 -0.595405 0.1214 Inf -4.906 0.0004
 -0.113153 0.0515 Inf -2.199 0.9341
 -0.948951 0.1208 Inf -7.857 <.0001
 0.145155 0.1230 Inf 1.180 1.0000
 -0.751928 0.1261 Inf -5.963 <.0001
 -0.427334 0.1186 Inf -3.604 0.0804
 -0.958743 0.1203 Inf -7.973 <.0001
 -0.476491 0.0481 Inf -9.904 <.0001
 -1.003611 0.1210 Inf -8.292 <.0001
 0.090494 0.1232 Inf 0.735 1.0000
 -0.806589 0.1262 Inf -6.392 <.0001
 -0.481995 0.1187 Inf -4.059 0.0161
 -1.013403 0.1207 Inf -8.396 <.0001
 -0.531151 0.0485 Inf -10.943 <.0001
 1.094106 0.1156 Inf 9.464 <.0001
 0.197023 0.1189 Inf 1.657 0.9988
 0.521617 0.1105 Inf 4.719 0.0009
 -0.009792 0.1123 Inf -0.087 1.0000
 0.472461 0.1106 Inf 4.270 0.0069
 -0.585731 0.0592 Inf -9.896 <.0001
 0.508375 0.1298 Inf 3.916 0.0277
 -0.388708 0.1329 Inf -2.926 0.4422
 -0.064114 0.1254 Inf -0.511 1.0000
 -0.595522 0.1270 Inf -4.689 0.0011
 -0.113270 0.1254 Inf -0.904 1.0000
 -0.949068 0.0563 Inf -16.854 <.0001
 0.145038 0.1286 Inf 1.127 1.0000
 -0.752045 0.1316 Inf -5.715 <.0001
 -0.427451 0.1242 Inf -3.442 0.1309
 -0.958860 0.1260 Inf -7.608 <.0001
 -0.476608 0.1241 Inf -3.840 0.0363
 -1.003729 0.0567 Inf -17.712 <.0001
 0.090377 0.1287 Inf 0.702 1.0000
 -0.806706 0.1316 Inf -6.130 <.0001
 -0.482112 0.1242 Inf -3.880 0.0315
 -1.013520 0.1264 Inf -8.021 <.0001
 -0.531268 0.1242 Inf -4.279 0.0066
 -0.897083 0.1214 Inf -7.392 <.0001
 -0.572489 0.1131 Inf -5.063 0.0002
 -1.103898 0.1149 Inf -9.604 <.0001
 -0.621645 0.1132 Inf -5.492 <.0001
 -1.679836 0.1299 Inf -12.928 <.0001
 -0.585731 0.0592 Inf -9.896 <.0001
 -1.482814 0.1351 Inf -10.977 <.0001
 -1.158220 0.1277 Inf -9.073 <.0001
 -1.689628 0.1294 Inf -13.058 <.0001
 -1.207376 0.1277 Inf -9.457 <.0001
 -2.043174 0.1285 Inf -15.895 <.0001
 -0.949068 0.0563 Inf -16.854 <.0001
 -1.846151 0.1337 Inf -13.803 <.0001
 -1.521557 0.1264 Inf -12.040 <.0001
 -2.052966 0.1283 Inf -15.995 <.0001
 -1.570714 0.1263 Inf -12.432 <.0001
 -2.097834 0.1288 Inf -16.291 <.0001
 -1.003729 0.0567 Inf -17.712 <.0001
 -1.900812 0.1338 Inf -14.205 <.0001
 -1.576218 0.1265 Inf -12.459 <.0001
 -2.107626 0.1287 Inf -16.372 <.0001
 -1.625374 0.1265 Inf -12.851 <.0001
 0.324594 0.1165 Inf 2.785 0.5580
 -0.206815 0.1182 Inf -1.749 0.9971
 0.275438 0.1166 Inf 2.362 0.8649
 -0.782753 0.1328 Inf -5.893 <.0001
 0.311352 0.1349 Inf 2.307 0.8917
 -0.585731 0.0592 Inf -9.896 <.0001
 -0.261137 0.1307 Inf -1.998 0.9792
 -0.792545 0.1322 Inf -5.993 <.0001
 -0.310293 0.1307 Inf -2.375 0.8579
 -1.146091 0.1316 Inf -8.711 <.0001
 -0.051985 0.1338 Inf -0.388 1.0000
 -0.949068 0.0563 Inf -16.854 <.0001
 -0.624474 0.1295 Inf -4.821 0.0006
 -1.155883 0.1313 Inf -8.802 <.0001
 -0.673631 0.1295 Inf -5.204 0.0001
 -1.200751 0.1319 Inf -9.106 <.0001
 -0.106646 0.1340 Inf -0.796 1.0000
 -1.003729 0.0567 Inf -17.712 <.0001
 -0.679135 0.1298 Inf -5.234 0.0001
 -1.210543 0.1318 Inf -9.186 <.0001
 -0.728291 0.1297 Inf -5.616 <.0001
 -0.531409 0.1098 Inf -4.840 0.0005
 -0.049156 0.1081 Inf -0.455 1.0000
 -1.107347 0.1254 Inf -8.829 <.0001
 -0.013241 0.1276 Inf -0.104 1.0000
 -0.910324 0.1308 Inf -6.962 <.0001
 -0.585731 0.0592 Inf -9.896 <.0001
 -1.117139 0.1248 Inf -8.950 <.0001
 -0.634887 0.1231 Inf -5.156 0.0001
 -1.470685 0.1239 Inf -11.866 <.0001
 -0.376579 0.1262 Inf -2.983 0.3974
 -1.273662 0.1293 Inf -9.847 <.0001
 -0.949068 0.0563 Inf -16.854 <.0001
 -1.480477 0.1237 Inf -11.969 <.0001
 -0.998225 0.1217 Inf -8.202 <.0001
 -1.525345 0.1242 Inf -12.281 <.0001
 -0.431240 0.1264 Inf -3.411 0.1432
 -1.328323 0.1294 Inf -10.263 <.0001
 -1.003729 0.0567 Inf -17.712 <.0001
 -1.535137 0.1241 Inf -12.369 <.0001
 -1.052885 0.1219 Inf -8.639 <.0001
 0.482252 0.1099 Inf 4.389 0.0042
 -0.575939 0.1269 Inf -4.538 0.0021
 0.518167 0.1292 Inf 4.011 0.0194
 -0.378916 0.1322 Inf -2.867 0.4903
 -0.054322 0.1247 Inf -0.436 1.0000
 -0.585731 0.0592 Inf -9.896 <.0001
 -0.103478 0.1246 Inf -0.830 1.0000
 -0.939276 0.1252 Inf -7.501 <.0001
 0.154829 0.1276 Inf 1.213 1.0000
 -0.742254 0.1306 Inf -5.684 <.0001
 -0.417660 0.1231 Inf -3.393 0.1505
 -0.949068 0.0563 Inf -16.854 <.0001
 -0.466816 0.1230 Inf -3.795 0.0427
 -0.993937 0.1252 Inf -7.937 <.0001
 0.100169 0.1276 Inf 0.785 1.0000
 -0.796914 0.1304 Inf -6.110 <.0001
 -0.472320 0.1230 Inf -3.840 0.0364
 -1.003729 0.0567 Inf -17.712 <.0001
 -0.521476 0.1229 Inf -4.242 0.0077
 -1.058191 0.1256 Inf -8.425 <.0001
 0.035915 0.1278 Inf 0.281 1.0000
 -0.861168 0.1309 Inf -6.578 <.0001
 -0.536574 0.1233 Inf -4.351 0.0049
 -1.067983 0.1250 Inf -8.545 <.0001
 -0.585731 0.0592 Inf -9.896 <.0001
 -1.421529 0.1242 Inf -11.447 <.0001
 -0.327423 0.1265 Inf -2.588 0.7175
 -1.224506 0.1296 Inf -9.451 <.0001
 -0.899912 0.1220 Inf -7.375 <.0001
 -1.431320 0.1239 Inf -11.550 <.0001
 -0.949068 0.0563 Inf -16.854 <.0001
 -1.476189 0.1244 Inf -11.863 <.0001
 -0.382083 0.1267 Inf -3.016 0.3725
 -1.279166 0.1297 Inf -9.866 <.0001
 -0.954572 0.1222 Inf -7.812 <.0001
 -1.485981 0.1244 Inf -11.950 <.0001
 -1.003729 0.0567 Inf -17.712 <.0001
 1.094106 0.1156 Inf 9.464 <.0001
 0.197023 0.1189 Inf 1.657 0.9988
 0.521617 0.1105 Inf 4.719 0.0009
 -0.009792 0.1123 Inf -0.087 1.0000
 0.472461 0.1106 Inf 4.270 0.0069
 -0.363338 0.0469 Inf -7.748 <.0001
 0.730768 0.1249 Inf 5.852 <.0001
 -0.166315 0.1278 Inf -1.301 1.0000
 0.158279 0.1202 Inf 1.316 1.0000
 -0.373130 0.1221 Inf -3.057 0.3424
 0.109123 0.1203 Inf 0.907 1.0000
 -0.417998 0.0473 Inf -8.833 <.0001
 0.676108 0.1250 Inf 5.411 <.0001
 -0.220975 0.1278 Inf -1.729 0.9976
 0.103619 0.1203 Inf 0.861 1.0000
 -0.427790 0.1224 Inf -3.495 0.1123
 0.054462 0.1203 Inf 0.453 1.0000
 -0.897083 0.1214 Inf -7.392 <.0001
 -0.572489 0.1131 Inf -5.063 0.0002
 -1.103898 0.1149 Inf -9.604 <.0001
 -0.621645 0.1132 Inf -5.492 <.0001
 -1.457443 0.1246 Inf -11.693 <.0001
 -0.363338 0.0469 Inf -7.748 <.0001
 -1.260421 0.1300 Inf -9.696 <.0001
 -0.935827 0.1224 Inf -7.643 <.0001
 -1.467235 0.1244 Inf -11.795 <.0001
 -0.984983 0.1225 Inf -8.041 <.0001
 -1.512104 0.1249 Inf -12.108 <.0001
 -0.417998 0.0473 Inf -8.833 <.0001
 -1.315081 0.1301 Inf -10.111 <.0001
 -0.990487 0.1226 Inf -8.080 <.0001
 -1.521896 0.1248 Inf -12.195 <.0001
 -1.039643 0.1226 Inf -8.477 <.0001
 0.324594 0.1165 Inf 2.785 0.5580
 -0.206815 0.1182 Inf -1.749 0.9971
 0.275438 0.1166 Inf 2.362 0.8649
 -0.560361 0.1278 Inf -4.384 0.0043
 0.533745 0.1302 Inf 4.099 0.0138
 -0.363338 0.0469 Inf -7.748 <.0001
 -0.038744 0.1258 Inf -0.308 1.0000
 -0.570152 0.1275 Inf -4.470 0.0029
 -0.087900 0.1258 Inf -0.699 1.0000
 -0.615021 0.1281 Inf -4.799 0.0006
 0.479085 0.1304 Inf 3.673 0.0644
 -0.417998 0.0473 Inf -8.833 <.0001
 -0.093404 0.1260 Inf -0.741 1.0000
 -0.624813 0.1280 Inf -4.881 0.0004
 -0.142561 0.1260 Inf -1.131 1.0000
 -0.531409 0.1098 Inf -4.840 0.0005
 -0.049156 0.1081 Inf -0.455 1.0000
 -0.884954 0.1199 Inf -7.379 <.0001
 0.209151 0.1224 Inf 1.709 0.9980
 -0.687932 0.1255 Inf -5.482 <.0001
 -0.363338 0.0469 Inf -7.748 <.0001
 -0.894746 0.1196 Inf -7.480 <.0001
 -0.412494 0.1177 Inf -3.503 0.1095
 -0.939615 0.1202 Inf -7.817 <.0001
 0.154491 0.1226 Inf 1.261 1.0000
 -0.742592 0.1256 Inf -5.913 <.0001
 -0.417998 0.0473 Inf -8.833 <.0001
 -0.949407 0.1201 Inf -7.908 <.0001
 -0.467154 0.1179 Inf -3.962 0.0233
 0.482252 0.1099 Inf 4.389 0.0042
 -0.353546 0.1213 Inf -2.914 0.4519
 0.740560 0.1239 Inf 5.977 <.0001
 -0.156523 0.1268 Inf -1.234 1.0000
 0.168071 0.1192 Inf 1.410 0.9999
 -0.363338 0.0469 Inf -7.748 <.0001
 0.118915 0.1192 Inf 0.998 1.0000
 -0.408206 0.1213 Inf -3.364 0.1629
 0.685900 0.1238 Inf 5.539 <.0001
 -0.211183 0.1267 Inf -1.667 0.9987
 0.113411 0.1191 Inf 0.952 1.0000
 -0.417998 0.0473 Inf -8.833 <.0001
 0.064254 0.1191 Inf 0.540 1.0000
 -0.835798 0.1201 Inf -6.961 <.0001
 0.258308 0.1226 Inf 2.108 0.9594
 -0.638775 0.1256 Inf -5.085 0.0002
 -0.314181 0.1179 Inf -2.666 0.6567
 -0.845590 0.1198 Inf -7.061 <.0001
 -0.363338 0.0469 Inf -7.748 <.0001
 -0.890459 0.1203 Inf -7.399 <.0001
 0.203647 0.1227 Inf 1.659 0.9988
 -0.693436 0.1257 Inf -5.516 <.0001
 -0.368842 0.1180 Inf -3.125 0.2955
 -0.900250 0.1202 Inf -7.490 <.0001
 -0.417998 0.0473 Inf -8.833 <.0001
 1.094106 0.1156 Inf 9.464 <.0001
 0.197023 0.1189 Inf 1.657 0.9988
 0.521617 0.1105 Inf 4.719 0.0009
 -0.009792 0.1123 Inf -0.087 1.0000
 0.472461 0.1106 Inf 4.270 0.0069
 -0.054660 0.0433 Inf -1.262 1.0000
 1.039446 0.1234 Inf 8.424 <.0001
 0.142363 0.1264 Inf 1.126 1.0000
 0.466956 0.1186 Inf 3.936 0.0257
 -0.064452 0.1205 Inf -0.535 1.0000
 0.417800 0.1187 Inf 3.519 0.1044
 -0.897083 0.1214 Inf -7.392 <.0001
 -0.572489 0.1131 Inf -5.063 0.0002
 -1.103898 0.1149 Inf -9.604 <.0001
 -0.621645 0.1132 Inf -5.492 <.0001
 -1.148766 0.1235 Inf -9.299 <.0001
 -0.054660 0.0433 Inf -1.262 1.0000
 -0.951743 0.1288 Inf -7.391 <.0001
 -0.627149 0.1211 Inf -5.180 0.0001
 -1.158558 0.1231 Inf -9.413 <.0001
 -0.676306 0.1212 Inf -5.581 <.0001
 0.324594 0.1165 Inf 2.785 0.5580
 -0.206815 0.1182 Inf -1.749 0.9971
 0.275438 0.1166 Inf 2.362 0.8649
 -0.251683 0.1267 Inf -1.986 0.9809
 0.842423 0.1289 Inf 6.533 <.0001
 -0.054660 0.0433 Inf -1.262 1.0000
 0.269934 0.1244 Inf 2.170 0.9432
 -0.261475 0.1262 Inf -2.071 0.9672
 0.220777 0.1245 Inf 1.774 0.9963
 -0.531409 0.1098 Inf -4.840 0.0005
 -0.049156 0.1081 Inf -0.455 1.0000
 -0.576277 0.1188 Inf -4.850 0.0005
 0.517829 0.1211 Inf 4.276 0.0067
 -0.379254 0.1243 Inf -3.052 0.3459
 -0.054660 0.0433 Inf -1.262 1.0000
 -0.586069 0.1183 Inf -4.954 0.0003
 -0.103817 0.1164 Inf -0.892 1.0000
 0.482252 0.1099 Inf 4.389 0.0042
 -0.044868 0.1202 Inf -0.373 1.0000
 1.049237 0.1226 Inf 8.558 <.0001
 0.152154 0.1256 Inf 1.212 1.0000
 0.476748 0.1178 Inf 4.048 0.0168
 -0.054660 0.0433 Inf -1.262 1.0000
 0.427592 0.1178 Inf 3.628 0.0744
 -0.527121 0.1189 Inf -4.433 0.0034
 0.566985 0.1212 Inf 4.677 0.0011
 -0.330098 0.1243 Inf -2.655 0.6654
 -0.005504 0.1164 Inf -0.047 1.0000
 -0.536913 0.1184 Inf -4.535 0.0022
 -0.054660 0.0433 Inf -1.262 1.0000
 1.094106 0.1156 Inf 9.464 <.0001
 0.197023 0.1189 Inf 1.657 0.9988
 0.521617 0.1105 Inf 4.719 0.0009
 -0.009792 0.1123 Inf -0.087 1.0000
 0.472461 0.1106 Inf 4.270 0.0069
 -0.897083 0.1214 Inf -7.392 <.0001
 -0.572489 0.1131 Inf -5.063 0.0002
 -1.103898 0.1149 Inf -9.604 <.0001
 -0.621645 0.1132 Inf -5.492 <.0001
 0.324594 0.1165 Inf 2.785 0.5580
 -0.206815 0.1182 Inf -1.749 0.9971
 0.275438 0.1166 Inf 2.362 0.8649
 -0.531409 0.1098 Inf -4.840 0.0005
 -0.049156 0.1081 Inf -0.455 1.0000
 0.482252 0.1099 Inf 4.389 0.0042

Results are given on the log (not the response) scale.
P value adjustment: tukey method for comparing a family of 30 estimates

lsmeans(AntennaeFeeding_glmmTMB1, pairwise ~ Focal_Treatment * Rival_Treatment, adjust="tukey")

$lsmeans
 Focal_Treatment Rival_Treatment lsmean SE df asymp.LCL asymp.UCL
 AntennaeRemoved Single 1.440 0.0802 Inf 1.283 1.597
 WT Single 0.346 0.0842 Inf 0.181 0.511
 AntennaeRemoved WingClip 1.243 0.0883 Inf 1.070 1.416
 WT WingClip 0.919 0.0767 Inf 0.768 1.069
 AntennaeRemoved WingRemoved 1.450 0.0790 Inf 1.295 1.605
 WT WingRemoved 0.968 0.0768 Inf 0.817 1.118

Results are averaged over the levels of: Week
Results are given on the log (not the response) scale.
Confidence level used: 0.95

$contrasts
 contrast estimate SE df
 AntennaeRemoved Single - WT Single 1.09411 0.116 Inf
 AntennaeRemoved Single - AntennaeRemoved WingClip 0.19702 0.119 Inf
 AntennaeRemoved Single - WT WingClip 0.52162 0.111 Inf
 AntennaeRemoved Single - AntennaeRemoved WingRemoved -0.00979 0.112 Inf
 AntennaeRemoved Single - WT WingRemoved 0.47246 0.111 Inf
 WT Single - AntennaeRemoved WingClip -0.89708 0.121 Inf
 WT Single - WT WingClip -0.57249 0.113 Inf
 WT Single - AntennaeRemoved WingRemoved -1.10390 0.115 Inf
 WT Single - WT WingRemoved -0.62165 0.113 Inf
 AntennaeRemoved WingClip - WT WingClip 0.32459 0.117 Inf
 AntennaeRemoved WingClip - AntennaeRemoved WingRemoved -0.20681 0.118 Inf
 AntennaeRemoved WingClip - WT WingRemoved 0.27544 0.117 Inf
 WT WingClip - AntennaeRemoved WingRemoved -0.53141 0.110 Inf
 WT WingClip - WT WingRemoved -0.04916 0.108 Inf
 AntennaeRemoved WingRemoved - WT WingRemoved 0.48225 0.110 Inf
 z.ratio p.value
 9.464 <.0001
 1.657 0.5606
 4.719 <.0001
 -0.087 1.0000
 4.270 0.0003
 -7.392 <.0001
 -5.063 <.0001
 -9.604 <.0001
 -5.492 <.0001
 2.785 0.0598
 -1.749 0.4989
 2.362 0.1699
 -4.840 <.0001
 -0.455 0.9976
 4.389 0.0002

Results are averaged over the levels of: Week
Results are given on the log (not the response) scale.
P value adjustment: tukey method for comparing a family of 6 estimates

lsmeans(AntennaeFeeding_glmmTMB1, pairwise ~ Focal_Treatment, adjust="tukey")

NOTE: Results may be misleading due to involvement in interactions

$lsmeans
 Focal_Treatment lsmean SE df asymp.LCL asymp.UCL
 AntennaeRemoved 1.378 0.0479 Inf 1.284 1.472
 WT 0.744 0.0463 Inf 0.653 0.835

Results are averaged over the levels of: Rival_Treatment, Week
Results are given on the log (not the response) scale.
Confidence level used: 0.95

$contrasts
 contrast estimate SE df z.ratio p.value
 AntennaeRemoved - WT 0.634 0.0659 Inf 9.617 <.0001

Results are averaged over the levels of: Rival_Treatment, Week
Results are given on the log (not the response) scale.

lsmeans(AntennaeFeeding_glmmTMB1, pairwise ~ Rival_Treatment, adjust="tukey")

NOTE: Results may be misleading due to involvement in interactions

$lsmeans
 Rival_Treatment lsmean SE df asymp.LCL asymp.UCL
 Single 0.893 0.0584 Inf 0.779 1.01
 WingClip 1.081 0.0587 Inf 0.966 1.20
 WingRemoved 1.209 0.0553 Inf 1.101 1.32

Results are averaged over the levels of: Focal_Treatment, Week
Results are given on the log (not the response) scale.
Confidence level used: 0.95

$contrasts
 contrast estimate SE df z.ratio p.value
 Single - WingClip -0.188 0.0820 Inf -2.288 0.0574
 Single - WingRemoved -0.316 0.0797 Inf -3.959 0.0002
 WingClip - WingRemoved -0.128 0.0801 Inf -1.598 0.2464

Results are averaged over the levels of: Focal_Treatment, Week
Results are given on the log (not the response) scale.
P value adjustment: tukey method for comparing a family of 3 estimates

lsmeans(AntennaeFeeding_glmmTMB1, pairwise ~ Week, adjust="tukey")

$lsmeans
 Week lsmean SE df asymp.LCL asymp.UCL
 1 0.931 0.0472 Inf 0.839 1.024
 2 0.459 0.0557 Inf 0.350 0.568
 3 1.045 0.0463 Inf 0.954 1.135
 4 1.408 0.0425 Inf 1.325 1.491
 5 1.463 0.0427 Inf 1.379 1.546

Results are averaged over the levels of: Focal_Treatment, Rival_Treatment
Results are given on the log (not the response) scale.
Confidence level used: 0.95

$contrasts
 contrast estimate SE df z.ratio p.value
 Week1 - Week2 0.4726 0.0600 Inf 7.870 <.0001
 Week1 - Week3 -0.1132 0.0515 Inf -2.199 0.1799
 Week1 - Week4 -0.4765 0.0481 Inf -9.904 <.0001
 Week1 - Week5 -0.5312 0.0485 Inf -10.943 <.0001
 Week2 - Week3 -0.5857 0.0592 Inf -9.896 <.0001
 Week2 - Week4 -0.9491 0.0563 Inf -16.854 <.0001
 Week2 - Week5 -1.0037 0.0567 Inf -17.712 <.0001
 Week3 - Week4 -0.3633 0.0469 Inf -7.748 <.0001
 Week3 - Week5 -0.4180 0.0473 Inf -8.833 <.0001
 Week4 - Week5 -0.0547 0.0433 Inf -1.262 0.7148

Results are averaged over the levels of: Focal_Treatment, Rival_Treatment
Results are given on the log (not the response) scale.
P value adjustment: tukey method for comparing a family of 5 estimates

##Antennae Food Boxplot
Antennae_Food$PercentSuccess <- Antennae_Food$Success / 10
Antennae_Food_subset <- subset(Antennae_Food, PercentSuccess > 0)
Antennae_Food_subset$Rival_Treatment <- factor(Antennae_Food_subset$Rival_Treatment, levels = c("Single", "WingClip", "WingRemoved"))
Antennae_Food_subset$Focal_Treatment <- factor(Antennae_Food_subset$Focal_Treatment, levels = c("WT", "AntennaeRemoved"))
Antennae_Food_subset$Interaction <- interaction(Antennae_Food_subset$Focal_Treatment,Antennae_Food_subset$Rival_Treatment)
Antennae_Food_subset$Interaction <- factor(Antennae_Food_subset$Interaction, levels = c("WT.Single", "WT.WingClip", "WT.WingRemoved", "AntennaeRemoved.Single", "AntennaeRemoved.WingClip", "AntennaeRemoved.WingRemoved"))

Antennae_Food_Summary <- summarySE(Antennae_Food_subset,
 measurevar = "PercentSuccess",
 groupvars = c("Week", "Interaction", "Focal_Treatment", "Rival_Treatment"))

Antennae_Food_Line <- ggplot(Antennae_Food_Summary) +
 aes(
 x = Week,
 y = PercentSuccess,
 colour = Interaction,
 group = Interaction
 ) +
 scale_colour_manual(name = "Treatment",
 labels = c("Antennae Intact kept alone",
 "Antennae Intact kept with Wing Clipped Rival",
 "Antennae Intact kept with Wing Removed Rival",
 "Antennae Removed kept alone",
 "Antennae Removed kept with Wing Clipped Rival",
 "Antennae Removed kept with Wing Removed Rival"),
 values = c("black",
 "grey70",
 "grey42",
 "black",
 "grey70",
 "grey42")) +
 geom_line(aes(linetype = Interaction), size = 3) +
 geom_point() +
 geom_errorbar(aes(ymin=PercentSuccess-se, ymax=PercentSuccess+se), width=0.2, size = 1.5) +
 scale_linetype_manual(name = "Treatment",
 labels = c("Antennae Intact kept alone",
 "Antennae Intact kept with Wing Clipped Rival",
 "Antennae Intact kept with Wing Removed Rival",
 "Antennae Removed kept alone",
 "Antennae Removed kept with Wing Clipped Rival",
 "Antennae Removed kept with Wing Removed Rival"),
 values = c("solid", "solid", "solid", "dotdash", "dotdash", "dotdash")) +
 labs(
 x = "Week post Eclosion",
 y = "Proportion of Time Spent on Food"
 ) +
 ylim(0, 1.05) +
 theme_bw() +
 guides(colour = guide_legend(override.aes = list(linetype = c("solid", "solid", "solid", "dotdash", "dotdash", "dotdash"))))

Antennae_Food_Line_Plot <- Antennae_Food_Line + theme_bw() + theme(panel.grid.major = element_blank(), panel.grid.minor = element_blank()) +
 theme(axis.text=element_text(size=55, face="bold", colour="black"),
 axis.title=element_text(size=75, face="bold", colour="black"),
 legend.text=element_text(size=55, face="bold", colour="black"),
 legend.title=element_text(size=75, face="bold", colour="black"),
 strip.text=element_text(size=75, face="bold", colour="black")) + theme(axis.line = element_line(size = 5), panel.border = element_rect(size = 5)) + theme( axis.ticks.length = unit(0.5, "cm"), axis.ticks = element_line(size = 5))
Antennae_Food_Line_Plot


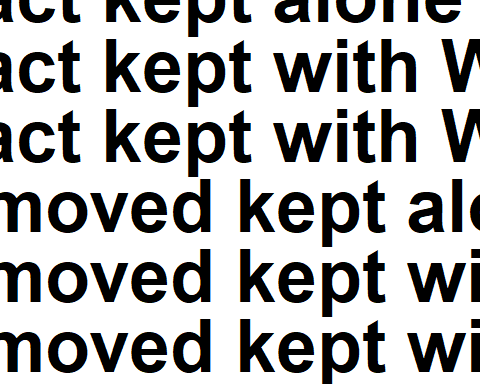


###Antennae Walking
##Antennae Walking Model
Antennae_Walking$Week <- as.factor(Antennae_Walking$Week)
AntennaeWalking_glmmTMB1 <- glmmTMB(Success ~ Focal_Treatment * Rival_Treatment + Week + (1|Fly_ID), family=poisson(link = "log"), data = Antennae_Walking)
AntennaeWalking_glmmTMB2 <- glmmTMB(Success ~ Focal_Treatment + Rival_Treatment + Week + (1|Fly_ID), family=poisson(link = "log"), data = Antennae_Walking)
anova(AntennaeWalking_glmmTMB1, AntennaeWalking_glmmTMB2, test="Chi")

Data: Antennae_Walking
Models:
AntennaeWalking_glmmTMB2: Success ~ Focal_Treatment + Rival_Treatment + Week + (1 | Fly_ID), zi=~0, disp=~1
AntennaeWalking_glmmTMB1: Success ~ Focal_Treatment * Rival_Treatment + Week + (1 | Fly_ID), zi=~0, disp=~1
 Df AIC BIC logLik deviance Chisq Chi Df
AntennaeWalking_glmmTMB2 9 4813.7 4859.7 -2397.9 4795.7
AntennaeWalking_glmmTMB1 11 4812.1 4868.4 -2395.1 4790.1 5.6009 2
 Pr(>Chisq)
AntennaeWalking_glmmTMB2
AntennaeWalking_glmmTMB1 0.06078 .
---
Signif. codes: 0 '***' 0.001 '**' 0.01 '*' 0.05 '.' 0.1 ' ' 1

AntennaeWalking_glmmTMB3 <- glmmTMB(Success ~ Focal_Treatment + Week + (1|Fly_ID), family=poisson(link = "log"), data = Antennae_Walking)
anova(AntennaeWalking_glmmTMB2, AntennaeWalking_glmmTMB3, test="Chi")

Data: Antennae_Walking
Models:
AntennaeWalking_glmmTMB3: Success ~ Focal_Treatment + Week + (1 | Fly_ID), zi=~0, disp=~1
AntennaeWalking_glmmTMB2: Success ~ Focal_Treatment + Rival_Treatment + Week + (1 | Fly_ID), zi=~0, disp=~1
 Df AIC BIC logLik deviance Chisq Chi Df
AntennaeWalking_glmmTMB3 7 4835.9 4871.7 -2410.9 4821.9
AntennaeWalking_glmmTMB2 9 4813.7 4859.7 -2397.9 4795.7 26.158 2
 Pr(>Chisq)
AntennaeWalking_glmmTMB3
AntennaeWalking_glmmTMB2 2.088e-06 ***
---
Signif. codes: 0 '***' 0.001 '**' 0.01 '*' 0.05 '.' 0.1 ' ' 1

AntennaeWalking_glmmTMB4 <- glmmTMB(Success ~ Rival_Treatment + Week + (1|Fly_ID), family=poisson(link = "log"), data = Antennae_Walking)
anova(AntennaeWalking_glmmTMB2, AntennaeWalking_glmmTMB4, test="Chi")

Data: Antennae_Walking
Models:
AntennaeWalking_glmmTMB4: Success ~ Rival_Treatment + Week + (1 | Fly_ID), zi=~0, disp=~1
AntennaeWalking_glmmTMB2: Success ~ Focal_Treatment + Rival_Treatment + Week + (1 | Fly_ID), zi=~0, disp=~1
 Df AIC BIC logLik deviance Chisq Chi Df
AntennaeWalking_glmmTMB4 8 4816.3 4857.2 -2400.2 4800.3
AntennaeWalking_glmmTMB2 9 4813.7 4859.7 -2397.9 4795.7 4.5979 1
 Pr(>Chisq)
AntennaeWalking_glmmTMB4
AntennaeWalking_glmmTMB2 0.03201 *
---
Signif. codes: 0 '***' 0.001 '**' 0.01 '*' 0.05 '.' 0.1 ' ' 1

AntennaeWalking_glmmTMB5 <- glmmTMB(Success ~ Focal_Treatment + Rival_Treatment + (1|Fly_ID), family=poisson(link = "log"), data = Antennae_Walking)
anova(AntennaeWalking_glmmTMB2, AntennaeWalking_glmmTMB5, test="Chi")

Data: Antennae_Walking
Models:
AntennaeWalking_glmmTMB5: Success ~ Focal_Treatment + Rival_Treatment + (1 | Fly_ID), zi=~0, disp=~1
AntennaeWalking_glmmTMB2: Success ~ Focal_Treatment + Rival_Treatment + Week + (1 | Fly_ID), zi=~0, disp=~1
 Df AIC BIC logLik deviance Chisq Chi Df
AntennaeWalking_glmmTMB5 5 4977.1 5002.6 -2483.5 4967.1
AntennaeWalking_glmmTMB2 9 4813.7 4859.7 -2397.9 4795.7 171.34 4
 Pr(>Chisq)
AntennaeWalking_glmmTMB5
AntennaeWalking_glmmTMB2 < 2.2e-16 ***
---
Signif. codes: 0 '***' 0.001 '**' 0.01 '*' 0.05 '.' 0.1 ' ' 1

AntennaeWalking_glmmTMB_null <- glmmTMB(Success ~ (1|Fly_ID), family=poisson(link = "log"), data = Antennae_Walking)
anova(AntennaeWalking_glmmTMB1, AntennaeWalking_glmmTMB_null, test="Chi")

Data: Antennae_Walking
Models:
AntennaeWalking_glmmTMB_null: Success ~ (1 | Fly_ID), zi=~0, disp=~1
AntennaeWalking_glmmTMB1: Success ~ Focal_Treatment * Rival_Treatment + Week + (1 | Fly_ID), zi=~0, disp=~1
 Df AIC BIC logLik deviance Chisq Chi Df
AntennaeWalking_glmmTMB_null 2 5001.5 5011.7 -2498.8 4997.5
AntennaeWalking_glmmTMB1 11 4812.1 4868.4 -2395.1 4790.1 207.37 9
 Pr(>Chisq)
AntennaeWalking_glmmTMB_null
AntennaeWalking_glmmTMB1 < 2.2e-16 ***
---
Signif. codes: 0 '***' 0.001 '**' 0.01 '*' 0.05 '.' 0.1 ' ' 1

lsmeans(AntennaeWalking_glmmTMB2, pairwise ~ Focal_Treatment + Rival_Treatment * Week, adjust="tukey")

$lsmeans
 Focal_Treatment Rival_Treatment Week lsmean SE df asymp.LCL asymp.UCL
 AntennaeRemoved Single 1 1.001 0.0947 Inf 0.8152 1.186
 WT Single 1 1.190 0.0879 Inf 1.0181 1.363
 AntennaeRemoved WingClip 1 0.632 0.0992 Inf 0.4380 0.827
 WT WingClip 1 0.822 0.0917 Inf 0.6422 1.002
 AntennaeRemoved WingRemoved 1 0.458 0.0972 Inf 0.2676 0.649
 WT WingRemoved 1 0.648 0.0931 Inf 0.4653 0.830
 AntennaeRemoved Single 2 1.290 0.0930 Inf 1.1081 1.473
 WT Single 2 1.480 0.0862 Inf 1.3110 1.649
 AntennaeRemoved WingClip 2 0.922 0.0974 Inf 0.7309 1.113
 WT WingClip 2 1.112 0.0901 Inf 0.9350 1.288
 AntennaeRemoved WingRemoved 2 0.748 0.0954 Inf 0.5607 0.935
 WT WingRemoved 2 0.937 0.0913 Inf 0.7583 1.116
 AntennaeRemoved Single 3 0.754 0.0973 Inf 0.5637 0.945
 WT Single 3 0.944 0.0908 Inf 0.7661 1.122
 AntennaeRemoved WingClip 3 0.386 0.1016 Inf 0.1869 0.585
 WT WingClip 3 0.576 0.0944 Inf 0.3905 0.761
 AntennaeRemoved WingRemoved 3 0.212 0.0995 Inf 0.0166 0.407
 WT WingRemoved 3 0.401 0.0956 Inf 0.2140 0.589
 AntennaeRemoved Single 4 0.674 0.0984 Inf 0.4814 0.867
 WT Single 4 0.864 0.0920 Inf 0.6837 1.044
 AntennaeRemoved WingClip 4 0.306 0.1027 Inf 0.1047 0.507
 WT WingClip 4 0.496 0.0956 Inf 0.3082 0.683
 AntennaeRemoved WingRemoved 4 0.132 0.1008 Inf -0.0659 0.329
 WT WingRemoved 4 0.321 0.0969 Inf 0.1314 0.511
 AntennaeRemoved Single 5 0.790 0.0977 Inf 0.5988 0.982
 WT Single 5 0.980 0.0911 Inf 0.8014 1.158
 AntennaeRemoved WingClip 5 0.422 0.1018 Inf 0.2223 0.621
 WT WingClip 5 0.612 0.0946 Inf 0.4260 0.797
 AntennaeRemoved WingRemoved 5 0.248 0.1001 Inf 0.0514 0.444
 WT WingRemoved 5 0.437 0.0961 Inf 0.2489 0.626

Results are given on the log (not the response) scale.
Confidence level used: 0.95

$contrasts
 contrast estimate
[truncated: 214,791 more chars]
